# Asymmetric (3 + 3) and (4 + 2) Annulation Reactions of 2,3-Dioxopyrrolidines with 3-Alkylidene Oxindoles to Construct Diverse Chiral Heterocyclic Frameworks

**DOI:** 10.1021/acs.joc.4c00933

**Published:** 2024-06-08

**Authors:** Shi-Hang Huang, I-Ting Chen, Jeng-Liang Han

**Affiliations:** Department of Chemistry, National Chung Hsing University, Taichung City 40227, Taiwan, R.O.C.

## Abstract

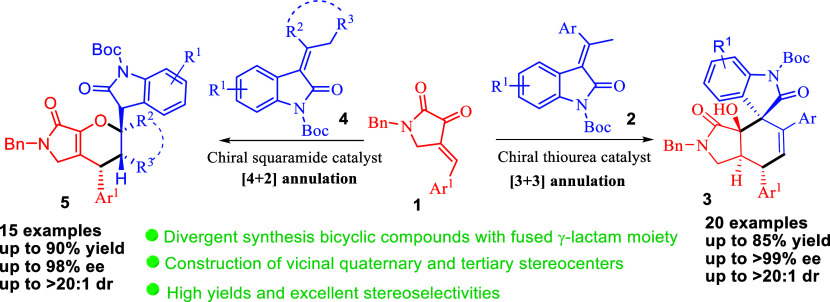

Two substrate-controlled regiodivergent annulation protocols for 2,3-dioxopyrrolidines with 3-alkylidene oxindoles have been developed, which furnished a series of fused dihydropyrrolidone derivatives in high yields with excellent stereoselectivities. Plausible mechanistic pathways for both annulation reactions are proposed that [3 + 3] annulation reaction involves vinylogous Michael addition followed by intramolecular aldol cyclization, while [4 + 2] annulation reaction occurs through a vinylogous Michael addition and a subsequent intramolecular *oxa*-Michael cyclization.

## Introduction

**Figure 1:**
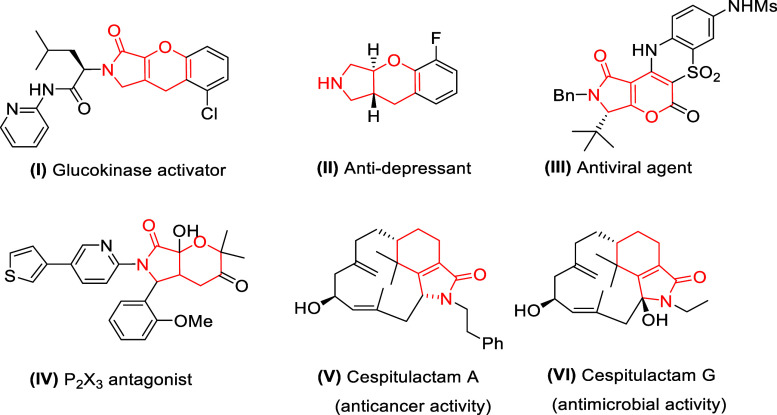
Selected man-made bioactive products and natural products containing a fused γ-lactam moiety.

The development of efficient asymmetric routes for the synthesis of privileged heterocyclic pharmacophores is the most promising drug design strategy in organic chemistry as well as medicinal chemistry.^1^ Among these, multifunctionalized bicyclic compounds with 5–6 fused rings, especially those with a γ-lactam or pyrrolidine moiety, are core structures in many man-made bioactive molecules (Figure 1, I–IV) and natural products (Figure 1, V and VI). For example, compounds (I), (II), (III), and (IV) are proved to behave as glucokinase activators,^2^ antidepressants,^3^ antiviral agents,^4^ and P_2_X_3_ receptor antagonists,^5^ respectively. On the other hand, natural products cespitulactam A (V) and cespitulactam G (VI) showed anticancer and antimicrobial activities, respectively.^6^

**Scheme 1:**
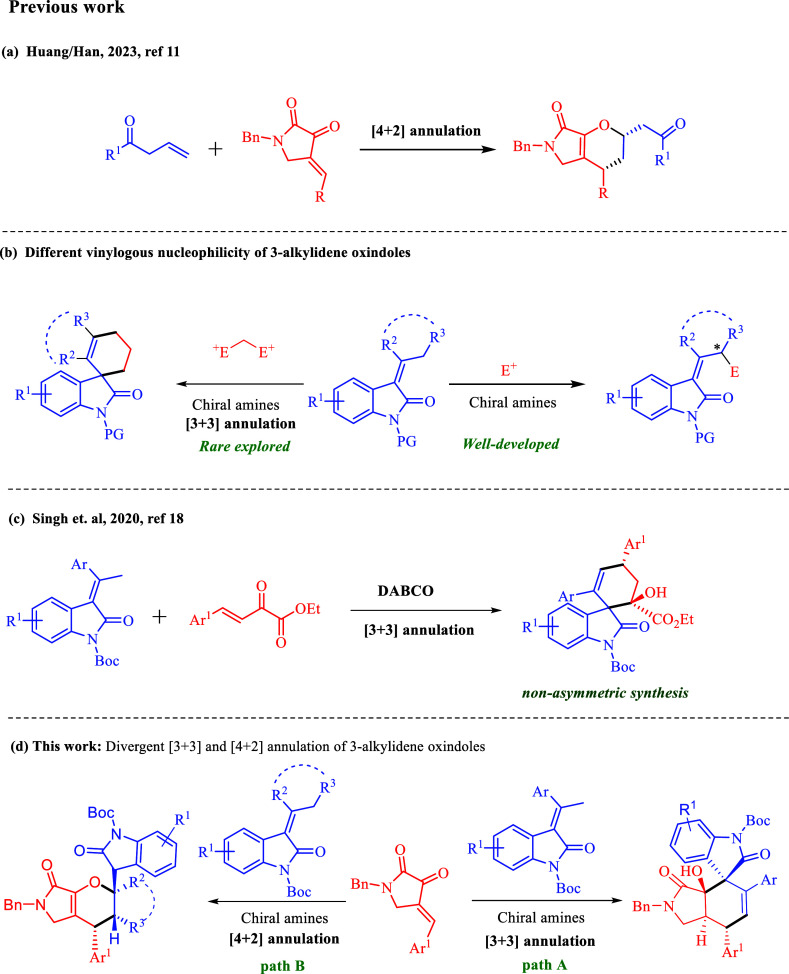
Annulation Reactions of Different Vinylogous Donors

Therefore, the design of efficient catalytic reaction to construct enantioenriched fused γ-lactam or pyrrolidine derivatives is important and has drawn the attention of synthetic chemists.^7^ Most of the reported methods involved an [4 + 2] annulation reaction strategy using oxidative NHC organocatalysts,^8^ amine catalysts,^9^ bifunctional amine catalysts,^10^ and phosphine catalysts.^11^ Recently, our group reported a [4 + 2] annulation reaction between 2,3-dioxopyrrolidines and allyl aryl ketones catalyzed by a quinine-derived squaramide catalyst with moderate diastereoselectivity (Scheme 1a).^12^ Despite several efforts being made, the development of an elegant strategy for the asymmetric synthesis of enantioenriched fused γ-lactam or pyrrolidine derivatives is still highly demand.

On the other hand, 3-alkylidene oxindoles are found in several natural products and pharmaceutical compounds with useful biological activities.^13^ 3-Alkylidene oxindoles also have been documented as a vinylogous nucleophile to functionalize at the γ-position and participate in Michael addition,^14^ vinylogous aldol,^15^ vinylogous Michael–Michael cascade,^16^ and vinylogous aldol-cyclization/lactonization cascade reactions^17^ employing hydrogen-bonding organocatalysts. However, the reactions between 3-alkylidene oxindoles, which acted as a C3-bis-nucleophile, and bis-electrophiles are rare (Scheme 1b). To the best our knowledge, Singh and co-workers reported the only one example that a [3 + 3] annulation reaction of 3-alkylidene-2-oxindole and β,γ-unsaturated α-keto esters could be achieved with DABCO as the catalyst (Scheme 1c).^18^ Hence, designing an asymmetric [3 + 3] annulation reaction between 3-alkylidene-2-oxindole and C3-biselectrophiles has remained unexplored and demands attention.

In continuation of our ongoing research toward the development of (asymmetric) organocatalytic annulation using vinylogous nucleophiles,^19^ we envisioned that the electrophilic properties of 2,3-dioxopyrrolidines could serve as a bis-electrophile for [3 + 3] annulation reaction if a vinylogous nucleophile could be identified as a 3C bis-nucleophile.

In this regard, we recognized that 3-alkylidene oxindoles can be suitable 3C synthons to react with 2,3-dioxopyrrolidines to access enantioenriched multifunctionalized bicyclic compounds with 5–6 fused rings (Scheme 1d, path A). To our surprise, unprecedented fused tricyclic pyran derivatives with an oxindole scaffold were obtained via a [4 + 2] annulation reaction of 2,3-dioxopyrrolidines with dialkyl- or ring-substituted 3-alkylidene oxindoles (Scheme 1d, path B).^20^ Therefore, we herein describe a substrate-controlled regiodivergent protocol for these two interesting annulation reactions, their substrate scope, and a mechanistic hypothesis for rationalization of the observed regioselectivity.

## Results and Discussion

**Scheme 2:**
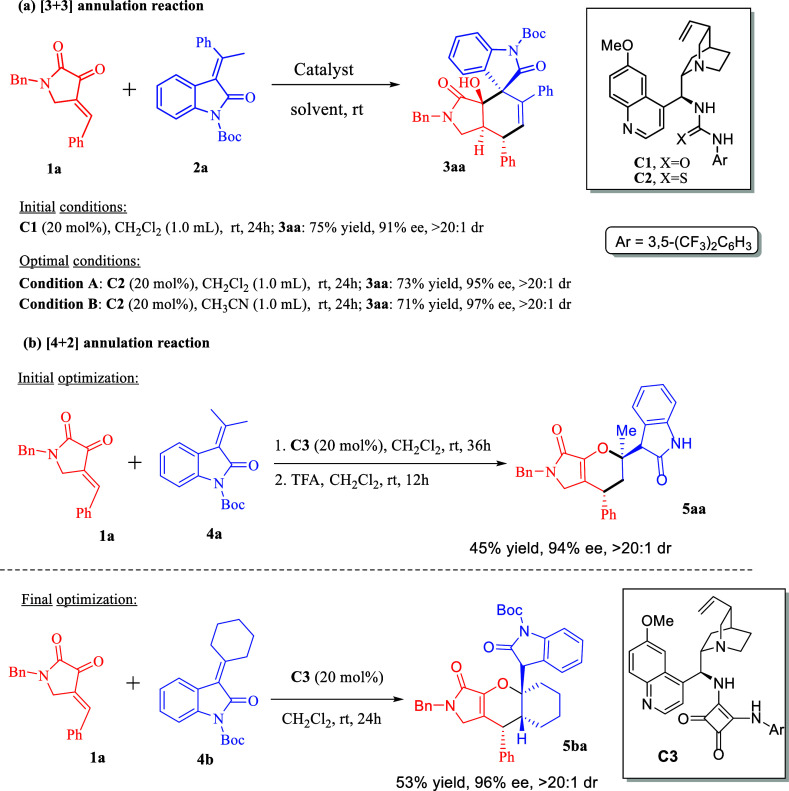
Optimal Conditions for [3 + 3] and [4 + 2] Annulation Reaction

To check the feasibility of our hypothesis, we initiated our investigation by comprising 2,3-dioxopyrrolidine **1a** as the acceptor and 3-alkylidene oxindole **2a** as the vinylogous donor in the presence of 20 mol % quinine-based urea catalyst in CH_2_Cl_2_ at room temperature. The [3 + 3] annulated product **3aa** was obtained in 73% yield, 95% ee with >20:1dr (Scheme 2a). The final optimal conditions were chosen by conducting the reaction at room temperature in CH_2_Cl_2_ (condition A) or CH_3_CN (condition B) with 20 mol % of thiourea **C2** at 0.1 M substrate concentration (Scheme 2a) (for detailed optimization, see the Supporting Information). For the formation of [4 + 2]-type annulated product **5aa**, initially, treatment of **1a** and **4a** with squaramide catalyst **C3** in CH_2_Cl_2_ at room temperature for 36 h provided the corresponding product in 45% yield, 95% ee with >20:1dr after deprotection of Boc group with trifluoroacetic acid (TFA). The removal of the Boc group was necessary due to difficulties in obtaining product purity for data characterization. The final optimal reaction conditions were chosen by conducting the reaction with 3-alkylidene oxindole **4b** as the vinylogous donor and 2,3-dioxopyrrolidine **1a** as the acceptor using squaramide **C3** as the catalyst. The desired [4 + 2] annulation product **5ba** was isolated in 53% yield, 96% ee, and excellent diastereoselectivity (>20:1) without the need for deprotection of Boc group (Scheme 2b) (for detailed optimization, see the Supporting Information).

**Scheme 3:**
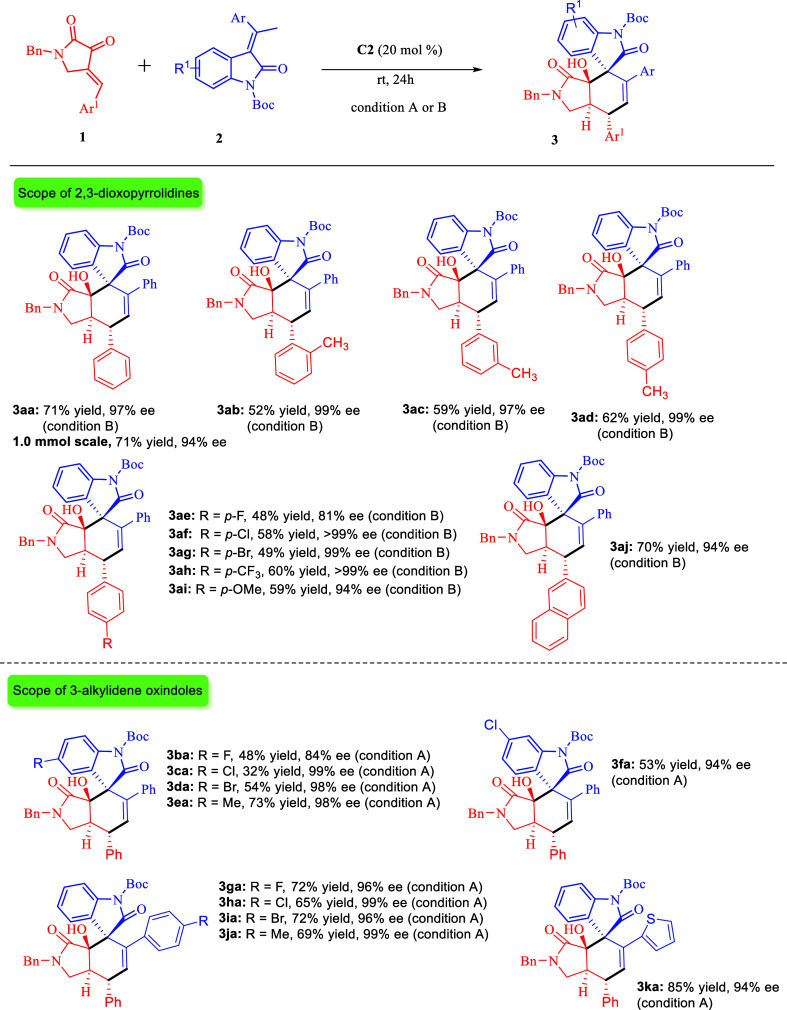
Scope of Asymmetric (3 + 3) Annulation Unless otherwise noted, the reaction was carried out by using 0.1 mmol of **1**, 0.15 mmol of **2**, and 20 mol % of **C2** in 1.0 mL of CH_2_Cl_2_ (condition A) or CH_3_CN (condition B) at rt for 24 h. Isolated yields. ee was determined by HPLC analysis using a chiral column. dr was determined from ^1^H NMR of the crude reaction mixture, and in all cases >20:1 dr were observed.

With the optimal conditions established, we next explored the general substrate scope of [3 + 3] annulation reaction with 2,3-dioxopyrrolidines **1** and 3-alkylidene oxindoles **2**. As shown in Scheme 3, several 2,3-dioxopyrrolidines (**1a**–**j**) were subjected to our optimized reaction conditions affording the corresponding [3 + 3] annulation products (**3aa–aj**) in moderate to good yields, excellent diastereo- and enantioselectivities. The reaction proceeded smoothly with a series of 2,3-dioxopyrrolidines having *ortho*, *meta*, or *para*-substituted phenyl group and desired products (**3ab–ai)** were in moderate to good yields (up to 62%) with excellent stereoselectivities (up to >20:1 dr, up to 99% ee). Some substituted dioxopyrrolidines have lower reaction yields because small amounts of vinylogous Michael adducts (15–20%) were formed and the following intramolecular cyclization did not occur. It is possible the strong electronegativity of halide atoms retarded the intramolecular cyclization.

The diminished reactivity of these substituted dioxopyrrolidines slowed the reaction, resulting in the observation of small amounts of vinylogous Michael adducts (15–20%). We tried to convert the vinylogous Michael adducts to **3**. However, no good results were obtained (see the Supporting Information for details). Furthermore, the naphthyl substituted 2,3-dioxopyrrolidine (**1j**) was also well tolerated and resulted in 70% yield, 94% ee, and >20:1 dr (**3aj**). A scale-up [3 + 3] annulation reaction of **1a** and **2a** could smoothly take place to give product **3aa** in 71% yield with high stereoselectivity, which was similar to the result of a small-scale reaction.

We then turned our attention to the [3 + 3] annulation reaction of 3-alkylidene oxindoles **2** bearing different substituents with 2,3-dioxopyrrolidine **1a**. In all cases, the reactions proceeded well, obtaining **3ba–3ka** with moderate to high yields (up to 85%) and excellent stereoselectivities (up to >20:1 dr, up to 99% ee). The position of substituents on the oxindole ring of 3-alkylidene oxindoles showed a significant influence on the reaction outcome (**3ba–3da** and **3fa**). Moreover, the incorporation of thienyl group into the 3-alkylidene oxindole structure gave the best reaction outcome, resulting in 85% yield, 94% ee, and >20:1 dr (**3ka**).

**Scheme 4:**
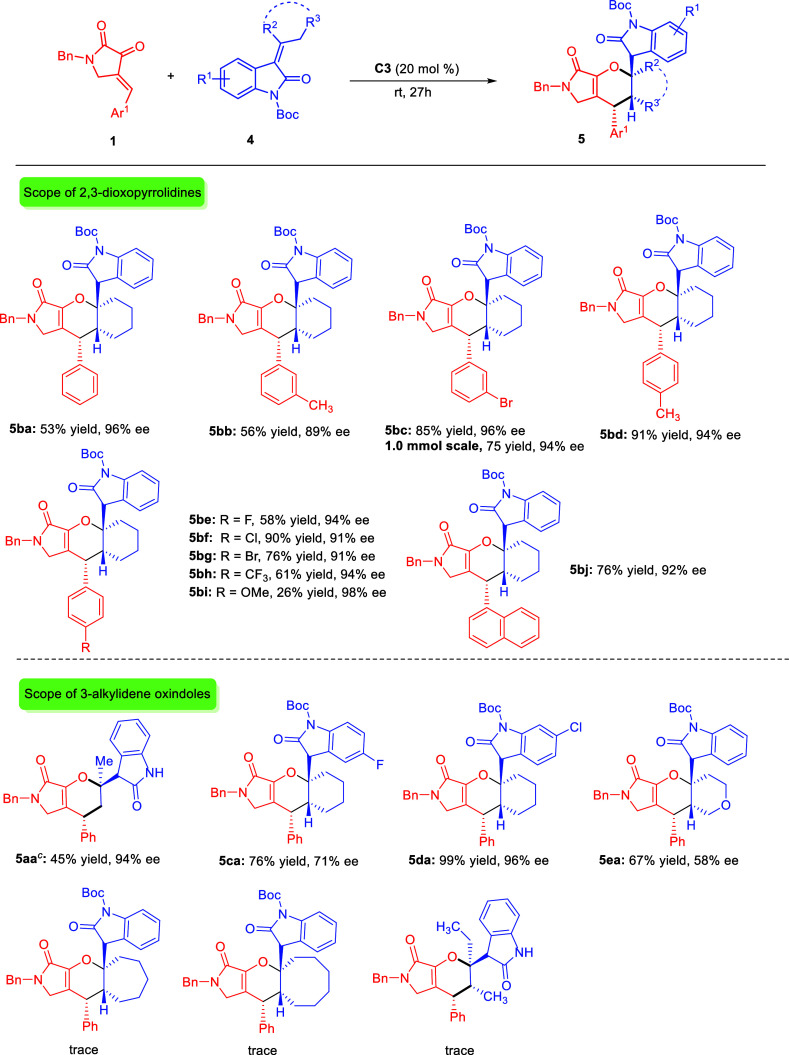
Scope of Asymmetric (4 + 2) Annulation Unless otherwise noted, the reaction was carried out by using 0.1 mmol of **1**, 0.15 mmol of **2**, and 20 mol % of **C3** in 1.0 mL of CH_2_Cl_2_ at rt for 27 h. Isolated yields. **5aa** was obtained after deprotection of Boc group with TFA. ee was determined by HPLC analysis using a chiral column. dr was determined from ^1^H NMR of the crude reaction mixture, and in all cases >20:1 dr were observed.

We next started to investigate the scope of (4 + 2) annulation reaction of differently substituted 2,3-dioxopyrrolidines **1** and 3-alkylidene oxindole **4b**. As shown in Scheme 4, all reactions generally proceeded well to deliver the corresponding products **5ba–5bj** in good to high isolated yields, excellent diastereoselectivities, and enantioselectivities. The position of substitution had a minimal effect on the reactivity, yielding the desired products **5bb–5bh** in 53–91% yields, >20:1 dr, and 87–96% ee. However, it is worth noting that a *p*-OMe-substituted phenyl ring at the alkene end of 2,3-dioxopyrrolidine (**1i**) resulted in a lower yield (**5bi**, 26%) due to the electron-donating effect of the OMe group, which reduced the reactivity of **1i** and led to the recovery of the starting material. Moreover, the 1-naphthyl-substituted 2,3-dioxopyrrolidine was also well tolerated and resulted in 76% yield, >20:1 dr, and 92% ee. A scale-up [4 + 2] annulation reaction of **1c** and **4b** could smoothly take place to give product **5bc** in 80% yield with high stereoselectivities. The reaction yield was similar to the result of a small-scale reaction.

Subsequently, we evaluated the scope of 3-alkylidene oxindoles **4** bearing different aromatic substituents with 2,3-dioxopyrrolidine **1a**. The [4 + 2] annulation product **5aa** is obtained and described in Scheme 2b. The influence of distinct positions of substituted 3-alkylidene oxindoles was first investigated. The 6-position of substitution had little effect on the reactivity and gave the desired products **5da** in 99% yields, >20:1 dr, and 95% ee. Alkylidene oxindoles containing a 5-F substitution (**4c**) or a pyran (**4e**) substitution at the alkene end gave the products **5ca** and **5ea** in good yields with moderate enantioselectivities. No conversion was observed with alkylidene oxindoles containing a cycloheptyl, cyclooctyl, or diethyl substitution.

The stereochemistry of **3ka** (CCDC 2327247) and **5bf** (CCDC 2327249) were determined by X-ray crystallographic analysis (for details, see the Supporting Information).^21^ The absolute configurations of other [3 + 3] and [4 + 2] annulated products were unequivocally assigned by analogy.

Based on the X-ray crystal structure results, previous mechanism studies,^17e^ and experiment results, the plausible reaction mechanism for both [3 + 3] and [4 + 2] annulation reactions were proposed.

**Scheme 5:**
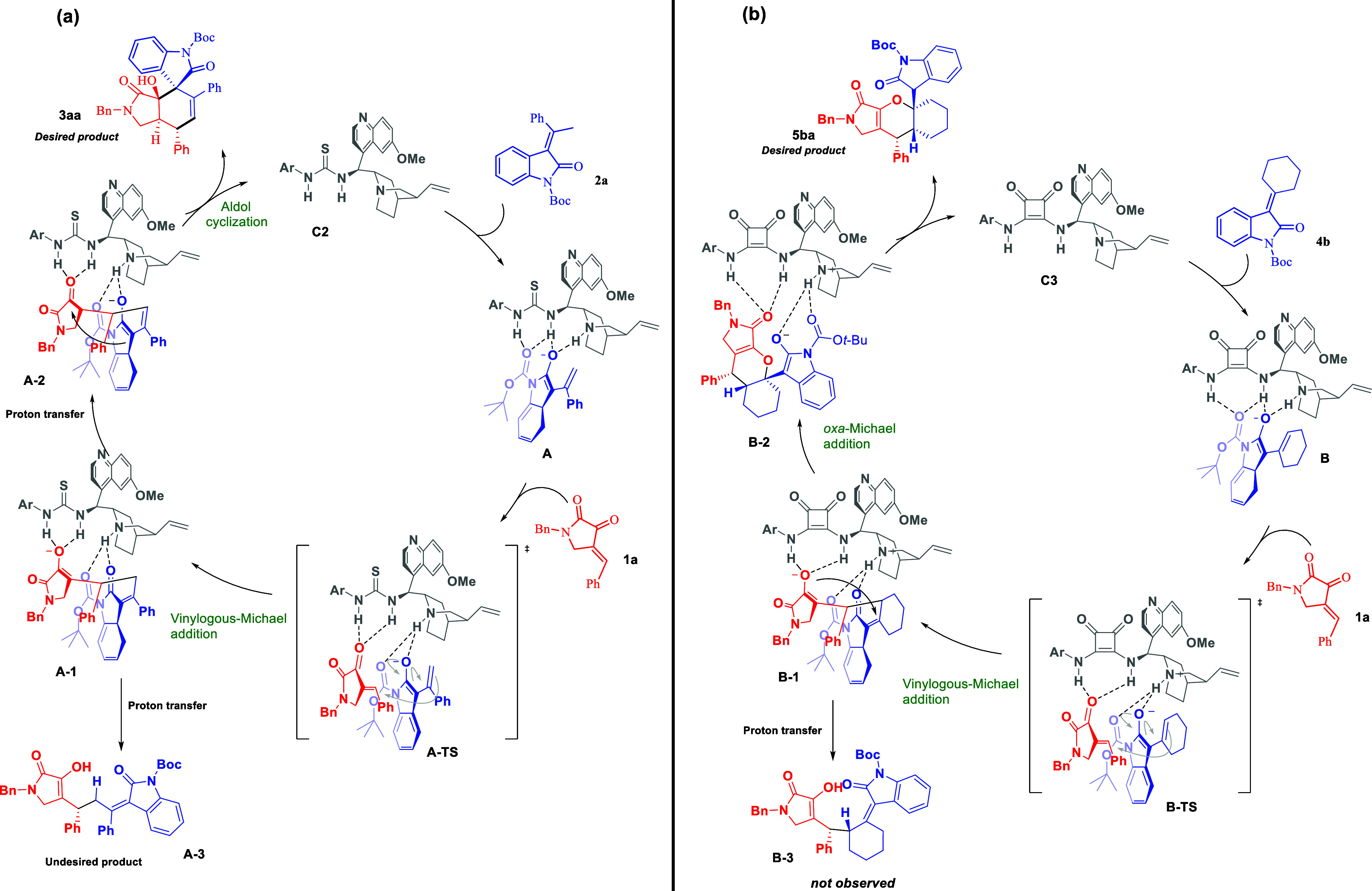
Proposed Mechanism for [3 + 3] and [4 + 2] Annulation Reactions

As shown in Scheme 5a, the quinuclidine base of the catalyst deprotonates the H atom from the γ-position of 3-alkylidene oxindole **2a** and generates vinylogous dienolate species **A**. Meanwhile, the 2,3-dioxopyrrolidine **1a** also develops hydrogen bonding with the thiourea group of the catalyst and finally two substrates are synergistically activated based on the activation pathway proposed by Takemoto.^22^

The direct 1,4-addition of **2a** with 2,3-dioxopyrrolidine **1a** through the *Si* face in **A-TS** generates intermediate **A-1**. This intermediate then undergoes proton transfer and generates vinylogous dienolate species **A-2**. The β-phenyl group might enhance the acidity and facilitate the second vinylogous deprotonation. Hence, the desired [3 + 3] annulation product **3aa** could be obtained after the intramolecular aldol cyclization and protonation and releases the catalyst. The intermediate **A-1** could also undergo another proton-transfer pathway and deliver undesired vinylogous Michael adduct **A-3** (see Table S1 in the Supporting Information).

On the other hand, the [4 + 2] annulation reaction pathway starts with deprotonation of H atom from the γ-position of 3-alkylidene oxindole **4b** by the quinuclidine base of the catalyst **C3** and generates vinylogous dienolate species **B** (Scheme 5b). Similar to the activation pathway for [3 + 3] annulation, 2,3-dioxopyrrolidine **1a** and 3-alkylidene oxindole **4b** are synergistically activated by the catalyst **C3**.

Then, the vinylogous Michael addition of **4b** with **1a** through the *Si* face in **B-TS** generated intermediate enolate **B-1**, which participate in the following intramolecular *oxa*-Michael cyclization. Finally, protonation of **B-2** leads to the formation of a major stereoisomer of **5ba**. In contrast to [3 + 3] annulation reaction, we did not observe the undesired vinylogous Michael adduct **B-3** in the [4 + 2] annulation reaction.

**Figure 2:**
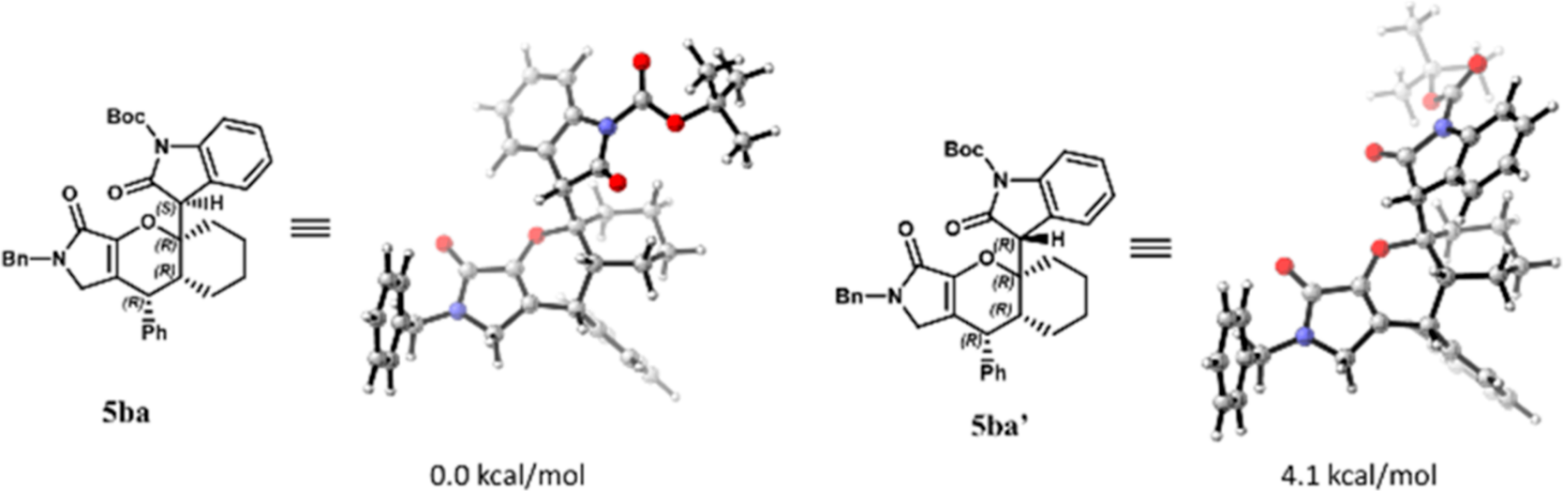
Optimized geometry of diastereomer **5ba** and **5ba′**.

We also discovered that the stereogenic center in the labile C3 position of the oxindole motif of products **5** is well controlled. The DFT calculation showed that **5ba** is 4.1 kcal/mol more stable than diastereomer **5ba′**. It is possible that the oxindole motif in **5ba′** have steric interactions with the axial protons of cyclohexyl ring (Figure 2).

**Scheme 6:**
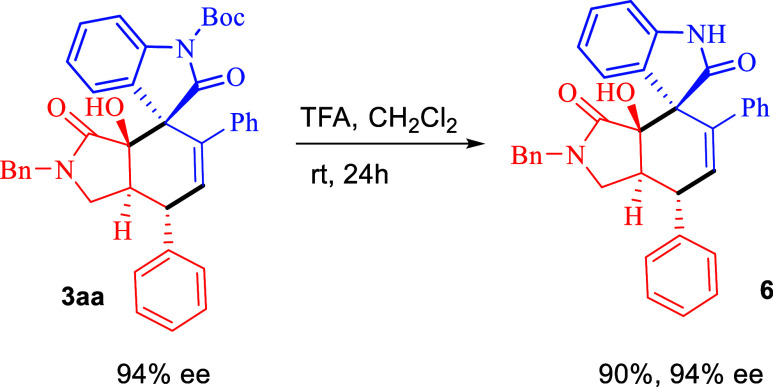
Transformation of **3aa**

To demonstrate the synthetic utility of these methodologies, the product **3aa** could be deprotected under acidic conditions at room temperature for 24 h to give **6** in 90% yield without a loss of enantiopurity (Scheme 6).

In summary, we have developed two protocols for annulation reactions with 2,3-dioxopyrrolidines (**1a**) and 3-alkylidene oxindoles (**2** and **4**), which allowed a rapid and highly efficient assembly of enantioenriched multifunctionalized bicyclic compounds with 5–6 fused rings (**3**) and fused tricyclic pyran derivatives (**5**) through [3 + 3] and [4 + 2] annulation reactions, respectively, in high yields with excellent stereoselectivities. Moreover, the methodology for [3 + 3] annulation reaction is the first example of asymmetric reactions between 3-alkylidene oxindoles and C3-biselectrophiles. Plausible mechanistic pathways for both annulation reactions are proposed on the basis of our results and findings to rationalize the regiodivergence: [3 + 3] annulation reaction involves vinylogous Michael addition followed by intramolecular aldol cyclization; [4 + 2] annulation reaction occurs through a vinylogous Michael addition and a subsequent intramolecular *oxa*-Michael cyclization. These two novel annulation reactions may find applications in drug discovery and natural product synthesis.

## Experimental Section

All commercially available reagents were used without further purification unless otherwise stated. All reaction solvents were purified before use. Proton nuclear magnetic resonance (^1^H NMR) spectra were recorded on a commercial instrument at 400 MHz. Carbon-13 nuclear magnetic resonance (^13^C{^1^H} NMR) spectra were recorded at 100 MHz. The proton signal for residual nondeuterated solvents (δ 7.26 for CHCl_3_) was used as an internal reference for ^1^H NMR spectra. For ^13^C{^1^H} NMR spectra, chemical shifts are reported relative to the δ 77.0 resonance of CHCl_3_. Coupling constants are reported in Hz. Melting points were determined on a BUCHI B-545 melting point apparatus and are uncorrected. High-resolution mass spectra were recorded on a Thermo Fisher Scientific LTQ Orbitrap XL mass spectrometer. The single crystal was measured by a Bruker D8 VENTURE X-ray Single-Crystal Diffractometer. Analytical thin-layer chromatography was performed on silica gel 60 F254 precoated plates with visualization under UV light. Column chromatography was generally performed using 40–63 μm (230–400 mesh) silica gel, typically using a 50–100:1 weight ratio of silica gel to crude product. The ee value determination was carried out using chiral high-performance liquid chromatography (HPLC) with Daicel Chiralpak AD-H or Chiralpak IG and IF columns on JASCO with a UV-2075 detector or a UV-4075 detector. 2,3-Dioxopyrrolidines **1** were prepared according to known procedures.^23^ 3-Alkylidene oxindoles **2** and **4** were prepared according to known procedures.^24^

## General Procedure for the Synthesis of **3**

In a 7 mL glass vial, 2-ethylidene 1,3-indandiones **2** (0.15 mmol), **1** (0.1 mmol), and catalyst **C2** (12.0 mg, 0.02 mmol, 20 mol %) were dissolved in 1.0 mL of CH_2_Cl_2_ or CH_3_CN and stirred for 24 h at room temperature. The solvent was removed in vacuo, and then the reaction mixture was purified by column chromatography (SiO_2_, hexanes: EtOAc, 10:1 to 5:1 to 2:1) to obtain pure products **3**.

## General Procedure for the Synthesis of 1.0 mmol Scale of **3aa**

In a 25 mL glass vial, 2-ethylidene 1,3-indandione **2a** (75 mg, 1.5 mmol), **1a** (83.1 mg, 0.3 mmol), and catalyst **C2** (36.0 mg, 0.06 mmol, 20 mol %) were dissolved in 3 mL of CH_2_Cl_2_ and stirred for 24 h at room temperature. The solvent was removed in vacuo, then the reaction mixture was purified by column chromatography (SiO_2_, hexanes: EtOAc, 10:1 to 5:1 to 2:1) to obtain pure products **3aa** (130 mg, 71% yield).

## General Procedure for the Synthesis of **5**

In a 7 mL glass vial, 2-ethylidene 1,3-indandiones **2** (0.15 mmol), **1** (0.1 mmol), and catalyst **C3** (13.0 mg, 0.02 mmol, 20 mol %) were dissolved in 1.0 mL of CH_2_Cl_2_ and stirred for 27 h at room temperature. The solvent was removed in vacuo, then the reaction mixture was purified by column chromatography (SiO_2_, hexanes: EtOAc, 10:1 to 5:1 to 3:1) to obtain pure products **5**.

## General Procedure for the Synthesis of 1.0 mmol Scale of **5bc**

In a 25 mL glass vial, 2-ethylidene 1,3-indandione **2a** (248 mg, 1.5 mmol), **1a** (355 mg, 1.0 mmol), and catalyst **C3** (130 mg, 0.2 mmol, 20 mol %) were dissolved in 10 mL of CH_2_Cl_2_ and stirred for 27 h at room temperature. The solvent was removed in vacuo, then the reaction mixture was purified by column chromatography (SiO_2_, hexanes: EtOAc, 10:1 to 5:1 to 3:1) to obtain pure products **5bc** (485 mg, 75% yield).

## General Procedure for the Synthesis of **6**

In a 25 mL glass vial, compound **3aa** (139 mg, 1.0 mmol) in 3.0 mL CH_2_Cl_2_ was added TFA (1.0 mL) and stirred for 24 h at room temperature. The solvent was removed in vacuo, then the reaction mixture was purified by column chromatography (SiO_2_, hexanes: EtOAc, 10:1 to 5:1 to 2:1) to obtain pure products **6** (116 mg, 90% yield).

**Caution!** TFA is a highly corrosive acid that is extremely destructive to the upper respiratory tract, eyes, and skin. TFA is also volatile. All TFA handling steps should be conducted wearing appropriate PPE and in a chemical fume hood.

### *tert*-Butyl (3*R*,3a′*R*,7′*S*,7a′*S*)-2′-Benzyl-3a′-hydroxy-2,3′-dioxo-5′,7′-diphenyl-1′,2′,3′,3a′,7′,7a′-hexahydrospiro[indoline-3,4′-isoindole]-1-carboxylate (**3aa**)

White powder (purified by silica gel column chromatography eluting with hexane/EA 10:1 to 5:1 to 2:1); dr: >20:1; yield: 43.6 mg (71%); [α]_D_^20^ + 52.9 (*c* 0.48, CH_2_Cl_2_); mp 102.0–103.0 °C; ^1^H NMR (400 MHz, CDCl_3_): δ 7.92 (d, *J* = 1.1 Hz, 1H), 7.47–7.42 (m, 2H), 7.38–7.33 (m, 2H), 7.29–7.21 (m, 8H), 7.15–7.07 (m, 3H), 6.99–6.97 (m, 2H), 6.81–6.79 (m, 2H), 6.18 (d, *J* = 2.2 Hz, 1H), 4.36 (d, *J* = 15.1 Hz, 1H), 4.14 (d, *J* = 15.1 Hz, 1H), 4.07 (dd, *J* = 11.0, 2.3 Hz, 1H), 3.49 (t, *J* = 9.0 Hz, 1H), 2.98–2.94 (m, 1H), 2.92–2.81 (m, 1H), 1.56 (s, 9H); ^13^C{^1^H} NMR (101 MHz, CDCl_3_): δ 177.1, 171.6, 148.5, 142.0, 141.7, 139.6, 137.5, 136.1, 134.2, 129.8, 129.2, 128.7, 128.3, 128.0, 127.8, 127.7, 127.6, 127.3, 125.1, 124.5, 116.1, 84.8, 79.2, 56.8, 48.0, 46.4, 43.5, 42.1, 28.1; HRMS (ESI) *m*/*z*: calcd for C_39_H_36_N_2_O_5_Na [M + Na]^+^, 635.2516; found, 635.2515; 97% ee was determined by HPLC analysis [chiralpak IG, *n*-hexane/*i*-PrOH 80:20, 0.2 mL/min, λ = 220 nm, *t*_minor_ = 73.84 min, *t*_major_ = 34.39 min].

### *tert*-Butyl (3*R*,3a′*R*,7′*S*,7a′*S*)-2′-Benzyl-3a′-hydroxy-2,3′-dioxo-5′-phenyl-7′-(*o*-tolyl)-1′,2′,3′,3a′,7′,7a′-hexahydrospiro[indoline-3,4′-isoindole]-1-carboxylate (**3ab**)

White powder (purified by silica gel column chromatography eluting with hexane/EA 10:1 to 5:1 to 2:1); dr: >20:1; yield: 32.4 mg (52%); [α]_D_^20^ + 65.2 (*c* 0.50, CH_2_Cl_2_); mp 104.6–106.6 °C; ^1^H NMR (400 MHz, CDCl_3_): δ 7.92 (d, *J* = 7.5 Hz, 1H), 7.54 (d, *J* = 7.4 Hz, 1H), 7.44 (td, *J* = 8.0, 1.3 Hz, 1H), 7.35 (d, *J* = 7.7 Hz, 1H), 7.31–6.86 (m, 14H), 6.86–6.75 (m, 2H), 6.07 (d, 1H), 5.65 (s, 1H), 4.38 (dd, *J* = 10.7, 2.2 Hz, 1H), 4.30 (d, *J* = 15.0 Hz, 1H), 4.21 (d, *J* = 15.1 Hz, 1H), 3.59–3.41 (m, 1H), 3.08–2.91 (m, 2H), 2.40 (s, 3H), 1.58 (s, 9H); ^13^C{^1^H}NMR (101 MHz, CDCl_3_): δ 177.1, 171.5, 148.3, 141.5, 139.9, 139.4, 136.7, 136.1, 136.0, 135.0, 130.8, 129.7, 128.6, 128.1, 127.8, 127.6, 127.6, 127.4, 126.9, 126.8, 124.9, 124.4, 115.9, 84.7, 79.2, 56.5, 47.8, 46.2, 43.6, 28.0, 20.0; HRMS (ESI) *m*/*z*: calcd for C_40_H_38_N_2_O_5_Na [M + Na]^+^, 649.2672; found, 649.2675; 99% ee was determined by HPLC analysis [chiralpak IG, *n*-hexane/*i*-PrOH 80:20, 0.4 mL/min, λ = 220 nm, *t*_major_ = 25.61 min, *t*_minor_ = 33.96 min].

### *tert*-Butyl (3*R*,3a′*R*,7′*S*,7a′*S*)-2′-Benzyl-3a′-hydroxy-2,3′-dioxo-5′-phenyl-7′-(*m*-tolyl)-1′,2′,3′,3a′,7′,7a′-hexahydrospiro[indoline-3,4′-isoindole]-1-carboxylate (**3ac**)

White powder (purified by silica gel column chromatography eluting with hexane/EA 10:1 to 5:1 to 2:1); dr: >20:1; yield: 37.3 mg (59%); [α]_D_^20^ + 51.8 (*c* 0.51, CH_2_Cl_2_); mp 112.4–114.4 °C; ^1^H NMR (400 MHz, CDCl_3_): δ 7.95 (d, *J* = 7.5 Hz, 1H), 7.49–7.42 (m, 2H), 7.28–7.26 (m, 5H), 7.20–7.09 (m, 6H), 7.03–6.99 (m, 2H), 6.84–6.80 (m, 2H), 6.22 (d, *J* = 2.1 Hz, 1H), 5.61 (d, *J* = 2.9 Hz, 1H), 4.40 (d, *J* = 15.1 Hz, 1H), 4.18 (d, *J* = 15.0 Hz, 1H), 4.07 (dd, *J* = 10.9, 2.1 Hz, 1H), 3.54 (t, *J* = 9.1 Hz, 1H), 3.05–2.95 (m, 1H), 2.95–2.81 (m, 1H), 2.37 (s, 3H), 1.58 (s, 9H); ^13^C{^1^H}NMR (101 MHz, CDCl_3_): δ 177.0, 171.5, 148.3, 141.8, 141.5, 139.5, 138.7, 137.1, 136.0, 134.3, 129.7, 128.9, 128.7, 128.6, 128.4, 128.23, 128.17, 127.9, 127.8, 127.7, 127.63, 127.57, 127.52, 127.42, 126.40, 125.0, 124.6, 124.3, 115.9, 84.7, 79.1, 56.6, 47.9, 46.2, 43.2, 41.9, 28.0, 21.5.; HRMS (ESI) *m*/*z*: calcd for C_40_H_38_N_2_O_5_Na [M + Na]^+^, 649.2672; found, 649.2682; 97% ee was determined by HPLC analysis [chiralpak IG, *n*-hexane/*i*-PrOH 80:20, 0.4 mL/min, λ = 220 nm, *t*_major_ = 30.62 min, *t*_minor_ = 73.91 min].

### *tert*-Butyl (3*R*,3a′*R*,7′*S*,7a′*S*)-2′-Benzyl-3a′-hydroxy-2,3′-dioxo-5′-phenyl-7′-(*p*-tolyl)-1′,2′,3′,3a′,7′,7a′-hexahydro-spiro[indoline-3,4′-isoindole]-1-carboxylate (**3ad**)

White powder (purified by silica gel column chromatography eluting with hexane/EA 10:1 to 5:1 to 2:1); dr: >20:1; yield: 38.6 mg (62%); [α]_D_^20^ + 64.6 (*c* 0.50, CH_2_Cl_2_); mp 110.5–112.5 °C; ^1^H NMR (400 MHz, CDCl_3_): δ 8.01 (d, *J* = 8.2 Hz, 1H), 7.55–7.49 (m, 2H), 7.37–7.17 (m, 11H), 7.12–7.05 (m, 2H), 6.91–6.86 (m, 2H), 6.27 (d, *J* = 2.2 Hz, 1H), 5.67 (d, *J* = 1.8 Hz, 1H), 4.44 (d, *J* = 15.1 Hz, 1H), 4.24 (d, *J* = 15.1 Hz, 1H), 4.12 (dd, *J* = 10.9, 2.2 Hz, 1H), 3.58 (t, *J* = 9.1 Hz, 1H), 3.08–3.04 (m, 1H), 2.98–2.91 (m, 1H), 2.42 (s, 3H), 1.66 (s, 9H); ^13^C{^1^H}NMR (101 MHz, CDCl_3_): δ 177.0, 171.5, 148.4, 141.5, 139.5, 138.8, 137.2, 136.8, 136.0, 134.3, 129.71, 129.66, 128.7, 128.6, 128.22, 128.15, 127.63, 127.60, 127.55, 127.49, 127.42, 125.0, 124.3, 115.9, 84.7, 79.1, 56.6, 47.9, 46.2, 43.3, 41.5, 28.0, 21.0; HRMS (ESI) *m*/*z*: calcd for C_40_H_38_N_2_O_5_Na [M + Na]^+^, 649.2672; found, 649.2672; 99% ee was determined by HPLC analysis [chiralpak IG, *n*-hexane/*i*-PrOH 80:20, 0.4 mL/min, λ = 220 nm, *t*_major_ = 29.31 min, *t*_minor_ = 54.25 min].

### *tert*-Butyl (3*R*,3a′*R*,7′*S*,7a′*S*)-2′-Benzyl-7′-(4-fluorophenyl)-3a′-hydroxy-2,3′-dioxo-5′-phenyl-1′,2′,3′,3a′,7′,7a′-hexahydrospiro[indoline-3,4′-isoindole]-1-carboxylate (**3ae**)

White powder (purified by silica gel column chromatography eluting with hexane/EA 10:1 to 5:1 to 2:1); dr: >20:1; yield: 37.2 mg (48%); [α]_D_^20^ + 53.5 (*c* 0.52, CH_2_Cl_2_); mp 102.5–103.5 °C; ^1^H NMR (400 MHz, CDCl_3_): δ 7.96 (d, *J* = 8.2 Hz, 1H), 7.48–7.44 (m, 2H), 7.29–7.00 (m, 13H), 6.83 (d, *J* = 7.0 Hz, 2H), 6.17 (d, *J* = 2.1 Hz, 1H), 5.63 (d, *J* = 1.7 Hz, 1H), 4.40 (d, *J* = 15.0 Hz, 1H), 4.22–4.11 (m, 1H), 4.09 (dd, *J* = 10.9, 2.1 Hz, 1H), 3.51 (t, *J* = 9.1 Hz, 1H), 2.98 (dd, *J* = 8.9, 6.9 Hz, 1H), 2.89–2.84 (m, 1H), 1.60 (s, 9H); ^13^C{^1^H}NMR (101 MHz, CDCl_3_): δ 176.9, 171.4, 161.9 (d, *J* = 245 Hz), 148.3, 141.5, 139.3, 137.6, 137.5 (d, *J* = 4.0 Hz), 135.9, 133.8, 129.8, 129.13, 129.05, 128.7, 128.6, 128.3, 128.2, 127.7, 127.61, 127.55, 127.47, 126.45, 124.6 (d, *J* = 45 Hz), 116.04, 115.99i, 115.8, 84.7, 78.9, 56.5, 47.7, 46.2, 43.4, 41.3, 28.0; ^19^F {^1^H} NMR (376 MHz, CDCl_3_): δ −116.0 (s, 1F); HRMS (ESI) *m*/*z*: calcd for C_39_H_35_N_2_O_5_FNa [M + Na]^+^, 653.2422; found, 653.2416; 81% ee was determined by HPLC analysis [chiralpak IG, *n*-hexane/*i*-PrOH 80:20, 0.4 mL/min, λ = 220 nm, *t*_major_ = 35.96 min, *t*_minor_ = 65.02 min].

### *tert*-Butyl (3*R*,3a′*R*,7′*S*,7a′*S*)-2′-Benzyl-7′-(4-chlorophenyl)-3a′-hydroxy-2,3′-dioxo-5′-phenyl-1′,2′,3′,3a′,7′,7a′-hexahydrospiro[indoline-3,4′-isoindole]-1-carboxylate (**3af**)

White powder (purified by silica gel column chromatography eluting with hexane/EA 10:1 to 5:1 to 2:1); dr: >20:1; yield: 37.5 mg (58%); [α]_D_^20^ + 60.8 (*c* 0.51, CH_2_Cl_2_); mp 111.4–112.4 °C; ^1^H NMR (400 MHz, CDCl_3_): δ 7.98 (d, *J* = 8.2 Hz, 1H), 7.51–7.45 (m, 2H), 7.40–7.14 (m, 11H), 7.05–7.03 (m, 2H), 6.86–6.84 (m, 2H), 6.19 (d, *J* = 2.1 Hz, 1H), 5.66 (d, *J* = 1.6 Hz, 1H), 4.41 (d, *J* = 15.1 Hz, 1H), 4.20 (d, *J* = 15.0 Hz, 1H), 4.09 (dd, *J* = 10.9, 2.2 Hz, 1H), 3.53 (t, *J* = 9.1 Hz, 1H), 2.97 (dd, *J* = 8.9, 6.9 Hz, 1H), 2.90–2.85 (m, 1H), 1.62 (s, 9H); ^13^C{^1^H}NMR (101 MHz, CDCl_3_): δ 176.8, 171.3, 148.3, 141.5, 140.3, 139.2, 137.9, 135.9, 133.4, 133.0, 129.8, 129.2, 129.1, 129.0, 128.6, 128.2, 127.8, 127.62, 127.55, 127.50, 127.4, 124.8, 124.4, 116.0, 84.8, 78.9, 56.5, 47.7, 46.2, 43.3, 41.5, 28.0; HRMS (ESI) *m*/*z*: calcd for C_39_H_35_N_2_O_5_NaCl [M + Na]^+^, 669.2127; found, 669.2133; >99% ee was determined by HPLC analysis [chiralpak IG, *n*-hexane/*i*-PrOH 80:20, 0.4 mL/min, λ = 220 nm, *t*_major_ = 34.47 min, *t*_minor_ = 60.00 min].

### *tert*-Butyl (3*R*,3a′*R*,7′*S*,7a′*S*)-2′-Benzyl-7′-(4-bromophenyl)-3a′-hydroxy-2,3′-dioxo-5′-phenyl-1′,2′,3′,3a′,7′,7a′-hexahydrospiro[indoline-3,4′-isoindole]-1-carboxylate (**3ag**)

White powder (purified by silica gel column chromatography eluting with hexane/EA 10:1 to 5:1 to 2:1); dr: >20:1; yield: 34.0 mg (49%); [α]_D_^20^ + 63.5 (*c* 0.48, CH_2_Cl_2_); mp 131.7–132.7 °C; ^1^H NMR (400 MHz, CDCl_3_): δ 7.93 (d, *J* = 8.2 Hz, 1H), 7.50–7.34 (m, 4H), 7.33–7.06 (m, 9H), 7.00–6.98 (m, 2H), 6.80 (d, *J* = 7.5 Hz, 2H), 6.13 (s, 1H), 5.60 (s, 1H), 4.36 (d, *J* = 15.1 Hz, 1H), 4.16 (d, *J* = 15.0 Hz, 1H), 4.05 (d, *J* = 11.3 Hz, 1H), 3.47 (t, *J* = 9.1 Hz, 1H), 2.96–2.92 (m, 1H), 2.85–2.78 (m, 1H), 1.57 (s, 9H); ^13^C{^1^H}NMR (101 MHz, CDCl_3_): δ 176.8, 171.3, 148.3, 141.5, 140.9, 139.2, 137.9, 135.9, 133.3, 132.2, 129.8, 129.3, 128.8, 128.6, 128.2, 127.8, 127.6, 127.54, 127.50, 127.4, 124.8, 124.4, 121.0, 116.0, 84.8, 78.9, 56.5, 47.6, 46.2, 43.3, 41.5, 28.0; HRMS (ESI) *m*/*z*: calcd for C_39_H_35_N_2_O_5_NaBr [M + Na]^+^, 713.1622; found, 713.1613; 99% ee was determined by HPLC analysis [chiralpak IG, *n*-hexane/*i*-PrOH 80:20, 0.4 mL/min, λ = 220 nm, *t*_major_ = 35.59 min, *t*_minor_ = 51.13 min].

### *tert*-Butyl (3*R*,3a′*R*,7′*S*,7a′*S*)-2′-Benzyl-3a′-hydroxy-2,3′-dioxo-5′-phenyl-7′-(4-(trifluoromethyl)phenyl)-1′,2′,3′,3a′,7′,7a′-hexahydrospiro[indoline-3,4′-isoindole]-1-carboxylate (**3ah**)

White powder (purified by silica gel column chromatography eluting with hexane/EA 10:1 to 5:1 to 2:1); dr: >20:1; yield: 41.1 mg (60%); [α]_D_^20^ + 56.1 (*c* 0.54, CH_2_Cl_2_); mp 115.2–116.2 °C; ^1^H NMR (400 MHz, CDCl_3_): δ 7.96 (d, *J* = 8.2 Hz, 1H), 7.65 (d, *J* = 7.9 Hz, 2H), 7.54–7.40 (m, 4H), 7.29–7.24 (m, 4H), 7.19–7.11 (m, 3H), 7.02–7.00 (m, 2H), 6.86–6.79 (m, 2H), 6.18 (d, *J* = 2.1 Hz, 1H), 5.67 (s, 1H), 4.40 (d, *J* = 15.1 Hz, 1H), 4.20–4.15 (m, 2H), 3.53 (t, *J* = 9.0 Hz, 1H), 2.97 (t, 1H), 2.99–2.84 (m, 2H), 1.60 (s, 8H); ^13^C{^1^H}NMR (101 MHz, CDCl_3_): δ 176.7, 171.2, 148.3, 146.0, 141.5, 139.1, 138.3, 135.8, 132.8, 129.9, 129.7, 129.1, 128.6, 128.5, 128.2, 128.0, 127.8, 127.6, 127.5, 127.3, 126.1 (q, *J* = 4 Hz), 124.7, 124.4, 116.0, 84.8, 78.8, 56.5, 47.6, 46.2, 43.2, 41.9, 28.0; ^19^F {^1^H} NMR (376 MHz, CDCl_3_): δ −63.2 (s, 3F); HRMS (ESI) *m*/*z*: calcd for C_40_H_35_N_2_O_5_F_3_Na [M + Na]^+^, 703.2390; found, 703.2390; >99% ee was determined by HPLC analysis [chiralpak IG, *n*-hexane/*i*-PrOH 80:20, 0.4 mL/min, λ = 220 nm, *t*_major_ = 27.33 min, *t*_minor_ = 33.04 min].

### *tert*-Butyl (3*R*,3a′*R*,7′*S*,7a′*S*)-2′-Benzyl-3a′-hydroxy-7′-(4-methoxyphenyl)-2,3′-dioxo-5′-phenyl-1′,2′,3′,3a′,7′,7a′-hexahydrospiro[indoline-3,4′-isoindole]-1-carboxylate (**3ai**)

White powder (purified by silica gel column chromatography eluting with hexane/EA 10:1 to 5:1 to 2:1); dr: >20:1; yield: 37.4 mg (59[α]_D_^20^%) + 68.6 (*c* 0.50, CH_2_Cl_2_); mp 108.1–109.1 °C; ^1^H NMR (400 MHz, CDCl_3_): δ 7.96 (dd, *J* = 8.2, 1.1 Hz, 1H), 7.53–7.42 (m, 2H), 7.39–7.20 (m, 6H), 7.20–7.09 (m, 3H), 7.07–6.98 (m, 2H), 6.97–6.89 (m, 2H), 6.88–6.79 (m, 2H), 6.20 (d, *J* = 2.1 Hz, 1H), 5.62 (d, *J* = 1.7 Hz, 1H), 4.39 (d, *J* = 15.1 Hz, 1H), 4.19 (d, *J* = 15.0 Hz, 1H), 4.06 (dd, *J* = 10.9, 2.2 Hz, 1H), 3.83 (s, 3H), 3.51 (t, 1H), 3.00 (dd, *J* = 9.0, 6.9 Hz, 1H), 2.94–2.82 (m, 1H), 1.61 (s, 9H); ^13^C{^1^H}NMR (101 MHz, CDCl_3_): δ 177.0, 171.5, 158.7, 148.3, 141.5, 139.4, 137.1, 135.9, 134.5, 133.8, 129.7, 128.8, 128.6, 128.1, 127.8, 127.61, 127.58, 127.53, 127.4, 124.9, 124.3, 115.9, 114.4, 84.7, 79.0, 56.6, 55.3, 47.8, 46.2, 43.4, 41.1, 28.0; HRMS (ESI) *m*/*z*: calcd for C_40_H_38_N_2_O_6_Na [M + Na]^+^, 665.2622; found, 665.2631; 94% ee was determined by HPLC analysis [chiralpak IG, *n*-hexane/*i*-PrOH 80:20, 0.4 mL/min, λ = 220 nm, *t*_major_ = 47.87 min, *t*_minor_ = 71.60 min].

### *tert*-Butyl (3*R*,3a′*R*,7′*S*,7a′*S*)-2′-Benzyl-3a′-hydroxy-7′-(naphthalen-2-yl)-2,3′-dioxo-5′-phenyl-1′,2′,3′,3a′,7′,7a′-hexahydrospiro[indoline-3,4′-isoindole]-1-carboxylate (**3aj**)

White powder (purified by silica gel column chromatography eluting with hexane/EA 10:1 to 5:1 to 2:1); dr: >20:1; yield: 46.2 mg (70[α]_D_^20^%) + 101.3 (*c* 0.48, CH_2_Cl_2_); mp 132.5–133.5 °C; ^1^H NMR (400 MHz, CDCl_3_): δ 8.01 (d, *J* = 7.2 Hz, 1H), 7.96–7.85 (m, 3H), 7.81–7.73 (m, 1H), 7.65–7.41 (m, 5H), 7.35–7.12 (m, 7H), 7.06–6.97 (m, 2H), 6.95–6.86 (m, 2H), 6.35 (d, *J* = 2.1 Hz, 1H), 5.69 (d, *J* = 1.4 Hz, 1H), 4.41 (d, *J* = 15.1 Hz, 1H), 4.31 (dd, *J* = 10.4, 2.2 Hz, 1H), 4.20 (d, *J* = 15.0 Hz, 1H), 3.65–3.59 (m, 1H), 3.05–2.94 (m, 2H), 1.63 (s, 9H); ^13^C{^1^H} NMR (101 MHz, CDCl_3_): δ 176.9, 171.4, 148.3, 141.5, 139.4, 139.2, 137.6, 135.9, 134.0, 133.6, 132.5, 129.7, 128.9, 128.7, 128.6, 128.3, 128.2, 127.71, 127.67, 127.6, 127.5, 127.4, 126.4, 126.3, 126.2, 125.9, 125.8, 124.9, 124.4, 116.0, 84.7, 79.1, 56.6, 47.8, 46.2, 43.4, 42.1, 28.0; HRMS (ESI) *m*/*z*: calcd for C_43_H_38_N_2_O_5_Na [M + Na]^+^, 685.2672; found, 685.2678; 94% ee was determined by HPLC analysis [chiralpak IG, *n*-hexane/i-PrOH 80:20, 0.4 mL/min, λ = 220 nm, *t*_major_ = 42.47 min, *t*_minor_ = 67.87 min].

### *tert*-Butyl (3*R*,3a′*R*,7′*S*,7a′*S*)-2′-Benzyl-5-fluoro-3a′-hydroxy-2,3′-dioxo-5′,7′-diphenyl-1′,2′,3′,3a′,7′,7a′-hexahydrospiro[indoline-3,4′-isoindole]-1-carboxylate (**3ba**)

White powder (purified by silica gel column chromatography eluting with hexane/EA 10:1 to 5:1 to 2:1); dr: >20:1; yield: 30.2 mg (48[α]_D_^20^%) + 36.0 (*c* 0.50, CH_2_Cl_2_); mp 93.9–94.9 °C; ^1^H NMR (400 MHz, CDCl_3_): δ 7.93 (d, *J* = 8.1 Hz, 1H), 7.48–7.20 (m, 11H), 7.01–6.99 (m, 2H), 6.83–6.76 (m, 4H), 6.14 (d, *J* = 2.1 Hz, 1H), 5.59 (s, 1H), 4.38 (d, *J* = 15.1 Hz, 1H), 4.15 (d, *J* = 15.0 Hz, 1H), 4.07 (dd, *J* = 10.9, 2.1 Hz, 1H), 3.50 (t, *J* = 9.1 Hz, 1H), 2.98 (dd, *J* = 9.0, 6.9 Hz, 1H), 2.91–2.84 (m, 1H), 1.59 (s, 9H); ^13^C{^1^H}NMR (101 MHz, CDCl_3_): δ 176.6, 171.3, 159.7 (d, *J* = 242 Hz), 148.3, 141.5, 139.1, 137.5, 137.0, 135.8, 134.6, 129.4, 129.3, 129.2, 129.0, 128.8, 128.7, 128.3, 128.2, 128.1, 127.8, 127.65, 127.61, 127.58, 127.3, 127.1, 126.5, 117.3, 117.2, 116.2 (d, *J* = 23 Hz), 112.5 (d, *J* = 25 Hz), 84.9, 79.0, 56.7, 47.9, 46.3, 43.5, 41.9, 28.0; ^19^F {^1^H} NMR (376 MHz, CDCl_3_): δ −118.1 (s, F); HRMS (ESI) *m*/*z*: calcd for C_39_H_35_N_2_O_5_FNa [M + Na]^+^, 653.2422; found, 653.2413; 84% ee was determined by HPLC analysis [chiralpak IG, *n*-hexane/*i*-PrOH 80:20, 0.4 mL/min, λ = 220 nm, *t*_major_ = 24.75 min, *t*_minor_ = 90.43 min].

### *tert*-Butyl (3*R*,3a′*R*,7′*S*,7a′*S*)-2′-Benzyl-6-chloro-3a′-hydroxy-2,3′-dioxo-5′,7′-diphenyl-1′,2′,3′,3a′,7′,7a′-hexahydrospiro[indoline-3,4′-isoindole]-1-carboxylate (**3ca**)

White powder (purified by silica gel column chromatography eluting with hexane/EA 10:1 to 5:1 to 2:1); dr: >20:1; yield: 20.7 mg (32%); [α]_D_^20^ – 1.509 (*c* 0.53, CH_2_Cl_2_); mp 117.0–118.0 °C; ^1^H NMR (400 MHz, CDCl_3_): δ 8.01 (d, *J* = 2.0 Hz, 1H), 7.39–7.311 (m, 13H), 7.01 (dd, *J* = 7.4, 2.0 Hz, 2H), 6.85–6.81 (m, 2H), 6.20 (d, *J* = 2.1 Hz, 1H), 5.47 (d, *J* = 1.7 Hz, 1H), 4.41 (d, *J* = 15.0 Hz, 1H), 4.13 (d, *J* = 15.0 Hz, 1H), 4.07 (dd, *J* = 11.0, 2.2 Hz, 1H), 3.51 (t, *J* = 9.2 Hz, 1H), 2.98 (dd, *J* = 9.1, 6.9 Hz, 1H), 2.77 (q, *J* = 9.3 Hz, 1H), 1.57 (s, 9H); ^13^C{^1^H} NMR (101 MHz, CDCl_3_): δ 176.4, 171.4, 148.1, 142.3, 141.6, 139.2, 136.9, 135.8, 135.6, 134.4, 129.1, 128.7, 128.3, 127.8, 127.64, 127.58, 127.55, 127.3, 126.1, 125.7, 124.4, 116.7, 85.3, 79.0, 56.4, 47.9, 46.3, 43.5, 41.9, 27.9; HRMS (ESI) *m*/*z*: calcd for C_39_H_35_N_2_O_5_NaCl [M + Na]^+^, 669.2127; found, 669.2137; 94% ee was determined by HPLC analysis [chiralpak IG, *n*-hexane/*i*-PrOH 80:20, 0.4 mL/min, λ = 220 nm, *t*_major_ = 26.47 min, *t*_minor_ = 40.08 min].

### *tert*-Butyl (3*R*,3a′*R*,7′*S*,7a′*S*)-2′-Benzyl-5-bromo-3a′-hydroxy-2,3′-dioxo-5′,7′-diphenyl-1′,2′,3′,3a′,7′,7a′-hexahydrospiro[indoline-3,4′-isoindole]-1-carboxylate (**3da**)

White powder (purified by silica gel column chromatography eluting with hexane/EA 10:1 to 5:1 to 2:1); dr: >20:1; yield: 37.1 mg (54%); [α]_D_^20^ + 49.8 (*c* 0.49, CH_2_Cl_2_); mp 128.6–127.6 °C; ^1^H NMR (400 MHz, CDCl_3_): δ 7.84 (d, *J* = 9.2 Hz, 1H), 7.58–7.55 (m, 2H), 7.44–7.12 (m, 11H), 7.01 (d, *J* = 7.4 Hz, 2H), 6.84–6.82 (m, 2H), 6.22 (d, *J* = 2.1 Hz, 1H), 5.52 (s, 1H), 4.41 (d, *J* = 15.1 Hz, 1H), 4.16 (d, *J* = 15.1 Hz, 1H), 4.08 (dd, *J* = 11.0, 2.1 Hz, 1H), 3.53 (t, *J* = 9.2 Hz, 1H), 3.02 (dd, *J* = 9.1, 6.9 Hz, 1H), 2.80–2.75 (m, 1H), 1.56 (s, 9H); ^13^C{^1^H} NMR (101 MHz, CDCl_3_): δ 176.1, 171.3, 148.1, 141.5, 140.6, 139.1, 136.8, 135.8, 134.7, 132.6, 129.9, 129.2, 128.8, 128.3, 127.9, 127.61, 127.57, 127.51, 127.3, 117.5, 117.3, 85.1, 79.1, 56.5, 47.9, 46.2, 43.6, 41.9, 28.0; HRMS (ESI) *m*/*z*: calcd for C_39_H_35_N_2_O_5_NaBr [M + Na]^+^, 713.1621; found, 713.1623; 98% ee was determined by HPLC analysis [chiralpak IG, *n*-hexane/*i*-PrOH 80:20, 0.4 mL/min, λ = 220 nm, *t*_major_ = 24.91 min, *t*_minor_ = 77.94 min].

### *tert*-Butyl (3*R*,3a′*R*,7′*S*,7a′*S*)-2′-Benzyl-3a′-hydroxy-5-methyl-2,3′-dioxo-5′,7′-diphenyl-1′,2′,3′,3a′,7′,7a′-hexahydrospiro[indoline-3,4′-isoindole]-1-carboxylate (**3ea**)

White powder (purified by silica gel column chromatography eluting with hexane/EA 10:1 to 5:1 to 2:1); dr: >20:1; yield: 45.7 mg (73%); [α]_D_^20^ + 58.7 (*c* 0.53, CH_2_Cl_2_); mp 123.5–124.5 °C; ^1^H NMR (400 MHz, CDCl_3_): δ 7.84 (d, *J* = 8.7 Hz, 1H), 7.42–7.39 (m, 2H), 7.34–7.11 (m, 11H), 7.04–7.01 (m, 2H), 6.84 (d, *J* = 7.4 Hz, 2H), 6.23 (d, *J* = 2.1 Hz, 1H), 5.65 (s, 1H), 4.43 (d, *J* = 15.1 Hz, 1H), 4.17 (d, *J* = 15.1 Hz, 1H), 4.11 (dd, *J* = 10.9, 2.2 Hz, 1H), 3.54 (t, *J* = 9.1 Hz, 1H), 3.02 (dd, *J* = 8.9, 6.9 Hz, 1H), 2.92–2.87 (m, 1H), 2.43 (s, 3H), 1.59 (s, 9H); ^13^C{^1^H} NMR (101 MHz, CDCl_3_): δ 177.1, 171.5, 148.4, 142.0, 139.5, 139.2, 137.4, 136.0, 133.9, 133.9, 130.2, 129.1, 128.6, 128.2, 127.9, 127.7, 127.6, 127.5, 127.4, 127.1, 126.3, 125.5, 115.7, 84.5, 79.0, 56.6, 47.8, 46.2, 43.3, 42.0, 28.0, 21.4; HRMS (ESI) *m*/*z*: calcd for C_40_H_38_N_2_O_5_Na [M + Na]^+^, 649.2672; found, 649.2673; 98% ee was determined by HPLC analysis [chiralpak IG, *n*-hexane/*i*-PrOH 80:20, 0.4 mL/min, λ = 220 nm, *t*_major_ = 35.78 min, *t*_minor_ = 104.70 min].

### *tert*-Butyl (3*R*,3a′*R*,7′*S*,7a′*S*)-2′-Benzyl-5-chloro-3a′-hydroxy-2,3′-dioxo-5′,7′-diphenyl-1′,2′,3′,3a′,7′,7a′-hexahydrospiro[indoline-3,4′-isoindole]-1-carboxylate (**3fa**)

White powder (purified by silica gel column chromatography eluting with hexane/EA 10:1 to 5:1 to 2:1); dr: >20:1; yield: 34.0 mg (53%); [α]_D_^20^ + 55.7 (*c* 0.47, CH_2_Cl_2_); mp 125.0–126.0 °C; ^1^H NMR (400 MHz, CDCl_3_): δ 7.90–7.88 (m, 1H), 7.42–7.38 (m, 3H), 7.31–7.24 (m, 7H), 7.19–7.11 (m, 3H), 7.02–7.00 (m, 2H), 6.85–6.81 (m, 2H), 6.20 (d, *J* = 2.1 Hz, 1H), 5.47 (d, *J* = 1.7 Hz, 1H), 4.41 (d, *J* = 15.0 Hz, 1H), 4.13 (d, *J* = 15.0 Hz, 1H), 4.07 (dd, *J* = 11.0, 2.2 Hz, 1H), 3.51 (t, *J* = 9.2 Hz, 1H), 2.98 (dd, *J* = 9.1, 6.9 Hz, 1H), 2.78–2.75 (m, 1H), 1.57 (s, 9H); ^13^C{^1^H} NMR (101 MHz, CDCl_3_): δ 176.2, 171.3, 148.2, 141.5, 140.0, 139.1, 136.9, 135.8, 135.6, 134.7, 129.9, 129.7, 129.6, 129.2, 128.7, 128.3, 127.8, 127.6, 127.6, 127.5, 127.3, 125.0, 117.1, 85.1, 79.0, 56.6, 47.9, 46.3, 43.6, 41.9, 28.0; HRMS (ESI) *m*/*z*: calcd for C_39_H_35_N_2_O_5_NaCl [M + Na]^+^, 669.2126; found, 669.2137; 94% ee was determined by HPLC analysis [chiralpak IG, *n*-hexane/*i*-PrOH 80:20, 0.4 mL/min, λ = 220 nm, *t*_major_ = 26.47 min, *t*_minor_ = 40.06 min].

### *tert*-Butyl (3*R*,3a′*R*,7′*S*,7a′*S*)-2′-Benzyl-5′-(4-fluorophenyl)-3a′-hydroxy-2,3′-dioxo-7′-phenyl-1′,2′,3′,3a′,7′,7a′-hexahydrospiro[indoline-3,4′-isoindole]-1-carboxylate (**3ga**)

White powder (purified by silica gel column chromatography eluting with hexane/EA 10:1 to 5:1 to 2:1); dr: >20:1; yield: 45.1 mg (72%); [α]_D_^20^ + 57.8 (*c* 0.50, CH_2_Cl_2_); mp 122.5–123.5 °C; ^1^H NMR (400 MHz, CDCl_3_): δ 7.93 (d, *J* = 8.1 Hz, 1H), 7.48–7.21 (m, 11H), 7.01–6.98 (m, 2H), 6.83–6.76 (m, 4H), 6.14 (d, *J* = 2.1 Hz, 1H), 5.59 (d, *J* = 1.7 Hz, 1H), 4.38 (d, *J* = 15.1 Hz, 1H), 4.15 (d, *J* = 15.0 Hz, 1H), 4.07 (dd, *J* = 10.8, 2.1 Hz, 1H), 3.50 (t, *J* = 9.1 Hz, 1H), 2.98 (dd, *J* = 9.0, 6.9 Hz, 1H), 3.00–2.87 (m, 1H), 1.59 (s, 9H); ^13^C{^1^H} NMR (101 MHz, CDCl_3_): δ 176.9, 171.4, 162.3 (d, *J* = 246 Hz), 148.2, 141.7, 141.5, 136.4, 135.9, 135.4, 134.5, 134.4, 129.8, 129.4 (d, *J* = 8 Hz), 129.1, 128.6, 127.6, 127.5, 127.3, 127.2, 124.9, 124.4, 115.9, 115.0 (d, *J* = 25.7 Hz), 84.9, 79.0, 56.7, 47.8, 46.2, 43.3, 41.9, 28.0; ^19^F {^1^H} NMR (376 MHz, CDCl_3_): δ −114.4 (s, 1F); HRMS (ESI) *m*/*z*: calcd for C_39_H_35_N_2_O_5_FNa [M + Na]^+^, 653.2422; found, 653.2420; 96% ee was determined by HPLC analysis [chiralpak IG, *n*-hexane/*i*-PrOH 80:20, 0.4 mL/min, λ = 220 nm, *t*_major_ = 34.46 min, *t*_minor_ = 88.07 min].

### *tert*-Butyl (3*R*,3a′*R*,7′*S*,7a′*S*)-2′-Benzyl-5′-(4-chlorophenyl)-3a′-hydroxy-2,3′-dioxo-7′-phenyl-1′,2′,3′,3a′,7′,7a′-hexahydrospiro[indoline-3,4′-isoindole]-1-carboxylate (**3ha**)

White powder (purified by silica gel column chromatography eluting with hexane/EA 10:1 to 5:1 to 2:1); dr: >20:1; yield: 41.9 mg (65%); [α]_D_^20^ + 54.6 (*c* 0.50, CH_2_Cl_2_); mp 106.6–107.6 °C; ^1^H NMR (400 MHz, CDCl_3_): δ 7.92 (d, *J* = 7.9 Hz, 1H), 7.46–7.42 (m, 1H), 7.39–7.34 (m, 2H), 7.32–7.27 (m, 4H), 7.23–7.21 (m, 4H), 7.08 (d, *J* = 8.0 Hz, 2H), 7.01–6.94 (m, 2H), 6.74 (d, *J* = 8.5 Hz, 2H), 6.16 (d, *J* = 2.1 Hz, 1H), 5.54 (d, *J* = 1.7 Hz, 1H), 4.51 (d, *J* = 6.2 Hz, 1H), 4.37 (d, *J* = 15.1 Hz, 1H), 4.14 (d, *J* = 15.1 Hz, 1H), 4.06 (dd, *J* = 10.9, 2.2 Hz, 1H), 3.49 (t, *J* = 9.1 Hz, 1H), 2.97 (dd, *J* = 8.9, 6.9 Hz, 1H), 2.90–2.83 (m, 1H), 1.57 (s, 9H); ^13^C{^1^H} NMR (101 MHz, CDCl_3_): δ 176.8, 171.3, 148.2, 141.6, 141.5, 137.8, 136.3, 135.9, 134.7, 133.6, 129.9, 129.1, 129.0, 128.61, 128.55, 128.3, 127.8, 127.6, 127.5, 127.3, 127.2, 124.9, 124.5, 116.0, 84.9, 79.0, 56.6, 47.8, 46.2, 43.2, 42.0, 28.0; HRMS (ESI) *m*/*z*: calcd for C_39_H_35_N_2_O_5_NaCl [M + Na]^+^, 669.2126; found, 669.2120; 99% ee was determined by HPLC analysis [chiralpak IG, *n*-hexane/*i*-PrOH 80:20, 0.4 mL/min, λ = 220 nm, *t*_major_ = 38.00 min, *t*_minor_ = 94.81 min].

### *tert*-Butyl (3*R*,3a′*R*,7′*S*,7a′*S*)-2′-Benzyl-5′-(4-bromophenyl)-3a′-hydroxy-2,3′-dioxo-7′-phenyl-1′,2′,3′,3a′,7′,7a′-hexahydrospiro[indoline-3,4′-isoindole]-1-carboxylate (**3ia**)

White powder (purified by silica gel column chromatography eluting with hexane/EA 10:1 to 5:1 to 2:1); dr: >20:1; yield: 49.9 mg (72%); [α]_D_^20^ + 55.6 (*c* 0.50, CH_2_Cl_2_); mp 129.6–130.6 °C; ^1^H NMR (400 MHz, CDCl_3_): δ 7.96 (d, *J* = 7.9 Hz, 1H), 7.49–7.12 (m, 13H), 7.03–7.01 (m, 2H), 6.71 (d, *J* = 8.5 Hz, 2H), 6.20 (d, *J* = 2.1 Hz, 1H), 5.57 (d, *J* = 1.7 Hz, 1H), 4.41 (d, *J* = 15.1 Hz, 1H), 4.17 (d, *J* = 15.1 Hz, 1H), 4.09 (dd, *J* = 10.9, 2.1 Hz, 1H), 3.53 (t, *J* = 9.1 Hz, 1H), 3.00 (dd, *J* = 8.9, 6.9 Hz, 1H), 2.94–2.87 (m, 1H), 1.63 (s, 9H); ^13^C{^1^H} NMR (101 MHz, CDCl_3_): δ 176.8, 171.3, 148.2, 141.5, 141.4, 138.3, 136.3, 135.9, 134.8, 131.3, 129.9, 129.3, 129.1, 129.0, 128.6, 127.6, 127.5, 127.3, 127.2, 124.9, 124.5, 121.9, 116.0, 85.0, 78.9, 60.4, 56.5, 47.8, 46.2, 43.2, 42.0, 28.0; HRMS (ESI) *m*/*z*: calcd for C_39_H_35_N_2_O_5_NaBr [M + Na]^+^, 713.1621; found, 713.1629; 96% ee was determined by HPLC analysis [chiralpak IG, *n*-hexane/*i*-PrOH 80:20, 0.4 mL/min, λ = 220 nm, *t*_major_ = 42.75 min, *t*_minor_ = 119.76 min].

### *tert*-Butyl (3*R*,3a′*R*,7′*S*,7a′*S*)-2′-Benzyl-3a′-hydroxy-2,3′-dioxo-7′-phenyl-5′-(*p*-tolyl)-1′,2′,3′,3a′,7′,7a′-hexahydrospiro[indoline-3,4′-isoindole]-1-carboxylate (**3ja**)

White powder (purified by silica gel column chromatography eluting with hexane/EA 10:1 to 5:1 to 2:1); dr: >20:1; yield: 43.1 mg (69%); [α]_D_^20^ + 59.0 (*c* 0.51, CH_2_Cl_2_); mp 124.6–125.6 °C; ^1^H NMR (400 MHz, CDCl_3_): δ 7.89 (d, *J* = 8.0 Hz, 1H), 7.42–7.16 (m, 11H), 6.96–6.93 (m, 1H), 6.87 (d, *J* = 7.9 Hz, 2H), 6.67 (d, *J* = 8.0 Hz, 2H), 6.13 (d, *J* = 2.1 Hz, 1H), 5.55 (s, 1H), 4.33 (d, *J* = 15.1 Hz, 1H), 4.13–4.01 (m, 2H), 3.46 (t, *J* = 9.1 Hz, 1H), 2.92 (dd, *J* = 8.9, 6.9 Hz, 1H), 2.94–2.81 (m, 1H), 2.19 (s, 3H), 1.55 (s, 9H); ^13^C{^1^H} NMR (101 MHz, CDCl_3_): δ 177.1, 171.5, 148.4, 142.0, 141.5, 137.3, 137.2, 136.5, 136.0, 133.6, 129.6, 129.0, 128.9, 128.6, 127.70, 127.66, 127.59, 127.43, 127.37, 127.1, 125.0, 124.3, 115.9, 84.7, 79.0, 56.7, 47.9, 46.2, 43.3, 42.0, 28.0, 21.0; HRMS (ESI) *m*/*z*: calcd for C_40_H_38_N_2_O_5_Na [M + Na]^+^, 649.2672; found, 649.2678; 99% ee was determined by HPLC analysis [chiralpak AD-H, *n*-hexane/*i*-PrOH 80:20, 0.2 mL/min, λ = 220 nm, *t*_major_ = 38.36 min, *t*_minor_ = 41.46 min].

### *tert*-Butyl (3*R*,3a′*R*,7′*S*,7a′*S*)-2′-Benzyl-3a′-hydroxy-2,3′-dioxo-7′-phenyl-5′-(thiophen-2-yl)-1′,2′,3′,3a′,7′,7a′-hexahydrospiro[indoline-3,4′-isoindole]-1-carboxylate (**3ka**)

White powder (purified by silica gel column chromatography eluting with hexane/EA 10:1 to 5:1 to 2:1); dr: >20:1; yield: 52.3 mg (85%); [α]_D_^20^ + 28.0 (*c* 0.49, CH_2_Cl_2_); mp 123.3–124.3 °C; ^1^H NMR (400 MHz, CDCl_3_): δ 8.04 (d, *J* = 8.1 Hz, 1H), 7.52–7.39 (m, 4H), 7.35–7.32 (m, 3H), 7.29–7.24 (m, 4H), 7.10–6.94 (m, 3H), 6.81 (dd, *J* = 5.1, 3.6 Hz, 1H), 6.52 (d, 2H), 6.49 (d, *J* = 2.2 Hz, 1H), 5.46 (d, *J* = 1.7 Hz, 1H), 4.40 (d, *J* = 15.1 Hz, 1H), 4.17 (d, *J* = 15.1 Hz, 1H), 4.11 (dd, *J* = 10.9, 2.2 Hz, 1H), 3.50 (t, *J* = 9.1 Hz, 1H), 2.96 (dd, *J* = 8.9, 6.9 Hz, 1H), 2.92–2.70 (m, 1H), 1.68 (s, 9H); ^13^C{^1^H} NMR (101 MHz, CDCl_3_): δ 176.6, 171.2, 148.5, 141.9, 141.6, 141.4, 135.9, 134.6, 130.9, 130.0, 129.1, 128.6, 127.7, 127.6, 127.5, 127.3, 127.2, 127.0, 125.0, 124.7, 124.4, 124.1, 116.1, 84.9, 78.9, 56.7, 47.7, 46.2, 43.2, 42.0, 28.1; HRMS (ESI) *m*/*z*: calcd for C_37_H_34_N_2_O_5_NaS [M + Na]^+^, 641.2080; found, 641.2076; 94% ee was determined by HPLC analysis [chiralpak IG, *n*-hexane/*i*-PrOH 80:20, 0.4 mL/min, λ = 220 nm, *t*_major_ = 38.18 min, *t*_minor_ = 91.08 min].

### *tert*-Butyl (*R*)-3-((4a*R*,8a*R*,9*R*)-2-Benzyl-3-oxo-9-phenyl-2,3,5,6,7,8,8a,9-octahydrochromeno[2,3-*c*]pyrrol-4a(1*H*)-yl)-2-oxoindoline-1-carboxylate (**5ba**)

White powder (purified by silica gel column chromatography eluting with hexane/EA 10:1 to 5:1 to 3:1); dr: >20:1; yield: 31.4 mg (53%); [α]_D_^20^ + 31.7 (*c* 0.48, CH_2_Cl_2_); mp 151.1–152.1 °C; ^1^H NMR (400 MHz, CDCl_3_): δ 7.73–7.70 (m, 2H), 7.32–7.18 (m, 12H), 4.73 (d, *J* = 14.9 Hz, 1H), 4.60 (d, *J* = 14.9 Hz, 1H), 4.49 (d, *J* = 6.0 Hz, 1H), 4.25 (s, 1H), 3.90 (d, *J* = 18.9 Hz, 1H), 3.55 (dd, *J* = 18.5, 1.7 Hz, 1H), 2.89–2.84 (m, 1H), 1.66 (s, 9H), 1.64–1.60 (m, 2H), 1.39–1.01 (m, 5H); ^13^C{^1^H} NMR (101 MHz, CDCl_3_): δ 173.0, 165.5, 148.9, 144.4, 140.3, 138.6, 137.0, 129.6, 128.9, 128.50, 128.46, 128.34, 128.28, 128.0, 127.8, 127.2, 127.1, 125.3, 124.5, 123.8, 114.4, 84.9, 84.2, 48.9, 47.9, 46.8, 41.3, 38.9, 30.8, 28.1, 24.9, 23.2, 20.8; HRMS (FAB) *m*/*z*: calcd for C_37_H_38_N_2_O_5_ [M + H]^+^, 591.2859; found, 591.2857; 96% ee was determined by HPLC analysis [chiralpak AD-H, *n*-hexane/*i*-PrOH 80:20, 0.2 mL/min, λ = 220 nm, *t*_major_ = 62.53 min, *t*_minor_ = 30.57 min].

### *tert*-Butyl (*R*)-3-((4a*R*,8a*R*,9*R*)-2-Benzyl-3-oxo-9-(*m*-tolyl)-2,3,5,6,7,8,8a,9-octahydrochromeno[2,3-*c*]pyrrol-4a(1*H*)-yl)-2-oxoindoline-1-carboxylate (**5bb**)

White powder (purified by silica gel column chromatography eluting with hexane/EA 10:1 to 5:1 to 3:1); dr: >20:1; yield: 33.8 mg (56%); [α]_D_^20^ + 34.0 (*c* 0.48, CH_2_Cl_2_); mp 174.5–175.5 °C; ^1^H NMR (400 MHz, CDCl_3_): δ 7.73–7.69 (m, 2H), 7.32–7.16 (m, 8H), 7.05–7.03 (m, 2H), 6.94 (d, *J* = 7.7 Hz, 1H), 4.72 (d, *J* = 14.8 Hz, 1H), 4.61 (d, *J* = 14.9 Hz, 1H), 4.44 (d, *J* = 5.9 Hz, 1H), 4.24 (s, 1H), 3.89 (d, *J* = 17.5 Hz, 1H), 3.54 (d, *J* = 17.5 Hz, 1H), 2.86 (ddd, *J* = 12.7, 6.0, 4.0 Hz, 1H), 2.31 (s, 3H), 1.66 (s, 9H), 1.63–1.60 (m, 2H), 1.43–0.94 (m, 5H); ^13^C {^1^H}NMR (101 MHz, CDCl_3_): δ 173.1, 165.5, 148.9, 144.3, 140.3, 138.5, 138.3, 137.0, 130.5, 128.9, 128.5, 128.3, 128.2, 127.8, 127.2, 126.5, 125.3, 124.5, 124.0, 114.4, 85.0, 84.2, 48.9, 47.9, 46.8, 41.2, 38.9, 30.8, 28.2, 24.9, 23.2, 21.5, 20.8; HRMS (FAB) *m*/*z*: calcd for C_38_H_40_N_2_O_5_ [M + H]^+^, 605.3015; found, 605.3009; 89% ee was determined by HPLC analysis [chiralpak AD-H, *n*-hexane/*i*-PrOH 50:50, 0.2 mL/min, λ = 220 nm, *t*_major_ = 20.32 min, *t*_minor_ = 17.97 min].

### *tert*-Butyl (*R*)-3-((4a*R*,8a*R*,9*R*)-2-Benzyl-9-(3-bromophenyl)-3-oxo-2,3,5,6,7,8,8a,9-octahydrochromeno[2,3-*c*]pyrrol-4a(1*H*)-yl)-2-oxoindoline-1-carboxylate (**5bc**)

White powder (purified by silica gel column chromatography eluting with hexane/EA 10:1 to 5:1 to 3:1); dr: >20:1; yield: 56.9 mg (85%); [α]_D_^20^ + 30.9 (*c* 0.43, CH_2_Cl_2_); mp 148.2–149.2 °C; ^1^H NMR (400 MHz, CDCl_3_): δ 7.72–7.69 (m, 2H), 7.39–7.22 (m, 9H), 7.17 (t, *J* = 7.6 Hz, 1H), 7.15–7.09 (m, 1H), 4.75 (d, *J* = 15.0 Hz, 1H), 4.59 (d, *J* = 14.8 Hz, 1H), 4.45 (d, *J* = 5.8 Hz, 1H), 4.20 (s, 1H), 3.86 (d, *J* = 18.5 Hz, 1H), 3.55 (dd, *J* = 18.6, 1.7 Hz, 1H), 2.89–2.85 (m, 1H), 1.66 (s, 9H), 1.64–1.59 (m, 2H), 1.39–1.03 (m, 5H); ^13^C{^1^H} NMR (101 MHz, CDCl_3_): δ 173.0, 165.3, 148.9, 144.7, 141.1, 140.3, 136.8, 132.5, 130.3, 130.0, 128.9, 128.6, 128.2, 128.1, 127.9, 127.1, 125.3, 124.3, 122.75, 122.69, 114.5, 85.1, 84.2, 48.9, 47.7, 46.8, 41.1, 38.8, 30.8, 28.2, 24.8, 23.2, 20.7; HRMS (FAB) *m*/*z*: calcd for C_37_H_37_BrN_2_O_5_ [M + H]^+^, 669.1964; found, 669.1968; 96% ee was determined by HPLC analysis [chiralpak AD-H, *n*-hexane/*i*-PrOH 50:50, 0.2 mL/min, λ = 254 nm, *t*_major_ = 22.60 min, *t*_minor_ = 19.03 min].

### *tert*-Butyl (*R*)-3-((4a*R*,8a*R*,9*R*)-2-Benzyl-3-oxo-9-(*p*-tolyl)-2,3,5,6,7,8,8a,9-octahydrochromeno[2,3-*c*]pyrrol-4a(1*H*)-yl)-2-oxoindoline-1-carboxylate (**5bd**)

White powder (purified by silica gel column chromatography eluting with hexane/EA 10:1 to 5:1 to 3:1); dr: >20:1; yield: 45.5 mg (91%); [α]_D_^20^ + 28.5 (*c* 0.47, CH_2_Cl_2_); mp 155.1–156.1 °C; ^1^H NMR (400 MHz, CDCl_3_): δ 7.74–7.71 (m, 2H), 7.35–7.22 (m, 7H), 7.12 (d, *J* = 8.1 Hz, 2H), 7.09 (d, *J* = 8.2 Hz, 2H), 4.73 (d, *J* = 14.9 Hz, 1H), 4.61 (d, *J* = 14.9 Hz, 1H), 4.46 (d, *J* = 5.8 Hz, 1H), 4.25 (s, 1H), 3.90 (d, *J* = 18.5 Hz, 1H), 3.55 (dd, *J* = 18.5, 1.8 Hz, 1H), 2.86 (ddd, *J* = 12.7, 5.9, 4.0 Hz, 1H), 2.33 (s, 3H), 1.67 (s, 9H), 1.65–1.57 (m, 2H), 1.41–1.01 (m, 5H); ^13^C{^1^H} NMR (101 MHz, CDCl_3_): δ 172.9, 165.4, 148.8, 144.1, 140.1, 136.8, 136.6, 135.3, 129.4, 129.0, 128.7, 128.3, 128.2, 127.6, 127.0, 125.1, 124.4, 124.0, 114.2, 84.8, 84.0, 48.8, 47.8, 46.7, 40.8, 38.8, 30.7, 28.1, 24.8, 23.0, 21.0, 20.6; HRMS (FAB) *m*/*z*: calcd for C_38_H_40_N_2_O_5_ [M + H]^+^, 605.3015; found, 605.3017; 94% ee was determined by HPLC analysis [chiralpak AD-H, *n*-hexane/*i*-PrOH 50:50, 0.2 mL/min, λ = 220 nm, *t*_major_ = 21.31 min, *t*_minor_ = 17.93 min].

### *tert*-Butyl (*R*)-3-((4a*R*,8a*R*,9*R*)-2-Benzyl-9-(4-fluorophenyl)-3-oxo-2,3,5,6,7,8,8a,9-octahydrochromeno[2,3-*c*]pyrrol-4a(1*H*)-yl)-2-oxoindoline-1-carboxylate (**5be**)

White powder (purified by silica gel column chromatography eluting with hexane/EA 10:1 to 5:1 to 3:1); dr: >20:1; yield: 49.9 mg (58%); [α]_D_^20^ + 31.0 (*c* 0.49, CH_2_Cl_2_); mp 98.8–99.8 °C; ^1^H NMR (400 MHz, CDCl_3_): δ 7.73–7.70 (m, 2H), 7.36–7.23 (m, 7H), 7.17–7.14 (m, 2H), 7.02–6.98 (m, 2H), 4.74 (d, *J* = 14.9 Hz, 1H), 4.61 (d, *J* = 14.9 Hz, 1H), 4.49 (d, *J* = 5.8 Hz, 1H), 4.23 (s, 1H), 3.85 (d, *J* = 18.4 Hz, 1H), 3.56 (dd, *J* = 18.4, 1.7 Hz, 1H), 2.84 (ddd, *J* = 12.7, 4.7, 4.7 Hz, 1H), 1.67 (s, 9H), 1.64–1.61 (m, 2H), 1.41–1.07 (m, 5H); ^13^C{^1^H} NMR (101 MHz, CDCl_3_): δ 172.9, 165.2, 161.8 (d, *J* = 245 Hz), 148.7, 144.4, 140.1, 136.7, 134.1, 134.0, 130.8 (d, *J* = 8 Hz), 128.8, 128.4, 128.1, 127.7, 127.0, 125.1, 124.3, 123.3, 115.2 (d, *J* = 21 Hz), 114.3, 84.8, 84.0, 48.8, 47.6, 46.7, 40.5, 38.7, 30.6, 28.1, 24.7, 22.9, 20.6; ^19^F {^1^H} NMR (376 MHz, CDCl_3_): δ −115.3 (s, 1F); HRMS (FAB) *m*/*z*: calcd for C_37_H_37_FN_2_O_5_ [M + H]^+^, 609.2765; found, 609.2775; 94% ee was determined by HPLC analysis [chiralpak AD-H, *n*-hexane/*i*-PrOH 50:50, 0.2 mL/min, λ = 220 nm, *t*_major_ = 30.32 min, *t*_minor_ = 23.63 min].

### *tert*-Butyl (*R*)-3-((4a*R*,8a*R*,9*R*)-2-Benzyl-9-(4-chlorophenyl)-3-oxo-2,3,5,6,7,8,8a,9-octahydrochromeno[2,3-*c*]pyrrol-4a(1*H*)-yl)-2-oxoindoline-1-carboxylate (**5bf**)

White powder (purified by silica gel column chromatography eluting with hexane/EA 10:1 to 5:1 to 3:1); dr: >20:1; yield: 47 mg (90%); [α]_D_^20^ + 31.4 (*c* 0.49, CH_2_Cl_2_); mp 133.1–134.1 °C; ^1^H NMR (400 MHz, CDCl_3_): δ 7.73–7.70 (m, 2H), 7.35–7.23 (m, 9H), 7.13 (d, *J* = 8.4 Hz, 2H), 4.73 (d, *J* = 14.9 Hz, 1H), 4.61 (d, *J* = 14.9 Hz, 1H), 4.49 (t, *J* = 6.4 Hz, 1H), 4.23 (s, 1H), 3.85 (dd, *J* = 18.5, 1.7 Hz, 1H), 3.56 (dd, *J* = 18.5, 1.7 Hz, 1H), 2.85 (ddd, *J* = 12.7, 5.9, 3.8 Hz, 1H), 1.67 (s, 9H), 1.64–1.59 (m, 2H), 1.40–1.02 (m, 5H); ^13^C{^1^H} NMR (101 MHz, CDCl_3_): δ 172.9, 165.2, 148.7, 144.4, 140.1, 136.9, 136.7, 132.8, 130.7, 128.8, 128.5, 128.4, 128.1, 127.8, 127.7, 126.9, 125.2, 124.2, 122.9, 114.3, 84.9, 84.0, 48.7, 47.6, 46.7, 40.6, 38.6, 30.6, 28.1, 24.6, 22.9, 20.5; HRMS (FAB) *m*/*z*: calcd for C_37_H_37_ClN_2_O_5_ [M + H]^+^, 625.2469; found, 625.2475; 91% ee was determined by HPLC analysis [chiralpak AD-H, *n*-hexane/*i*-PrOH 50:50, 0.2 mL/min, λ = 220 nm, *t*_major_ = 29.91 min, *t*_minor_ = 21.17 min].

### *tert*-Butyl (*R*)-3-((4a*R*,8a*R*,9*R*)-2-Benzyl-9-(4-bromophenyl)-3-oxo-2,3,5,6,7,8,8a,9-octahydrochromeno[2,3-*c*]pyrrol-4a(1*H*)-yl)-2-oxoindoline-1-carboxylate (**5bg**)

White powder (purified by silica gel column chromatography eluting with hexane/EA 10:1 to 5:1 to 3:1); dr: >20:1; yield: 43 mg (76%); [α]_D_^20^ + 29.2 (*c* 0.48, CH_2_Cl_2_); mp 208.0–209.0 °C; ^1^H NMR (400 MHz, CDCl_3_): δ 7.71 (d, *J* = 4.2 Hz, 2H), 7.43 (d, *J* = 8.4 Hz, 2H), 7.35–7.23 (m, 7H), 7.08 (d, *J* = 8.4 Hz, 2H), 4.72 (d, *J* = 14.9 Hz, 1H), 4.62 (d, *J* = 14.8 Hz, 1H), 4.47 (d, *J* = 5.8 Hz, 1H), 4.22 (s, 1H), 3.84 (dd, *J* = 18.5, 1.1 Hz, 1H), 3.55 (dd, *J* = 18.5, 1.8 Hz, 1H), 2.85 (ddd, *J* = 12.8, 5.9, 3.9 Hz, 1H), 1.67 (s, 9H), 1.64–1.56 (m, 2H), 1.40–1.03 (m, 5H); ^13^C{^1^H} NMR (101 MHz, CDCl_3_): δ 172.9, 165.1, 148.7, 144.5, 140.1, 137.5, 136.7, 131.5, 131.1, 128.8, 128.4, 128.1, 127.7, 127.0, 125.1, 124.2, 122.8, 120.9, 114.3, 84.9, 84.0, 48.8, 47.6, 46.7, 40.7, 38.6, 30.6, 28.1, 24.7, 22.9, 20.5; HRMS (FAB) *m*/*z*: calcd for C_37_H_37_BrN_2_O_5_ [M + H]^+^, 669.1964; found, 669.1965; 91% ee was determined by HPLC analysis [chiralpak AD-H, *n*-hexane/*i*-PrOH 50:50, 0.2 mL/min, λ = 220 nm, *t*_major_ = 24.64 min, *t*_minor_ = 14.95 min].

### *tert*-Butyl (*R*)-3-((4a*R*,8a*R*,9*R*)-2-Benzyl-3-oxo-9-(4-(trifluoromethyl)phenyl)-2,3,5,6,7,8,8a,9-octahydro-chromeno[2,3-*c*]pyrrol-4a(1*H*)-yl)-2-oxoindoline-1-carboxylate (**5bh**)

White powder (purified by silica gel column chromatography eluting with hexane/EA 10:1 to 5:1 to 3:1); dr: >20:1; yield: 40.1 mg (61%); [α]_D_^20^ + 41.1 (*c* 0.47, CH_2_Cl_2_); mp 187.3–188.3 °C; ^1^H NMR (400 MHz, CDCl_3_): δ 7.71 (d, *J* = 1.3 Hz, 2H), 7.56 (d, *J* = 8.0 Hz, 2H), 7.33–7.22 (m, 9H), 4.72 (d, *J* = 14.9 Hz, 1H), 4.61 (d, *J* = 14.9 Hz, 1H), 4.56 (d, *J* = 5.8 Hz, 1H), 4.22 (s, 1H), 3.86 (d, *J* = 18.5 Hz, 1H), 3.56 (dd, *J* = 18.5, 1.7 Hz, 1H), 2.89 (ddd, *J* = 12.6, 5.9, 3.7 Hz, 1H), 1.66 (s, 9H), 1.62–1.57 (m, 2H), 1.40–0.99 (m, 5H); ^13^C{^1^H} NMR (101 MHz, CDCl_3_): δ 173.0, 165.2, 148.8, 144.8, 143.0, 140.3, 136.8, 129.9, 129.0, 128.6, 128.3, 127.9, 127.1, 125.4 (q, *J* = 9 Hz), 125.3, 124.3, 122.5, 114.5, 85.1, 84.2, 48.9, 47.7, 46.9, 41.3, 38.9, 30.8, 28.2, 24.8, 23.1, 20.7; ^19^F {^1^H} NMR (376 MHz, CDCl_3_): δ −63.3 (s, 3F); HRMS (FAB) *m*/*z*: calcd for C_38_H_37_F_3_N_2_O_5_ [M + H]^+^, 659.2733; found, 659.2735; 94% ee was determined by HPLC analysis [chiralpak AD-H, *n*-hexane/*i*-PrOH 50:50, 0.2 mL/min, λ = 220 nm, *t*_major_ = 25.37 min, *t*_minor_ = 19.87 min].

### *tert*-Butyl (*R*)-3-((4a*R*,8a*R*,9*R*)-2-Benzyl-9-(4-methoxyphenyl)-3-oxo-2,3,5,6,7,8,8a,9-octahydrochromeno[2,3-*c*]pyrrol-4a(1*H*)-yl)-2-oxoindoline-1-carboxylate (**5bi**)

White powder (purified by silica gel column chromatography eluting with hexane/EA 10:1 to 5:1 to 3:1); dr: >20:1; yield: 16 mg (26%); [α]_D_^20^ + 29.7 (*c* 0.33, CH_2_Cl_2_); mp 150.2–151.2 °C; ^1^H NMR (400 MHz, CDCl_3_): δ 7.72–7.70 (m, 2H), 7.32–7.22 (m, 7H), 7.09 (d, *J* = 8.7 Hz, 2H), 6.83 (d, *J* = 8.7 Hz, 2H), 4.72 (d, *J* = 14.9 Hz, 1H), 4.59 (d, *J* = 14.9 Hz, 1H), 4.43 (d, *J* = 5.8 Hz, 1H), 4.23 (s, 1H), 3.86 (d, *J* = 18.5 Hz, 1H), 3.78 (s, 3H), 3.54 (dd, *J* = 18.6, 1.8 Hz, 1H), 2.82 (ddd, *J* = 12.7, 5.9, 4.1 Hz, 1H), 1.66 (s, 9H), 1.63–1.60 (m, 2H), 1.40–1.02 (m, 5H); ^13^C{^1^H} NMR (101 MHz, CDCl_3_): δ 173.0, 158.7, 144.3, 140.3, 137.0, 130.6, 130.4, 128.9, 128.5, 128.3, 127.8, 127.2, 125.3, 124.5, 124.2, 114.4, 113.8, 84.9, 84.2, 55.4, 48.9, 47.9, 46.8, 40.5, 39.0, 30.8, 28.2, 24.9, 23.2, 20.8; HRMS (FAB) *m*/*z*: calcd for C_38_H_40_N_2_O_6_ [M + H]^+^, 621.2965; found, 621.2961; 98% ee was determined by HPLC analysis [chiralpak AD-H, *n*-hexane/*i*-PrOH 80:20, 0.2 mL/min, λ = 220 nm, *t*_major_ = 40.04 min, *t*_minor_ = 30.26 min].

### *tert*-Butyl (*R*)-3-((4a*R*,8a*R*,9*R*)-2-Benzyl-9-(naphthalen-1-yl)-3-oxo-2,3,5,6,7,8,8a,9-octahydrochromeno[2,3-*c*]pyrrol-4a(1*H*)-yl)-2-oxoindoline-1-carboxylate (**5bj**)

White powder (purified by silica gel column chromatography eluting with hexane/EA 10:1 to 5:1 to 3:1); dr: >20:1; yield: 40.8 mg (76%); [α]_D_^20^ + 124.4 (*c* 0.50, CH_2_Cl_2_); mp 211.1–212.1 °C; ^1^H NMR (400 MHz, CDCl_3_): δ 8.72 (d, *J* = 8.6 Hz, 1H), 7.88–7.66 (m, 5H), 7.52 (dd, *J* = 8.1, 6.9 Hz, 1H), 7.40–7.23 (m, 8H), 7.09 (dd, *J* = 7.2, 1.2 Hz, 1H), 5.23 (d, *J* = 6.1 Hz, 1H), 4.77 (d, *J* = 14.9 Hz, 1H), 4.65 (d, *J* = 14.9 Hz, 1H), 4.40 (s, 1H), 4.05 (d, *J* = 18.6 Hz, 1H), 3.56 (dd, *J* = 18.5, 1.6 Hz, 1H), 3.17 (ddd, *J* = 12.8, 6.1, 4.0 Hz, 1H), 1.71 (s, 9H), 1.69–1.46 (m, 2H), 1.38–1.19 (m, 3H), 1.02–0.82 (m, 2H); ^13^C{^1^H} NMR (101 MHz, CDCl_3_): δ 173.4, 165.4, 148.8, 145.0, 140.3, 136.8, 134.4, 133.9, 132.0, 128.8, 128.6, 128.4, 128.1, 127.71, 127.68, 127.2, 127.0, 126.7, 126.0, 125.2, 124.8, 124.4, 124.3, 123.7, 114.3, 84.8, 84.1, 49.0, 48.0, 46.7, 37.1, 36.8, 30.8, 28.1, 24.8, 24.2, 20.5; HRMS (FAB) *m*/*z*: calcd for C_41_H_40_N_2_O_5_ [M + H]^+^, 641.3015; found, 641.3015; 92% ee was determined by HPLC analysis [chiralpak IG, *n*-hexane/*i*-PrOH 80:20, 0.2 mL/min, λ = 220 nm, *t*_major_ = 100.06 min, *t*_minor_ = 55.48 min].

### (2*R*,4*R*)-6-Benzyl-2-methyl-2-((*S*)-2-oxoindolin-3-yl)-4-phenyl-3,4,5,6-tetrahydropyrano[2,3-*c*]pyrrol-7(2*H*)-one (**5aa**)

White powder (purified by silica gel column chromatography eluting with hexane/EA 5:1 to 2:1); 45% yield (20.2 mg); mp 111.1–112.1 °C; [α]_D_^20^ – 14.2 (*c* 1.4, CH_2_Cl_2_); ^1^H NMR (400 MHz, CDCl_3_): δ 8.13 (s, 1H), 7.65 (d, *J* = 7.5 Hz, 1H), 7.40–7.23 (m, 5H), 7.27–7.16 (m, 6H), 7.09 (t, *J* = 7.7 Hz, 1H), 6.85 (d, *J* = 7.7 Hz, 1H), 4.75 (d, *J* = 15.0 Hz, 1H), 4.52 (d, *J* = 15.0 Hz, 1H), 4.02–3.94 (m, 2H), 3.54 (s, 2H), 3.20 (dd, *J* = 14.1, 6.1 Hz, 1H), 1.91 (dd, *J* = 14.1, 11.3 Hz, 1H), 1.15 (s, 3H); ^13^C{^1^H} NMR (101 MHz, CDCl_3_): δ 175.9, 165.6, 144.6, 141.4, 141.3, 137.0, 129.0, 128.9, 128.4, 128.2, 128.0, 127.74, 127.70, 127.3, 126.3, 124.4, 123.4, 109.2, 81.3, 48.2, 47.7, 46.7, 40.2, 37.1, 22.2; HRMS (ESI) *m*/*z*: [M + H]^+^ calcd for C_29_H_27_N_2_O_3_, 451.2016; found, 451.1999; 94% ee was determined by HPLC analysis [chiralpak AD-H, hexane/*i*-PrOH = 70/30, flow rate = 0.4 mL/min, λ = 220 nm, *t*_minor_ = 38.32 min, *t*_major_ = 41.08 min].

### *tert*-Butyl (*R*)-3-((4a*R*,8a*R*,9*R*)-2-Benzyl-3-oxo-9-phenyl-2,3,5,6,7,8,8a,9-octahydrochromeno[2,3-*c*]pyrrol-4a(1*H*)-yl)-5-fluoro-2-oxoindoline-1-carboxylate (**5ca**)

White powder (purified by silica gel column chromatography eluting with hexane/EA 10:1 to 5:1 to 3:1); dr: >20:1; yield: 38.5 mg (76%); [α]_D_^20^ + 13.4 (*c* 0.47, CH_2_Cl_2_); mp 174.5–175.5 °C; ^1^H NMR (400 MHz, CDCl_3_): δ 7.71 (dd, *J* = 9.0, 4.5 Hz, 1H), 7.51 (dd, *J* = 8.2, 1.1 Hz, 1H), 7.33–7.17 (m, 10H), 7.01 (td, *J* = 8.8, 2.8 Hz, 1H), 4.71 (d, *J* = 14.9 Hz, 1H), 4.61 (d, *J* = 14.9 Hz, 1H), 4.45 (d, *J* = 5.8 Hz, 1H), 4.23 (s, 1H), 3.90 (d, *J* = 18.6 Hz, 1H), 3.54 (dd, *J* = 18.6, 1.7 Hz, 1H), 2.85 (ddd, *J* = 12.8, 5.9, 3.9 Hz, 1H), 1.65 (s, 9H), 1.62–1.57 (m, 2H), 1.41–1.00 (m, 5H); ^13^C{^1^H} NMR (101 MHz, CDCl_3_): δ 172.5, 165.3, 160.1 (d, *J* = 243 Hz), 148.8, 144.3, 138.5, 137.0, 136.3, 129.6, 128.9, 128.5, 128.4, 127.8, 127.1, 126.3 (d, *J* = 9 Hz), 123.9, 115.7, 115.6, 115.1 (d, *J* = 23 Hz), 114.9, 114.6, 85.1, 84.1, 49.2, 47.9, 46.8, 41.3, 38.9, 30.9, 28.2, 24.9, 23.2, 20.7; ^19^F {^1^H} NMR (376 MHz, CDCl_3_): δ −115.8 (s, 1F); HRMS (FAB) *m*/*z*: calcd for C_37_H_37_FN_2_O_5_ [M + H]^+^, 609.2765; found, 609.2761; 71% ee was determined by HPLC analysis [chiralpak AD-H, *n*-hexane/*i*-PrOH 50:50, 0.2 mL/min, λ = 220 nm, *t*_major_ = 23.20 min, *t*_minor_ = 19.24 min].

### *tert*-Butyl (*R*)-3-((4a*R*,8a*R*,9*R*)-2-Benzyl-3-oxo-9-phenyl-2,3,5,6,7,8,8a,9-octahydrochromeno[2,3-*c*]pyrrol-4a(1*H*)-yl)-6-chloro-2-oxoindoline-1-carboxylate (**5da**)

White powder (purified by silica gel column chromatography eluting with hexane/EA 10:1 to 5:1 to 3:1); dr: >20:1; yield: 45.7 mg (99%); [α]_D_^20^ – 0.8 (*c* 0.50, CH_2_Cl_2_); mp 168.5–169.5 °C; ^1^H NMR (400 MHz, CDCl_3_): δ 7.79 (d, *J* = 2.0 Hz, 1H), 7.64 (d, *J* = 1.0 Hz, 1H), 7.32–7.16 (m, 11H), 4.72 (d, *J* = 14.9 Hz, 1H), 4.59 (d, *J* = 14.9 Hz, 1H), 4.45 (d, *J* = 5.8 Hz, 1H), 4.21 (s, 1H), 3.90 (d, *J* = 18.6 Hz, 1H), 3.55 (dd, *J* = 18.6, 1.8 Hz, 1H), 2.83 (ddd, *J* = 12.8, 5.9, 3.9 Hz, 1H), 1.66 (s, 9H), 1.66–1.60 (m, 2H), 1.41–0.97 (m, 5H); ^13^C{^1^H} NMR (101 MHz, CDCl_3_): δ 172.5, 165.4, 148.6, 144.3, 141.2, 138.4, 136.9, 134.3, 129.6, 128.9, 128.5, 128.3, 128.1, 127.8, 127.2, 125.3, 123.9, 122.8, 115.3, 85.5, 84.1, 48.7, 47.9, 46.8, 41.3, 38.9, 30.8, 28.2, 24.9, 23.2, 20.7; HRMS (FAB) *m*/*z*: calcd for C_37_H_37_ClN_2_O_5_ [M + H]^+^, 625.2469; found, 625.2466; 96% ee was determined by HPLC analysis [chiralpak AD-H, *n*-hexane/*i*-PrOH 50:50, 0.2 mL/min, λ = 220 nm, *t*_major_ = 25.53 min, *t*_minor_ = 18.72 min].

### *tert*-Butyl (*R*)-3-((4a*R*,8a*S*,9*R*)-2-Benzyl-3-oxo-9-phenyl-1,2,3,5,6,8,8a,9-octahydro-4aH-pyrano[3′,4′:5,6]pyrano[2,3-*c*]pyrrol-4a-yl)-2-oxoindoline-1-carboxylate (**5ea**)

White powder (purified by silica gel column chromatography eluting with hexane/EA 10:1 to 5:1 to 3:1); dr: >20:1; yield: 33.1 mg (67%); [α]_D_^20^ + 33.5 (*c* 0.49, CH_2_Cl_2_); mp 169.0–170.0 °C; ^1^H NMR (400 MHz, CDCl_3_): δ 7.77–7.72 (m, 2H), 7.34–7.23 (m, 10H), 7.17–7.15 (m, 2H), 4.75 (d, *J* = 14.9 Hz, 1H), 4.59 (d, *J* = 14.9 Hz, 1H), 4.49 (d, *J* = 5.6 Hz, 1H), 4.21 (s, 1H), 3.90 (d, *J* = 18.6 Hz, 1H), 3.74–3.56 (m, 3H), 3.43–3.36 (m, 1H), 3.25–3.18 (m, 1H), 1.65 (s, 9H), 1.52–1.49 (m, 2H); ^13^C{^1^H} NMR (101 MHz, CDCl_3_): δ 172.2, 165.1, 148.9, 144.4, 140.5, 137.1, 136.7, 129.0, 128.9, 128.3, 127.9, 127.6, 127.1, 125.3, 123.7, 123.4, 114.6, 85.1, 81.8, 64.8, 63.2, 48.7, 47.8, 46.9, 38.8, 37.8, 31.2, 28.2; HRMS (FAB) *m*/*z*: calcd for C_36_H_36_N_2_O_6_ [M + H]^+^, 593.2652; found, 593.2642; 58% ee was determined by HPLC analysis [chiralpak AD-H, *n*-hexane/*i*-PrOH 50:50, 0.2 mL/min, λ = 220 nm, *t*_major_ = 15.18 min, *t*_minor_ = 11.63 min].

### (3*R*,3a′*R*,7′*S*,7a′*S*)-2′-Benzyl-3a′-hydroxy-5′,7′-diphenyl-1′,2′,7′,7a′-tetrahydrospiro[indoline-3,4′-isoindole]-2,3′(3a′*H*)-dione (**6**)

Brown oil (purified by silica gel column chromatography eluting with hexane/EA 10:1 to 5:1 to 2:1); dr: >20:1; yield: 45.8 mg (90%); [α]_D_^20^ + 23.6 (*c* 0.50, CH_2_Cl_2_); ^1^H NMR (400 MHz, CDCl_3_): δ 9.26 (s, 1H), 7.48–7.16 (m, 11H), 7.16–6.99 (m, 6H), 6.99–6.86 (m, 2H), 6.78 (d, *J* = 1.1 Hz, 1H), 6.16 (d, *J* = 2.1 Hz, 1H), 4.40 (d, *J* = 15.1 Hz, 1H), 4.21 (d, *J* = 15.1 Hz, 1H), 4.12 (dd, *J* = 10.9, 2.2 Hz, 1H), 3.56 (t, *J* = 9.2 Hz, 1H), 3.02 (dd, *J* = 9.0, 6.9 Hz, 1H), 2.96–2.71 (m, 1H); ^13^C{^1^H} NMR (101 MHz, CDCl_3_): δ 179.1, 172.2, 143.1, 142.2, 139.8, 137.6, 136.1, 134.0, 129.4, 129.2, 129.1, 128.7, 128.1, 127.9, 127.8, 127.7, 127.5, 127.2, 125.0, 122.3, 111.7, 78.3, 56.2, 47.9, 46.4, 44.0, 42.0; HRMS (ESI) *m*/*z*: calcd for C_34_H_28_N_2_O_3_Na [M + Na]^+^, 535.1992; found, 535.1999; 94% ee was determined by HPLC analysis [chiralpak AD-H, *n*-hexane/*i*-PrOH 50:50, 0.2 mL/min, λ = 220 nm, *t*_major_ = 207.68 min, *t*_minor_ = 138.77 min].

## Data Availability

The data underlying this study are available in the published article and its Supporting Information.

## Supporting Information Available

General information, optimization studies, DFT calculations for **5ba** and **5ba′**, X-ray analysis data, NMR spectra, and HPLC spectra of all compounds (PDF)

## References

[ref1] aBreuer M.; Ditrich K.; Habicher T.; Hauer B.; Kesseler M.; Sturmer R.; Zelinski T. Industrial Methods for the Production of Optically Active Intermediates. Angew. Chem., Int. Ed. 2004, 43, 788 10.1002/anie.200300599. 14767950

[ref2] Sarabu R. Tricyclic Compounds. U.S. Patent 20,110,313,002 A1, 2011.

[ref3] Sidhu P. S.; Mosier P. D.; Zhou Q.; Desai U. R. On Scaffold Hopping: Challenges in the Discovery of Sulfated Small Molecules as Mimetics of Glycosaminoglycans. Bioorg. Med. Chem. Lett. 2013, 23, 355–359. 10.1016/j.bmcl.2012.10.079. 23164711 PMC3525718

[ref4] Fell J. B.; Mohr P.; Stengel P. J. Heterocyclic Antiviral Compounds. U.S. Patent 0,095,739 A1, 2008.

[ref5] Kai H.; Taoda Y.; Endoh T.; Asahi K.; Tobinaga H. Pyrolinone Derivative and Pharmaceutical Composition Comprising the Same. U.S. Patent 8,575,197 B2, 2013.

[ref6] Elshamy A. I.; Nassar M. I.; Mohamed T. A.; Hegazy M. E. F. Chemical and Biological Profile of Cespitularia Species: A Mini Review. J. Adv. Res. 2016, 7, 209–224. 10.1016/j.jare.2015.07.003. 26966562 PMC4767810

[ref7] aYe L. W.; Shu C.; Gagosz F. Recent Progress towards Transition Metal-Catalyzed Synthesis of γ-Lactams. Org. Biomol. Chem. 2014, 12, 1833–1845. 10.1039/C3OB42181C. 24473105

[ref8] aLi J. L.; Fu L.; Wu J.; Yang K. C.; Li Q. Z.; Gou X. J.; Peng C.; Han B.; Shen X. D. Highly Enantioselective Synthesis of Fused Bicyclic Dihydropyranones via Low-Loading N-Heterocyclic Carbene Organocatalysis. Chem. Commun. 2017, 53, 6875–6878. 10.1039/C7CC02921G. 28604911

[ref9] aLi J.-L.; Yang K.-C.; Li Y.; Li Q.; Zhu H.-P.; Han B.; Peng C.; Zhi Y.-G.; Gou X.-J. Asymmetric Synthesis of Bicyclic Dihydropyrans via Organocatalytic Inverse-Electron-Demand Oxo-Diels–Alder Reactions of Enolizable Aliphatic Aldehydes. Chem. Commun. 2016, 52, 10617–10620. 10.1039/C6CC05001H. 27436351

[ref10] aLi Q.; Zhou L.; Shen X.-D.; Yang K.-C.; Zhang X.; Dai Q.-S.; Leng H.-J.; Li Q.-Z.; Li J.-L. Stereoselective Construction of Halogenated Quaternary Carbon Centers by Brønsted Base Catalyzed [4 + 2] Cycloaddition of α-Haloaldehydes. Angew. Chem., Int. Ed. 2018, 57, 1913–1917. 10.1002/anie.201711813. 29276812

[ref11] Wu M.; Han Z.; Li K.; Wu J.; Ding K.; Lu Y. Cyclohexyl-Fused, Spirobiindane-Derived, Phosphine-Catalyzed Synthesis of Tricyclic γ-Lactams and Kinetic Resolution of γ-Substituted Allenoates. J. Am. Chem. Soc. 2019, 141, 16362–16373. 10.1021/jacs.9b07418. 31545594

[ref12] Huang B.-W.; Han J.-L. Regioselectivity Switch between Enantioselective 1,2- and 1,4-Addition of Allyl Aryl Ketones with 2,3-Dioxopyrrolidines. J. Org. Chem. 2023, 88, 16376–16390. 10.1021/acs.joc.3c01885. 37948045

[ref13] Millemaggi A.; Taylor R. J. K. 3-Alkenyl-Oxindoles: Natural Products, Pharmaceuticals, and Recent Synthetic Advances in Tandem/Telescoped Approaches. Eur. J. Org Chem. 2010, 2010, 4527–4547. 10.1002/ejoc.201000643.

[ref14] aCurti C.; Rassu G.; Zambrano V.; Pinna L.; Pelosi G.; Sartori A.; Battistini L.; Zanardi F.; Casiraghi G. Bifunctional Cinchona Alkaloid/Thiourea Catalyzes Direct and Enantioselective Vinylogous Michael Addition of 3-Alkylidene Oxindoles to Nitroolefins. Angew. Chem., Int. Ed. 2012, 51, 6200–6204. 10.1002/anie.201202027. 22565658

[ref15] aRassu G.; Zambrano V.; Tanca R.; Sartori A.; Battistini L.; Zanardi F.; Curti C.; Casiraghi G. 3-Alkenyl-2-Silyloxyindoles: An Enabling, Yet Understated Progeny of Vinylogous Carbon Nucleophiles. Eur. J. Org Chem. 2012, 2012, 466–470. 10.1002/ejoc.201101446.

[ref16] Feng J.; Li X. Enantioselective Vinylogous Michael-Michael Cascade Reactions of 3-Alkylidene Oxindoles and Nitroolefin Enoates. J. Org. Chem. 2017, 82, 7317–7323. 10.1021/acs.joc.7b00938. 28650159

[ref17] aHan J. L.; Chang C. H. An Asymmetric Assembly of Spirooxindole Dihydropyranones through a Direct Enantioselective Organocatalytic Vinylogous Aldol-Cyclization Cascade Reaction of 3-Alkylidene Oxindoles with Isatins. Chem. Commun. 2016, 52, 2322–2325. 10.1039/C5CC08883F. 26728396

[ref18] Jaiswal M. K.; Singh B.; De S.; Singh N.; Singh R. P. Stereoselective Formal [3 + 3] Annulation of 3-Alkylidene-2-Oxindoles with β,γ-Unsaturated α-Keto Esters. Org. Biomol. Chem. 2020, 18, 9852–9862. 10.1039/D0OB02046J. 33295933

[ref19] aChen I.-T.; Guan R.-Y.; Han J.-L. Asymmetric Sequential Vinylogous Mannich/Annulation/Acylation Process of 2-Ethylidene 1,3-Indandiones and Isatin N-Boc Ketimines: Access to Chiral Spiro-Oxindole Piperidine Derivatives. Adv. Synth. Catal. 2022, 364, 2613–2619. 10.1002/adsc.202200465.

[ref20] Recently Singh et al. reported a [4 + 2]-type annulation reaction between unsaturated thiazolones and 3-isopropylidene oxindoles while preparing this manuscript, see: Manna A.; Rohilla S.; Singh V. K. Enantioselective Synthesis of Thiazolopyran Derivatives via a Direct Vinylogous Michael–*oxa*-Michael Sequence. Org. Lett. 2024, 26, 280–285. 10.1021/acs.orglett.3c03971. 38127653

[ref21] For more details, see the Supporting Information.

[ref22] aTakemoto Y. Recognition and Activation by Ureas and Thioureas: Stereoselective Reactions Using Ureas and Thioureas as Hydrogen-Bonding Donors. Org. Biomol. Chem. 2005, 3, 4299 10.1039/b511216h. 16327888

[ref23] Han J.-L.; Tsai Y.-D.; Chang C.-H. Asymmetric Synthesis of Spirooxindole δ-Lactones with Vicinal Tertiary and Quaternary Stereocenters via Regio-Diastereo-and Enantioselective Organocatalytic Vinylogous Aldol-Cyclization Cascade Reaction. Adv. Synth. Catal. 2017, 359, 4043–4049. 10.1002/adsc.201701104.

[ref24] Huang Y.; Li Y.; Sun J.; Li J.; Zha Z.; Wang Z. Enantioselective Hetero-Diels–Alder Reaction and the Synthesis of Spiropyrrolidone Derivatives. J. Org. Chem. 2018, 83, 8464–8472. 10.1021/acs.joc.8b01057. 29974745

