# Supplementary material for: Asymmetric (3 + 3) and (4 + 2) Annulation Reactions of 2,3-Dioxopyrrolidines with 3-Alkylidene Oxindoles to Construct Diverse Chiral Heterocyclic Frameworks
Source: J Org Chem. 2024 Jun 8;89(12):8970–84. doi: 10.1021/acs.joc.4c00933 (PMC11197100; doi:10.1021/acs.joc.4c00933)
Supplement: Supplementary file 1 — jo4c00933_si_001.pdf [file jo4c00933_si_001.pdf]

## Supporting Information

# Asymmetric (3+3) and (4+2) Annulation Reactions of 2,3-Dioxopyrrolidines with 3-Alkylidene Oxindoles to Construct Diverse Chiral Heterocyclic Frameworks

Shi-Hang Huang[§], I-Ting Chen[§] and Jeng-Liang Han*
Department of Chemistry, National Chung Hsing University, 145 Xingda Rd., South Dist., Taichung City 402, Taiwan
E-mail: jlhan@nchu.edu.tw

**Table of Contents:**

1. **General Experimental Details……………………………………………..…....S2**
2. **Starting Materials……………………..……….……………………….…….....S3**
3. **Optimization of Reaction Conditions…………………………………………...S4**
4. **Computational Methods………………………………………………………S8**
5. **References……………………………………….………………………….S8**
6. **Absolute Configuration and X-Ray Analysis Data……………….…………...S10**
7. **Copies of NMR Spectra of Products…………………………….……………..S61**
8. **Copies of HPLC Spectra of Products…..……………………….……………..S138**
9. **Cartesian Coordinates of 5ba and 5ba'……………………………………S173**

## 1. General Experimental Details

All commercially available reagents were used without further purification unless otherwise stated. All reaction solvents were purified before use. Proton nuclear magnetic resonance ($^1$H NMR) spectra were recorded on a commercial instrument at 400 MHz. Carbon-13 nuclear magnetic resonance ($^{13}$C{$^1$H } NMR) spectra were recorded at 100 MHz. The proton signal for residual non-deuterated solvent (δ 7.26 for $CHCl_3$) was used as an internal reference for $^1$H NMR spectra. For $^{13}$C{$^1$H} NMR spectra, chemical shifts are reported relative to the δ 77.0 resonance of $CHCl_3$. Coupling constants are reported in Hz. Melting points were determined on a BUCHI B-545 melting point apparatus and are uncorrected. High resolution mass spectra were recorded on a Thermo Fisher Scientific LTQ Orbitrap XL mass spectrometer. The single crystal was measured by Bruker D8 VENTURE X-ray Single Crystal Diffractometer. Analytical thin-layer chromatography (TLC) was performed on silica gel 60 F254 pre-coated plates with visualization under UV light. Column chromatography was generally performed using 40-63 μm (230-400 mesh) silica gel, typically using a 50-100:1 weight ratio of silica gel to crude product. The *ee* value determination was carried out using chiral high-performance liquid chromatography (HPLC) with Daicel Chiralpak AD-H or Chiralpak IG and IF columns on JASCO with a UV-2075 detector or a UV-4075 detector.

## 2. Starting Materials

### Catalysts:

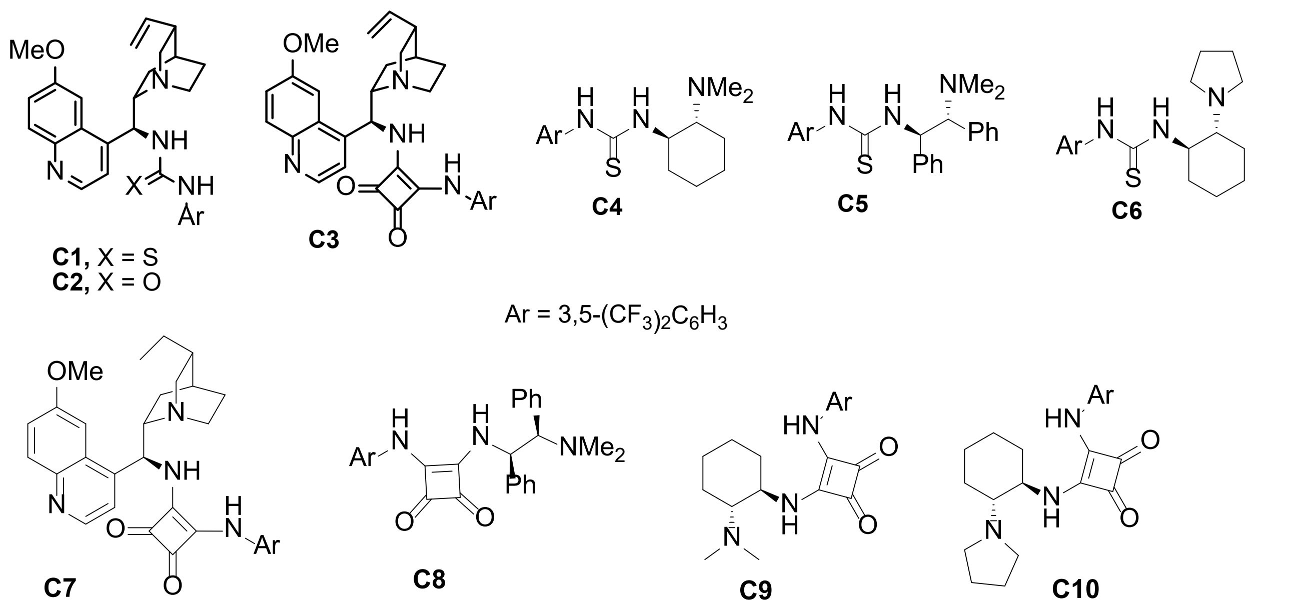


Catalysts **C1**[1], **C2**[1], **C3**[2], and **C7**[1] were prepared according to known procedures. Other catalysts are commercially available

## 3. Optimization of Reaction Conditions

### 3.1 Optimization of the [3+3] annulation reaction

An initial experiment comprising 2,3-dioxopyrrolidine **1a** as the acceptor and 3-alkylidene oxindole **2a** as the vinylogous donor in $CH_2Cl_2$ at rt was conducted using 20 mol% of **C1** as the catalyst. We discovered the [3+3] annulation product **3aa** was obtained in 75% yield, 91% ee and >20:1 dr (Table S1, entry 1). We then examined several organocatalysts and better results were obtained when **C2** was used as the catalyst (entries 2-6). The screening of catalyst loadings and reaction concentration did not improve the outcomes (entries 7-10). The investigations of various solvents showed the $CH_3CN$ gave the similar enantioselectivity, diastereoselectivity and product yield (entries 11-14). Examination of the reaction temperature did not improve the outcomes (entries 15 and 16). In addition, small amounts (6-24% yield) of vinylogous Michael adduct **3aa-1** were found in some conditions (see Table 1). This result indicated that [3+3] annulation is through vinylogous Michael–aldol cascade reaction. Finally, the optimal conditions for the [3+3] annulation reaction were chosen by performing the reaction in $CH_2Cl_2$ (entry 2, condition A) or $CH_3CN$ (entry 14, condition B) at rt with 20 mol% of **C1**.

**Table S1.** Optimization of the [3+3] annulation reaction[a]

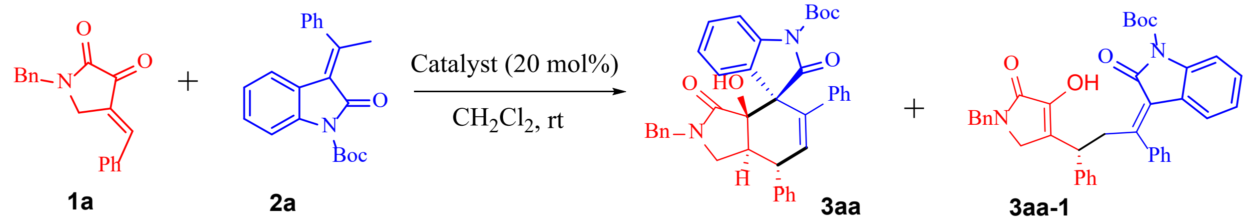


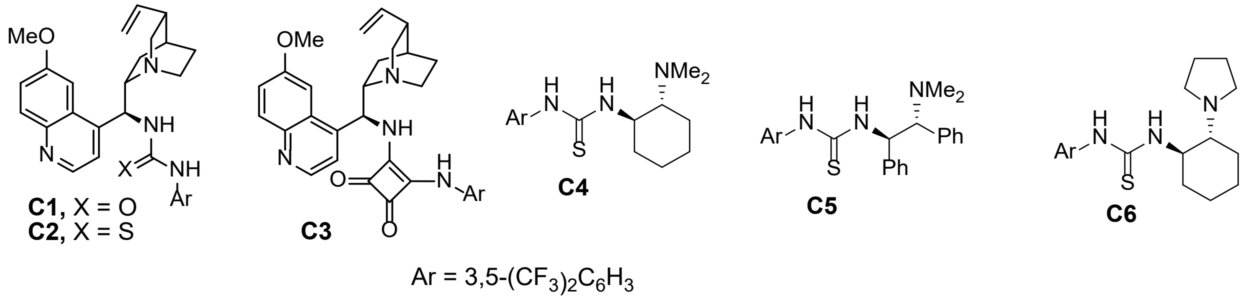


| Entry | Cat. | Solvent | Time (h) | **3aa** Yield (%)[b] | **3aa-1** Yield (%)[b] | ee (%)[c] | dr[d] |
|---|---|---|---|---|---|---|---|
| 1 | **C1** | $CH_2Cl_2$ | 24 | 75 | 10 | 91 | >20:1 |
| 2 | **C2** | $CH_2Cl_2$ | 24 | 73 | <5 | 95 | >20:1 |
| 3 | **C3** | $CH_2Cl_2$ | 24 | 64 | 8 | 93 | >20:1 |
| 4 | **C4** | $CH_2Cl_2$ | 24 | 66 | 10 | 89 | >20:1 |
| 5 | **C5** | $CH_2Cl_2$ | 24 | 50 | 6 | 93 | >20:1 |
| 6 | **C6** | $CH_2Cl_2$ | 48 | trace | trace | - | - |
| 7[e] | **C2** | $CH_2Cl_2$ | 24 | 66 | 13 | 99 | >20:1 |
| 8[f] | **C2** | $CH_2Cl_2$ | 24 | 22 | 20 | 99 | >20:1 |
| 9[g] | **C2** | $CH_2Cl_2$ | 24 | 66 | 14 | 97 | >20:1 |
| 10[h] | **C2** | $CH_2Cl_2$ | 24 | 64 | 15 | 95 | >20:1 |
| 11 | **C2** | toluene | 24 | 53 | 15 | 99 | >20:1 |
| 12 | **C2** | THF | 24 | 48 | 24 | 96 | >20:1 |
| 13 | **C2** | EA | 24 | 59 | 17 | 97 | >20:1 |
| 14 | **C2** | $CH_3CN$ | 24 | 71 | <5 | 97 | >20:1 |
| 15[i] | **C2** | $CH_2Cl_2$ | 24 | 59 | 10 | 95 | >20:1 |
| 16[j] | **C2** | $CH_2Cl_2$ | 24 | 38 | 23 | 95 | >20:1 |

[a]Unless otherwise noted, the reaction was carried out by using 0.1 mmol of **1a**, 0.15 mmol of **2a**, 20 mol % of catalyst in 1.0 mL of solvent at rt (25°C) for 24-48h. [b]Isolated yields. [c]Determined by chiral HPLC analysis. [d]Determined by crude NMR analysis. [e] 10 mol% of **C2**. [f]5 mol% of **C2**. [g]0.5 mL of $CH_2Cl_2$. [h]0.25 mL of $CH_2Cl_2$. [i]50°C. [j]-10°C.

## 3.2 Attempt to convert vinylogous Michael adducts 3aa-1 to 3aa

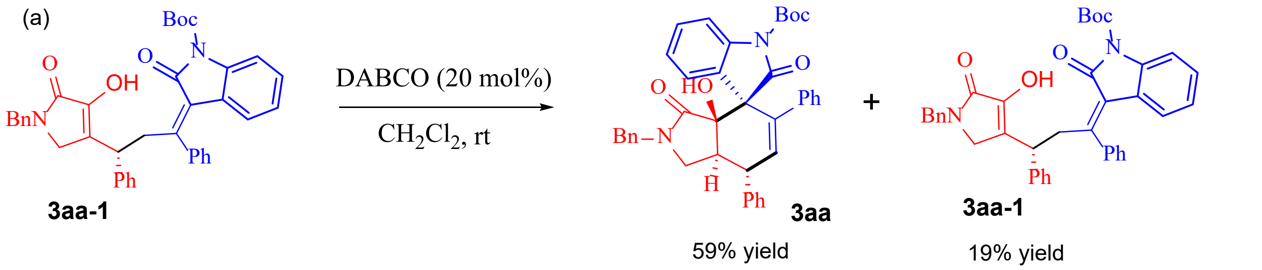


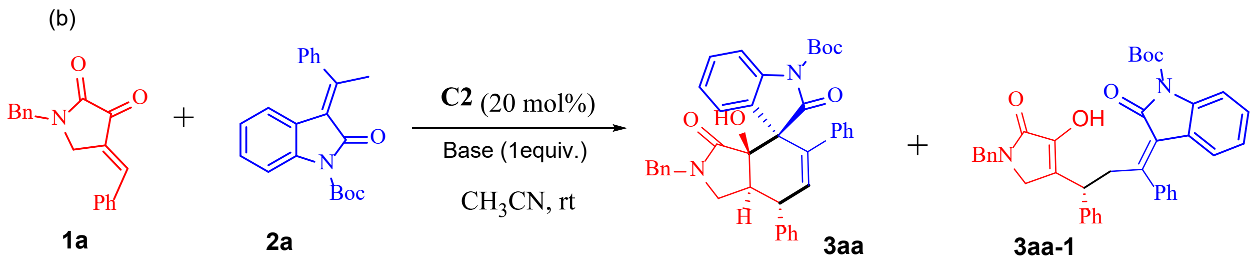


| Entry | Base | Time | Yield (3aa) | Yield (3aa-1) | *ee*% (3aa) | dr (3aa) |
|---|---|---|---|---|---|---|
| 1 | $K_2CO_3$ | 24hr | 38% | 15% | 39 | 3:2 |
| 2 | DABCO | 24hr | 58% | 18% | 73 | 1:2 |
| | | | | | | |

### 3.3 Optimization of the [4+2] annulation reaction

We then performed the reaction in $CH_2Cl_2$ at rt with 3-alkylidene oxindole **4a** as the vinylogous donor and 2,3-dioxopyrrolidine **1a** as the acceptor using quinine urea **C1** as the catalyst. As shown in Table S2, the desired [4+2] annulation product **5aa** was isolated in 25% yield, 92% ee and excellent diastereoselectivity (>20:1) after deprotection of Boc group with TFA (entry 1). We removed the Boc group because it was not easy to get good purity of product for data characterization. We then examined several catalysts bearing different hydrogen-bonding donors and quinine squaramide **C3** delivered better product yield (45%) (entries 2-7). Different solvents were tested, achieving lower product yields compared to $CH_2Cl_2$ (entries 8-12). The screening of reaction concentration did not improve the product yields (entries 13 and 14). We next tried the reaction in $CH_2Cl_2$ at rt with 3-alkylidene oxindole **4b** as the vinylogous donor and 2,3-dioxopyrrolidine **1a** as the acceptor using squaramide **C3** as the catalyst. To our delight, the desired [4+2] annulation product **5ba** was isolated in 53% yield, 96% ee and excellent diastereoselectivity (>20:1) without the need for deprotection of Boc group (Scheme S1). Hence, the optimal conditions for the [4+2] annulation reaction were chosen at rt in $CH_2Cl_2$ with 20 mol% of **C3** and 1.5 equiv. of **2c**.

**Table S2.** Optimization of the [4+2] annulation reaction[a]

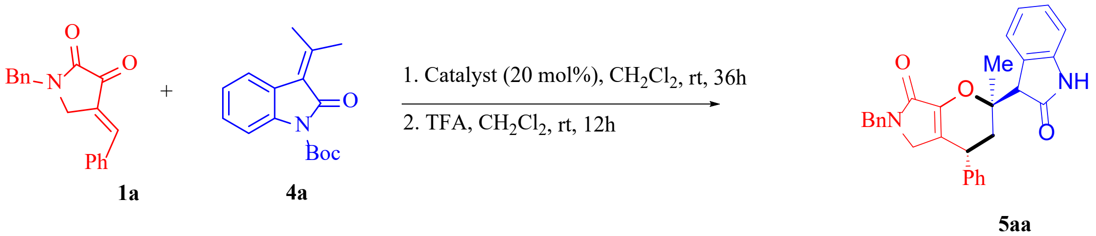


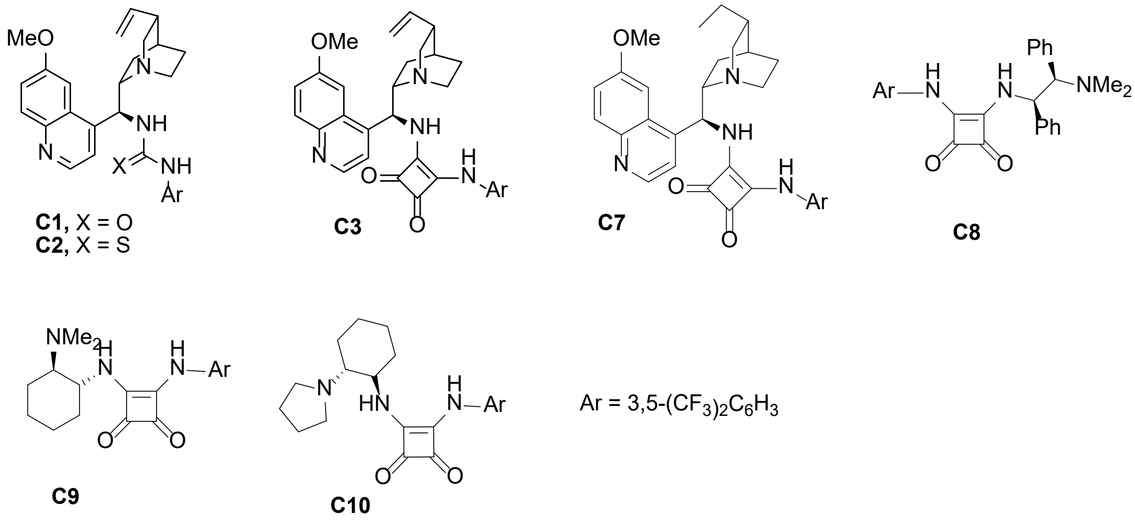


| Entry | **Cat.** | Solvent | Yield (%)[b] | ee (%)[c] | dr[d] |
|---|---|---|---|---|---|
| 1 | **C1** | $CH_2Cl_2$ | 25 | 92 | >20:1 |
| 2 | **C2** | $CH_2Cl_2$ | 32 | 92 | >20:1 |
| 3 | **C3** | $CH_2Cl_2$ | 45 | 94 | >20:1 |
| 4 | **C4** | $CH_2Cl_2$ | 22 | 90 | >20:1 |
| 5 | **C5** | $CH_2Cl_2$ | 38 | 97 | >20:1 |
| 6 | **C6** | $CH_2Cl_2$ | 20 | 86 | >20:1 |
| 7 | **C7** | $CH_2Cl_2$ | 18 | 82 | >20:1 |
| 8 | **C3** | THF | 15 | 94 | >20:1 |
| 9 | **C3** | $CH_3CN$ | 38 | 95 | >20:1 |
| 10 | **C3** | EA | 39 | 91 | >20:1 |
| 11 | **C3** | toluene | 42 | 92 | >20:1 |
| 12 | **C3** | 1,2-DCE | 18 | 93 | >20:1 |
| 13[e] | **C3** | $CH_2Cl_2$ | 25 | 90 | >20:1 |
| 14[f] | **C3** | $CH_2Cl_2$ | 23 | 91 | >20:1 |

[a]Unless otherwise noted, the reaction was carried out by using 0.1 mmol of **1a**, 0.15 mmol of **2b**, 20 mol % of catalyst in 1.0 mL of solvent at rt (25°C) for 36h, then switching to $CH_2Cl_2$ (1 mL) and TFA (1 mL) was added and stirred at rt (25°C) for 12h. [b]Isolated yields. [c]Determined by chiral HPLC analysis. [d]Determined by crude NMR analysis. [e]0.5 mL of $CH_2Cl_2$. [f]0.25 mL of $CH_2Cl_2$.

**Scheme S1.** [4+2] annulation reaction of 3-alkylidene oxindole **2c** with 2,3-dioxopyrrolidine **1a**

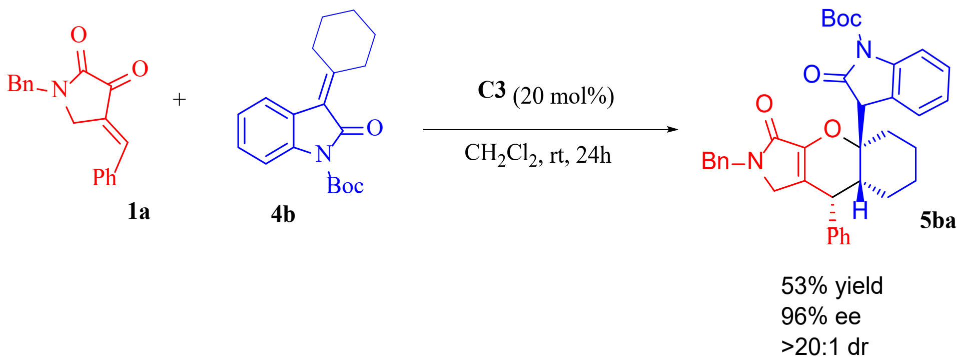


## 4. Computational Methods

All calculations reported in this paper were performed using the Gaussian16 (Revision 1.1).[3] Equilibrium structures were optimized using the Becke's three parameter functional varied by Lee-Yang-Parr correlation functional (B3LYP)[4-6], add the D3 version[7] of Grimme's dispersion with Becke-Johnson damping (B3LYP-D3(BJ)). The split-valence 6-311G(d,p)[8,9] basis set and an ultrafine integration grid within the IEFPCM model. Single point energies were calculated using M06-2X[10] add the D3 version of Grimme's dispersion (M06-2X(D3)), the def2-TZVPP[11] basis set and an ultrafine integration grid within the SMD model.

All the resulting energies were used to correct the energies obtained from the B3LYP-D3(BJ) optimizations. Computed structures are illustrated with CYLView20.[12]

## 5. References

1. (a) Vakulya, B.; Varga, S.; Csámpai, A.; Soós, T. *Org. Lett.* **2005**, *7*, 1967. (b) Asano, K.; Matsubara, S. *J. Am. Chem. Soc.* **2011**, *133*, 16711.
2. Yang, W.; Du, D.-M. *Org. Lett.* **2010**, *12*, 5450.
3. Gaussian 16, Revision 1.1, Frisch, M. J.; Trucks, G. W.; Schlegel, H. B.; Scuseria, G. E.; Robb, M. A.; Cheeseman, J. R.; Scalmani, G.; Barone, V.; Petersson, G. A.; Nakatsuji, H.; Li, X.; Caricato, M.; Marenich, A. V.; Bloino, J.; Janesko, B. G.; Gomperts, R.; Mennucci, B.; Hratchian, H. P.; Ortiz, J. V.; Izmaylov, A. F.; Sonnenberg, J. L.; Williams-Young, D.; Ding, F.; Lipparini, F.; Egidi, F.; Goings, J.; Peng, B.; Petrone, A.; Henderson, T.; Ranasinghe, D.; Zakrzewski, V. G.; Gao, J.; Rega, N.; Zheng, G.; Liang, W.; Hada, M.; Ehara, M.; Toyota, K.; Fukuda, R.; Hasegawa, J.; Ishida, M.; Nakajima, T.; Honda, Y.; Kitao, O.; Nakai, H.; Vreven, T.; Throssell, K.; Montgomery, J. A., Jr.; Peralta, J. E.; Ogliaro, F.; Bearpark, M. J.; Heyd, J. J.; Brothers, E. N.; Kudin, K. N.; Staroverov, V. N.; Keith, T. A.; Kobayashi, R.; Normand, J.; Raghavachari, K.; Rendell, A. P.; Burant, J. C.; Iyengar, S. S.; Tomasi, J.; Cossi, M.; Millam, J. M.; Klene, M.; Adamo, C.; Cammi, R.; Ochterski, J. W.; Martin, R. L.; Morokuma, K.; Farkas, O.; Foresman, J. B.; Fox, D. J. Gaussian, Inc., Wallingford CT, 2016.

4. Becke, A. D. *J. Chem. Phys.* **1992**, *96*, 2155.
5. Becke, A. D. *J. Chem. Phys.* **1993**, *98*, 5648.
6. Lee, C.; Yang, W.; Parr, R. G. *J. Chem. Phys.* **1980**, *72*, 5639.
7. Grimme, S.; Ehrlich, S.; Goerigk, L. *J. Comp. Chem*. **2011**, *32*, 1456.
8. Tomasi, J.; Mennucci, B.; Cammi, R. *Chem. Rev*. **2005**, *105*, 2999.
9. Raghavachari, K.; Binkley, J. S.; Seeger, R.; Pople, J. A. *J. Chem. Phys.* **1980**, *72*, 650.
10. Zhao, Y.; Truhlar, D. G. *Theor. Chem. Acc.* **2008**, *120*, 215.
11. Weigend, F.; Ahlrichs, R. *Phys. Chem. Chem. Phys.*, **2005**, *7,* 3297.
12. CYLview20; C. Y. Legault, Université de Sherbrooke, **2020** (http://www.cylview.org).

## 6. Absolute Configuration and X-Ray Analysis Data

**CCDC 2327247 (3ka)**

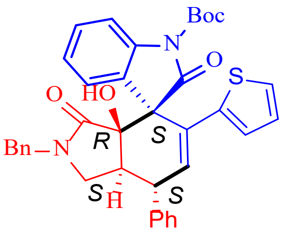


**3ka**

ORTEP drawing of **3ka** showing thermal ellipsoids at the 50% probability level

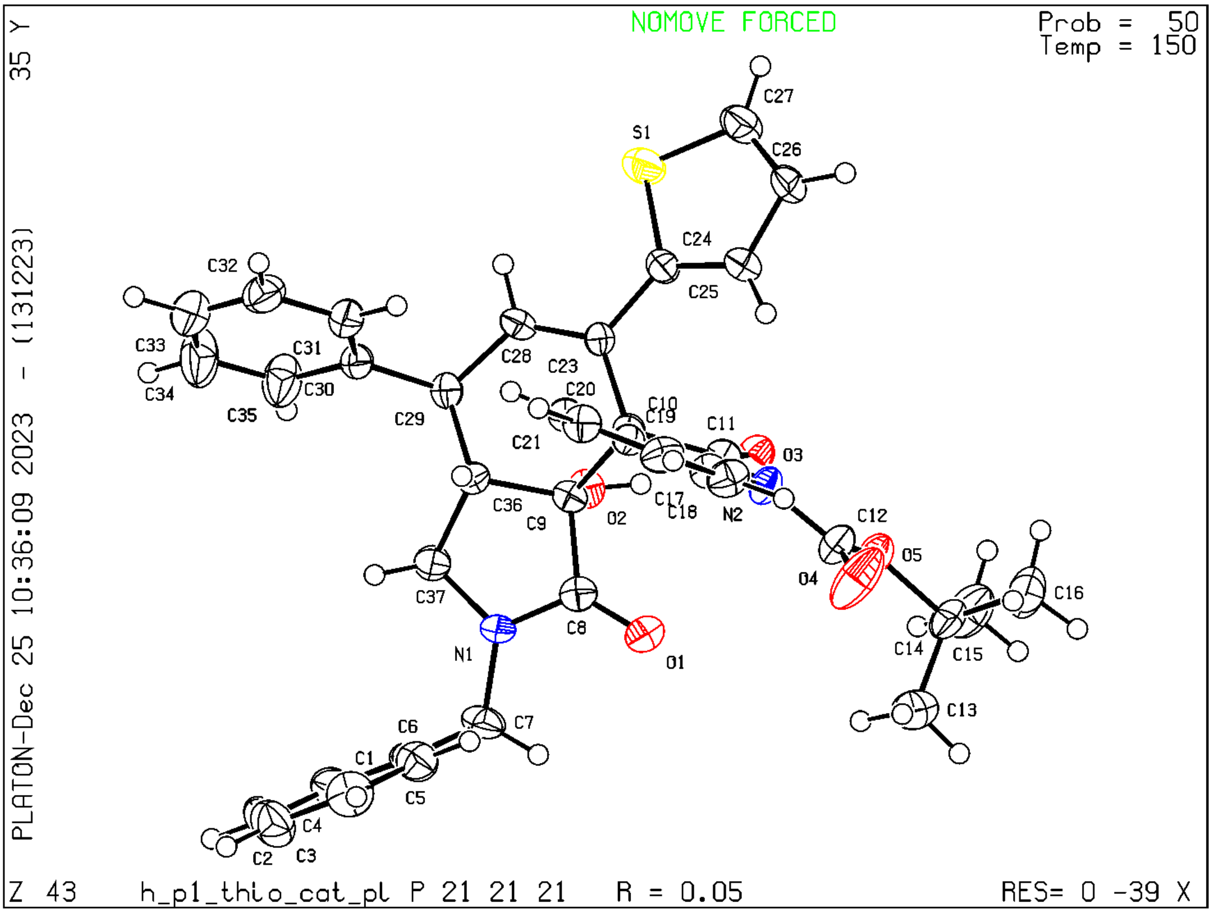


The crystal was obtained by slow evaporation of **3ka** in Hexane : DCM = 3:1 at room temperature.

**Table S3**. Crystal data and structure refinement for **3ka**.

| | | |
|---|---|---|
| Identification code | h_p1_thio_cat_pl | |
| Empirical formula | C37 H34 N2 O5 S | |
| Formula weight | 618.72 | |
| Temperature | 150.15 K | |
| Wavelength | 0.71076 Å | |
| Crystal system | Orthorhombic | |
| Space group | $P2_12_12_1$ | |
| Unit cell dimensions | a = 9.3784(10) Å | a= 90°. |
| | b = 17.1241(19) Å | b= 90°. |
| | c = 19.794(2) Å | g = 90°. |
| Volume | 3178.9(6) $Å^3$ | |
| Z | 4 | |
| Density (calculated) | 1.293 $Mg/m^3$ | |
| Absorption coefficient | 0.149 $mm^{-1}$ | |
| F(000) | 1304 | |
| Crystal size | | |
| Theta range for data collection | 2.682 to 26.432°. | |
| Index ranges | -11<=h<=11, -21<=k<=21, -24<=l<=24 | |
| Reflections collected | 84336 | |
| Independent reflections | 6318 [R(int) = 0.1212] | |
| Completeness to theta = 25.243° | 98.7 % | |
| Absorption correction | None | |
| Refinement method | Full-matrix least-squares on $F^2$ | |
| Data / restraints / parameters | 6318 / 0 / 411 | |
| Goodness-of-fit on $F^2$ | 1.127 | |
| Final R indices [I>2sigma(I)] | R1 = 0.0462, wR2 = 0.0873 | |
| R indices (all data) | R1 = 0.1306, wR2 = 0.1219 | |
| Absolute structure (Flack) parameter | -0.04(16) | |
| Extinction coefficient | n/a | |
| Largest diff. peak and hole | 0.254 and -0.473 e.$Å^{-3}$ | |

**Table S4**. Atomic coordinates ( x $10^4$) and equivalent isotropic displacement parameters ($Å^2$x $10^3$) for **3ka**. U(eq) is defined as one third of the trace of the orthogonalized $U^{ij}$ tensor.

| | x | y | z | U(eq) |
|---|---|---|---|---|
| S(1) | 2617(2) | 2373(1) | 2893(1) | 44(1) |
| O(1) | 7396(3) | -748(2) | 3752(2) | 33(1) |
| O(2) | 4264(3) | -438(2) | 3258(2) | 30(1) |
| O(3) | 6052(3) | 419(2) | 2465(2) | 33(1) |
| O(4) | 10239(4) | 766(3) | 3117(2) | 65(1) |
| O(5) | 8850(3) | 326(2) | 2275(2) | 36(1) |
| N(1) | 5556(4) | -1227(2) | 4407(2) | 30(1) |
| N(2) | 7831(4) | 897(2) | 3179(2) | 28(1) |
| C(1) | 5378(6) | -2750(3) | 5570(3) | 40(1) |
| C(2) | 5490(6) | -2913(3) | 6253(3) | 47(2) |
| C(3) | 6449(6) | -2519(4) | 6646(3) | 52(2) |
| C(4) | 7305(6) | -1948(3) | 6355(3) | 46(1) |
| C(5) | 7190(6) | -1785(3) | 5678(3) | 39(1) |
| C(6) | 6232(5) | -2187(3) | 5273(2) | 34(1) |
| C(7) | 6116(6) | -2005(3) | 4532(2) | 38(1) |
| C(8) | 6186(5) | -709(3) | 3982(2) | 29(1) |
| C(9) | 5037(5) | -115(3) | 3813(2) | 25(1) |
| C(10) | 5495(5) | 735(3) | 3641(2) | 25(1) |
| C(11) | 6440(5) | 663(3) | 3011(2) | 28(1) |
| C(12) | 9102(5) | 659(3) | 2861(2) | 35(1) |
| C(13) | 10728(7) | -661(4) | 2290(3) | 63(2) |
| C(14) | 10043(5) | -19(3) | 1884(2) | 39(1) |
| C(15) | 9273(6) | -350(4) | 1275(3) | 58(2) |
| C(16) | 11084(7) | 607(4) | 1664(3) | 56(2) |
| C(17) | 7836(5) | 1204(3) | 3854(2) | 28(1) |
| C(18) | 8919(5) | 1598(3) | 4183(3) | 36(1) |
| C(19) | 8619(5) | 1882(3) | 4824(2) | 34(1) |
| C(20) | 7308(5) | 1801(3) | 5118(2) | 31(1) |
| C(21) | 6231(5) | 1423(3) | 4770(2) | 29(1) |
| C(22) | 6499(5) | 1112(3) | 4137(2) | 24(1) |
| C(23) | 4127(5) | 1203(3) | 3563(2) | 25(1) |
| C(24) | 4162(5) | 1916(3) | 3148(2) | 29(1) |
| C(25) | 5312(5) | 2299(3) | 2882(2) | 35(1) |

| | | | | |
|---|---|---|---|---|
| C(26) | 4954(6) | 2952(3) | 2478(3) | 39(1) |
| C(27) | 3525(6) | 3065(3) | 2453(3) | 45(1) |
| C(28) | 2929(5) | 992(3) | 3877(2) | 29(1) |
| C(29) | 2653(5) | 256(3) | 4277(2) | 28(1) |
| C(30) | 1808(5) | 400(3) | 4916(2) | 30(1) |
| C(31) | 2067(5) | 1051(3) | 5317(2) | 35(1) |
| C(32) | 1329(6) | 1176(3) | 5914(3) | 43(1) |
| C(33) | 344(6) | 647(4) | 6132(3) | 51(2) |
| C(34) | 97(7) | -5(4) | 5752(3) | 64(2) |
| C(35) | 811(6) | -126(4) | 5144(3) | 54(2) |
| C(36) | 4056(5) | -144(3) | 4418(2) | 27(1) |
| C(37) | 4082(5) | -1019(3) | 4581(2) | 33(1) |

**Table S5**. Bond lengths [Å] and angles [°] for **3ka**.

| | |
|---|---|
| S(1)-C(24) | 1.723(5) |
| S(1)-C(27) | 1.699(6) |
| O(1)-C(8) | 1.225(5) |
| O(2)-H(2) | 0.8400 |
| O(2)-C(9) | 1.428(5) |
| O(3)-C(11) | 1.214(5) |
| O(4)-C(12) | 1.194(6) |
| O(5)-C(12) | 1.313(6) |
| O(5)-C(14) | 1.484(6) |
| N(1)-C(7) | 1.453(6) |
| N(1)-C(8) | 1.357(6) |
| N(1)-C(37) | 1.469(6) |
| N(2)-C(11) | 1.404(6) |
| N(2)-C(12) | 1.409(6) |
| N(2)-C(17) | 1.436(6) |
| C(1)-H(1) | 0.9500 |
| C(1)-C(2) | 1.384(7) |
| C(1)-C(6) | 1.385(7) |
| C(2)-H(2A) | 0.9500 |
| C(2)-C(3) | 1.367(8) |
| C(3)-H(3) | 0.9500 |
| C(3)-C(4) | 1.390(8) |
| C(4)-H(4) | 0.9500 |
| C(4)-C(5) | 1.373(7) |
| C(5)-H(5) | 0.9500 |
| C(5)-C(6) | 1.386(7) |
| C(6)-C(7) | 1.503(6) |
| C(7)-H(7A) | 0.9900 |
| C(7)-H(7B) | 0.9900 |
| C(8)-C(9) | 1.518(6) |
| C(9)-C(10) | 1.556(6) |
| C(9)-C(36) | 1.510(6) |
| C(10)-C(11) | 1.536(6) |
| C(10)-C(22) | 1.505(6) |
| C(10)-C(23) | 1.520(6) |
| C(13)-H(13A) | 0.9800 |

| | |
|---|---|
| C(13)-H(13B) | 0.9800 |
| C(13)-H(13C) | 0.9800 |
| C(13)-C(14) | 1.505(7) |
| C(14)-C(15) | 1.515(7) |
| C(14)-C(16) | 1.514(8) |
| C(15)-H(15A) | 0.9800 |
| C(15)-H(15B) | 0.9800 |
| C(15)-H(15C) | 0.9800 |
| C(16)-H(16A) | 0.9800 |
| C(16)-H(16B) | 0.9800 |
| C(16)-H(16C) | 0.9800 |
| C(17)-C(18) | 1.383(7) |
| C(17)-C(22) | 1.383(6) |
| C(18)-H(18) | 0.9500 |
| C(18)-C(19) | 1.388(7) |
| C(19)-H(19) | 0.9500 |
| C(19)-C(20) | 1.367(7) |
| C(20)-H(20) | 0.9500 |
| C(20)-C(21) | 1.384(6) |
| C(21)-H(21) | 0.9500 |
| C(21)-C(22) | 1.385(6) |
| C(23)-C(24) | 1.472(6) |
| C(23)-C(28) | 1.333(6) |
| C(24)-C(25) | 1.368(7) |
| C(25)-H(25) | 0.9500 |
| C(25)-C(26) | 1.415(7) |
| C(26)-H(26) | 0.9500 |
| C(26)-C(27) | 1.355(8) |
| C(27)-H(27) | 0.9500 |
| C(28)-H(28) | 0.9500 |
| C(28)-C(29) | 1.511(6) |
| C(29)-H(29) | 1.0000 |
| C(29)-C(30) | 1.513(6) |
| C(29)-C(36) | 1.510(6) |
| C(30)-C(31) | 1.390(7) |
| C(30)-C(35) | 1.376(7) |
| C(31)-H(31) | 0.9500 |
| C(31)-C(32) | 1.385(7) |

| | |
|---|---|
| C(32)-H(32) | 0.9500 |
| C(32)-C(33) | 1.364(8) |
| C(33)-H(33) | 0.9500 |
| C(33)-C(34) | 1.365(8) |
| C(34)-H(34) | 0.9500 |
| C(34)-C(35) | 1.393(8) |
| C(35)-H(35) | 0.9500 |
| C(36)-H(36) | 1.0000 |
| C(36)-C(37) | 1.532(6) |
| C(37)-H(37A) | 0.9900 |
| C(37)-H(37B) | 0.9900 |
| C(27)-S(1)-C(24) | 92.6(3) |
| C(9)-O(2)-H(2) | 109.5 |
| C(12)-O(5)-C(14) | 119.9(4) |
| C(7)-N(1)-C(37) | 121.5(4) |
| C(8)-N(1)-C(7) | 123.2(4) |
| C(8)-N(1)-C(37) | 113.4(4) |
| C(11)-N(2)-C(12) | 126.7(4) |
| C(11)-N(2)-C(17) | 109.1(4) |
| C(12)-N(2)-C(17) | 121.2(4) |
| C(2)-C(1)-H(1) | 119.7 |
| C(2)-C(1)-C(6) | 120.7(5) |
| C(6)-C(1)-H(1) | 119.7 |
| C(1)-C(2)-H(2A) | 119.8 |
| C(3)-C(2)-C(1) | 120.5(5) |
| C(3)-C(2)-H(2A) | 119.8 |
| C(2)-C(3)-H(3) | 120.3 |
| C(2)-C(3)-C(4) | 119.4(5) |
| C(4)-C(3)-H(3) | 120.3 |
| C(3)-C(4)-H(4) | 119.9 |
| C(5)-C(4)-C(3) | 120.2(5) |
| C(5)-C(4)-H(4) | 119.9 |
| C(4)-C(5)-H(5) | 119.5 |
| C(4)-C(5)-C(6) | 120.9(5) |
| C(6)-C(5)-H(5) | 119.5 |
| C(1)-C(6)-C(5) | 118.4(5) |
| C(1)-C(6)-C(7) | 121.1(5) |
| C(5)-C(6)-C(7) | 120.5(5) |

| | |
|---|---|
| N(1)-C(7)-C(6) | 112.5(4) |
| N(1)-C(7)-H(7A) | 109.1 |
| N(1)-C(7)-H(7B) | 109.1 |
| C(6)-C(7)-H(7A) | 109.1 |
| C(6)-C(7)-H(7B) | 109.1 |
| H(7A)-C(7)-H(7B) | 107.8 |
| O(1)-C(8)-N(1) | 126.6(4) |
| O(1)-C(8)-C(9) | 127.8(4) |
| N(1)-C(8)-C(9) | 105.4(4) |
| O(2)-C(9)-C(8) | 105.7(3) |
| O(2)-C(9)-C(10) | 109.5(3) |
| O(2)-C(9)-C(36) | 106.7(3) |
| C(8)-C(9)-C(10) | 118.6(4) |
| C(36)-C(9)-C(8) | 103.6(4) |
| C(36)-C(9)-C(10) | 111.8(4) |
| C(11)-C(10)-C(9) | 105.2(4) |
| C(22)-C(10)-C(9) | 115.5(4) |
| C(22)-C(10)-C(11) | 101.7(3) |
| C(22)-C(10)-C(23) | 111.7(4) |
| C(23)-C(10)-C(9) | 106.4(3) |
| C(23)-C(10)-C(11) | 116.5(4) |
| O(3)-C(11)-N(2) | 126.0(4) |
| O(3)-C(11)-C(10) | 125.2(4) |
| N(2)-C(11)-C(10) | 108.7(4) |
| O(4)-C(12)-O(5) | 127.0(4) |
| O(4)-C(12)-N(2) | 121.5(4) |
| O(5)-C(12)-N(2) | 111.5(4) |
| H(13A)-C(13)-H(13B) | 109.5 |
| H(13A)-C(13)-H(13C) | 109.5 |
| H(13B)-C(13)-H(13C) | 109.5 |
| C(14)-C(13)-H(13A) | 109.5 |
| C(14)-C(13)-H(13B) | 109.5 |
| C(14)-C(13)-H(13C) | 109.5 |
| O(5)-C(14)-C(13) | 109.5(4) |
| O(5)-C(14)-C(15) | 101.8(4) |
| O(5)-C(14)-C(16) | 110.7(4) |
| C(13)-C(14)-C(15) | 110.8(5) |
| C(13)-C(14)-C(16) | 113.3(5) |

| | |
|---|---|
| C(16)-C(14)-C(15) | 110.1(5) |
| C(14)-C(15)-H(15A) | 109.5 |
| C(14)-C(15)-H(15B) | 109.5 |
| C(14)-C(15)-H(15C) | 109.5 |
| H(15A)-C(15)-H(15B) | 109.5 |
| H(15A)-C(15)-H(15C) | 109.5 |
| H(15B)-C(15)-H(15C) | 109.5 |
| C(14)-C(16)-H(16A) | 109.5 |
| C(14)-C(16)-H(16B) | 109.5 |
| C(14)-C(16)-H(16C) | 109.5 |
| H(16A)-C(16)-H(16B) | 109.5 |
| H(16A)-C(16)-H(16C) | 109.5 |
| H(16B)-C(16)-H(16C) | 109.5 |
| C(18)-C(17)-N(2) | 128.3(4) |
| C(22)-C(17)-N(2) | 109.4(4) |
| C(22)-C(17)-C(18) | 122.1(4) |
| C(17)-C(18)-H(18) | 121.5 |
| C(17)-C(18)-C(19) | 117.0(5) |
| C(19)-C(18)-H(18) | 121.5 |
| C(18)-C(19)-H(19) | 118.8 |
| C(20)-C(19)-C(18) | 122.4(5) |
| C(20)-C(19)-H(19) | 118.8 |
| C(19)-C(20)-H(20) | 120.3 |
| C(19)-C(20)-C(21) | 119.5(4) |
| C(21)-C(20)-H(20) | 120.3 |
| C(20)-C(21)-H(21) | 120.1 |
| C(20)-C(21)-C(22) | 119.9(4) |
| C(22)-C(21)-H(21) | 120.1 |
| C(17)-C(22)-C(10) | 110.6(4) |
| C(17)-C(22)-C(21) | 119.2(4) |
| C(21)-C(22)-C(10) | 129.9(4) |
| C(24)-C(23)-C(10) | 118.4(4) |
| C(28)-C(23)-C(10) | 121.3(4) |
| C(28)-C(23)-C(24) | 120.2(4) |
| C(23)-C(24)-S(1) | 121.4(4) |
| C(25)-C(24)-S(1) | 109.4(3) |
| C(25)-C(24)-C(23) | 129.1(4) |
| C(24)-C(25)-H(25) | 122.9 |

| | |
|---|---|
| C(24)-C(25)-C(26) | 114.2(5) |
| C(26)-C(25)-H(25) | 122.9 |
| C(25)-C(26)-H(26) | 124.2 |
| C(27)-C(26)-C(25) | 111.6(5) |
| C(27)-C(26)-H(26) | 124.2 |
| S(1)-C(27)-H(27) | 123.9 |
| C(26)-C(27)-S(1) | 112.2(4) |
| C(26)-C(27)-H(27) | 123.9 |
| C(23)-C(28)-H(28) | 116.0 |
| C(23)-C(28)-C(29) | 127.9(4) |
| C(29)-C(28)-H(28) | 116.0 |
| C(28)-C(29)-H(29) | 107.5 |
| C(28)-C(29)-C(30) | 113.1(4) |
| C(30)-C(29)-H(29) | 107.5 |
| C(36)-C(29)-C(28) | 109.0(4) |
| C(36)-C(29)-H(29) | 107.5 |
| C(36)-C(29)-C(30) | 112.1(4) |
| C(31)-C(30)-C(29) | 121.2(4) |
| C(35)-C(30)-C(29) | 121.6(5) |
| C(35)-C(30)-C(31) | 117.1(5) |
| C(30)-C(31)-H(31) | 119.2 |
| C(32)-C(31)-C(30) | 121.6(5) |
| C(32)-C(31)-H(31) | 119.2 |
| C(31)-C(32)-H(32) | 119.8 |
| C(33)-C(32)-C(31) | 120.3(5) |
| C(33)-C(32)-H(32) | 119.8 |
| C(32)-C(33)-H(33) | 120.5 |
| C(32)-C(33)-C(34) | 119.0(5) |
| C(34)-C(33)-H(33) | 120.5 |
| C(33)-C(34)-H(34) | 119.5 |
| C(33)-C(34)-C(35) | 121.1(6) |
| C(35)-C(34)-H(34) | 119.5 |
| C(30)-C(35)-C(34) | 120.8(6) |
| C(30)-C(35)-H(35) | 119.6 |
| C(34)-C(35)-H(35) | 119.6 |
| C(9)-C(36)-C(29) | 111.7(4) |
| C(9)-C(36)-H(36) | 108.0 |
| C(9)-C(36)-C(37) | 100.9(4) |

| | |
|---|---|
| C(29)-C(36)-H(36) | 108.0 |
| C(29)-C(36)-C(37) | 119.7(4) |
| C(37)-C(36)-H(36) | 108.0 |
| N(1)-C(37)-C(36) | 101.6(4) |
| N(1)-C(37)-H(37A) | 111.4 |
| N(1)-C(37)-H(37B) | 111.4 |
| C(36)-C(37)-H(37A) | 111.4 |
| C(36)-C(37)-H(37B) | 111.4 |
| H(37A)-C(37)-H(37B) | 109.3 |

Symmetry transformations used to generate equivalent atoms:

**Table S6**. Anisotropic displacement parameters ($Å^2$x $10^3$) for **3ka**. The anisotropic displacement factor exponent takes the form: $-2p^2[ h^2 a^{*2}U^{11} + ... + 2 h k a^* b^* U^{12} ]$

| | $U^{11}$ | $U^{22}$ | $U^{33}$ | $U^{23}$ | $U^{13}$ | $U^{12}$ |
|---|---|---|---|---|---|---|
| S(1) | 41(1) | 42(1) | 51(1) | 15(1) | -3(1) | 6(1) |
| O(1) | 30(2) | 33(2) | 38(2) | -5(2) | 0(2) | 2(2) |
| O(2) | 32(2) | 30(2) | 26(2) | -5(2) | -4(2) | -6(2) |
| O(3) | 32(2) | 38(2) | 28(2) | -5(2) | -1(2) | 0(2) |
| O(4) | 28(2) | 108(4) | 60(3) | -40(3) | -2(2) | 1(2) |
| O(5) | 33(2) | 48(2) | 27(2) | -7(2) | 3(2) | 3(2) |
| N(1) | 33(2) | 21(2) | 36(2) | 4(2) | 4(2) | 6(2) |
| N(2) | 24(2) | 37(3) | 24(2) | -6(2) | 1(2) | 1(2) |
| C(1) | 39(3) | 35(3) | 46(3) | 3(3) | -4(3) | -1(2) |
| C(2) | 49(3) | 46(4) | 47(3) | 16(3) | -3(3) | -9(3) |
| C(3) | 59(4) | 57(4) | 40(3) | 11(3) | -4(3) | 1(3) |
| C(4) | 48(3) | 46(3) | 46(3) | -7(3) | -5(3) | -2(3) |
| C(5) | 46(3) | 35(3) | 36(3) | -3(2) | -1(3) | 1(3) |
| C(6) | 32(3) | 28(3) | 41(3) | 3(2) | -3(2) | 5(2) |
| C(7) | 52(3) | 23(3) | 37(3) | 1(2) | -4(3) | 6(3) |
| C(8) | 31(3) | 28(3) | 29(3) | -4(2) | -3(2) | 0(2) |
| C(9) | 29(3) | 22(3) | 24(2) | -1(2) | -4(2) | -1(2) |
| C(10) | 24(2) | 29(3) | 23(2) | 1(2) | -2(2) | 0(2) |
| C(11) | 29(3) | 28(3) | 27(3) | 0(2) | -4(2) | -1(2) |
| C(12) | 27(3) | 44(3) | 33(3) | -5(3) | 4(2) | 4(2) |
| C(13) | 73(4) | 62(4) | 55(4) | 10(3) | 14(3) | 31(4) |
| C(14) | 37(3) | 43(3) | 37(3) | -7(3) | 11(2) | 8(3) |
| C(15) | 53(4) | 78(5) | 43(3) | -22(3) | 2(3) | 6(3) |
| C(16) | 53(4) | 55(4) | 60(4) | -5(3) | 26(3) | -4(3) |
| C(17) | 29(3) | 29(3) | 27(2) | -4(2) | -3(2) | 1(2) |
| C(18) | 29(3) | 35(3) | 45(3) | -5(2) | -3(2) | -1(3) |
| C(19) | 34(3) | 28(3) | 40(3) | -9(2) | -5(2) | 0(2) |
| C(20) | 35(3) | 32(3) | 26(2) | -6(2) | -5(2) | -1(2) |
| C(21) | 30(3) | 27(3) | 30(3) | 0(2) | 1(2) | 1(2) |
| C(22) | 24(2) | 23(3) | 25(2) | 1(2) | -1(2) | 0(2) |
| C(23) | 27(2) | 27(3) | 21(2) | 1(2) | 2(2) | 1(2) |
| C(24) | 33(3) | 26(3) | 27(2) | 3(2) | 1(2) | -1(2) |
| C(25) | 41(3) | 28(3) | 37(3) | 8(2) | 3(2) | 4(2) |

| | | | | | | |
|---|---|---|---|---|---|---|
| C(26) | 49(3) | 27(3) | 42(3) | 10(2) | 10(3) | -1(3) |
| C(27) | 57(4) | 40(3) | 37(3) | 11(3) | 0(3) | 8(3) |
| C(28) | 32(3) | 25(3) | 30(3) | 6(2) | -4(2) | 2(2) |
| C(29) | 27(2) | 29(3) | 28(2) | 1(2) | 2(2) | -3(2) |
| C(30) | 27(2) | 28(3) | 34(3) | 2(2) | 4(2) | -1(2) |
| C(31) | 33(3) | 37(3) | 37(3) | 1(2) | 6(2) | 0(2) |
| C(32) | 44(3) | 42(3) | 42(3) | -4(3) | 3(3) | 10(3) |
| C(33) | 54(4) | 56(4) | 44(3) | 1(3) | 14(3) | 8(3) |
| C(34) | 68(4) | 58(4) | 66(4) | -3(4) | 28(4) | -23(4) |
| C(35) | 59(4) | 43(4) | 58(4) | -12(3) | 22(3) | -15(3) |
| C(36) | 28(2) | 25(3) | 27(2) | -1(2) | 2(2) | 2(2) |
| C(37) | 37(3) | 25(3) | 37(3) | -1(2) | 5(2) | 0(2) |

**Table S7**. Hydrogen coordinates (x $10^4$) and isotropic displacement parameters ($Å^2$x $10^3$) for **3ka**.

| | x | y | z | U(eq) |
|---|---|---|---|---|
| H(2) | 4668 | -315 | 2894 | 44 |
| H(1) | 4708 | -3028 | 5302 | 48 |
| H(2A) | 4896 | -3300 | 6450 | 57 |
| H(3) | 6531 | -2635 | 7114 | 62 |
| H(4) | 7971 | -1670 | 6625 | 56 |
| H(5) | 7774 | -1391 | 5484 | 47 |
| H(7A) | 7072 | -2050 | 4323 | 45 |
| H(7B) | 5485 | -2394 | 4315 | 45 |
| H(13A) | 11237 | -433 | 2675 | 95 |
| H(13B) | 11403 | -947 | 2004 | 95 |
| H(13C) | 9991 | -1019 | 2454 | 95 |
| H(15A) | 8576 | -740 | 1424 | 87 |
| H(15B) | 9965 | -596 | 971 | 87 |
| H(15C) | 8780 | 73 | 1036 | 87 |
| H(16A) | 10561 | 1041 | 1457 | 84 |
| H(16B) | 11755 | 388 | 1336 | 84 |
| H(16C) | 11611 | 798 | 2059 | 84 |
| H(18) | 9827 | 1670 | 3980 | 43 |
| H(19) | 9352 | 2143 | 5068 | 40 |
| H(20) | 7139 | 2003 | 5558 | 37 |
| H(21) | 5309 | 1378 | 4965 | 34 |
| H(25) | 6268 | 2140 | 2961 | 42 |
| H(26) | 5632 | 3272 | 2253 | 47 |
| H(27) | 3082 | 3482 | 2217 | 54 |
| H(28) | 2156 | 1348 | 3845 | 35 |
| H(29) | 2077 | -101 | 3985 | 33 |
| H(31) | 2768 | 1418 | 5180 | 42 |
| H(32) | 1509 | 1633 | 6172 | 52 |
| H(33) | -162 | 731 | 6541 | 62 |
| H(34) | -572 | -382 | 5906 | 77 |
| H(35) | 606 | -579 | 4884 | 64 |
| H(36) | 4532 | 139 | 4798 | 32 |

| | | | | |
|---|---|---|---|---|
| H(37A) | 3880 | -1117 | 5064 | 39 |
| H(37B) | 3388 | -1311 | 4301 | 39 |

**Table S8**. Torsion angles [°] for**3ka**.

| | |
|---|---|
| S(1)-C(24)-C(25)-C(26) | 0.1(5) |
| O(1)-C(8)-C(9)-O(2) | 89.8(5) |
| O(1)-C(8)-C(9)-C(10) | -33.4(7) |
| O(1)-C(8)-C(9)-C(36) | -158.1(4) |
| O(2)-C(9)-C(10)-C(11) | -60.3(4) |
| O(2)-C(9)-C(10)-C(22) | -171.6(3) |
| O(2)-C(9)-C(10)-C(23) | 63.9(4) |
| O(2)-C(9)-C(36)-C(29) | -54.6(5) |
| O(2)-C(9)-C(36)-C(37) | 73.8(4) |
| N(1)-C(8)-C(9)-O(2) | -85.8(4) |
| N(1)-C(8)-C(9)-C(10) | 151.0(4) |
| N(1)-C(8)-C(9)-C(36) | 26.3(4) |
| N(2)-C(17)-C(18)-C(19) | 175.7(5) |
| N(2)-C(17)-C(22)-C(10) | -0.9(5) |
| N(2)-C(17)-C(22)-C(21) | -174.9(4) |
| C(1)-C(2)-C(3)-C(4) | 0.6(9) |
| C(1)-C(6)-C(7)-N(1) | 112.6(5) |
| C(2)-C(1)-C(6)-C(5) | -0.6(8) |
| C(2)-C(1)-C(6)-C(7) | -179.9(5) |
| C(2)-C(3)-C(4)-C(5) | -0.3(9) |
| C(3)-C(4)-C(5)-C(6) | -0.4(8) |
| C(4)-C(5)-C(6)-C(1) | 0.9(8) |
| C(4)-C(5)-C(6)-C(7) | -179.9(5) |
| C(5)-C(6)-C(7)-N(1) | -66.6(6) |
| C(6)-C(1)-C(2)-C(3) | -0.1(8) |
| C(7)-N(1)-C(8)-O(1) | -14.7(7) |
| C(7)-N(1)-C(8)-C(9) | 160.9(4) |
| C(7)-N(1)-C(37)-C(36) | 175.0(4) |
| C(8)-N(1)-C(7)-C(6) | 130.2(5) |
| C(8)-N(1)-C(37)-C(36) | -20.5(5) |
| C(8)-C(9)-C(10)-C(11) | 61.1(5) |
| C(8)-C(9)-C(10)-C(22) | -50.2(5) |
| C(8)-C(9)-C(10)-C(23) | -174.7(4) |
| C(8)-C(9)-C(36)-C(29) | -165.9(4) |
| C(8)-C(9)-C(36)-C(37) | -37.6(4) |
| C(9)-C(10)-C(11)-O(3) | 62.6(6) |

| | |
|---|---|
| C(9)-C(10)-C(11)-N(2) | -115.1(4) |
| C(9)-C(10)-C(22)-C(17) | 110.4(4) |
| C(9)-C(10)-C(22)-C(21) | -76.4(6) |
| C(9)-C(10)-C(23)-C(24) | -155.9(4) |
| C(9)-C(10)-C(23)-C(28) | 26.2(6) |
| C(9)-C(36)-C(37)-N(1) | 34.8(4) |
| C(10)-C(9)-C(36)-C(29) | 65.2(5) |
| C(10)-C(9)-C(36)-C(37) | -166.5(4) |
| C(10)-C(23)-C(24)-S(1) | 166.9(3) |
| C(10)-C(23)-C(24)-C(25) | -9.8(7) |
| C(10)-C(23)-C(28)-C(29) | -7.3(8) |
| C(11)-N(2)-C(12)-O(4) | -165.2(5) |
| C(11)-N(2)-C(12)-O(5) | 15.1(7) |
| C(11)-N(2)-C(17)-C(18) | -169.9(5) |
| C(11)-N(2)-C(17)-C(22) | 4.7(5) |
| C(11)-C(10)-C(22)-C(17) | -2.9(5) |
| C(11)-C(10)-C(22)-C(21) | 170.4(5) |
| C(11)-C(10)-C(23)-C(24) | -39.1(6) |
| C(11)-C(10)-C(23)-C(28) | 143.1(5) |
| C(12)-O(5)-C(14)-C(13) | 60.9(6) |
| C(12)-O(5)-C(14)-C(15) | 178.2(5) |
| C(12)-O(5)-C(14)-C(16) | -64.7(6) |
| C(12)-N(2)-C(11)-O(3) | -23.6(8) |
| C(12)-N(2)-C(11)-C(10) | 154.1(4) |
| C(12)-N(2)-C(17)-C(18) | 28.1(7) |
| C(12)-N(2)-C(17)-C(22) | -157.2(4) |
| C(14)-O(5)-C(12)-O(4) | 4.9(8) |
| C(14)-O(5)-C(12)-N(2) | -175.4(4) |
| C(17)-N(2)-C(11)-O(3) | 175.7(5) |
| C(17)-N(2)-C(11)-C(10) | -6.6(5) |
| C(17)-N(2)-C(12)-O(4) | -6.6(8) |
| C(17)-N(2)-C(12)-O(5) | 173.6(4) |
| C(17)-C(18)-C(19)-C(20) | -1.7(7) |
| C(18)-C(17)-C(22)-C(10) | 174.2(4) |
| C(18)-C(17)-C(22)-C(21) | 0.1(7) |
| C(18)-C(19)-C(20)-C(21) | 0.0(7) |
| C(19)-C(20)-C(21)-C(22) | 1.9(7) |
| C(20)-C(21)-C(22)-C(10) | -174.6(5) |

| | |
|---|---|
| C(20)-C(21)-C(22)-C(17) | -1.9(7) |
| C(22)-C(10)-C(11)-O(3) | -176.6(5) |
| C(22)-C(10)-C(11)-N(2) | 5.7(5) |
| C(22)-C(10)-C(23)-C(24) | 77.2(5) |
| C(22)-C(10)-C(23)-C(28) | -100.6(5) |
| C(22)-C(17)-C(18)-C(19) | 1.6(7) |
| C(23)-C(10)-C(11)-O(3) | -54.9(7) |
| C(23)-C(10)-C(11)-N(2) | 127.4(4) |
| C(23)-C(10)-C(22)-C(17) | -127.8(4) |
| C(23)-C(10)-C(22)-C(21) | 45.4(6) |
| C(23)-C(24)-C(25)-C(26) | 177.2(5) |
| C(23)-C(28)-C(29)-C(30) | 138.8(5) |
| C(23)-C(28)-C(29)-C(36) | 13.4(7) |
| C(24)-S(1)-C(27)-C(26) | 1.5(5) |
| C(24)-C(23)-C(28)-C(29) | 174.9(4) |
| C(24)-C(25)-C(26)-C(27) | 1.0(7) |
| C(25)-C(26)-C(27)-S(1) | -1.7(6) |
| C(27)-S(1)-C(24)-C(23) | -178.2(4) |
| C(27)-S(1)-C(24)-C(25) | -0.9(4) |
| C(28)-C(23)-C(24)-S(1) | -15.2(6) |
| C(28)-C(23)-C(24)-C(25) | 168.0(5) |
| C(28)-C(29)-C(30)-C(31) | -40.1(6) |
| C(28)-C(29)-C(30)-C(35) | 143.6(5) |
| C(28)-C(29)-C(36)-C(9) | -40.6(5) |
| C(28)-C(29)-C(36)-C(37) | -158.1(4) |
| C(29)-C(30)-C(31)-C(32) | -178.1(4) |
| C(29)-C(30)-C(35)-C(34) | 176.5(5) |
| C(29)-C(36)-C(37)-N(1) | 157.8(4) |
| C(30)-C(29)-C(36)-C(9) | -166.5(4) |
| C(30)-C(29)-C(36)-C(37) | 76.0(5) |
| C(30)-C(31)-C(32)-C(33) | 1.7(8) |
| C(31)-C(30)-C(35)-C(34) | 0.1(9) |
| C(31)-C(32)-C(33)-C(34) | -0.2(8) |
| C(32)-C(33)-C(34)-C(35) | -1.4(10) |
| C(33)-C(34)-C(35)-C(30) | 1.4(10) |
| C(35)-C(30)-C(31)-C(32) | -1.6(7) |
| C(36)-C(9)-C(10)-C(11) | -178.4(3) |
| C(36)-C(9)-C(10)-C(22) | 70.3(5) |

| | |
|---|---|
| C(36)-C(9)-C(10)-C(23) | -54.2(4) |
| C(36)-C(29)-C(30)-C(31) | 83.6(6) |
| C(36)-C(29)-C(30)-C(35) | -92.7(6) |
| C(37)-N(1)-C(7)-C(6) | -66.8(6) |
| C(37)-N(1)-C(8)-O(1) | -179.0(4) |
| C(37)-N(1)-C(8)-C(9) | -3.3(5) |

Symmetry transformations used to generate equivalent atoms:

**CCDC 2327249 (5bf)**

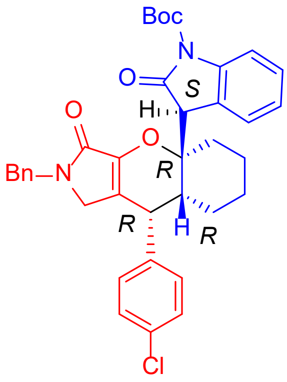


**5bf**

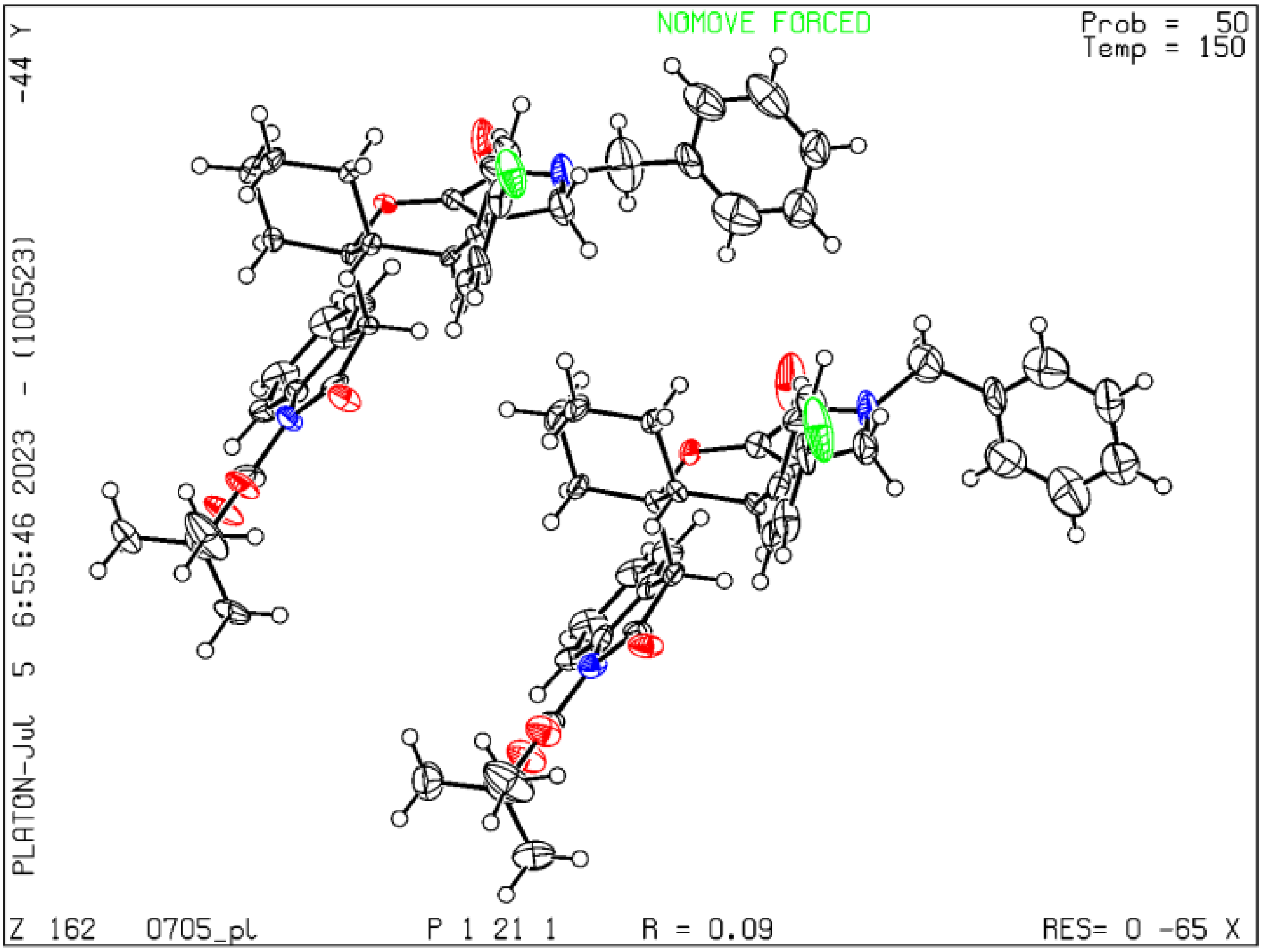


ORTEP drawing of **5bf** showing thermal ellipsoids at the 50% probability level

The crystal was obtained by slow evaporation of **4r** in Hexane : DCM = 3:1 at room temperature.

**Table S9**. Crystal data and structure refinement for **5bf**.

| | | |
|---|---|---|
| Identification code | 1226 | |
| Empirical formula | C74 H74 Cl2 N4 O10 | |
| Formula weight | 1250.27 | |
| Temperature | 150.15 K | |
| Wavelength | 0.71076 Å | |
| Crystal system | Monoclinic | |
| Space group | P 1 21 1 | |
| Unit cell dimensions | a = 11.4388(11) Å | a= 90°. |
| | b = 19.614(2) Å | b= 110.612(4)°. |
| | c = 14.9240(15) Å | g = 90°. |
| Volume | 3134.0(5) Å3 | |
| Z | 2 | |
| Density (calculated) | 1.325 Mg/m3 | |
| Absorption coefficient | 0.170 mm-1 | |
| F(000) | 1320 | |
| Crystal size | | |
| Theta range for data collection | 2.538 to 24.500°. | |
| Index ranges | -13<=h<=13, -22<=k<=22, -17<=l<=17 | |
| Reflections collected | 77631 | |
| Independent reflections | 10152 [R(int) = 0.0633] | |
| Completeness to theta = 24.500° | 97.4 % | |
| Absorption correction | None | |
| Refinement method | Full-matrix least-squares on F2 | |
| Data / restraints / parameters | 10152 / 2020 / 754 | |
| Goodness-of-fit on F2 | 1.083 | |
| Final R indices [I>2sigma(I)] | R1 = 0.0874, wR2 = 0.1585 | |
| R indices (all data) | R1 = 0.1067, wR2 = 0.1696 | |
| Absolute structure (Flack) parameter | 0.02(15) | |
| Extinction coefficient | n/a | |
| Largest diff. peak and hole | 0.955 and -0.615 e.Å-3 | |

**Table S10**. Atomic coordinates (x $10^4$) and equivalent isotropic displacement parameters ($Å^2$x $10^3$) for **5bf**. U(eq) is defined as one third of the trace of the orthogonalized $U^{ij}$ tensor.

| | x | y | z | U(eq) |
|---|---|---|---|---|
| Cl(1) | -3683(3) | 3530(2) | 6332(3) | 81(1) |
| O(1) | 3389(5) | 4434(3) | 3780(5) | 40(2) |
| O(2) | 4725(6) | 3937(3) | 3173(5) | 46(2) |
| O(3) | 1697(5) | 3610(3) | 4026(4) | 36(1) |
| O(4) | 2403(4) | 1530(3) | 5306(3) | 24(1) |
| O(5) | 1827(7) | 92(4) | 4929(8) | 97(4) |
| N(1) | 3522(6) | 3293(3) | 3778(4) | 26(1) |
| N(2) | -242(7) | 449(4) | 4389(7) | 60(3) |
| C(1) | 3950(7) | 3921(4) | 3541(6) | 31(2) |
| C(2) | 3695(8) | 5141(5) | 3573(7) | 43(2) |
| C(3) | 2841(10) | 5547(6) | 3921(10) | 66(3) |
| C(4) | 3335(11) | 5228(6) | 2513(8) | 73(4) |
| C(5) | 5067(9) | 5279(6) | 4128(8) | 51(2) |
| C(11) | 3408(4) | 2135(2) | 3921(4) | 26(2) |
| C(10) | 3781(5) | 1464(2) | 3886(4) | 33(2) |
| C(9) | 4829(5) | 1324(2) | 3651(4) | 40(2) |
| C(8) | 5505(4) | 1855(3) | 3451(4) | 39(2) |
| C(7) | 5132(4) | 2526(2) | 3486(4) | 30(2) |
| C(6) | 4084(4) | 2666(2) | 3721(4) | 31(2) |
| C(12) | 2352(7) | 2418(4) | 4180(5) | 26(1) |
| C(13) | 2439(7) | 3168(4) | 3996(5) | 26(2) |
| C(14) | 2436(6) | 2269(4) | 5223(5) | 26(1) |
| C(15) | 3727(6) | 2483(4) | 5928(5) | 28(2) |
| C(16) | 3885(7) | 2329(5) | 6969(5) | 34(2) |
| C(17) | 2859(7) | 2680(5) | 7224(5) | 32(2) |
| C(18) | 1571(7) | 2456(4) | 6534(5) | 27(2) |
| C(19) | 1383(6) | 2591(4) | 5482(5) | 26(1) |
| C(20) | 73(6) | 2364(4) | 4788(5) | 26(1) |
| C(21) | -978(4) | 2616(2) | 5109(4) | 31(2) |
| C(22) | -1456(5) | 3263(2) | 4814(3) | 34(2) |
| C(23) | -2314(5) | 3550(2) | 5170(4) | 42(2) |
| C(24) | -2696(5) | 3190(3) | 5822(4) | 39(2) |
| C(25) | -2218(5) | 2543(3) | 6117(4) | 45(2) |

| | | | | |
|---|---|---|---|---|
| C(26) | -1359(5) | 2256(2) | 5760(4) | 36(2) |
| C(27) | -886(8) | 1091(5) | 4295(7) | 45(2) |
| C(28) | 134(7) | 1604(4) | 4682(6) | 28(2) |
| C(29) | 1216(7) | 1277(4) | 4935(6) | 31(2) |
| C(30) | 1030(9) | 538(5) | 4773(7) | 49(2) |
| C(31) | -941(12) | -187(7) | 4419(10) | 48(2) |
| C(32) | -2078(5) | -277(3) | 3556(4) | 49(2) |
| C(33) | -1999(5) | -182(4) | 2656(5) | 63(2) |
| C(34) | -3064(7) | -238(4) | 1841(4) | 69(2) |
| C(35) | -4208(5) | -389(4) | 1927(4) | 63(2) |
| C(36) | -4286(5) | -483(4) | 2827(5) | 54(2) |
| C(37) | -3221(6) | -427(4) | 3642(4) | 61(2) |
| Cl(2) | 1423(2) | 3413(1) | 11421(2) | 55(1) |
| O(6) | 8369(5) | 4360(3) | 8724(5) | 36(1) |
| O(7) | 9720(6) | 3873(3) | 8116(5) | 44(2) |
| O(8) | 6692(5) | 3526(3) | 8975(4) | 30(1) |
| O(9) | 7452(4) | 1461(2) | 10304(3) | 24(1) |
| O(10) | 6930(6) | 45(3) | 9743(8) | 83(3) |
| N(3) | 8541(6) | 3218(3) | 8740(5) | 28(2) |
| N(4) | 4874(7) | 394(4) | 9211(7) | 58(3) |
| C(38) | 8943(7) | 3845(4) | 8480(6) | 33(2) |
| C(39) | 8674(8) | 5061(4) | 8497(7) | 42(2) |
| C(40) | 7837(10) | 5487(5) | 8878(10) | 65(3) |
| C(41) | 8359(10) | 5141(5) | 7424(8) | 63(3) |
| C(42) | 10032(8) | 5222(5) | 9068(7) | 43(2) |
| C(48) | 8476(4) | 2064(2) | 8921(4) | 24(1) |
| C(47) | 8879(4) | 1395(2) | 8922(4) | 28(2) |
| C(46) | 9939(5) | 1257(2) | 8702(4) | 37(2) |
| C(45) | 10598(4) | 1787(3) | 8482(4) | 34(2) |
| C(44) | 10195(4) | 2456(2) | 8482(4) | 29(2) |
| C(43) | 9135(4) | 2594(2) | 8701(4) | 24(1) |
| C(49) | 7414(6) | 2340(4) | 9171(5) | 21(1) |
| C(50) | 7444(7) | 3107(4) | 8958(5) | 25(2) |
| C(51) | 7498(6) | 2206(3) | 10213(5) | 20(1) |
| C(52) | 8762(6) | 2422(4) | 10919(5) | 24(1) |
| C(53) | 8942(7) | 2292(4) | 11962(5) | 31(2) |
| C(54) | 7870(6) | 2630(4) | 12207(5) | 30(2) |
| C(55) | 6607(6) | 2377(4) | 11519(5) | 26(1) |

| | | | | |
|---|---|---|---|---|
| C(56) | 6429(6) | 2532(4) | 10469(5) | 23(1) |
| C(57) | 5118(6) | 2286(4) | 9772(5) | 23(1) |
| C(59) | 3598(4) | 3177(2) | 9859(3) | 29(2) |
| C(60) | 2763(4) | 3444(2) | 10253(4) | 32(2) |
| C(61) | 2396(4) | 3056(3) | 10888(4) | 31(2) |
| C(62) | 2864(5) | 2401(3) | 11128(4) | 33(2) |
| C(63) | 3700(5) | 2134(2) | 10734(4) | 31(2) |
| C(58) | 4067(4) | 2522(2) | 10099(3) | 25(1) |
| C(64) | 4193(8) | 1029(4) | 9159(7) | 42(2) |
| C(65) | 5212(7) | 1535(4) | 9623(5) | 26(1) |
| C(66) | 6284(7) | 1209(4) | 9888(5) | 25(1) |
| C(67) | 6131(8) | 478(4) | 9611(7) | 41(2) |
| C(69) | 3028(4) | -322(3) | 8378(4) | 39(2) |
| C(70) | 2276(6) | -260(4) | 7420(4) | 63(2) |
| C(71) | 998(5) | -371(4) | 7140(4) | 61(2) |
| C(72) | 470(4) | -545(3) | 7817(5) | 52(2) |
| C(73) | 1221(6) | -607(3) | 8775(4) | 55(2) |
| C(74) | 2500(5) | -496(3) | 9056(3) | 54(2) |
| C(75) | 4426(12) | -253(7) | 8636(10) | 47(2) |
| C(75A) | 4260(30) | -258(17) | 9390(30) | 47(2) |
| C(31A) | -550(30) | -180(20) | 3650(30) | 48(2) |

**Table S11**. Bond lengths [Å] and angles [°] for **5bf**.

| | |
|---|---|
| Cl(1)-C(24) | 1.706(4) |
| O(1)-C(1) | 1.309(10) |
| O(1)-C(2) | 1.490(10) |
| O(2)-C(1) | 1.196(10) |
| O(3)-C(13) | 1.225(9) |
| O(4)-C(14) | 1.457(9) |
| O(4)-C(29) | 1.365(9) |
| O(5)-C(30) | 1.226(12) |
| N(1)-C(1) | 1.415(10) |
| N(1)-C(6) | 1.404(7) |
| N(1)-C(13) | 1.409(9) |
| N(2)-C(27) | 1.439(12) |
| N(2)-C(30) | 1.374(12) |
| N(2)-C(31) | 1.492(14) |
| N(2)-C(31A) | 1.60(4) |
| C(2)-C(3) | 1.489(14) |
| C(2)-C(4) | 1.498(13) |
| C(2)-C(5) | 1.519(12) |
| C(3)-H(3A) | 0.9800 |
| C(3)-H(3B) | 0.9800 |
| C(3)-H(3C) | 0.9800 |
| C(4)-H(4A) | 0.9800 |
| C(4)-H(4B) | 0.9800 |
| C(4)-H(4C) | 0.9800 |
| C(5)-H(5A) | 0.9800 |
| C(5)-H(5B) | 0.9800 |
| C(5)-H(5C) | 0.9800 |
| C(11)-C(10) | 1.3900 |
| C(11)-C(6) | 1.3900 |
| C(11)-C(12) | 1.499(8) |
| C(10)-H(10) | 0.9500 |
| C(10)-C(9) | 1.3900 |
| C(9)-H(9) | 0.9500 |
| C(9)-C(8) | 1.3900 |
| C(8)-H(8) | 0.9500 |
| C(8)-C(7) | 1.3900 |

| | |
|---|---|
| C(7)-H(7) | 0.9500 |
| C(7)-C(6) | 1.3900 |
| C(12)-H(12) | 1.0000 |
| C(12)-C(13) | 1.506(11) |
| C(12)-C(14) | 1.554(9) |
| C(14)-C(15) | 1.539(9) |
| C(14)-C(19) | 1.526(9) |
| C(15)-H(15A) | 0.9900 |
| C(15)-H(15B) | 0.9900 |
| C(15)-C(16) | 1.530(9) |
| C(16)-H(16A) | 0.9900 |
| C(16)-H(16B) | 0.9900 |
| C(16)-C(17) | 1.520(10) |
| C(17)-H(17A) | 0.9900 |
| C(17)-H(17B) | 0.9900 |
| C(17)-C(18) | 1.534(9) |
| C(18)-H(18A) | 0.9900 |
| C(18)-H(18B) | 0.9900 |
| C(18)-C(19) | 1.530(9) |
| C(19)-H(19) | 1.0000 |
| C(19)-C(20) | 1.556(9) |
| C(20)-H(20) | 1.0000 |
| C(20)-C(21) | 1.525(8) |
| C(20)-C(28) | 1.502(11) |
| C(21)-C(22) | 1.3900 |
| C(21)-C(26) | 1.3900 |
| C(22)-H(22) | 0.9500 |
| C(22)-C(23) | 1.3900 |
| C(23)-H(23) | 0.9500 |
| C(23)-C(24) | 1.3900 |
| C(24)-C(25) | 1.3900 |
| C(25)-H(25) | 0.9500 |
| C(25)-C(26) | 1.3900 |
| C(26)-H(26) | 0.9500 |
| C(27)-H(27A) | 0.9900 |
| C(27)-H(27B) | 0.9900 |
| C(27)-C(28) | 1.496(11) |
| C(28)-C(29) | 1.326(11) |

| | |
|---|---|
| C(29)-C(30) | 1.472(13) |
| C(31)-H(31A) | 0.9900 |
| C(31)-H(31B) | 0.9900 |
| C(31)-C(32) | 1.483(13) |
| C(32)-C(33) | 1.3900 |
| C(32)-C(37) | 1.3900 |
| C(32)-C(31A) | 1.72(3) |
| C(33)-H(33) | 0.9500 |
| C(33)-C(34) | 1.3900 |
| C(34)-H(34) | 0.9500 |
| C(34)-C(35) | 1.3900 |
| C(35)-H(35) | 0.9500 |
| C(35)-C(36) | 1.3900 |
| C(36)-H(36) | 0.9500 |
| C(36)-C(37) | 1.3900 |
| C(37)-H(37) | 0.9500 |
| Cl(2)-C(61) | 1.728(4) |
| O(6)-C(38) | 1.323(10) |
| O(6)-C(39) | 1.487(10) |
| O(7)-C(38) | 1.195(10) |
| O(8)-C(50) | 1.196(9) |
| O(9)-C(51) | 1.469(8) |
| O(9)-C(66) | 1.353(8) |
| O(10)-C(67) | 1.211(11) |
| N(3)-C(38) | 1.414(10) |
| N(3)-C(43) | 1.411(7) |
| N(3)-C(50) | 1.420(10) |
| N(4)-C(64) | 1.456(12) |
| N(4)-C(67) | 1.359(11) |
| N(4)-C(75) | 1.517(15) |
| N(4)-C(75A) | 1.53(3) |
| C(39)-C(40) | 1.522(13) |
| C(39)-C(41) | 1.521(14) |
| C(39)-C(42) | 1.519(12) |
| C(40)-H(40A) | 0.9800 |
| C(40)-H(40B) | 0.9800 |
| C(40)-H(40C) | 0.9800 |
| C(41)-H(41A) | 0.9800 |

| | |
|---|---|
| C(41)-H(41B) | 0.9800 |
| C(41)-H(41C) | 0.9800 |
| C(42)-H(42A) | 0.9800 |
| C(42)-H(42B) | 0.9800 |
| C(42)-H(42C) | 0.9800 |
| C(48)-C(47) | 1.3900 |
| C(48)-C(43) | 1.3900 |
| C(48)-C(49) | 1.493(8) |
| C(47)-H(47) | 0.9500 |
| C(47)-C(46) | 1.3900 |
| C(46)-H(46) | 0.9500 |
| C(46)-C(45) | 1.3900 |
| C(45)-H(45) | 0.9500 |
| C(45)-C(44) | 1.3900 |
| C(44)-H(44) | 0.9500 |
| C(44)-C(43) | 1.3900 |
| C(49)-H(49) | 1.0000 |
| C(49)-C(50) | 1.540(10) |
| C(49)-C(51) | 1.547(9) |
| C(51)-C(52) | 1.520(8) |
| C(51)-C(56) | 1.542(9) |
| C(52)-H(52A) | 0.9900 |
| C(52)-H(52B) | 0.9900 |
| C(52)-C(53) | 1.518(9) |
| C(53)-H(53A) | 0.9900 |
| C(53)-H(53B) | 0.9900 |
| C(53)-C(54) | 1.546(10) |
| C(54)-H(54A) | 0.9900 |
| C(54)-H(54B) | 0.9900 |
| C(54)-C(55) | 1.532(9) |
| C(55)-H(55A) | 0.9900 |
| C(55)-H(55B) | 0.9900 |
| C(55)-C(56) | 1.538(9) |
| C(56)-H(56) | 1.0000 |
| C(56)-C(57) | 1.570(9) |
| C(57)-H(57) | 1.0000 |
| C(57)-C(58) | 1.520(8) |
| C(57)-C(65) | 1.500(11) |

| | |
|---|---|
| C(59)-H(59) | 0.9500 |
| C(59)-C(60) | 1.3900 |
| C(59)-C(58) | 1.3900 |
| C(60)-H(60) | 0.9500 |
| C(60)-C(61) | 1.3900 |
| C(61)-C(62) | 1.3900 |
| C(62)-H(62) | 0.9500 |
| C(62)-C(63) | 1.3900 |
| C(63)-H(63) | 0.9500 |
| C(63)-C(58) | 1.3900 |
| C(64)-H(64A) | 0.9900 |
| C(64)-H(64B) | 0.9900 |
| C(64)-C(65) | 1.501(11) |
| C(65)-C(66) | 1.313(11) |
| C(66)-C(67) | 1.487(12) |
| C(69)-C(70) | 1.3900 |
| C(69)-C(74) | 1.3900 |
| C(69)-C(75) | 1.514(13) |
| C(69)-C(75A) | 1.67(3) |
| C(70)-H(70) | 0.9500 |
| C(70)-C(71) | 1.3900 |
| C(71)-H(71) | 0.9500 |
| C(71)-C(72) | 1.3900 |
| C(72)-H(72) | 0.9500 |
| C(72)-C(73) | 1.3900 |
| C(73)-H(73) | 0.9500 |
| C(73)-C(74) | 1.3900 |
| C(74)-H(74) | 0.9500 |
| C(75)-H(75A) | 0.9900 |
| C(75)-H(75B) | 0.9900 |
| C(75A)-H(75C) | 0.9900 |
| C(75A)-H(75D) | 0.9900 |
| C(31A)-H(31C) | 0.9900 |
| C(31A)-H(31D) | 0.9900 |

| | |
|---|---|
| C(1)-O(1)-C(2) | 119.1(6) |
| C(29)-O(4)-C(14) | 112.3(6) |
| C(6)-N(1)-C(1) | 122.7(6) |

| | |
|---|---|
| C(6)-N(1)-C(13) | 108.6(6) |
| C(13)-N(1)-C(1) | 128.3(7) |
| C(27)-N(2)-C(31) | 118.3(9) |
| C(27)-N(2)-C(31A) | 128.5(13) |
| C(30)-N(2)-C(27) | 111.1(7) |
| C(30)-N(2)-C(31) | 126.4(9) |
| C(30)-N(2)-C(31A) | 109.0(14) |
| O(1)-C(1)-N(1) | 110.7(6) |
| O(2)-C(1)-O(1) | 128.3(8) |
| O(2)-C(1)-N(1) | 121.0(8) |
| O(1)-C(2)-C(4) | 109.1(8) |
| O(1)-C(2)-C(5) | 109.0(7) |
| C(3)-C(2)-O(1) | 101.3(7) |
| C(3)-C(2)-C(4) | 109.7(9) |
| C(3)-C(2)-C(5) | 113.2(10) |
| C(4)-C(2)-C(5) | 113.8(8) |
| C(2)-C(3)-H(3A) | 109.5 |
| C(2)-C(3)-H(3B) | 109.5 |
| C(2)-C(3)-H(3C) | 109.5 |
| H(3A)-C(3)-H(3B) | 109.5 |
| H(3A)-C(3)-H(3C) | 109.5 |
| H(3B)-C(3)-H(3C) | 109.5 |
| C(2)-C(4)-H(4A) | 109.5 |
| C(2)-C(4)-H(4B) | 109.5 |
| C(2)-C(4)-H(4C) | 109.5 |
| H(4A)-C(4)-H(4B) | 109.5 |
| H(4A)-C(4)-H(4C) | 109.5 |
| H(4B)-C(4)-H(4C) | 109.5 |
| C(2)-C(5)-H(5A) | 109.5 |
| C(2)-C(5)-H(5B) | 109.5 |
| C(2)-C(5)-H(5C) | 109.5 |
| H(5A)-C(5)-H(5B) | 109.5 |
| H(5A)-C(5)-H(5C) | 109.5 |
| H(5B)-C(5)-H(5C) | 109.5 |
| C(10)-C(11)-C(6) | 120.0 |
| C(10)-C(11)-C(12) | 130.2(4) |
| C(6)-C(11)-C(12) | 109.8(4) |
| C(11)-C(10)-H(10) | 120.0 |

| | |
|---|---|
| C(11)-C(10)-C(9) | 120.0 |
| C(9)-C(10)-H(10) | 120.0 |
| C(10)-C(9)-H(9) | 120.0 |
| C(10)-C(9)-C(8) | 120.0 |
| C(8)-C(9)-H(9) | 120.0 |
| C(9)-C(8)-H(8) | 120.0 |
| C(7)-C(8)-C(9) | 120.0 |
| C(7)-C(8)-H(8) | 120.0 |
| C(8)-C(7)-H(7) | 120.0 |
| C(6)-C(7)-C(8) | 120.0 |
| C(6)-C(7)-H(7) | 120.0 |
| C(11)-C(6)-N(1) | 110.0(4) |
| C(7)-C(6)-N(1) | 130.0(4) |
| C(7)-C(6)-C(11) | 120.0 |
| C(11)-C(12)-H(12) | 109.2 |
| C(11)-C(12)-C(13) | 101.7(5) |
| C(11)-C(12)-C(14) | 114.7(6) |
| C(13)-C(12)-H(12) | 109.2 |
| C(13)-C(12)-C(14) | 112.4(6) |
| C(14)-C(12)-H(12) | 109.2 |
| O(3)-C(13)-N(1) | 124.1(7) |
| O(3)-C(13)-C(12) | 126.4(7) |
| N(1)-C(13)-C(12) | 109.4(6) |
| O(4)-C(14)-C(12) | 106.1(6) |
| O(4)-C(14)-C(15) | 105.1(6) |
| O(4)-C(14)-C(19) | 110.1(6) |
| C(15)-C(14)-C(12) | 109.8(6) |
| C(19)-C(14)-C(12) | 113.6(6) |
| C(19)-C(14)-C(15) | 111.6(6) |
| C(14)-C(15)-H(15A) | 109.1 |
| C(14)-C(15)-H(15B) | 109.1 |
| H(15A)-C(15)-H(15B) | 107.8 |
| C(16)-C(15)-C(14) | 112.5(6) |
| C(16)-C(15)-H(15A) | 109.1 |
| C(16)-C(15)-H(15B) | 109.1 |
| C(15)-C(16)-H(16A) | 109.7 |
| C(15)-C(16)-H(16B) | 109.7 |
| H(16A)-C(16)-H(16B) | 108.2 |

| | |
|---|---|
| C(17)-C(16)-C(15) | 109.8(6) |
| C(17)-C(16)-H(16A) | 109.7 |
| C(17)-C(16)-H(16B) | 109.7 |
| C(16)-C(17)-H(17A) | 109.6 |
| C(16)-C(17)-H(17B) | 109.6 |
| C(16)-C(17)-C(18) | 110.2(6) |
| H(17A)-C(17)-H(17B) | 108.1 |
| C(18)-C(17)-H(17A) | 109.6 |
| C(18)-C(17)-H(17B) | 109.6 |
| C(17)-C(18)-H(18A) | 108.9 |
| C(17)-C(18)-H(18B) | 108.9 |
| H(18A)-C(18)-H(18B) | 107.7 |
| C(19)-C(18)-C(17) | 113.2(6) |
| C(19)-C(18)-H(18A) | 108.9 |
| C(19)-C(18)-H(18B) | 108.9 |
| C(14)-C(19)-C(18) | 110.5(6) |
| C(14)-C(19)-H(19) | 107.1 |
| C(14)-C(19)-C(20) | 111.9(6) |
| C(18)-C(19)-H(19) | 107.1 |
| C(18)-C(19)-C(20) | 112.9(6) |
| C(20)-C(19)-H(19) | 107.1 |
| C(19)-C(20)-H(20) | 107.5 |
| C(21)-C(20)-C(19) | 112.3(6) |
| C(21)-C(20)-H(20) | 107.5 |
| C(28)-C(20)-C(19) | 106.5(6) |
| C(28)-C(20)-H(20) | 107.5 |
| C(28)-C(20)-C(21) | 115.2(6) |
| C(22)-C(21)-C(20) | 117.5(4) |
| C(22)-C(21)-C(26) | 120.0 |
| C(26)-C(21)-C(20) | 122.1(4) |
| C(21)-C(22)-H(22) | 120.0 |
| C(21)-C(22)-C(23) | 120.0 |
| C(23)-C(22)-H(22) | 120.0 |
| C(22)-C(23)-H(23) | 120.0 |
| C(22)-C(23)-C(24) | 120.0 |
| C(24)-C(23)-H(23) | 120.0 |
| C(23)-C(24)-Cl(1) | 122.3(4) |
| C(25)-C(24)-Cl(1) | 117.6(4) |

| | |
|---|---|
| C(25)-C(24)-C(23) | 120.0 |
| C(24)-C(25)-H(25) | 120.0 |
| C(24)-C(25)-C(26) | 120.0 |
| C(26)-C(25)-H(25) | 120.0 |
| C(21)-C(26)-H(26) | 120.0 |
| C(25)-C(26)-C(21) | 120.0 |
| C(25)-C(26)-H(26) | 120.0 |
| N(2)-C(27)-H(27A) | 110.9 |
| N(2)-C(27)-H(27B) | 110.9 |
| N(2)-C(27)-C(28) | 104.4(7) |
| H(27A)-C(27)-H(27B) | 108.9 |
| C(28)-C(27)-H(27A) | 110.9 |
| C(28)-C(27)-H(27B) | 110.9 |
| C(27)-C(28)-C(20) | 130.6(7) |
| C(29)-C(28)-C(20) | 121.6(7) |
| C(29)-C(28)-C(27) | 107.9(7) |
| O(4)-C(29)-C(30) | 119.3(7) |
| C(28)-C(29)-O(4) | 129.4(8) |
| C(28)-C(29)-C(30) | 111.3(7) |
| O(5)-C(30)-N(2) | 126.6(9) |
| O(5)-C(30)-C(29) | 128.0(8) |
| N(2)-C(30)-C(29) | 105.3(8) |
| N(2)-C(31)-H(31A) | 109.0 |
| N(2)-C(31)-H(31B) | 109.0 |
| H(31A)-C(31)-H(31B) | 107.8 |
| C(32)-C(31)-N(2) | 112.9(9) |
| C(32)-C(31)-H(31A) | 109.0 |
| C(32)-C(31)-H(31B) | 109.0 |
| C(33)-C(32)-C(31) | 119.2(7) |
| C(33)-C(32)-C(37) | 120.0 |
| C(33)-C(32)-C(31A) | 69.8(13) |
| C(37)-C(32)-C(31) | 120.8(7) |
| C(37)-C(32)-C(31A) | 169.0(13) |
| C(32)-C(33)-H(33) | 120.0 |
| C(34)-C(33)-C(32) | 120.0 |
| C(34)-C(33)-H(33) | 120.0 |
| C(33)-C(34)-H(34) | 120.0 |
| C(33)-C(34)-C(35) | 120.0 |

| | |
|---|---|
| C(35)-C(34)-H(34) | 120.0 |
| C(34)-C(35)-H(35) | 120.0 |
| C(36)-C(35)-C(34) | 120.0 |
| C(36)-C(35)-H(35) | 120.0 |
| C(35)-C(36)-H(36) | 120.0 |
| C(35)-C(36)-C(37) | 120.0 |
| C(37)-C(36)-H(36) | 120.0 |
| C(32)-C(37)-H(37) | 120.0 |
| C(36)-C(37)-C(32) | 120.0 |
| C(36)-C(37)-H(37) | 120.0 |
| C(38)-O(6)-C(39) | 117.7(6) |
| C(66)-O(9)-C(51) | 112.5(5) |
| C(38)-N(3)-C(50) | 126.5(6) |
| C(43)-N(3)-C(38) | 122.4(6) |
| C(43)-N(3)-C(50) | 110.7(5) |
| C(64)-N(4)-C(75) | 128.1(8) |
| C(64)-N(4)-C(75A) | 117.0(15) |
| C(67)-N(4)-C(64) | 112.6(7) |
| C(67)-N(4)-C(75) | 116.3(9) |
| C(67)-N(4)-C(75A) | 120.3(14) |
| O(6)-C(38)-N(3) | 110.4(7) |
| O(7)-C(38)-O(6) | 127.6(8) |
| O(7)-C(38)-N(3) | 122.0(8) |
| O(6)-C(39)-C(40) | 101.2(7) |
| O(6)-C(39)-C(41) | 110.3(7) |
| O(6)-C(39)-C(42) | 109.9(7) |
| C(41)-C(39)-C(40) | 113.0(9) |
| C(42)-C(39)-C(40) | 109.3(9) |
| C(42)-C(39)-C(41) | 112.7(8) |
| C(39)-C(40)-H(40A) | 109.5 |
| C(39)-C(40)-H(40B) | 109.5 |
| C(39)-C(40)-H(40C) | 109.5 |
| H(40A)-C(40)-H(40B) | 109.5 |
| H(40A)-C(40)-H(40C) | 109.5 |
| H(40B)-C(40)-H(40C) | 109.5 |
| C(39)-C(41)-H(41A) | 109.5 |
| C(39)-C(41)-H(41B) | 109.5 |
| C(39)-C(41)-H(41C) | 109.5 |

| | |
|---|---|
| H(41A)-C(41)-H(41B) | 109.5 |
| H(41A)-C(41)-H(41C) | 109.5 |
| H(41B)-C(41)-H(41C) | 109.5 |
| C(39)-C(42)-H(42A) | 109.5 |
| C(39)-C(42)-H(42B) | 109.5 |
| C(39)-C(42)-H(42C) | 109.5 |
| H(42A)-C(42)-H(42B) | 109.5 |
| H(42A)-C(42)-H(42C) | 109.5 |
| H(42B)-C(42)-H(42C) | 109.5 |
| C(47)-C(48)-C(43) | 120.0 |
| C(47)-C(48)-C(49) | 129.9(4) |
| C(43)-C(48)-C(49) | 110.1(4) |
| C(48)-C(47)-H(47) | 120.0 |
| C(46)-C(47)-C(48) | 120.0 |
| C(46)-C(47)-H(47) | 120.0 |
| C(47)-C(46)-H(46) | 120.0 |
| C(47)-C(46)-C(45) | 120.0 |
| C(45)-C(46)-H(46) | 120.0 |
| C(46)-C(45)-H(45) | 120.0 |
| C(44)-C(45)-C(46) | 120.0 |
| C(44)-C(45)-H(45) | 120.0 |
| C(45)-C(44)-H(44) | 120.0 |
| C(43)-C(44)-C(45) | 120.0 |
| C(43)-C(44)-H(44) | 120.0 |
| C(48)-C(43)-N(3) | 109.3(4) |
| C(44)-C(43)-N(3) | 130.7(4) |
| C(44)-C(43)-C(48) | 120.0 |
| C(48)-C(49)-H(49) | 108.9 |
| C(48)-C(49)-C(50) | 102.9(5) |
| C(48)-C(49)-C(51) | 114.9(5) |
| C(50)-C(49)-H(49) | 108.9 |
| C(50)-C(49)-C(51) | 112.1(6) |
| C(51)-C(49)-H(49) | 108.9 |
| O(8)-C(50)-N(3) | 126.9(7) |
| O(8)-C(50)-C(49) | 126.7(7) |
| N(3)-C(50)-C(49) | 106.4(6) |
| O(9)-C(51)-C(49) | 105.7(5) |
| O(9)-C(51)-C(52) | 105.6(5) |

| | |
|---|---|
| O(9)-C(51)-C(56) | 109.4(5) |
| C(52)-C(51)-C(49) | 110.9(5) |
| C(52)-C(51)-C(56) | 110.9(6) |
| C(56)-C(51)-C(49) | 113.8(5) |
| C(51)-C(52)-H(52A) | 108.6 |
| C(51)-C(52)-H(52B) | 108.6 |
| H(52A)-C(52)-H(52B) | 107.6 |
| C(53)-C(52)-C(51) | 114.6(6) |
| C(53)-C(52)-H(52A) | 108.6 |
| C(53)-C(52)-H(52B) | 108.6 |
| C(52)-C(53)-H(53A) | 109.7 |
| C(52)-C(53)-H(53B) | 109.7 |
| C(52)-C(53)-C(54) | 109.8(6) |
| H(53A)-C(53)-H(53B) | 108.2 |
| C(54)-C(53)-H(53A) | 109.7 |
| C(54)-C(53)-H(53B) | 109.7 |
| C(53)-C(54)-H(54A) | 109.7 |
| C(53)-C(54)-H(54B) | 109.7 |
| H(54A)-C(54)-H(54B) | 108.2 |
| C(55)-C(54)-C(53) | 110.0(6) |
| C(55)-C(54)-H(54A) | 109.7 |
| C(55)-C(54)-H(54B) | 109.7 |
| C(54)-C(55)-H(55A) | 109.2 |
| C(54)-C(55)-H(55B) | 109.2 |
| C(54)-C(55)-C(56) | 111.9(6) |
| H(55A)-C(55)-H(55B) | 107.9 |
| C(56)-C(55)-H(55A) | 109.2 |
| C(56)-C(55)-H(55B) | 109.2 |
| C(51)-C(56)-H(56) | 108.0 |
| C(51)-C(56)-C(57) | 111.3(6) |
| C(55)-C(56)-C(51) | 109.8(6) |
| C(55)-C(56)-H(56) | 108.0 |
| C(55)-C(56)-C(57) | 111.6(6) |
| C(57)-C(56)-H(56) | 108.0 |
| C(56)-C(57)-H(57) | 106.7 |
| C(58)-C(57)-C(56) | 111.8(6) |
| C(58)-C(57)-H(57) | 106.7 |
| C(65)-C(57)-C(56) | 107.4(6) |

| | |
|---|---|
| C(65)-C(57)-H(57) | 106.7 |
| C(65)-C(57)-C(58) | 116.9(6) |
| C(60)-C(59)-H(59) | 120.0 |
| C(60)-C(59)-C(58) | 120.0 |
| C(58)-C(59)-H(59) | 120.0 |
| C(59)-C(60)-H(60) | 120.0 |
| C(59)-C(60)-C(61) | 120.0 |
| C(61)-C(60)-H(60) | 120.0 |
| C(60)-C(61)-Cl(2) | 119.4(3) |
| C(62)-C(61)-Cl(2) | 120.5(3) |
| C(62)-C(61)-C(60) | 120.0 |
| C(61)-C(62)-H(62) | 120.0 |
| C(61)-C(62)-C(63) | 120.0 |
| C(63)-C(62)-H(62) | 120.0 |
| C(62)-C(63)-H(63) | 120.0 |
| C(58)-C(63)-C(62) | 120.0 |
| C(58)-C(63)-H(63) | 120.0 |
| C(59)-C(58)-C(57) | 118.4(4) |
| C(63)-C(58)-C(57) | 121.0(4) |
| C(63)-C(58)-C(59) | 120.0 |
| N(4)-C(64)-H(64A) | 111.2 |
| N(4)-C(64)-H(64B) | 111.2 |
| N(4)-C(64)-C(65) | 103.1(7) |
| H(64A)-C(64)-H(64B) | 109.1 |
| C(65)-C(64)-H(64A) | 111.2 |
| C(65)-C(64)-H(64B) | 111.2 |
| C(57)-C(65)-C(64) | 129.3(7) |
| C(66)-C(65)-C(57) | 122.7(7) |
| C(66)-C(65)-C(64) | 108.0(7) |
| O(9)-C(66)-C(67) | 118.7(7) |
| C(65)-C(66)-O(9) | 128.8(7) |
| C(65)-C(66)-C(67) | 112.3(7) |
| O(10)-C(67)-N(4) | 127.5(8) |
| O(10)-C(67)-C(66) | 128.5(8) |
| N(4)-C(67)-C(66) | 103.9(7) |
| C(70)-C(69)-C(74) | 120.0 |
| C(70)-C(69)-C(75) | 118.0(7) |
| C(70)-C(69)-C(75A) | 160.7(12) |

| | |
|---|---|
| C(74)-C(69)-C(75) | 121.8(7) |
| C(74)-C(69)-C(75A) | 78.7(12) |
| C(69)-C(70)-H(70) | 120.0 |
| C(71)-C(70)-C(69) | 120.0 |
| C(71)-C(70)-H(70) | 120.0 |
| C(70)-C(71)-H(71) | 120.0 |
| C(70)-C(71)-C(72) | 120.0 |
| C(72)-C(71)-H(71) | 120.0 |
| C(71)-C(72)-H(72) | 120.0 |
| C(73)-C(72)-C(71) | 120.0 |
| C(73)-C(72)-H(72) | 120.0 |
| C(72)-C(73)-H(73) | 120.0 |
| C(72)-C(73)-C(74) | 120.0 |
| C(74)-C(73)-H(73) | 120.0 |
| C(69)-C(74)-H(74) | 120.0 |
| C(73)-C(74)-C(69) | 120.0 |
| C(73)-C(74)-H(74) | 120.0 |
| N(4)-C(75)-H(75A) | 109.8 |
| N(4)-C(75)-H(75B) | 109.8 |
| C(69)-C(75)-N(4) | 109.6(9) |
| C(69)-C(75)-H(75A) | 109.8 |
| C(69)-C(75)-H(75B) | 109.8 |
| H(75A)-C(75)-H(75B) | 108.2 |
| N(4)-C(75A)-C(69) | 101(2) |
| N(4)-C(75A)-H(75C) | 111.5 |
| N(4)-C(75A)-H(75D) | 111.5 |
| C(69)-C(75A)-H(75C) | 111.5 |
| C(69)-C(75A)-H(75D) | 111.5 |
| H(75C)-C(75A)-H(75D) | 109.3 |
| N(2)-C(31A)-C(32) | 97(2) |
| N(2)-C(31A)-H(31C) | 112.5 |
| N(2)-C(31A)-H(31D) | 112.5 |
| C(32)-C(31A)-H(31C) | 112.5 |
| C(32)-C(31A)-H(31D) | 112.5 |
| H(31C)-C(31A)-H(31D) | 110.0 |

Symmetry transformations used to generate equivalent atoms:

**Table S12.** Anisotropic displacement parameters ($Å^2$x $10^3$) for **5bf**. The anisotropic displacement factor exponent takes the form: $-2p^2[ h^2 a^{*2}U^{11} + ... + 2 h k a^* b^* U^{12} ]$

| | $U^{11}$ | $U^{22}$ | $U^{33}$ | $U^{23}$ | $U^{13}$ | $U^{12}$ |
|---|---|---|---|---|---|---|
| Cl(1) | 50(2) | 103(3) | 114(3) | -73(2) | 59(2) | -43(2) |
| O(1) | 30(3) | 40(4) | 54(4) | 14(3) | 22(3) | 5(3) |
| O(2) | 43(4) | 52(4) | 61(4) | 8(3) | 40(3) | 1(3) |
| O(3) | 29(3) | 44(4) | 40(3) | 20(3) | 19(3) | 10(3) |
| O(4) | 13(2) | 34(3) | 22(3) | 5(2) | 2(2) | -4(2) |
| O(5) | 44(4) | 29(4) | 160(9) | 3(5) | -38(5) | 6(3) |
| N(1) | 24(3) | 33(4) | 24(3) | 5(3) | 11(3) | 6(3) |
| N(2) | 41(4) | 28(4) | 81(6) | 6(4) | -16(4) | -18(3) |
| C(1) | 27(3) | 42(4) | 26(3) | 12(3) | 11(3) | -3(3) |
| C(2) | 33(4) | 39(5) | 58(5) | 24(4) | 17(4) | -2(4) |
| C(3) | 55(6) | 52(6) | 107(8) | 23(6) | 50(6) | 10(5) |
| C(4) | 72(7) | 59(7) | 69(7) | 37(6) | 0(6) | -7(6) |
| C(5) | 39(5) | 55(6) | 56(6) | -4(5) | 15(4) | -10(4) |
| C(11) | 29(3) | 37(3) | 13(3) | -5(3) | 8(2) | -2(3) |
| C(10) | 36(3) | 45(4) | 22(3) | -3(3) | 15(3) | -3(3) |
| C(9) | 54(4) | 39(4) | 28(4) | -2(3) | 15(3) | 11(4) |
| C(8) | 42(4) | 48(4) | 31(4) | -3(3) | 18(3) | 8(4) |
| C(7) | 34(3) | 39(4) | 20(3) | 3(3) | 13(3) | 1(3) |
| C(6) | 27(3) | 42(4) | 20(3) | 1(3) | 2(3) | -4(3) |
| C(12) | 23(3) | 37(3) | 17(3) | 3(3) | 6(2) | -7(3) |
| C(13) | 23(3) | 37(3) | 21(3) | 8(3) | 10(2) | -2(3) |
| C(14) | 23(3) | 31(3) | 21(3) | 3(3) | 6(2) | -7(3) |
| C(15) | 21(3) | 40(3) | 20(3) | 2(3) | 4(2) | -7(3) |
| C(16) | 26(3) | 51(4) | 20(3) | -2(3) | 3(3) | -7(3) |
| C(17) | 30(4) | 49(4) | 17(3) | -1(3) | 7(3) | -11(3) |
| C(18) | 24(3) | 36(3) | 19(3) | 1(3) | 7(2) | -6(3) |
| C(19) | 22(3) | 32(3) | 22(3) | 3(3) | 6(2) | -9(3) |
| C(20) | 22(3) | 33(3) | 25(3) | 4(3) | 10(2) | -8(3) |
| C(21) | 23(3) | 42(4) | 27(3) | -3(3) | 7(3) | -8(3) |
| C(22) | 25(3) | 43(4) | 34(3) | -4(3) | 8(3) | -11(3) |
| C(23) | 29(3) | 48(4) | 47(4) | -13(4) | 13(3) | -10(3) |
| C(24) | 28(4) | 52(4) | 40(4) | -18(4) | 16(3) | -23(3) |
| C(25) | 32(4) | 67(5) | 43(4) | -5(4) | 24(3) | -18(4) |

| | | | | | | |
|---|---|---|---|---|---|---|
| C(26) | 25(3) | 50(4) | 32(3) | 10(3) | 7(3) | -5(3) |
| C(27) | 32(3) | 38(4) | 51(4) | 8(3) | -2(3) | -6(3) |
| C(28) | 20(3) | 33(3) | 29(3) | 5(3) | 7(2) | -10(3) |
| C(29) | 29(3) | 34(3) | 26(3) | 1(3) | 6(2) | -7(3) |
| C(30) | 35(4) | 36(4) | 56(4) | 9(4) | -9(3) | -3(3) |
| C(31) | 46(3) | 39(3) | 53(3) | 3(3) | 11(3) | -8(3) |
| C(32) | 40(3) | 34(4) | 60(4) | -2(3) | 1(3) | -10(3) |
| C(33) | 67(4) | 54(4) | 70(4) | 2(4) | 25(2) | -6(4) |
| C(34) | 69(2) | 69(2) | 69(2) | 0(1) | 24(1) | 0(1) |
| C(35) | 57(3) | 47(4) | 76(5) | 0(4) | 14(3) | 4(4) |
| C(36) | 53(4) | 39(4) | 72(4) | -2(4) | 22(3) | -2(4) |
| C(37) | 70(4) | 45(4) | 64(4) | -7(4) | 18(3) | 2(4) |
| Cl(2) | 42(1) | 58(2) | 77(2) | -33(1) | 38(1) | -20(1) |
| O(6) | 33(3) | 16(3) | 63(4) | 15(3) | 23(3) | 3(2) |
| O(7) | 50(4) | 41(4) | 61(4) | 10(3) | 43(3) | -9(3) |
| O(8) | 22(3) | 25(3) | 46(3) | 15(2) | 18(2) | 8(2) |
| O(9) | 22(3) | 21(3) | 24(3) | 8(2) | 3(2) | -5(2) |
| O(10) | 38(4) | 18(3) | 157(8) | -2(4) | -12(4) | -2(3) |
| N(3) | 23(3) | 26(4) | 34(4) | 9(3) | 11(3) | -2(3) |
| N(4) | 28(4) | 22(4) | 97(7) | 9(4) | -10(4) | -7(3) |
| C(38) | 30(3) | 31(4) | 37(4) | 11(3) | 11(3) | 6(3) |
| C(39) | 39(4) | 24(4) | 59(5) | 13(4) | 12(4) | -6(4) |
| C(40) | 59(6) | 25(5) | 113(8) | 13(5) | 33(6) | 0(4) |
| C(41) | 64(6) | 40(5) | 68(6) | 35(5) | 4(5) | -18(5) |
| C(42) | 41(5) | 31(5) | 62(6) | 1(4) | 23(4) | -14(4) |
| C(48) | 24(3) | 27(3) | 21(3) | 4(3) | 9(2) | -1(3) |
| C(47) | 34(3) | 24(3) | 25(3) | 3(3) | 10(3) | 1(3) |
| C(46) | 46(4) | 35(4) | 32(4) | -3(3) | 14(3) | 12(3) |
| C(45) | 32(4) | 45(4) | 30(4) | 6(3) | 16(3) | 11(3) |
| C(44) | 25(3) | 39(4) | 25(3) | 8(3) | 13(3) | 2(3) |
| C(43) | 24(3) | 25(3) | 22(3) | 2(3) | 7(2) | 2(3) |
| C(49) | 19(3) | 24(3) | 18(3) | 4(2) | 6(2) | -3(2) |
| C(50) | 23(3) | 31(3) | 19(3) | 9(3) | 3(2) | 3(3) |
| C(51) | 16(3) | 23(3) | 18(3) | 2(2) | 2(2) | -3(2) |
| C(52) | 20(3) | 26(3) | 22(3) | 3(3) | 4(2) | -4(3) |
| C(53) | 24(3) | 40(4) | 19(3) | 0(3) | -4(3) | -9(3) |
| C(54) | 31(4) | 39(4) | 15(3) | -2(3) | 2(3) | -6(3) |
| C(55) | 23(3) | 31(3) | 20(3) | 3(3) | 3(2) | -6(3) |

| | | | | | | |
|---|---|---|---|---|---|---|
| C(56) | 19(3) | 28(3) | 21(3) | 2(2) | 5(2) | -5(2) |
| C(57) | 19(3) | 26(3) | 20(3) | 6(2) | 1(2) | -5(2) |
| C(59) | 26(3) | 26(3) | 31(3) | -3(3) | 7(3) | -9(3) |
| C(60) | 24(3) | 27(4) | 45(4) | -13(3) | 11(3) | -6(3) |
| C(61) | 19(3) | 41(4) | 37(4) | -20(3) | 15(3) | -3(3) |
| C(62) | 28(3) | 35(4) | 37(4) | 2(3) | 12(3) | -11(3) |
| C(63) | 25(3) | 32(3) | 35(3) | 4(3) | 12(3) | -3(3) |
| C(58) | 21(3) | 26(3) | 25(3) | 1(3) | 6(2) | -10(3) |
| C(64) | 34(3) | 32(3) | 51(4) | 2(3) | 4(3) | -7(3) |
| C(65) | 20(3) | 22(3) | 29(3) | 2(3) | 2(2) | -6(2) |
| C(66) | 22(3) | 25(3) | 27(3) | 5(2) | 5(2) | -4(2) |
| C(67) | 32(3) | 28(3) | 48(4) | 0(3) | -4(3) | -6(3) |
| C(69) | 39(3) | 21(3) | 52(4) | -8(3) | 12(3) | -10(3) |
| C(70) | 64(2) | 61(2) | 64(2) | -1(1) | 22(1) | 0(1) |
| C(71) | 62(4) | 41(4) | 67(5) | 3(4) | 6(4) | -7(4) |
| C(72) | 55(4) | 32(4) | 63(5) | -6(4) | 12(4) | -7(4) |
| C(73) | 62(4) | 37(4) | 69(5) | -7(4) | 25(4) | -2(4) |
| C(74) | 63(4) | 37(4) | 62(4) | -4(4) | 22(4) | -8(4) |
| C(75) | 45(3) | 39(3) | 55(3) | -7(3) | 13(3) | -7(3) |
| C(75A) | 45(3) | 39(3) | 55(3) | -7(3) | 13(3) | -7(3) |
| C(31A) | 46(3) | 39(3) | 53(3) | 3(3) | 11(3) | -8(3) |

**Table S13**. Hydrogen coordinates ( x $10^4$) and isotropic displacement parameters ($Å^2$x $10^3$) for **5bf**.

| | x | y | z | U(eq) |
|---|---|---|---|---|
| H(3A) | 1979 | 5395 | 3596 | 99 |
| H(3B) | 2910 | 6031 | 3784 | 99 |
| H(3C) | 3071 | 5483 | 4613 | 99 |
| H(4A) | 3782 | 4893 | 2267 | 110 |
| H(4B) | 3554 | 5689 | 2374 | 110 |
| H(4C) | 2433 | 5159 | 2205 | 110 |
| H(5A) | 5242 | 5200 | 4812 | 76 |
| H(5B) | 5263 | 5753 | 4029 | 76 |
| H(5C) | 5581 | 4972 | 3902 | 76 |
| H(10) | 3319 | 1102 | 4022 | 40 |
| H(9) | 5084 | 866 | 3627 | 48 |
| H(8) | 6221 | 1759 | 3290 | 47 |
| H(7) | 5594 | 2889 | 3349 | 36 |
| H(12) | 1544 | 2236 | 3725 | 31 |
| H(15A) | 4382 | 2239 | 5763 | 33 |
| H(15B) | 3843 | 2978 | 5858 | 33 |
| H(16A) | 3845 | 1831 | 7059 | 41 |
| H(16B) | 4712 | 2493 | 7398 | 41 |
| H(17A) | 2944 | 3181 | 7187 | 39 |
| H(17B) | 2941 | 2562 | 7889 | 39 |
| H(18A) | 1466 | 1963 | 6622 | 32 |
| H(18B) | 918 | 2702 | 6699 | 32 |
| H(19) | 1439 | 3095 | 5408 | 31 |
| H(20) | -54 | 2572 | 4149 | 31 |
| H(22) | -1195 | 3509 | 4368 | 41 |
| H(23) | -2641 | 3992 | 4968 | 50 |
| H(25) | -2478 | 2297 | 6562 | 54 |
| H(26) | -1033 | 1814 | 5962 | 44 |
| H(27A) | -1448 | 1090 | 4670 | 54 |
| H(27B) | -1384 | 1187 | 3616 | 54 |
| H(31A) | -383 | -584 | 4479 | 57 |
| H(31B) | -1184 | -177 | 4993 | 57 |

| | | | | |
|---|---|---|---|---|
| H(33) | -1218 | -79 | 2598 | 76 |
| H(34) | -3011 | -173 | 1226 | 83 |
| H(35) | -4936 | -427 | 1370 | 75 |
| H(36) | -5068 | -586 | 2885 | 65 |
| H(37) | -3275 | -492 | 4257 | 73 |
| H(40A) | 8076 | 5414 | 9569 | 97 |
| H(40B) | 6965 | 5350 | 8556 | 97 |
| H(40C) | 7932 | 5971 | 8753 | 97 |
| H(41A) | 8554 | 5606 | 7283 | 94 |
| H(41B) | 7468 | 5051 | 7091 | 94 |
| H(41C) | 8853 | 4816 | 7206 | 94 |
| H(42A) | 10179 | 5712 | 9033 | 65 |
| H(42B) | 10572 | 4968 | 8802 | 65 |
| H(42C) | 10223 | 5090 | 9738 | 65 |
| H(47) | 8429 | 1032 | 9072 | 33 |
| H(46) | 10214 | 800 | 8703 | 45 |
| H(45) | 11322 | 1693 | 8333 | 41 |
| H(44) | 10645 | 2819 | 8331 | 35 |
| H(49) | 6616 | 2142 | 8724 | 25 |
| H(52A) | 9422 | 2175 | 10764 | 29 |
| H(52B) | 8876 | 2915 | 10833 | 29 |
| H(53A) | 9754 | 2482 | 12377 | 37 |
| H(53B) | 8949 | 1795 | 12080 | 37 |
| H(54A) | 7955 | 2517 | 12874 | 36 |
| H(54B) | 7919 | 3132 | 12156 | 36 |
| H(55A) | 5929 | 2597 | 11682 | 31 |
| H(55B) | 6546 | 1878 | 11600 | 31 |
| H(56) | 6476 | 3037 | 10400 | 28 |
| H(57) | 4979 | 2510 | 9140 | 28 |
| H(59) | 3849 | 3442 | 9425 | 34 |
| H(60) | 2442 | 3892 | 10089 | 38 |
| H(62) | 2614 | 2136 | 11562 | 40 |
| H(63) | 4021 | 1686 | 10898 | 37 |
| H(64A) | 3608 | 993 | 9513 | 50 |
| H(64B) | 3719 | 1156 | 8486 | 50 |
| H(70) | 2637 | -141 | 6957 | 76 |
| H(71) | 484 | -329 | 6485 | 73 |
| H(72) | -404 | -622 | 7626 | 63 |

| | | | | |
|---|---|---|---|---|
| H(73) | 861 | -726 | 9238 | 66 |
| H(74) | 3014 | -538 | 9710 | 65 |
| H(75A) | 4632 | -236 | 8045 | 57 |
| H(75B) | 4853 | -652 | 9016 | 57 |
| H(75C) | 4001 | -212 | 9954 | 57 |
| H(75D) | 4823 | -655 | 9481 | 57 |
| H(31C) | -33 | -584 | 3925 | 57 |
| H(31D) | -461 | -53 | 3035 | 57 |

**Table S14**. Torsion angles [°] for **5bf**.

| | |
|---|---|
| Cl(1)-C(24)-C(25)-C(26) | -176.7(4) |
| O(4)-C(14)-C(15)-C(16) | -64.5(8) |
| O(4)-C(14)-C(19)-C(18) | 64.4(7) |
| O(4)-C(14)-C(19)-C(20) | -62.3(7) |
| O(4)-C(29)-C(30)-O(5) | -0.1(17) |
| O(4)-C(29)-C(30)-N(2) | -179.1(8) |
| N(2)-C(27)-C(28)-C(20) | -179.5(8) |
| N(2)-C(27)-C(28)-C(29) | 1.4(10) |
| N(2)-C(31)-C(32)-C(33) | 47.1(12) |
| N(2)-C(31)-C(32)-C(37) | -130.4(9) |
| C(1)-O(1)-C(2)-C(3) | 178.2(8) |
| C(1)-O(1)-C(2)-C(4) | 62.6(10) |
| C(1)-O(1)-C(2)-C(5) | -62.2(10) |
| C(1)-N(1)-C(6)-C(11) | -175.2(6) |
| C(1)-N(1)-C(6)-C(7) | 3.2(9) |
| C(1)-N(1)-C(13)-O(3) | -1.4(12) |
| C(1)-N(1)-C(13)-C(12) | 178.7(7) |
| C(2)-O(1)-C(1)-O(2) | 1.1(13) |
| C(2)-O(1)-C(1)-N(1) | -178.9(7) |
| C(11)-C(10)-C(9)-C(8) | 0.0 |
| C(11)-C(12)-C(13)-O(3) | 173.3(7) |
| C(11)-C(12)-C(13)-N(1) | -6.8(7) |
| C(11)-C(12)-C(14)-O(4) | -61.6(7) |
| C(11)-C(12)-C(14)-C(15) | 51.5(8) |
| C(11)-C(12)-C(14)-C(19) | 177.2(6) |
| C(10)-C(11)-C(6)-N(1) | 178.6(5) |
| C(10)-C(11)-C(6)-C(7) | 0.0 |
| C(10)-C(11)-C(12)-C(13) | -176.0(4) |
| C(10)-C(11)-C(12)-C(14) | 62.4(8) |
| C(10)-C(9)-C(8)-C(7) | 0.0 |
| C(9)-C(8)-C(7)-C(6) | 0.0 |
| C(8)-C(7)-C(6)-N(1) | -178.3(6) |
| C(8)-C(7)-C(6)-C(11) | 0.0 |
| C(6)-N(1)-C(1)-O(1) | -171.1(6) |
| C(6)-N(1)-C(1)-O(2) | 8.8(12) |
| C(6)-N(1)-C(13)-O(3) | -174.6(7) |

| | |
|---|---|
| C(6)-N(1)-C(13)-C(12) | 5.4(8) |
| C(6)-C(11)-C(10)-C(9) | 0.0 |
| C(6)-C(11)-C(12)-C(13) | 5.9(6) |
| C(6)-C(11)-C(12)-C(14) | -115.7(6) |
| C(12)-C(11)-C(10)-C(9) | -178.0(6) |
| C(12)-C(11)-C(6)-N(1) | -3.0(5) |
| C(12)-C(11)-C(6)-C(7) | 178.4(5) |
| C(12)-C(14)-C(15)-C(16) | -178.2(7) |
| C(12)-C(14)-C(19)-C(18) | -176.8(6) |
| C(12)-C(14)-C(19)-C(20) | 56.5(8) |
| C(13)-N(1)-C(1)-O(1) | 16.5(11) |
| C(13)-N(1)-C(1)-O(2) | -163.6(8) |
| C(13)-N(1)-C(6)-C(11) | -1.5(6) |
| C(13)-N(1)-C(6)-C(7) | 176.9(4) |
| C(13)-C(12)-C(14)-O(4) | -177.2(5) |
| C(13)-C(12)-C(14)-C(15) | -64.1(8) |
| C(13)-C(12)-C(14)-C(19) | 61.7(8) |
| C(14)-O(4)-C(29)-C(28) | -13.0(11) |
| C(14)-O(4)-C(29)-C(30) | 167.2(7) |
| C(14)-C(12)-C(13)-O(3) | -63.5(10) |
| C(14)-C(12)-C(13)-N(1) | 116.4(6) |
| C(14)-C(15)-C(16)-C(17) | -56.9(9) |
| C(14)-C(19)-C(20)-C(21) | 173.4(6) |
| C(14)-C(19)-C(20)-C(28) | 46.4(8) |
| C(15)-C(14)-C(19)-C(18) | -51.9(8) |
| C(15)-C(14)-C(19)-C(20) | -178.6(6) |
| C(15)-C(16)-C(17)-C(18) | 56.7(9) |
| C(16)-C(17)-C(18)-C(19) | -56.8(9) |
| C(17)-C(18)-C(19)-C(14) | 54.0(9) |
| C(17)-C(18)-C(19)-C(20) | -179.9(7) |
| C(18)-C(19)-C(20)-C(21) | 48.0(8) |
| C(18)-C(19)-C(20)-C(28) | -79.0(8) |
| C(19)-C(14)-C(15)-C(16) | 54.8(9) |
| C(19)-C(20)-C(21)-C(22) | 86.7(6) |
| C(19)-C(20)-C(21)-C(26) | -85.9(6) |
| C(19)-C(20)-C(28)-C(27) | 164.6(8) |
| C(19)-C(20)-C(28)-C(29) | -16.5(10) |
| C(20)-C(21)-C(22)-C(23) | -172.8(5) |

| | |
|---|---|
| C(20)-C(21)-C(26)-C(25) | 172.5(5) |
| C(20)-C(28)-C(29)-O(4) | -0.5(13) |
| C(20)-C(28)-C(29)-C(30) | 179.3(7) |
| C(21)-C(20)-C(28)-C(27) | 39.4(12) |
| C(21)-C(20)-C(28)-C(29) | -141.7(7) |
| C(21)-C(22)-C(23)-C(24) | 0.0 |
| C(22)-C(21)-C(26)-C(25) | 0.0 |
| C(22)-C(23)-C(24)-Cl(1) | 176.5(4) |
| C(22)-C(23)-C(24)-C(25) | 0.0 |
| C(23)-C(24)-C(25)-C(26) | 0.0 |
| C(24)-C(25)-C(26)-C(21) | 0.0 |
| C(26)-C(21)-C(22)-C(23) | 0.0 |
| C(27)-N(2)-C(30)-O(5) | -179.0(12) |
| C(27)-N(2)-C(30)-C(29) | 0.0(12) |
| C(27)-N(2)-C(31)-C(32) | 58.2(14) |
| C(27)-N(2)-C(31A)-C(32) | -55(3) |
| C(27)-C(28)-C(29)-O(4) | 178.6(8) |
| C(27)-C(28)-C(29)-C(30) | -1.5(10) |
| C(28)-C(20)-C(21)-C(22) | -151.1(5) |
| C(28)-C(20)-C(21)-C(26) | 36.3(7) |
| C(28)-C(29)-C(30)-O(5) | 180.0(12) |
| C(28)-C(29)-C(30)-N(2) | 1.0(11) |
| C(29)-O(4)-C(14)-C(12) | -80.6(7) |
| C(29)-O(4)-C(14)-C(15) | 163.0(6) |
| C(29)-O(4)-C(14)-C(19) | 42.7(7) |
| C(30)-N(2)-C(27)-C(28) | -0.8(12) |
| C(30)-N(2)-C(31)-C(32) | -147.1(11) |
| C(30)-N(2)-C(31A)-C(32) | 165.9(11) |
| C(31)-N(2)-C(27)-C(28) | 157.5(9) |
| C(31)-N(2)-C(30)-O(5) | 25(2) |
| C(31)-N(2)-C(30)-C(29) | -156.2(11) |
| C(31)-C(32)-C(33)-C(34) | -177.5(8) |
| C(31)-C(32)-C(37)-C(36) | 177.5(8) |
| C(32)-C(33)-C(34)-C(35) | 0.0 |
| C(33)-C(32)-C(37)-C(36) | 0.0 |
| C(33)-C(32)-C(31A)-N(2) | 124.5(19) |
| C(33)-C(34)-C(35)-C(36) | 0.0 |
| C(34)-C(35)-C(36)-C(37) | 0.0 |

| | |
|---|---|
| C(35)-C(36)-C(37)-C(32) | 0.0 |
| C(37)-C(32)-C(33)-C(34) | 0.0 |
| C(37)-C(32)-C(31A)-N(2) | -82(8) |
| Cl(2)-C(61)-C(62)-C(63) | -176.5(4) |
| O(9)-C(51)-C(52)-C(53) | -64.5(8) |
| O(9)-C(51)-C(56)-C(55) | 62.7(7) |
| O(9)-C(51)-C(56)-C(57) | -61.3(7) |
| O(9)-C(66)-C(67)-O(10) | 3.5(15) |
| O(9)-C(66)-C(67)-N(4) | -179.3(8) |
| N(4)-C(64)-C(65)-C(57) | 178.5(8) |
| N(4)-C(64)-C(65)-C(66) | -0.2(10) |
| C(38)-O(6)-C(39)-C(40) | 179.7(8) |
| C(38)-O(6)-C(39)-C(41) | 59.9(10) |
| C(38)-O(6)-C(39)-C(42) | -64.8(10) |
| C(38)-N(3)-C(43)-C(48) | -175.2(6) |
| C(38)-N(3)-C(43)-C(44) | 4.2(9) |
| C(38)-N(3)-C(50)-O(8) | -1.4(13) |
| C(38)-N(3)-C(50)-C(49) | 178.7(7) |
| C(39)-O(6)-C(38)-O(7) | 2.3(13) |
| C(39)-O(6)-C(38)-N(3) | -179.6(7) |
| C(48)-C(47)-C(46)-C(45) | 0.0 |
| C(48)-C(49)-C(50)-O(8) | 173.1(7) |
| C(48)-C(49)-C(50)-N(3) | -7.0(7) |
| C(48)-C(49)-C(51)-O(9) | -63.2(7) |
| C(48)-C(49)-C(51)-C(52) | 50.8(8) |
| C(48)-C(49)-C(51)-C(56) | 176.7(5) |
| C(47)-C(48)-C(43)-N(3) | 179.5(5) |
| C(47)-C(48)-C(43)-C(44) | 0.0 |
| C(47)-C(48)-C(49)-C(50) | -176.6(4) |
| C(47)-C(48)-C(49)-C(51) | 61.2(7) |
| C(47)-C(46)-C(45)-C(44) | 0.0 |
| C(46)-C(45)-C(44)-C(43) | 0.0 |
| C(45)-C(44)-C(43)-N(3) | -179.4(6) |
| C(45)-C(44)-C(43)-C(48) | 0.0 |
| C(43)-N(3)-C(38)-O(6) | -170.0(6) |
| C(43)-N(3)-C(38)-O(7) | 8.2(12) |
| C(43)-N(3)-C(50)-O(8) | -174.3(7) |
| C(43)-N(3)-C(50)-C(49) | 5.8(7) |

| | |
|---|---|
| C(43)-C(48)-C(47)-C(46) | 0.0 |
| C(43)-C(48)-C(49)-C(50) | 6.1(6) |
| C(43)-C(48)-C(49)-C(51) | -116.0(5) |
| C(49)-C(48)-C(47)-C(46) | -177.0(6) |
| C(49)-C(48)-C(43)-N(3) | -2.9(5) |
| C(49)-C(48)-C(43)-C(44) | 177.6(5) |
| C(49)-C(51)-C(52)-C(53) | -178.6(6) |
| C(49)-C(51)-C(56)-C(55) | -179.3(6) |
| C(49)-C(51)-C(56)-C(57) | 56.7(8) |
| C(50)-N(3)-C(38)-O(6) | 17.9(11) |
| C(50)-N(3)-C(38)-O(7) | -163.9(8) |
| C(50)-N(3)-C(43)-C(48) | -2.0(6) |
| C(50)-N(3)-C(43)-C(44) | 177.5(5) |
| C(50)-C(49)-C(51)-O(9) | 179.7(5) |
| C(50)-C(49)-C(51)-C(52) | -66.3(7) |
| C(50)-C(49)-C(51)-C(56) | 59.6(7) |
| C(51)-O(9)-C(66)-C(65) | -16.5(11) |
| C(51)-O(9)-C(66)-C(67) | 158.7(7) |
| C(51)-C(49)-C(50)-O(8) | -62.9(9) |
| C(51)-C(49)-C(50)-N(3) | 117.0(6) |
| C(51)-C(52)-C(53)-C(54) | -54.4(9) |
| C(51)-C(56)-C(57)-C(58) | 171.8(5) |
| C(51)-C(56)-C(57)-C(65) | 42.4(8) |
| C(52)-C(51)-C(56)-C(55) | -53.4(8) |
| C(52)-C(51)-C(56)-C(57) | -177.5(6) |
| C(52)-C(53)-C(54)-C(55) | 55.3(8) |
| C(53)-C(54)-C(55)-C(56) | -58.7(9) |
| C(54)-C(55)-C(56)-C(51) | 57.5(8) |
| C(54)-C(55)-C(56)-C(57) | -178.6(6) |
| C(55)-C(56)-C(57)-C(58) | 48.8(8) |
| C(55)-C(56)-C(57)-C(65) | -80.6(7) |
| C(56)-C(51)-C(52)-C(53) | 53.9(8) |
| C(56)-C(57)-C(58)-C(59) | 83.1(6) |
| C(56)-C(57)-C(58)-C(63) | -88.0(6) |
| C(56)-C(57)-C(65)-C(64) | 168.9(8) |
| C(56)-C(57)-C(65)-C(66) | -12.5(10) |
| C(57)-C(65)-C(66)-O(9) | -1.3(13) |
| C(57)-C(65)-C(66)-C(67) | -176.7(7) |

| | |
|---|---|
| C(59)-C(60)-C(61)-Cl(2) | 176.5(4) |
| C(59)-C(60)-C(61)-C(62) | 0.0 |
| C(60)-C(59)-C(58)-C(57) | -171.1(5) |
| C(60)-C(59)-C(58)-C(63) | 0.0 |
| C(60)-C(61)-C(62)-C(63) | 0.0 |
| C(61)-C(62)-C(63)-C(58) | 0.0 |
| C(62)-C(63)-C(58)-C(57) | 170.9(5) |
| C(62)-C(63)-C(58)-C(59) | 0.0 |
| C(58)-C(57)-C(65)-C(64) | 42.3(11) |
| C(58)-C(57)-C(65)-C(66) | -139.1(7) |
| C(58)-C(59)-C(60)-C(61) | 0.0 |
| C(64)-N(4)-C(67)-O(10) | -179.6(11) |
| C(64)-N(4)-C(67)-C(66) | 3.1(11) |
| C(64)-N(4)-C(75)-C(69) | -30.1(17) |
| C(64)-N(4)-C(75A)-C(69) | 67(2) |
| C(64)-C(65)-C(66)-O(9) | 177.6(8) |
| C(64)-C(65)-C(66)-C(67) | 2.2(10) |
| C(65)-C(57)-C(58)-C(59) | -152.6(5) |
| C(65)-C(57)-C(58)-C(63) | 36.4(7) |
| C(65)-C(66)-C(67)-O(10) | 179.4(11) |
| C(65)-C(66)-C(67)-N(4) | -3.3(11) |
| C(66)-O(9)-C(51)-C(49) | -76.7(7) |
| C(66)-O(9)-C(51)-C(52) | 165.7(6) |
| C(66)-O(9)-C(51)-C(56) | 46.3(7) |
| C(67)-N(4)-C(64)-C(65) | -2.0(11) |
| C(67)-N(4)-C(75)-C(69) | 170.9(9) |
| C(67)-N(4)-C(75A)-C(69) | -149.9(13) |
| C(69)-C(70)-C(71)-C(72) | 0.0 |
| C(70)-C(69)-C(74)-C(73) | 0.0 |
| C(70)-C(69)-C(75)-N(4) | 112.3(9) |
| C(70)-C(69)-C(75A)-N(4) | 29(5) |
| C(70)-C(71)-C(72)-C(73) | 0.0 |
| C(71)-C(72)-C(73)-C(74) | 0.0 |
| C(72)-C(73)-C(74)-C(69) | 0.0 |
| C(74)-C(69)-C(70)-C(71) | 0.0 |
| C(74)-C(69)-C(75)-N(4) | -72.8(11) |
| C(74)-C(69)-C(75A)-N(4) | -138(2) |
| C(75)-N(4)-C(64)-C(65) | -161.6(11) |

| | |
|---|---|
| C(75)-N(4)-C(67)-O(10) | -17.4(17) |
| C(75)-N(4)-C(67)-C(66) | 165.3(9) |
| C(75)-C(69)-C(70)-C(71) | 175.0(7) |
| C(75)-C(69)-C(74)-C(73) | -174.8(8) |
| C(75A)-N(4)-C(64)-C(65) | 143.8(17) |
| C(75A)-N(4)-C(67)-O(10) | 36(2) |
| C(75A)-N(4)-C(67)-C(66) | -141.4(19) |
| C(75A)-C(69)-C(70)-C(71) | -165(4) |
| C(75A)-C(69)-C(74)-C(73) | 175.0(13) |
| C(31A)-N(2)-C(27)-C(28) | -140(2) |
| C(31A)-N(2)-C(30)-O(5) | -32(2) |
| C(31A)-N(2)-C(30)-C(29) | 147.0(17) |
| C(31A)-C(32)-C(33)-C(34) | 174.4(15) |
| C(31A)-C(32)-C(37)-C(36) | -152(7) |

Symmetry transformations used to generate equivalent atoms:

## 7. Copies of NMR Spectra of Products

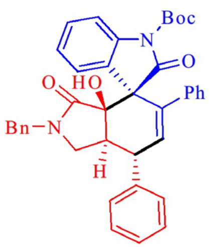

**3aa**

($^1$H NMR, 400MHz, $CDCl_3$)

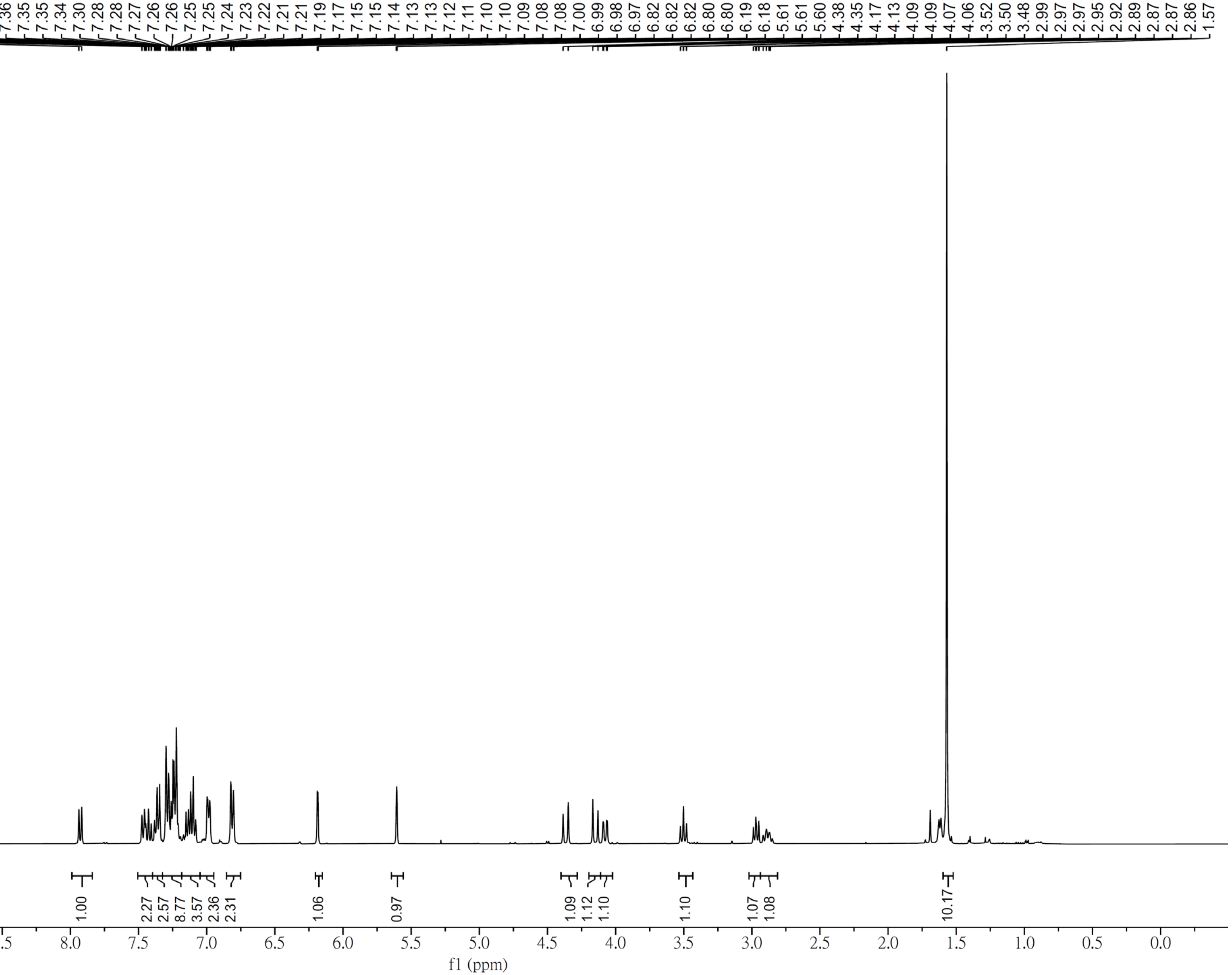

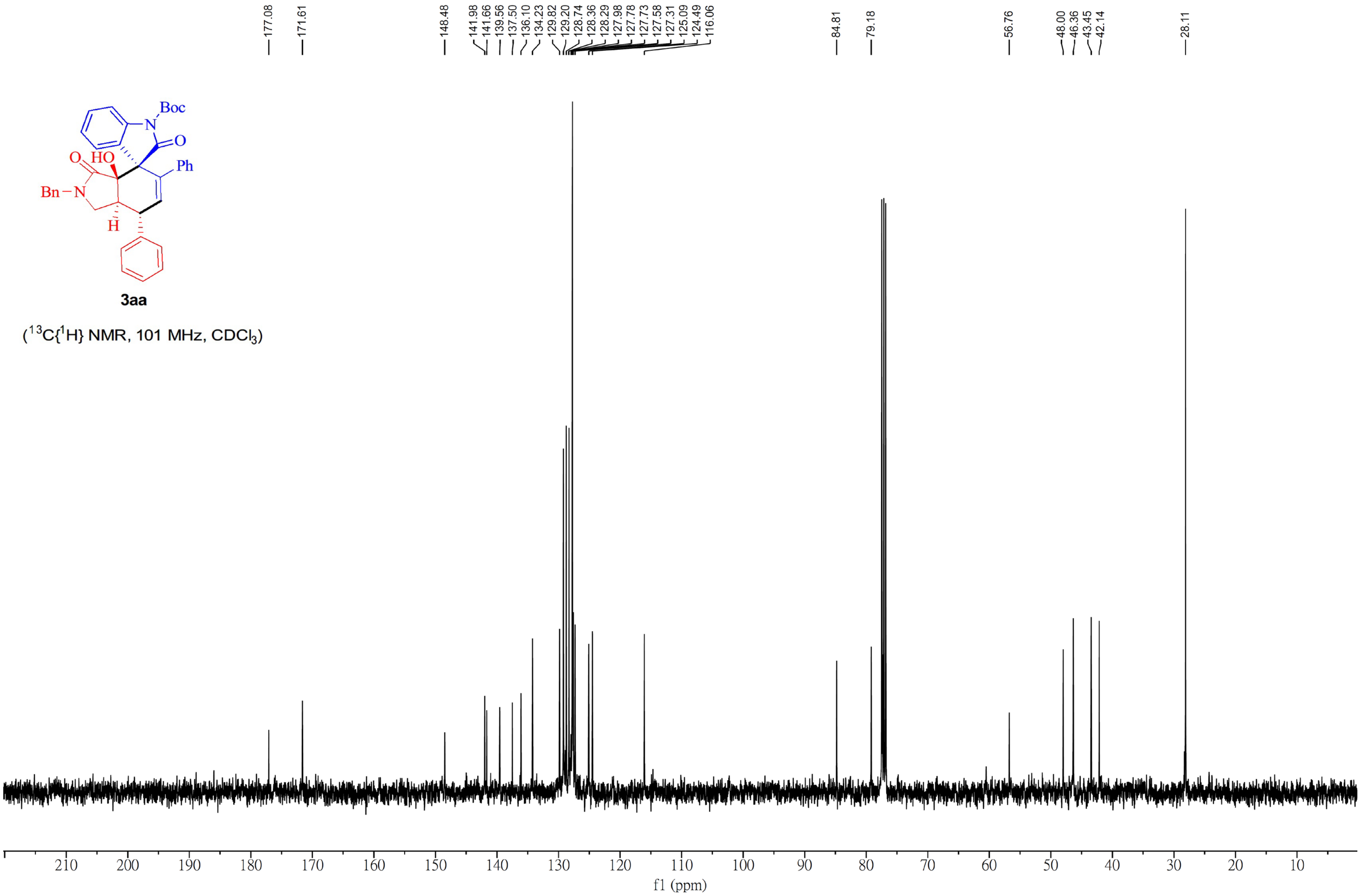
177.08
171.61
148.48
141.98
141.66
139.56
137.50
136.10
134.23
129.82
129.20
128.74
128.36
128.29
127.98
127.78
127.73
127.58
127.31
125.09
124.49
116.06
84.81
79.18
56.76
48.00
46.36
43.45
42.14
28.11
Boc
N
O
HO
O
Ph
Bn
N
H
3aa
($^{13}C\{^1H\}$ NMR, 101 MHz, $CDCl_3$)
210
200
190
180
170
160
150
140
130
120
110
100
90
80
70
60
50
40
30
20
10
f1 (ppm)

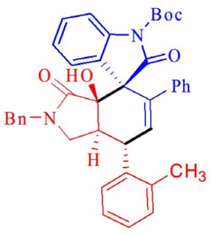

**3ab**

($^1$H NMR, 400MHz, CDCl$_3$)

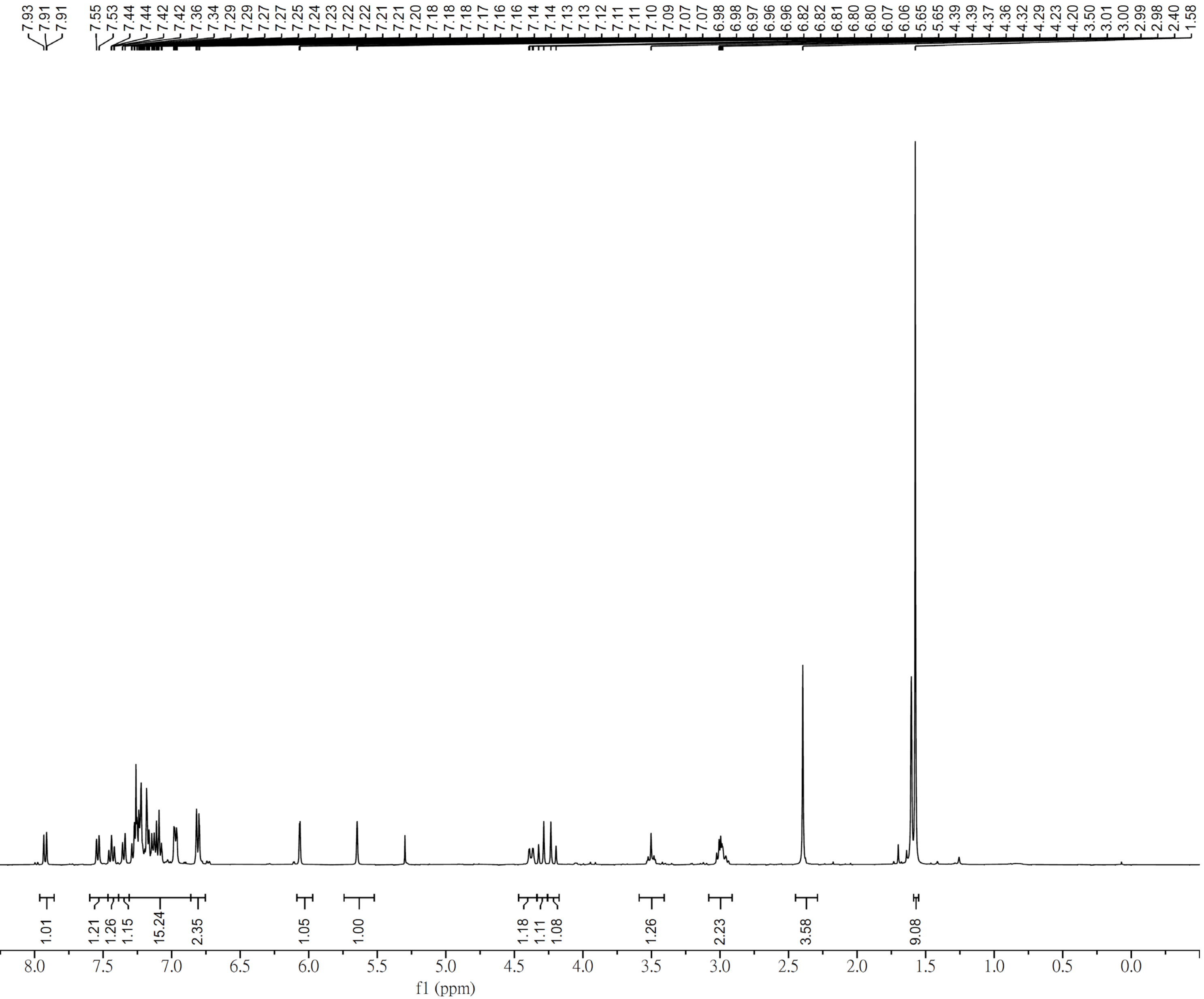

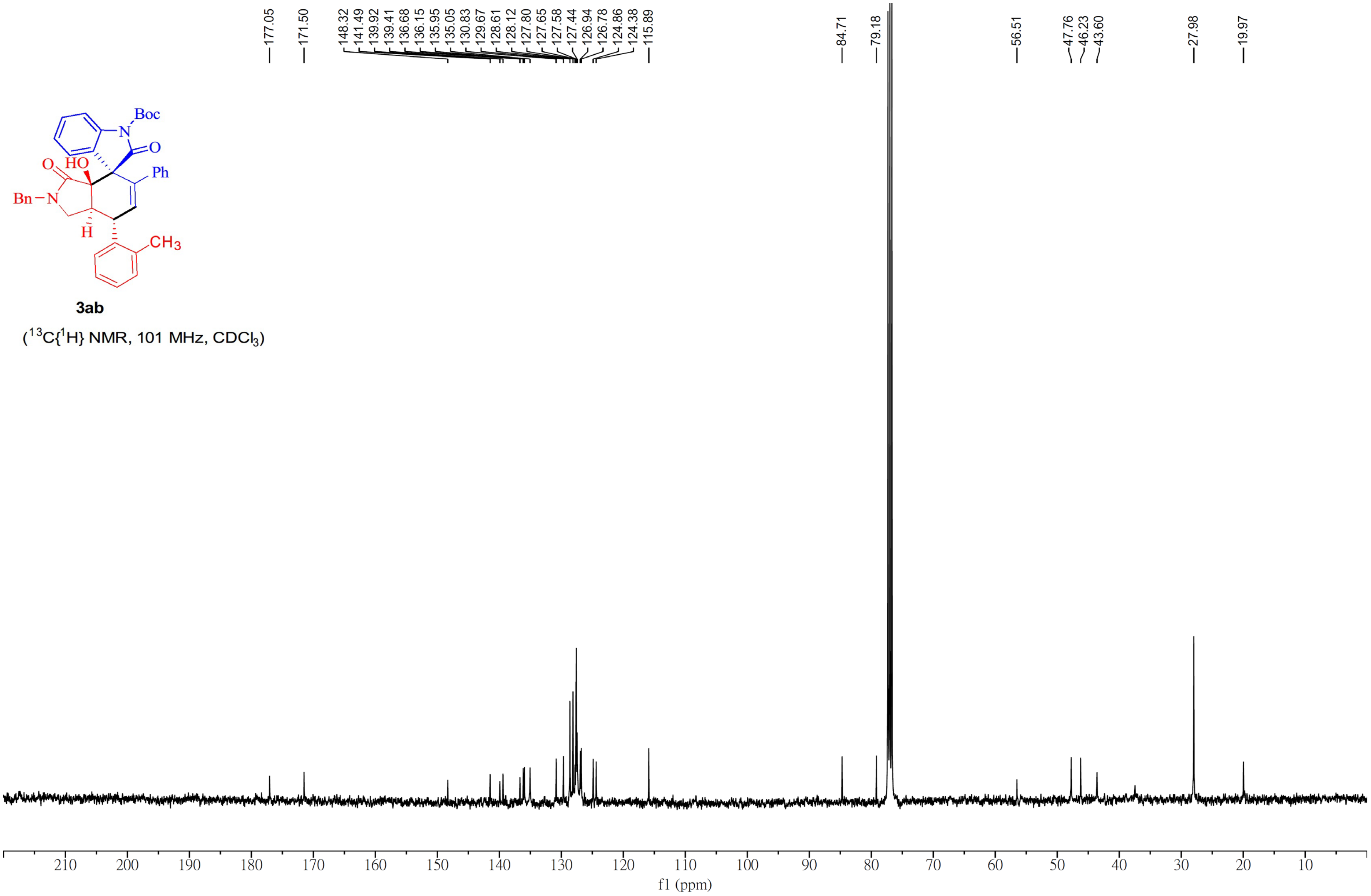


**3ab**

($^{13}C\{^{1}H\}$ NMR, 101 MHz, $CDCl_3$)

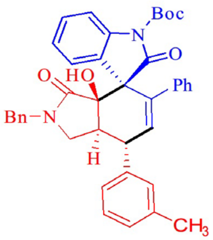


**3ac**

($^1$H NMR, 400MHz, $CDCl_3$)

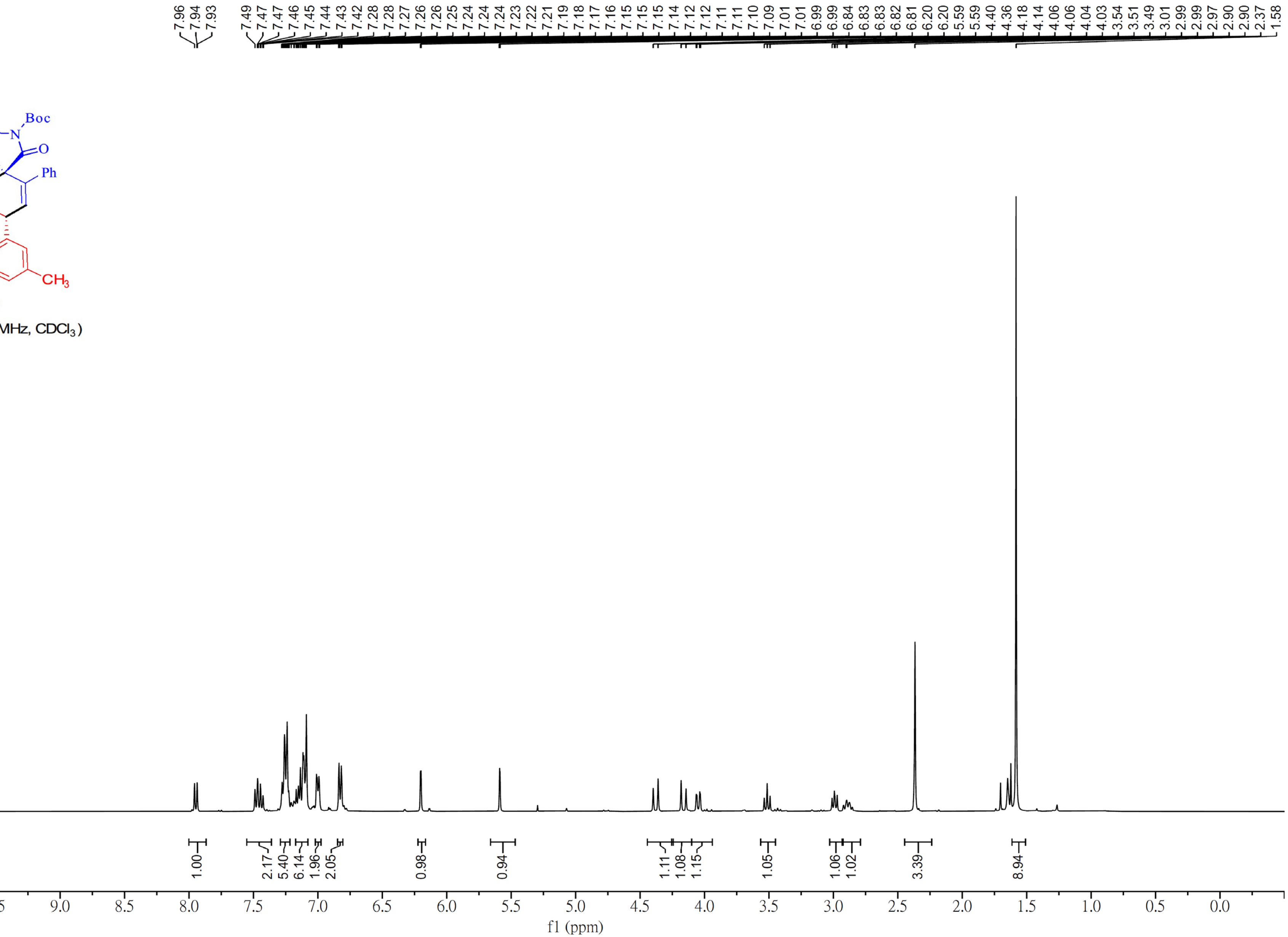

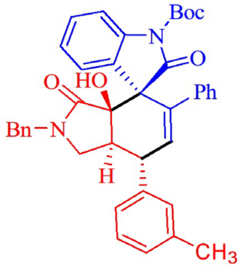


**3ac**

($^{13}C\{^{1}H\}$ NMR, 101 MHz, $CDCl_3$)

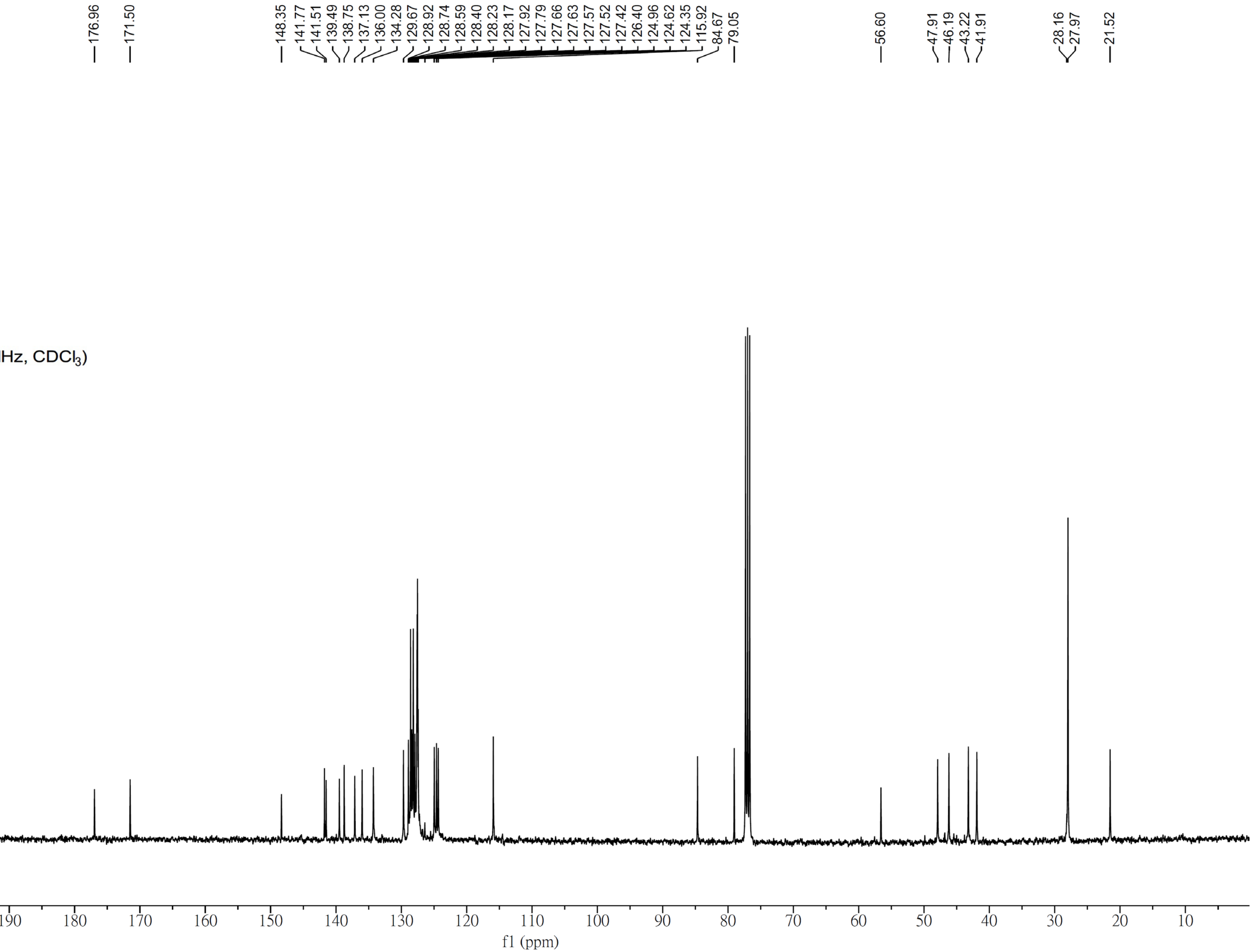

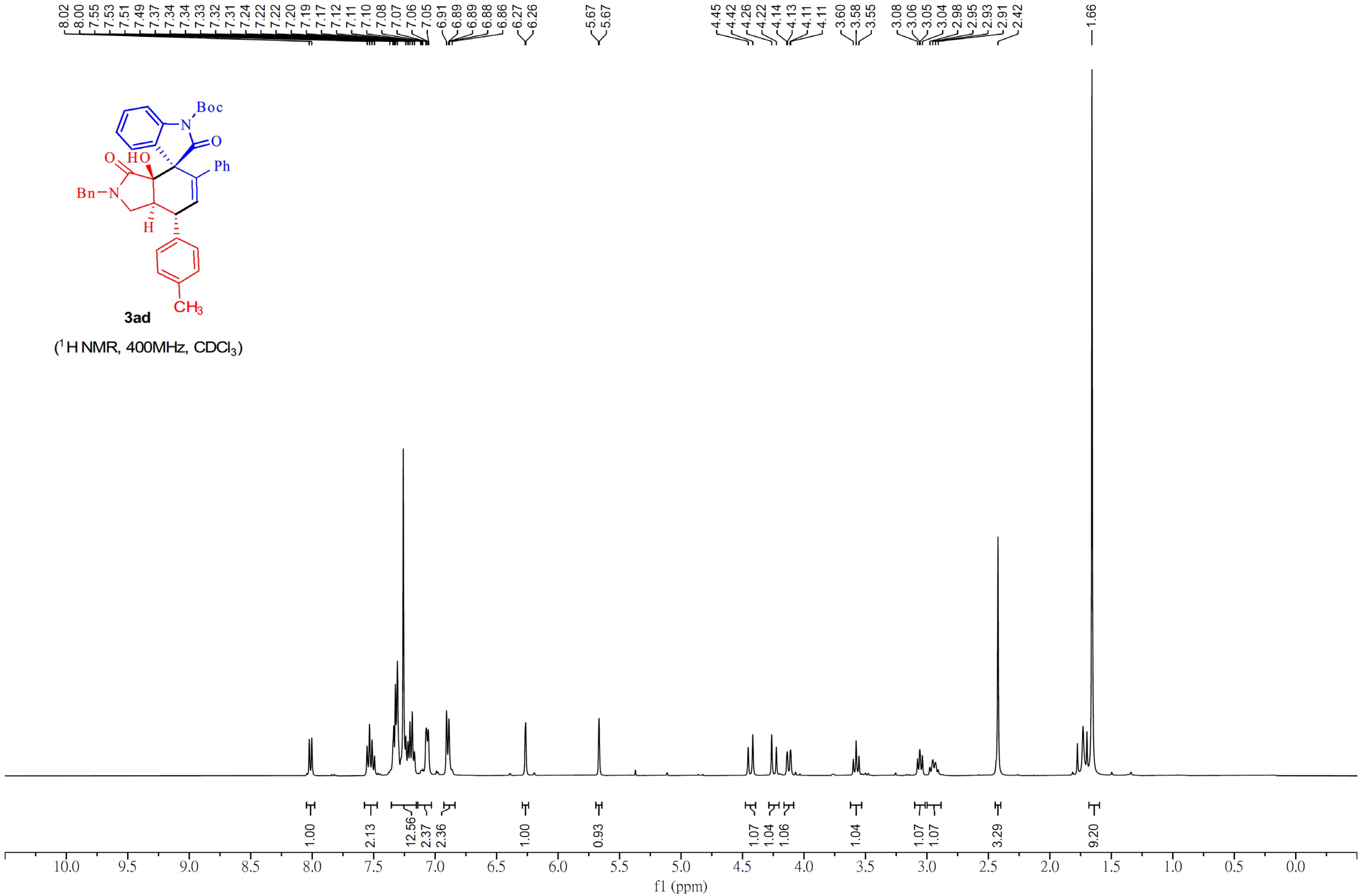
8.02
8.00
7.55
7.53
7.51
7.49
7.37
7.34
7.34
7.33
7.32
7.31
7.24
7.22
7.22
7.20
7.19
7.17
7.12
7.11
7.10
7.08
7.07
7.06
7.05
6.91
6.89
6.89
6.88
6.86
6.27
6.26
5.67
5.67
4.45
4.42
4.26
4.22
4.14
4.13
4.11
4.11
3.60
3.58
3.55
3.08
3.06
3.05
3.04
2.98
2.95
2.93
2.91
2.42
1.66
Boc
N
O
HO
O
Ph
Bn
N
H
CH3
3ad
(1H NMR, 400MHz, CDCl3)
1.00
2.13
12.56
2.37
2.36
1.00
0.93
1.07
1.04
1.06
1.04
1.07
1.07
3.29
9.20
10.0
9.5
9.0
8.5
8.0
7.5
7.0
6.5
6.0
5.5
5.0
4.5
4.0
3.5
3.0
2.5
2.0
1.5
1.0
0.5
0.0
f1 (ppm)

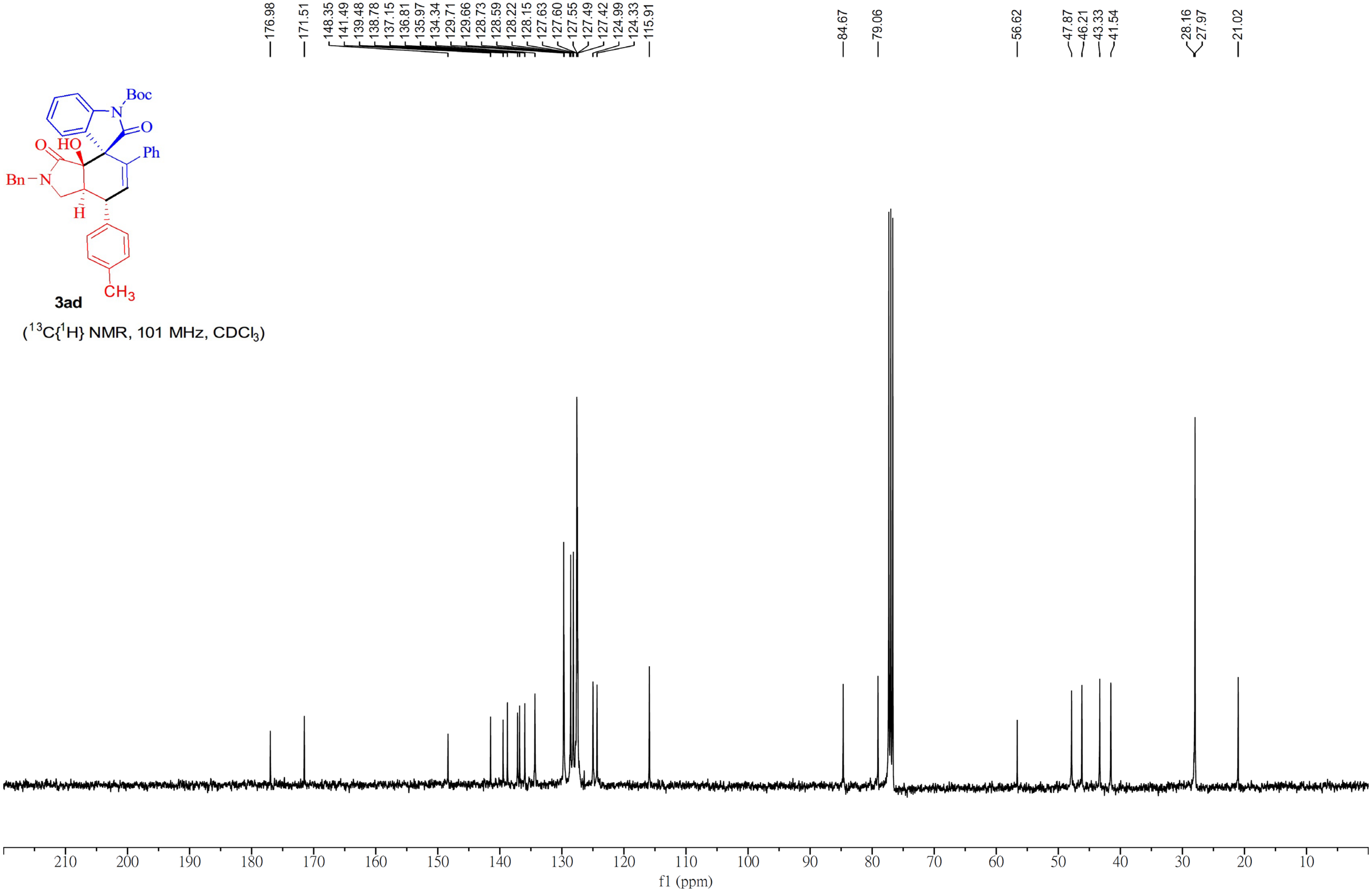
176.98
171.51
148.35
141.49
139.48
138.78
137.15
136.81
135.97
134.34
129.71
129.66
128.73
128.59
128.22
128.15
127.63
127.60
127.55
127.49
127.42
124.99
124.33
115.91
84.67
79.06
56.62
47.87
46.21
43.33
41.54
28.16
27.97
21.02
Boc
N
O
HO
O
Ph
Bn−N
H
$CH_3$
3ad
($^{13}C\{^1H\}$ NMR, 101 MHz, $CDCl_3$)
210
200
190
180
170
160
150
140
130
120
110
100
90
80
70
60
50
40
30
20
10
f1 (ppm)

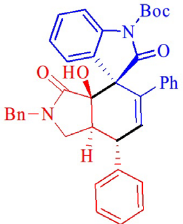


**3ae**

($^1$H NMR, 400MHz, $CDCl_3$)

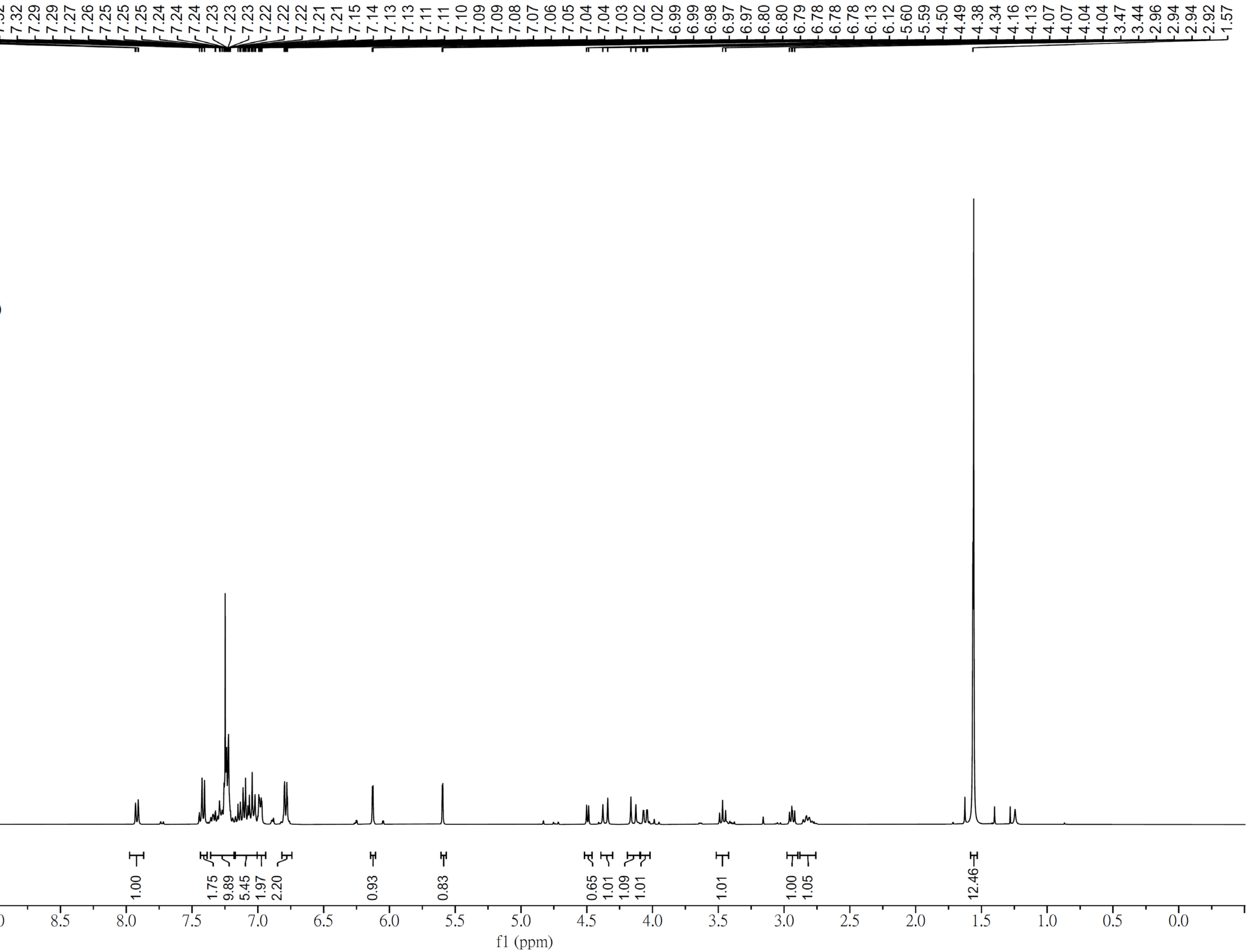

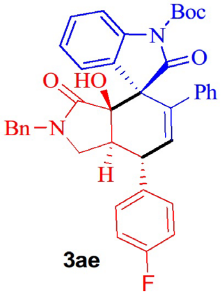


**3ae**

($^{13}C\{^1H\}$ NMR, 101 MHz, $CDCl_3$)

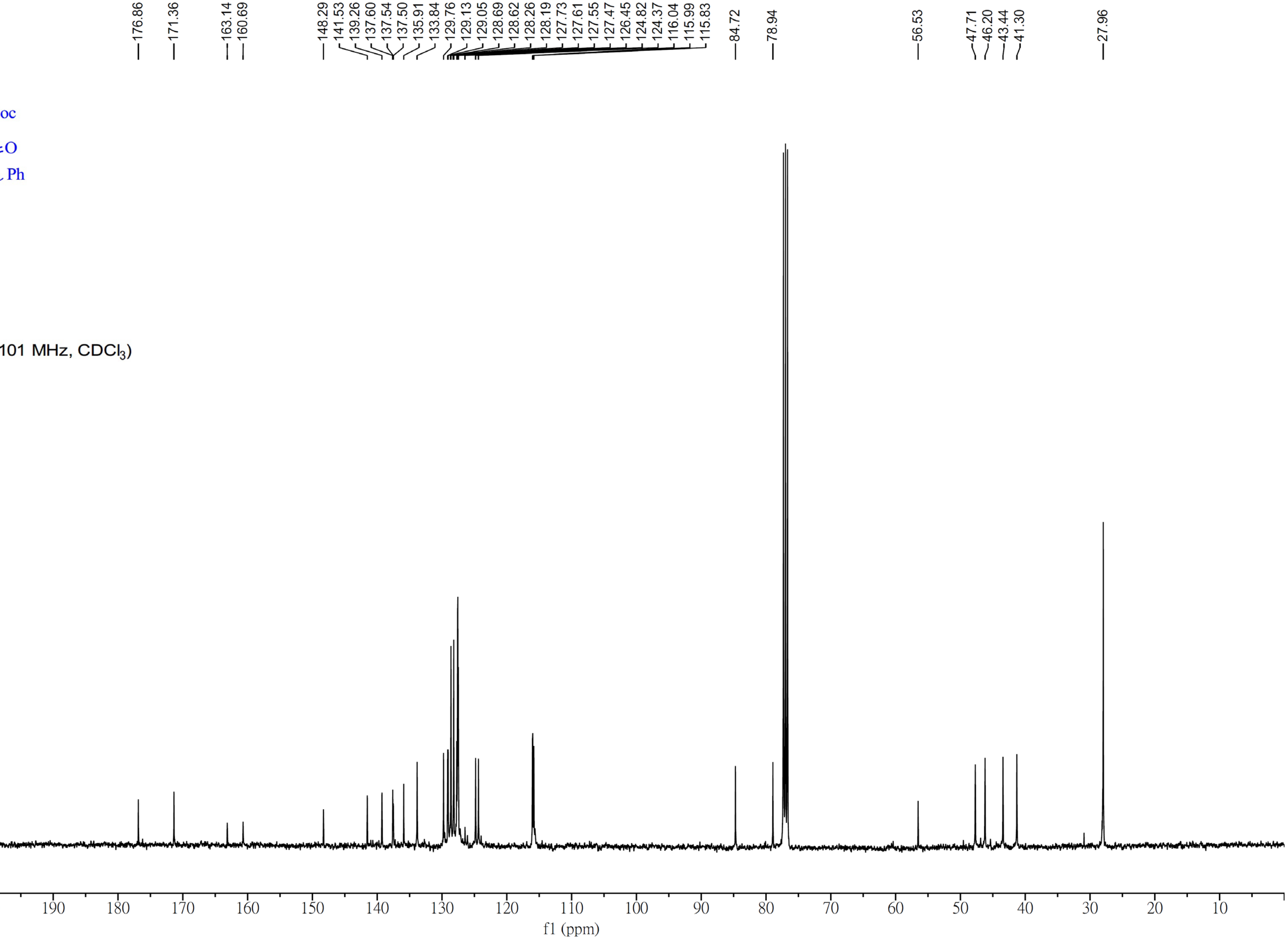

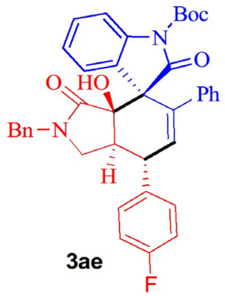


3ae

($^{19}$F NMR, 376MHz, $CDCl_3$)

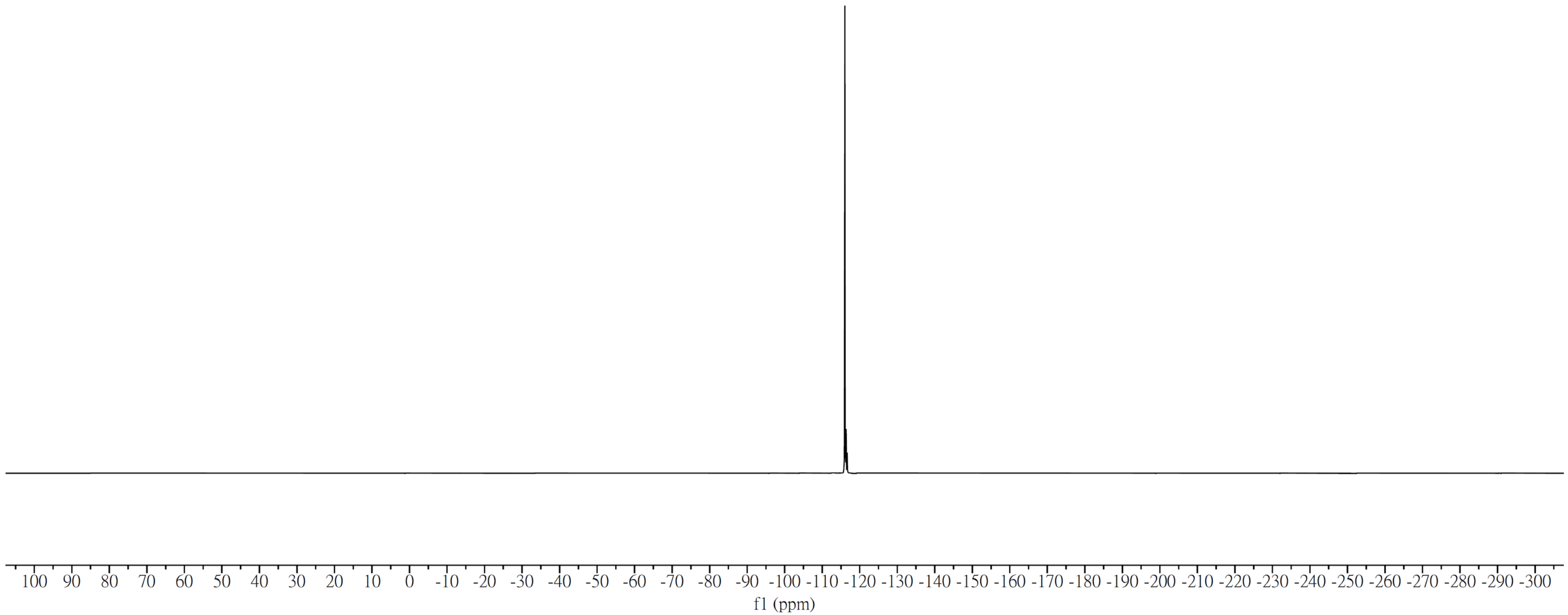

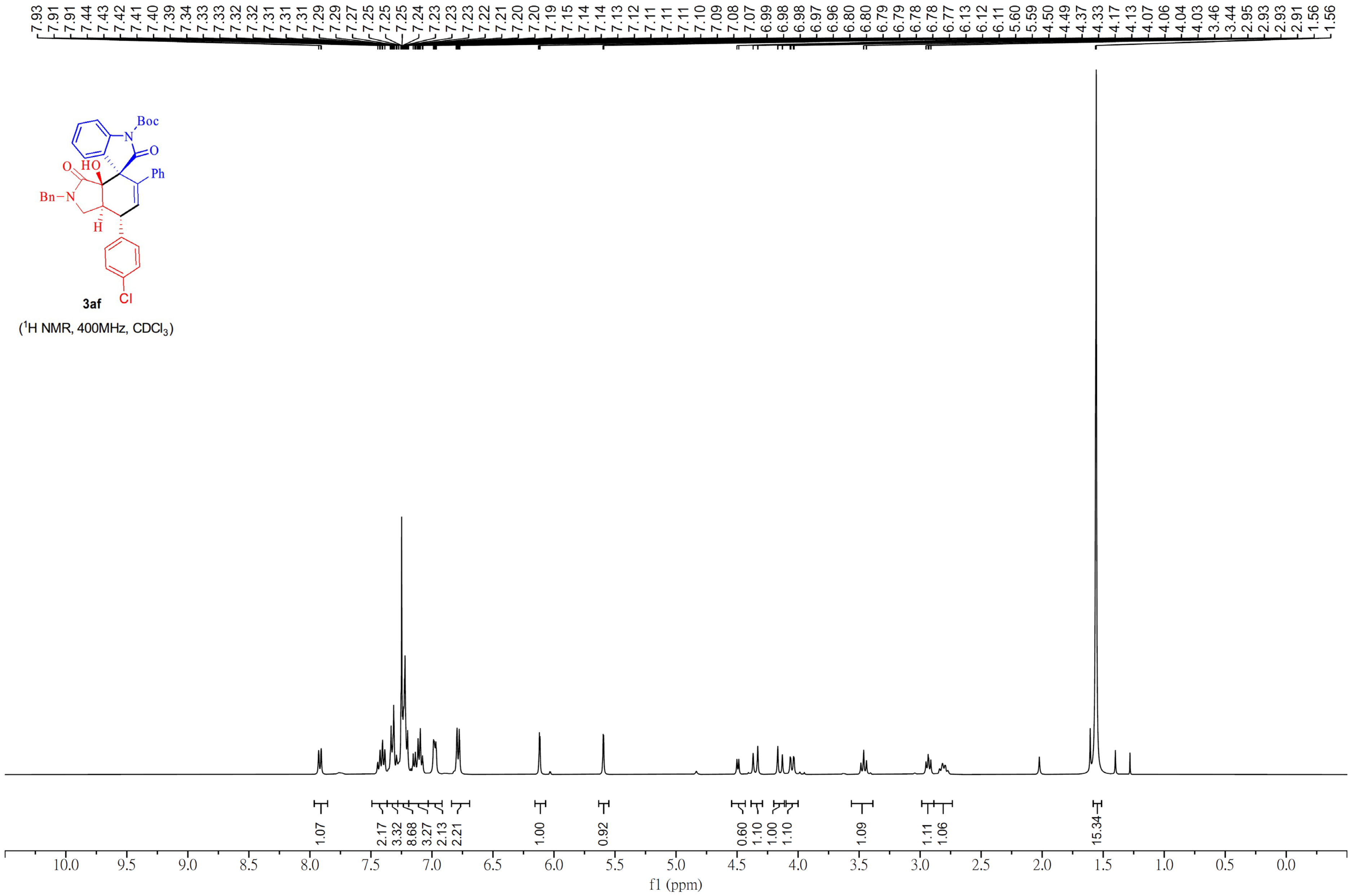


**3af**

($^1$H NMR, 400MHz, $CDCl_3$)

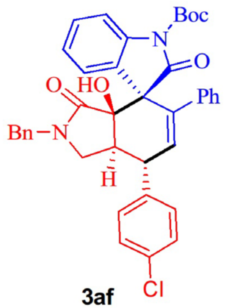


**3af**

($^{13}C\{^{1}H\}$ NMR, 101 MHz, $CDCl_3$)

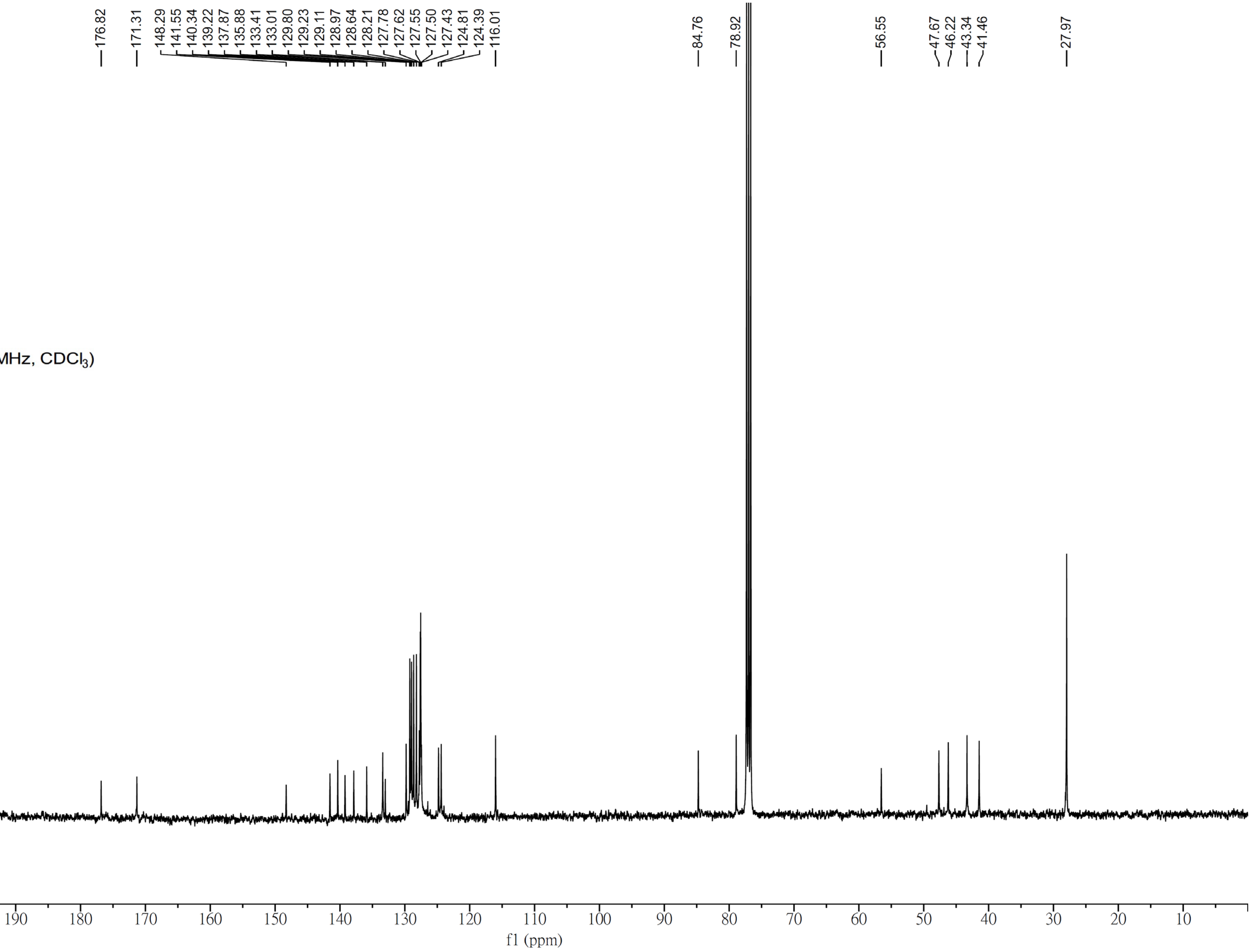

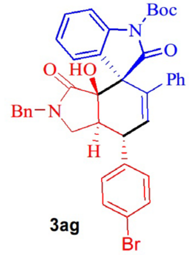

3ag

($^{1}$H NMR, 400MHz, $CDCl_3$)

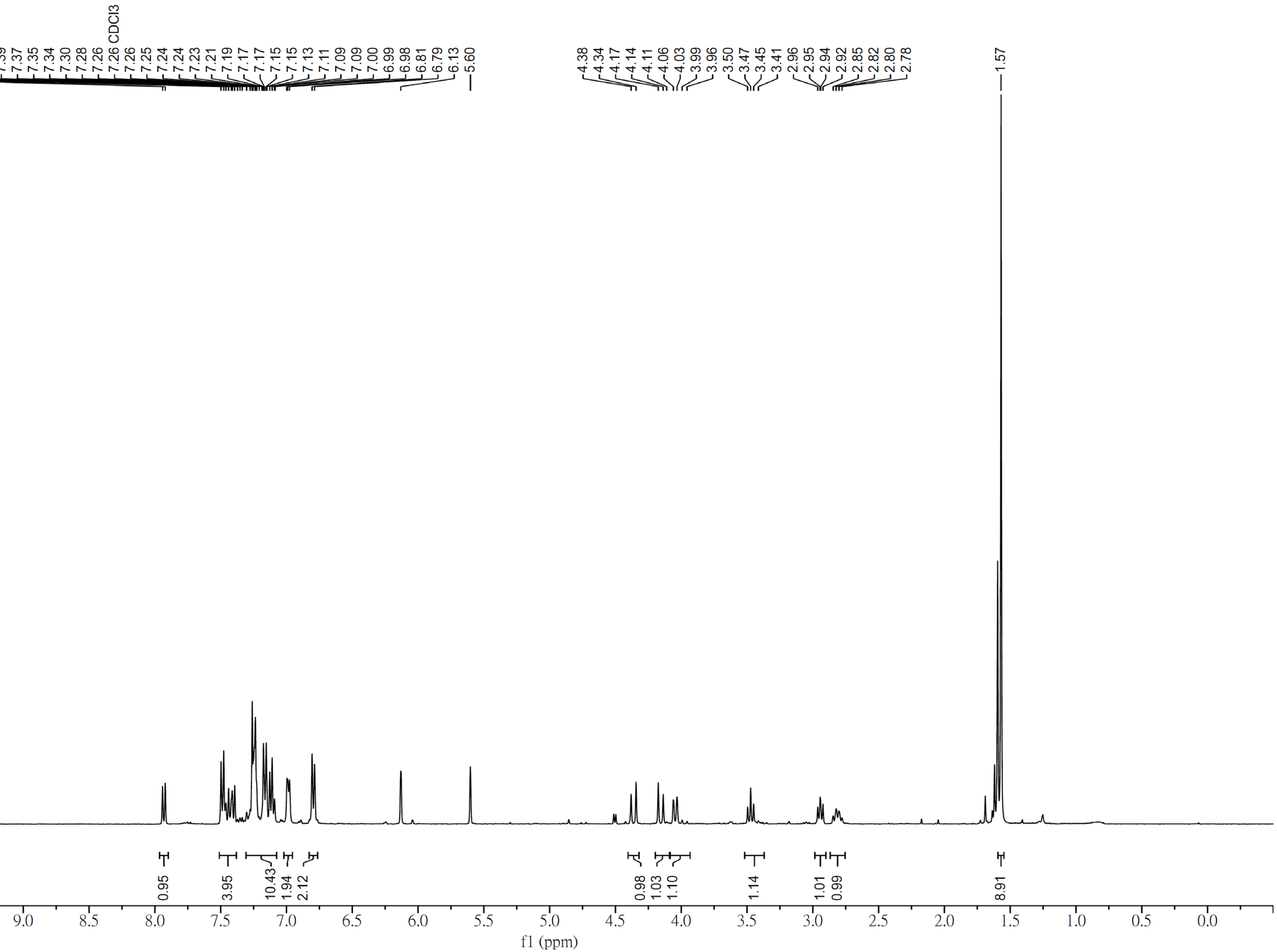

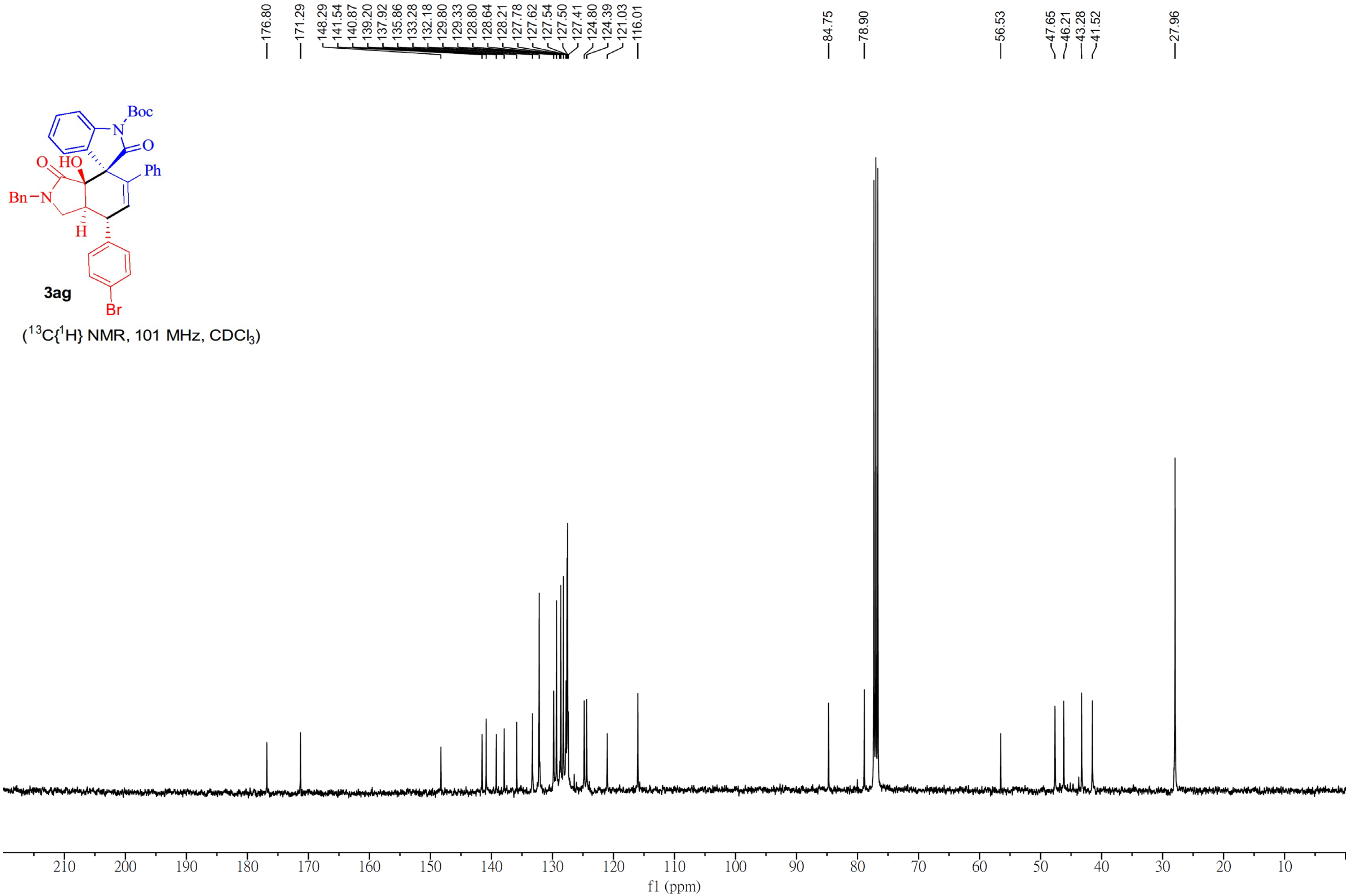


**3ag**

($^{13}C\{^{1}H\}$ NMR, 101 MHz, $CDCl_3$)

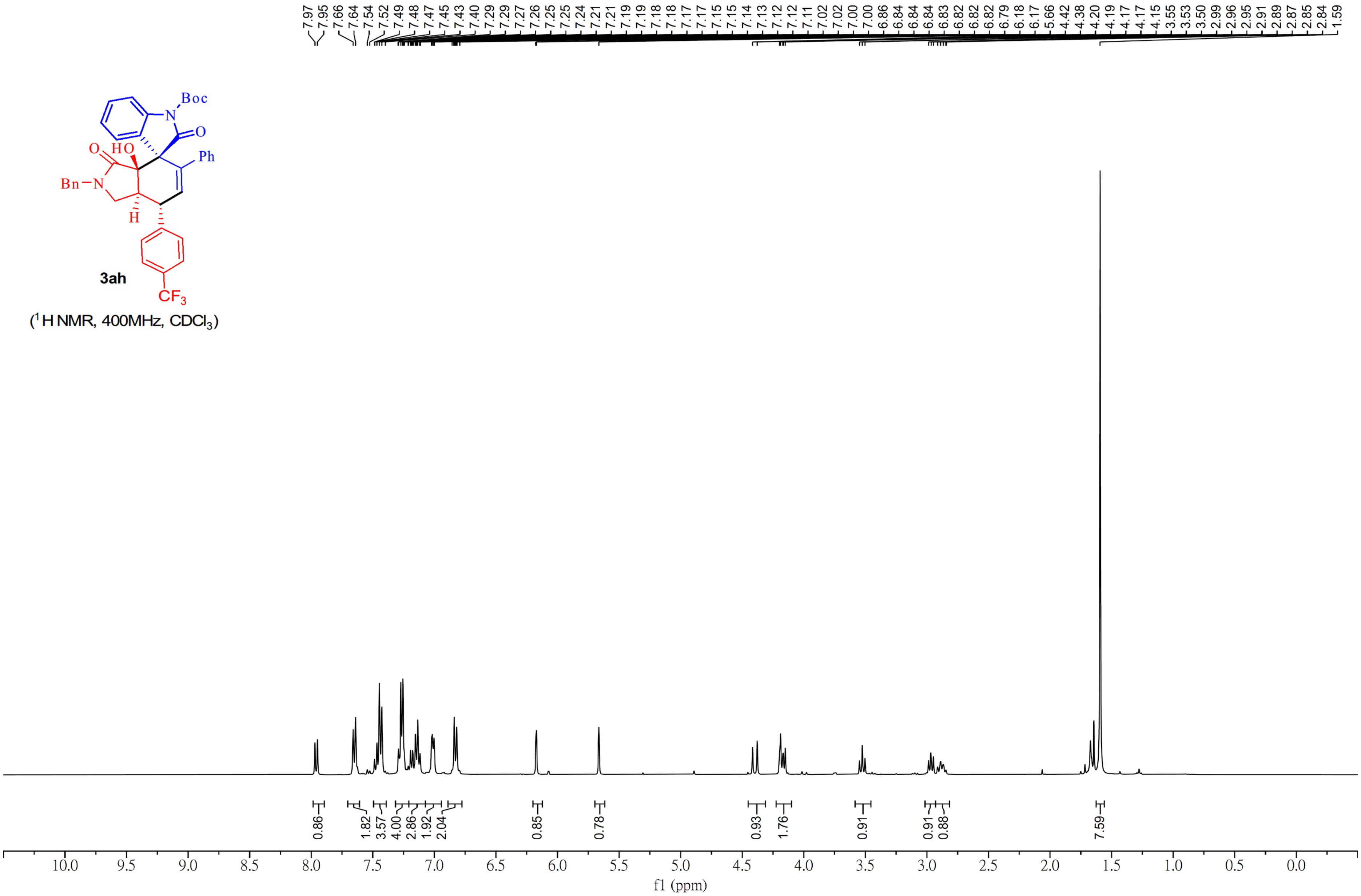

Boc
N
O
HO
O
Ph
Bn
N
H
CF3
3ah
(1H NMR, 400MHz, CDCl3)
f1 (ppm)

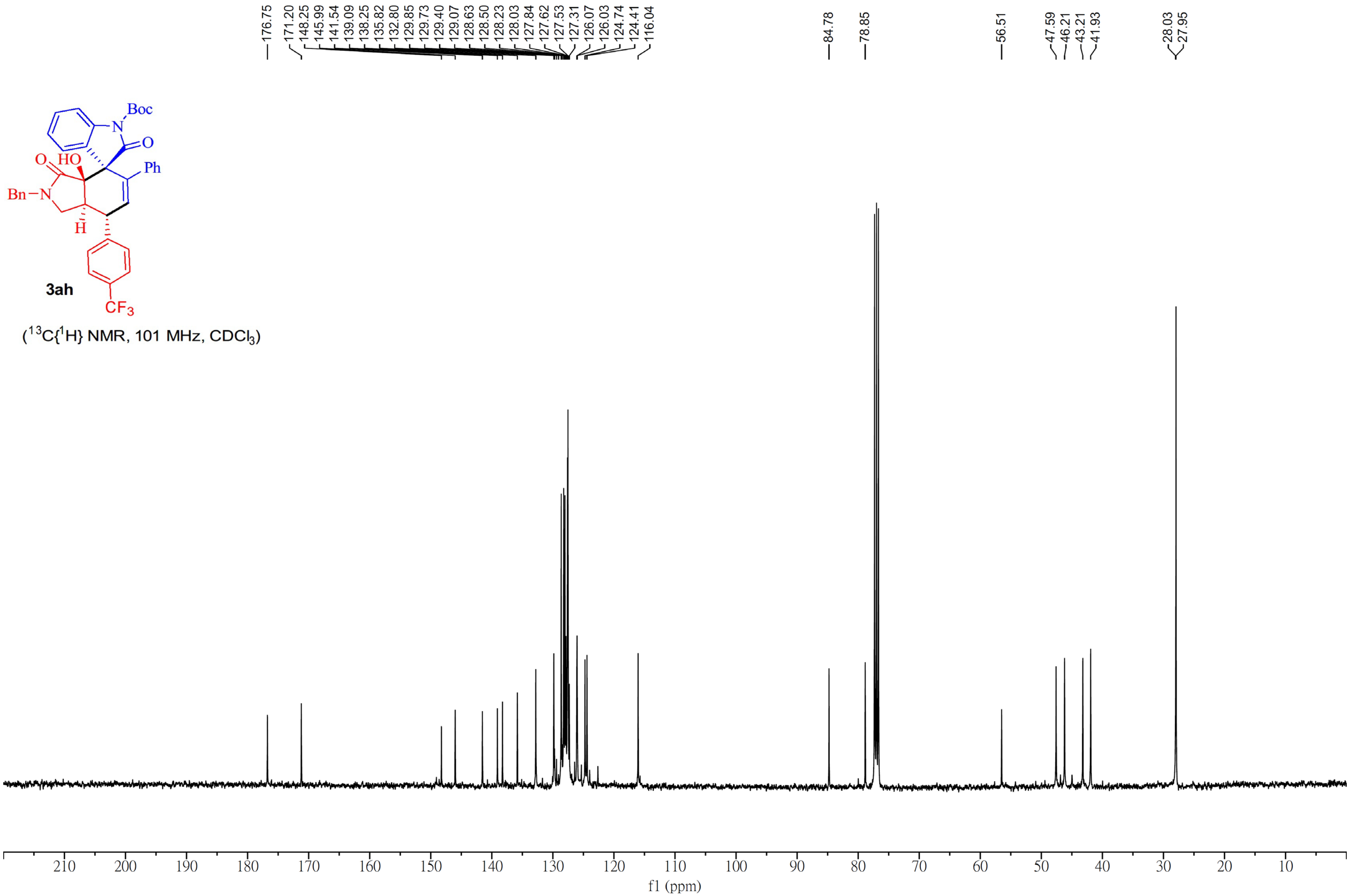


**3ah**

($^{13}C\{^1H\}$ NMR, 101 MHz, $CDCl_3$)

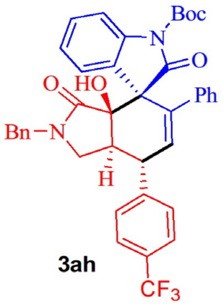


**3ah**

($^{19}$F NMR, 376MHz, $CDCl_3$)

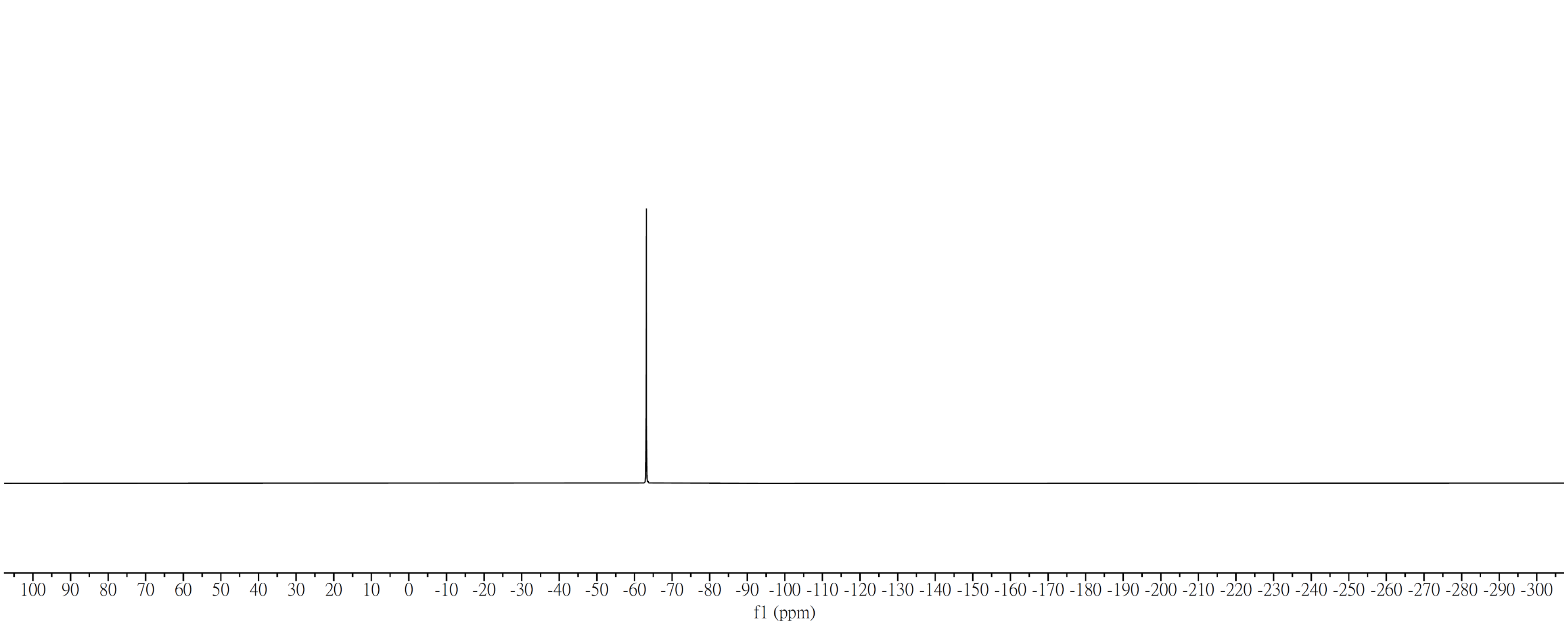

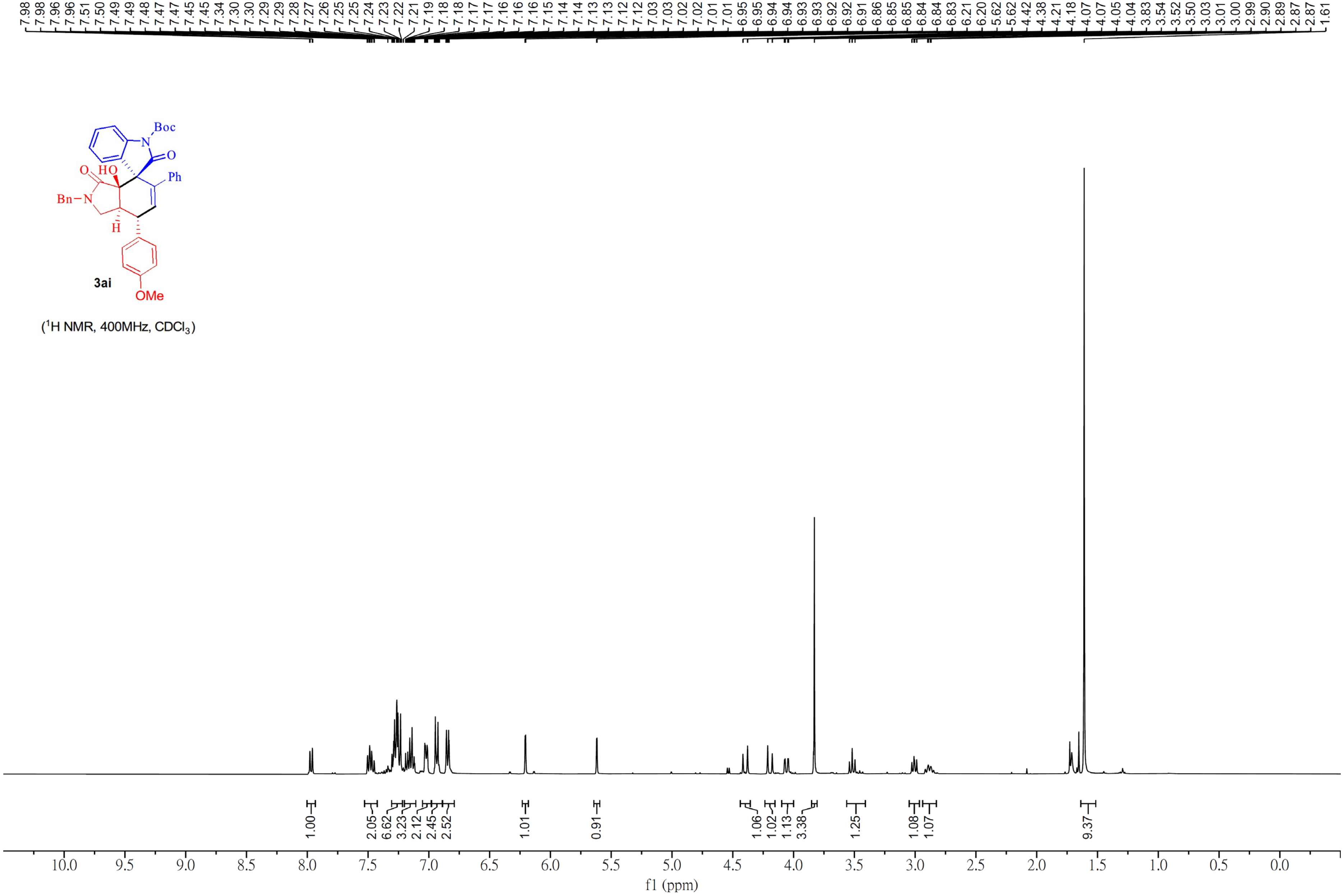
7.98 7.98 7.96 7.96 7.51 7.50 7.49 7.49 7.48 7.47 7.45 7.45 7.34 7.30 7.30 7.29 7.29 7.28 7.27 7.26 7.25 7.25 7.24 7.23 7.22 7.21 7.19 7.18 7.18 7.17 7.16 7.16 7.15 7.14 7.14 7.13 7.13 7.12 7.12 7.03 7.03 7.02 7.02 7.01 7.01 6.95 6.95 6.94 6.94 6.93 6.93 6.92 6.92 6.91 6.86 6.85 6.85 6.84 6.84 6.83 6.21 6.20 5.62 5.62 4.42 4.38 4.21 4.18 4.07 4.07 4.05 4.04 3.83 3.54 3.52 3.50 3.03 3.01 3.00 2.99 2.90 2.89 2.87 2.87 1.61
Boc
N
O
HO
O
Ph
Bn
N
H
OMe
3ai
(¹H NMR, 400MHz, CDCl₃)
1.00 2.05 6.62 3.23 2.12 2.45 2.52 1.01 0.91 1.06 1.02 1.13 3.38 1.25 1.08 1.07 9.37
10.0 9.5 9.0 8.5 8.0 7.5 7.0 6.5 6.0 5.5 5.0 4.5 4.0 3.5 3.0 2.5 2.0 1.5 1.0 0.5 0.0
f1 (ppm)

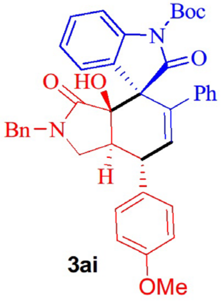


**3ai**

($^{13}C\{^{1}H\}$ NMR, 101 MHz, $CDCl_3$)

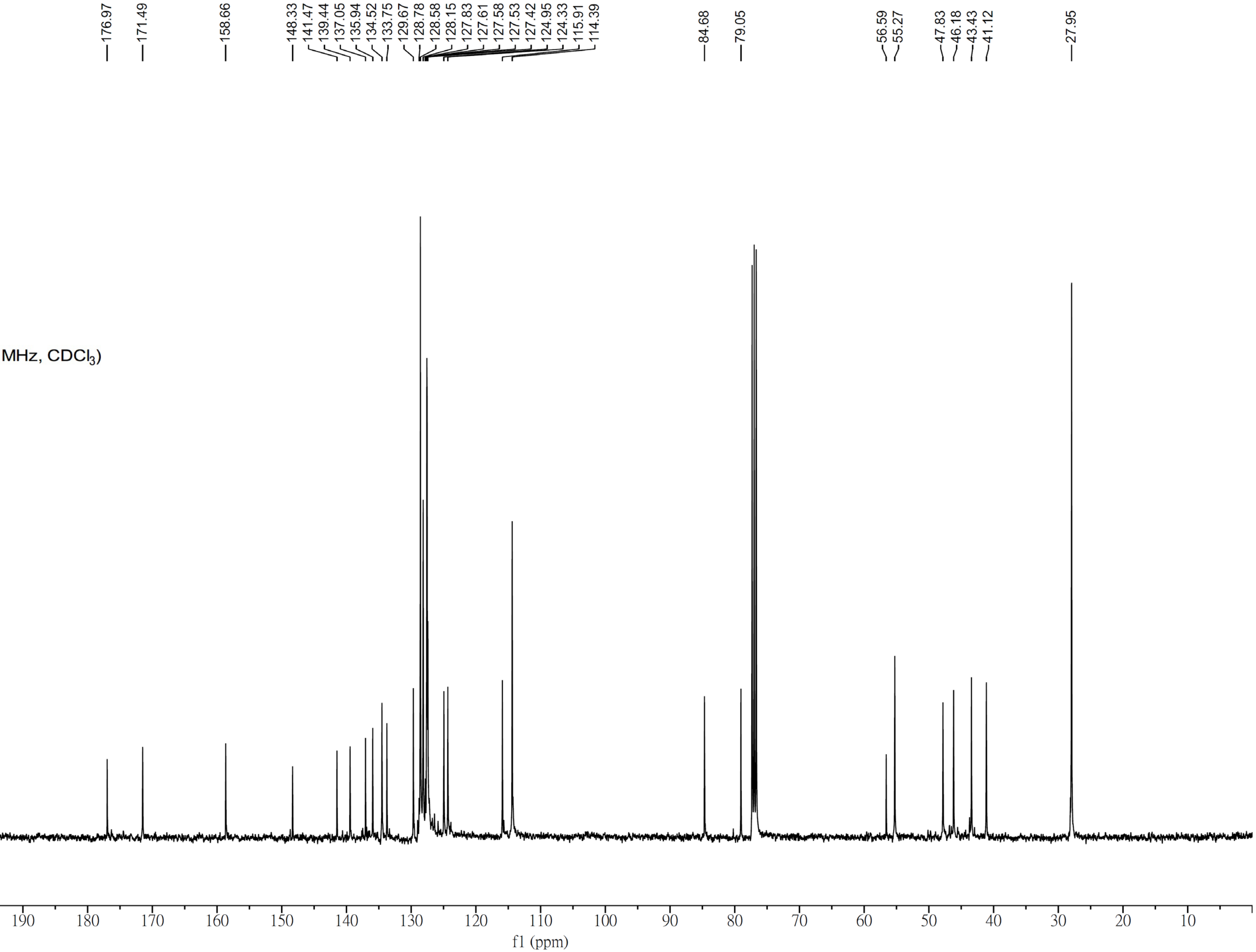

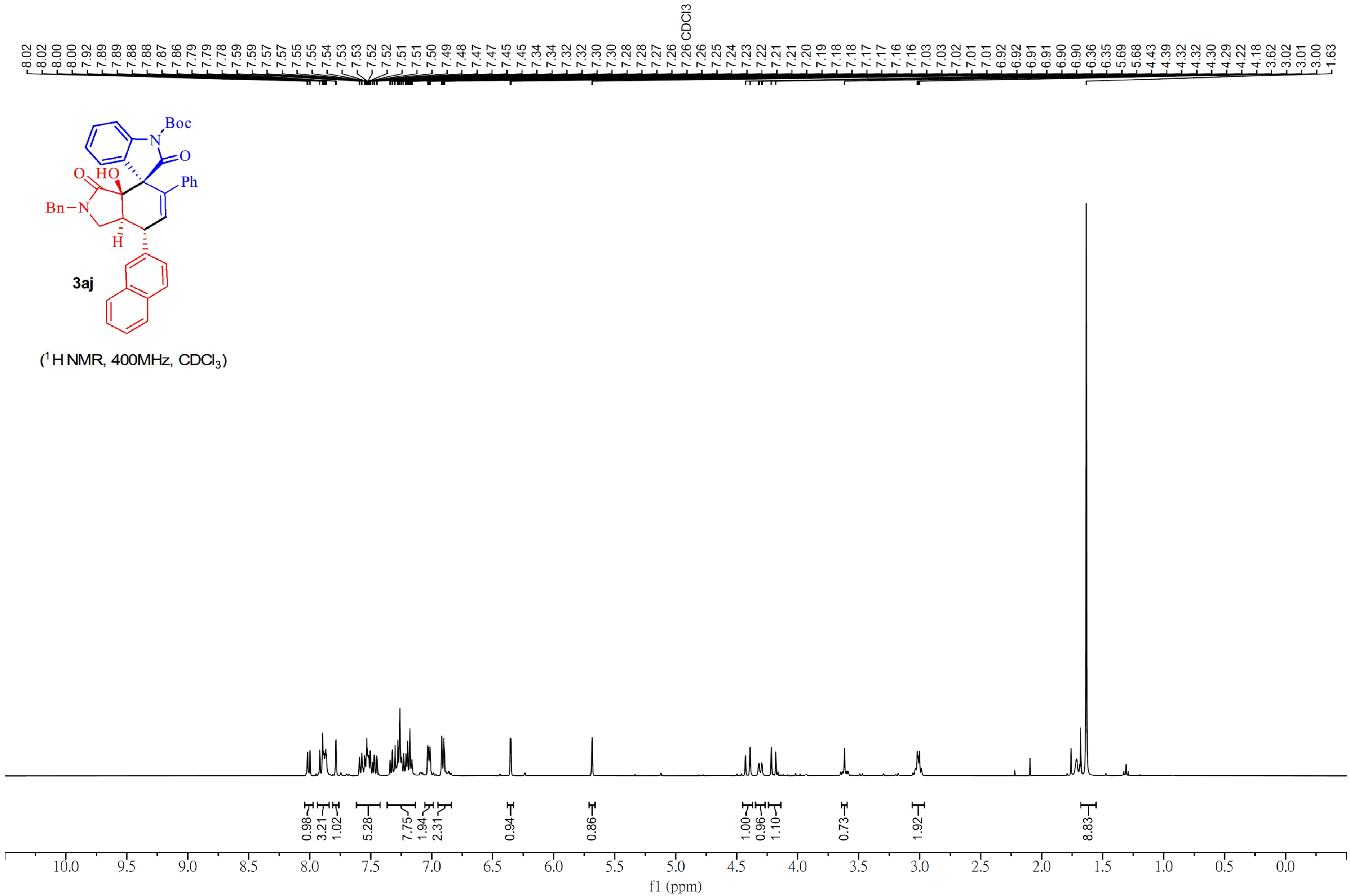

8.02 8.02 8.00 8.00 7.92 7.89 7.88 7.88 7.87 7.86 7.79 7.79 7.78 7.59 7.59 7.57 7.55 7.54 7.53 7.53 7.52 7.52 7.51 7.50 7.49 7.48 7.47 7.45 7.45 7.34 7.32 7.32 7.30 7.30 7.28 7.28 7.27 7.26 7.26 7.26 7.25 7.24 7.23 7.22 7.21 7.21 7.20 7.19 7.18 7.18 7.17 7.17 7.16 7.16 7.03 7.03 7.02 7.01 7.01 6.92 6.92 6.91 6.91 6.90 6.90 6.36 6.35 5.69 5.68 4.43 4.39 4.32 4.32 4.30 4.29 4.22 4.18 3.62 3.02 3.01 3.00 1.63
CDCl3
Boc
N
O
HO
O
Ph
Bn−N
H
3aj
(1H NMR, 400MHz, CDCl3)
0.98 3.21 1.02 5.28 7.75 1.94 2.31 0.94 0.86 1.00 0.96 1.10 0.73 1.92 8.83
10.0 9.5 9.0 8.5 8.0 7.5 7.0 6.5 6.0 5.5 5.0 4.5 4.0 3.5 3.0 2.5 2.0 1.5 1.0 0.5 0.0
f1 (ppm)

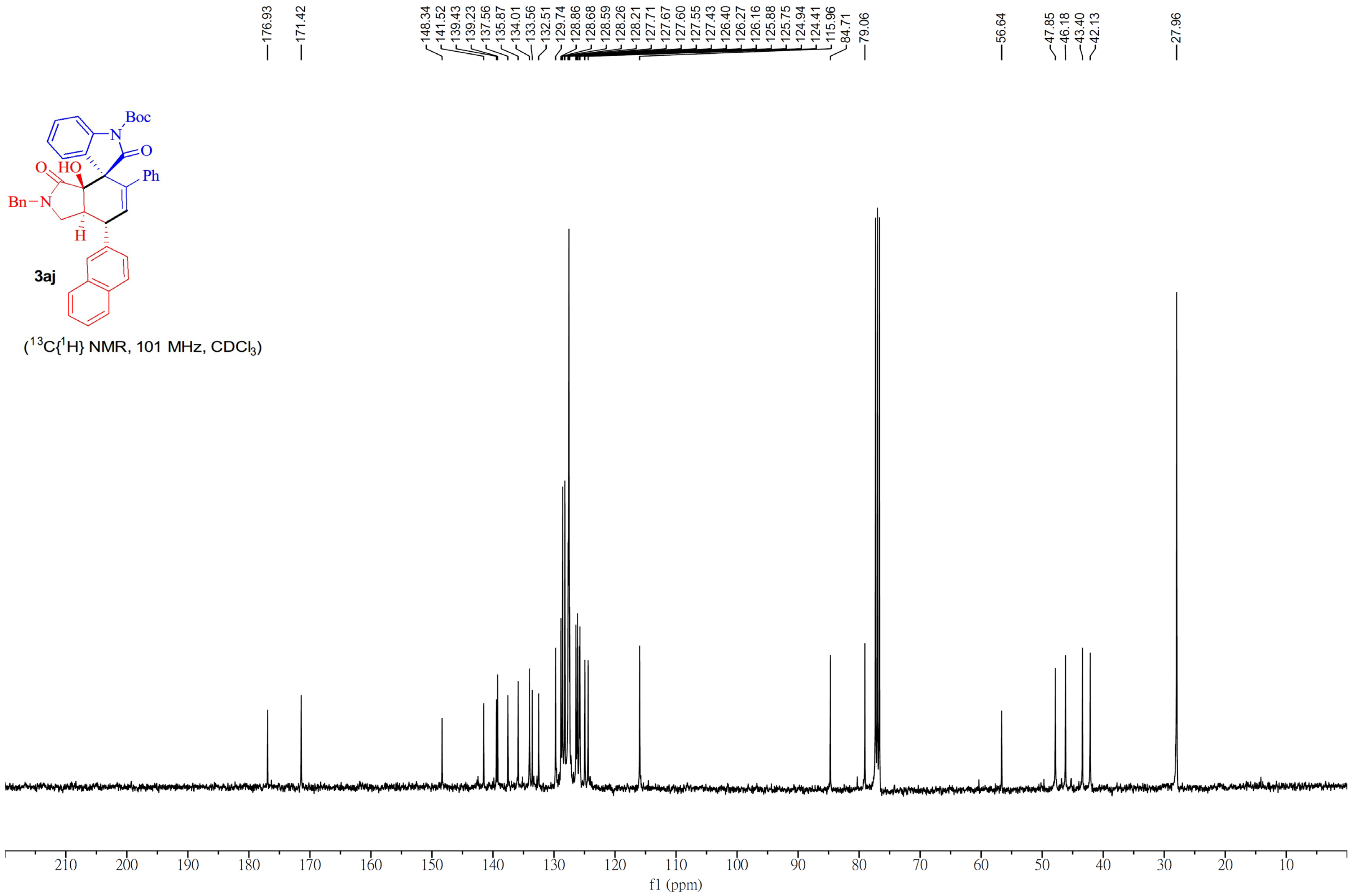
3aj
(13C{1H} NMR, 101 MHz, CDCl3)
176.93
171.42
148.34
141.52
139.43
139.23
137.56
135.87
134.01
133.56
132.51
129.74
128.86
128.68
128.59
128.26
128.21
127.71
127.67
127.60
127.55
127.43
126.40
126.27
126.16
125.88
125.75
124.94
124.41
115.96
84.71
79.06
56.64
47.85
46.18
43.40
42.13
27.96
210
200
190
180
170
160
150
140
130
120
110
100
90
80
70
60
50
40
30
20
10
f1 (ppm)

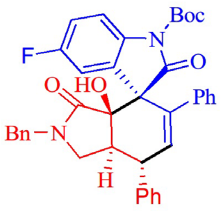


**3ba**

($^1$H NMR, 400MHz, $CDCl_3$)

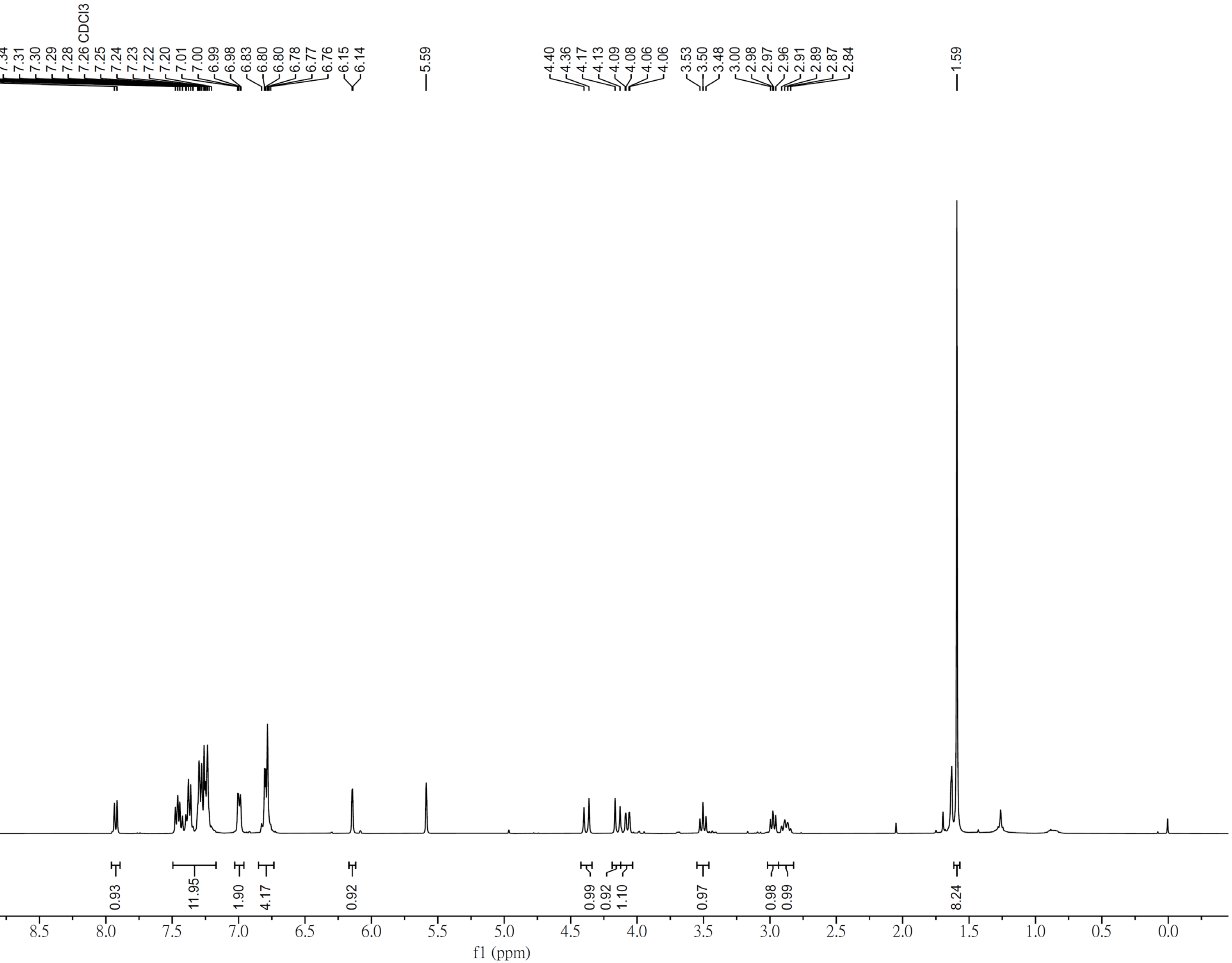

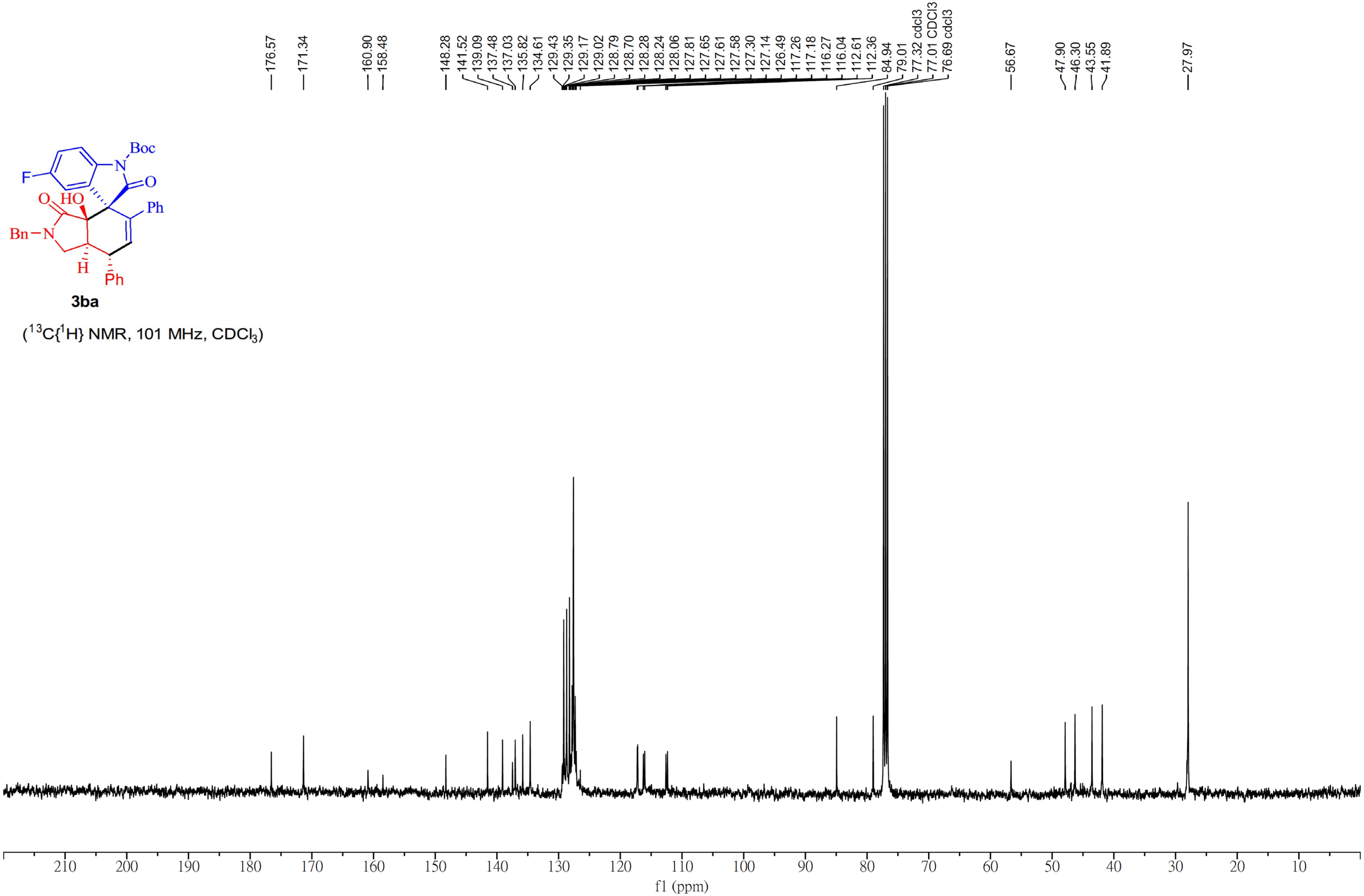

176.57
171.34
160.90
158.48
148.28
141.52
139.09
137.48
137.03
135.82
134.61
129.43
129.35
129.17
129.02
128.79
128.70
128.28
128.24
128.06
127.81
127.65
127.61
127.58
127.30
127.14
126.49
117.26
117.18
116.27
116.04
112.61
112.36
84.94
79.01
77.32 cdcl3
77.01 CDCl3
76.69 cdcl3
56.67
47.90
46.30
43.55
41.89
27.97
Boc
N
O
F
HO
O
Ph
Bn
N
H
Ph
3ba
($^{13}C\{^{1}H\}$ NMR, 101 MHz, $CDCl_3$)
210
200
190
180
170
160
150
140
130
120
110
100
90
80
70
60
50
40
30
20
10
f1 (ppm)

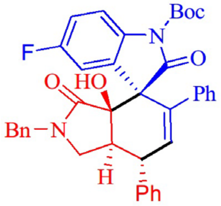


**3ba**

($^{19}F$ NMR, 376MHz, $CDCl_3$)

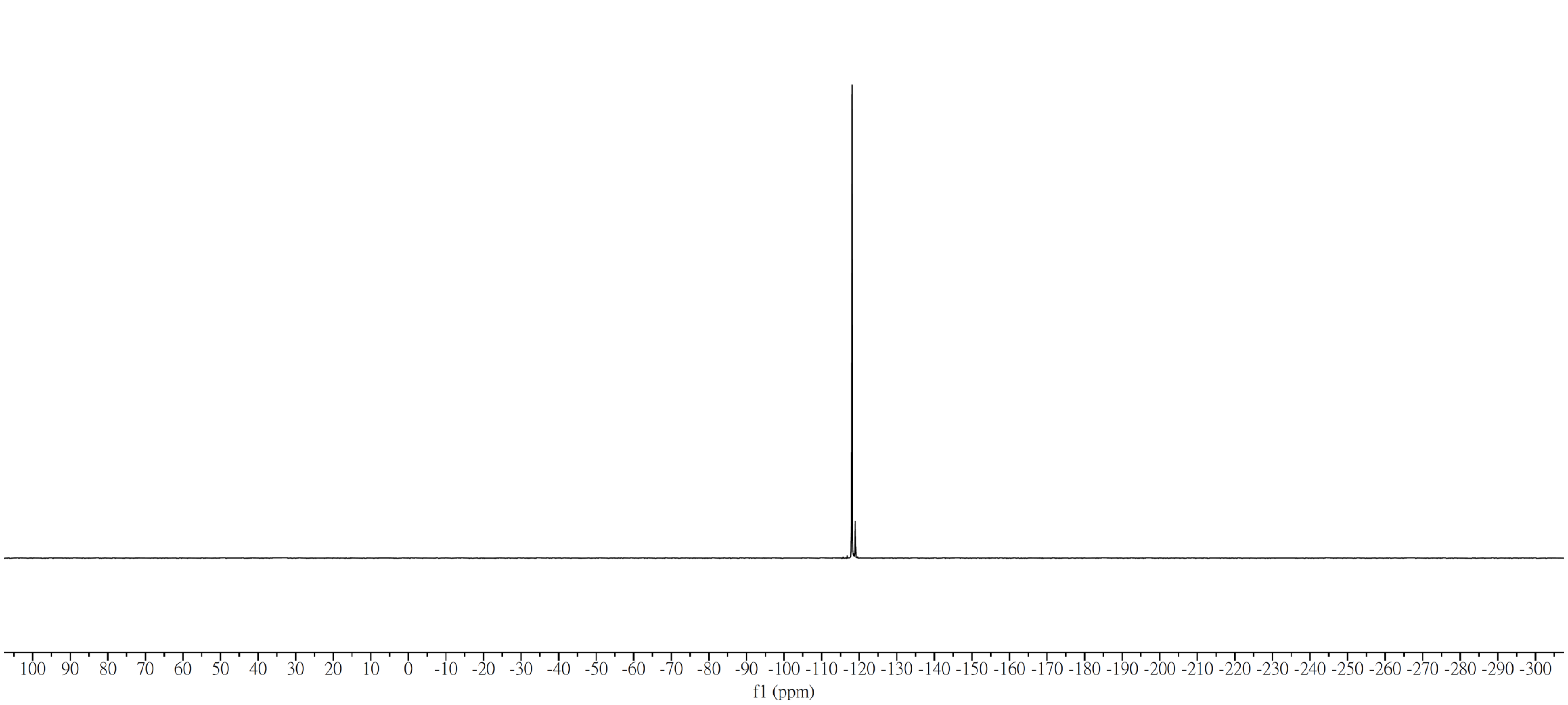

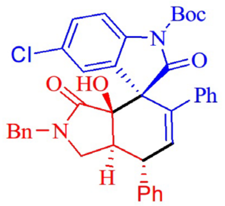


**3ca**

($^{1}H$ NMR, 400MHz, $CDCl_3$)

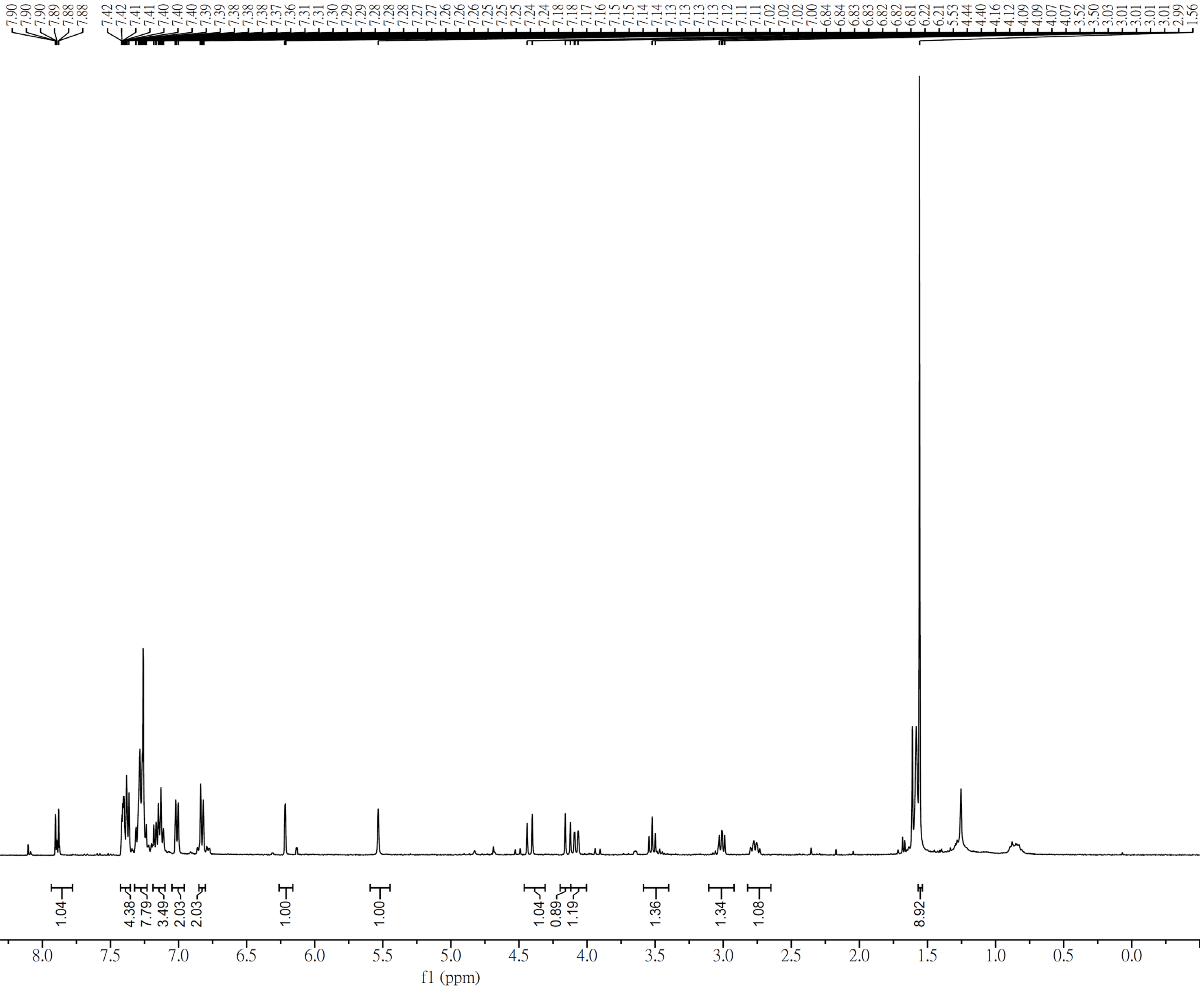

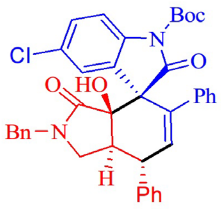


**3ca**

($^{13}C\{^{1}H\}$ NMR, 101 MHz, $CDCl_3$)

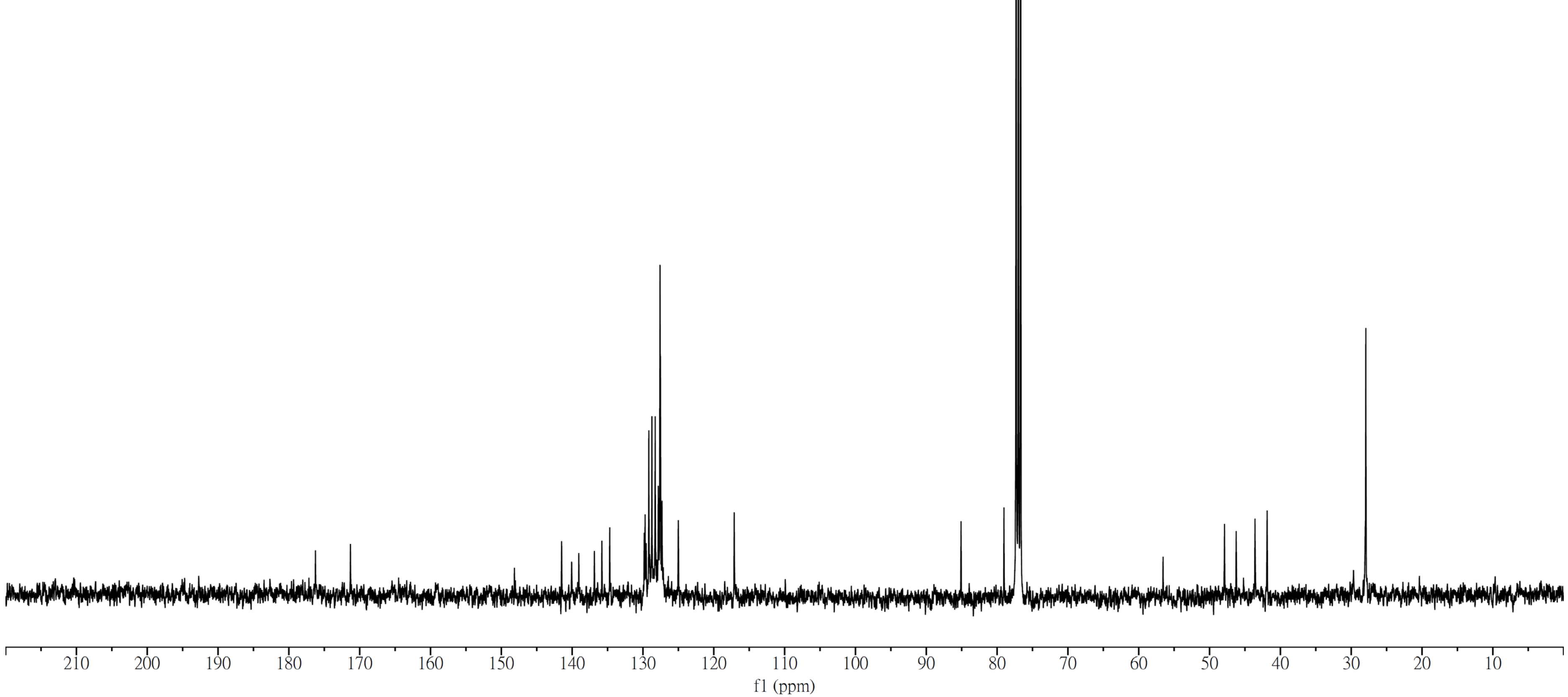

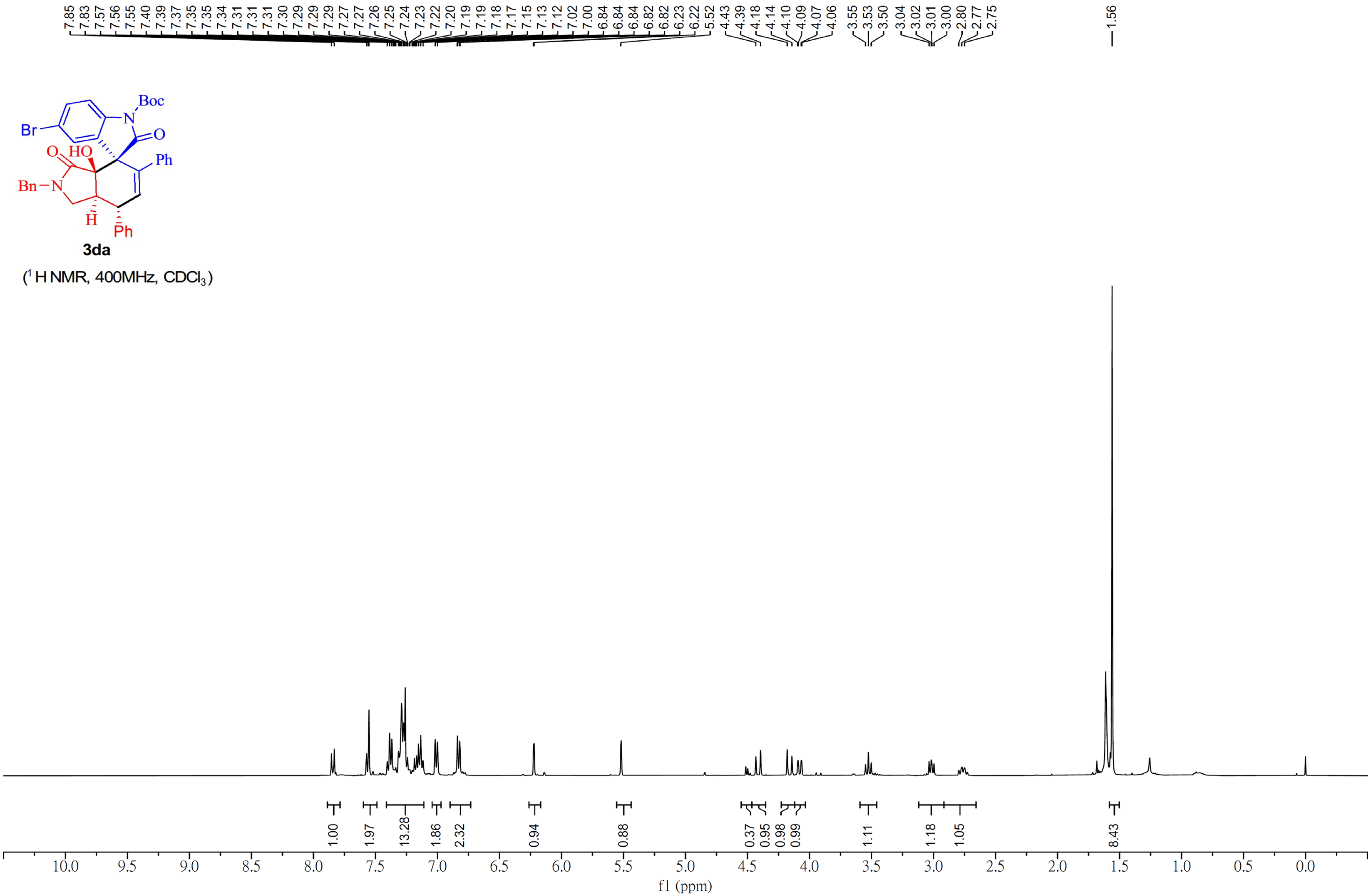


**3da**

($^1$H NMR, 400MHz, $CDCl_3$)

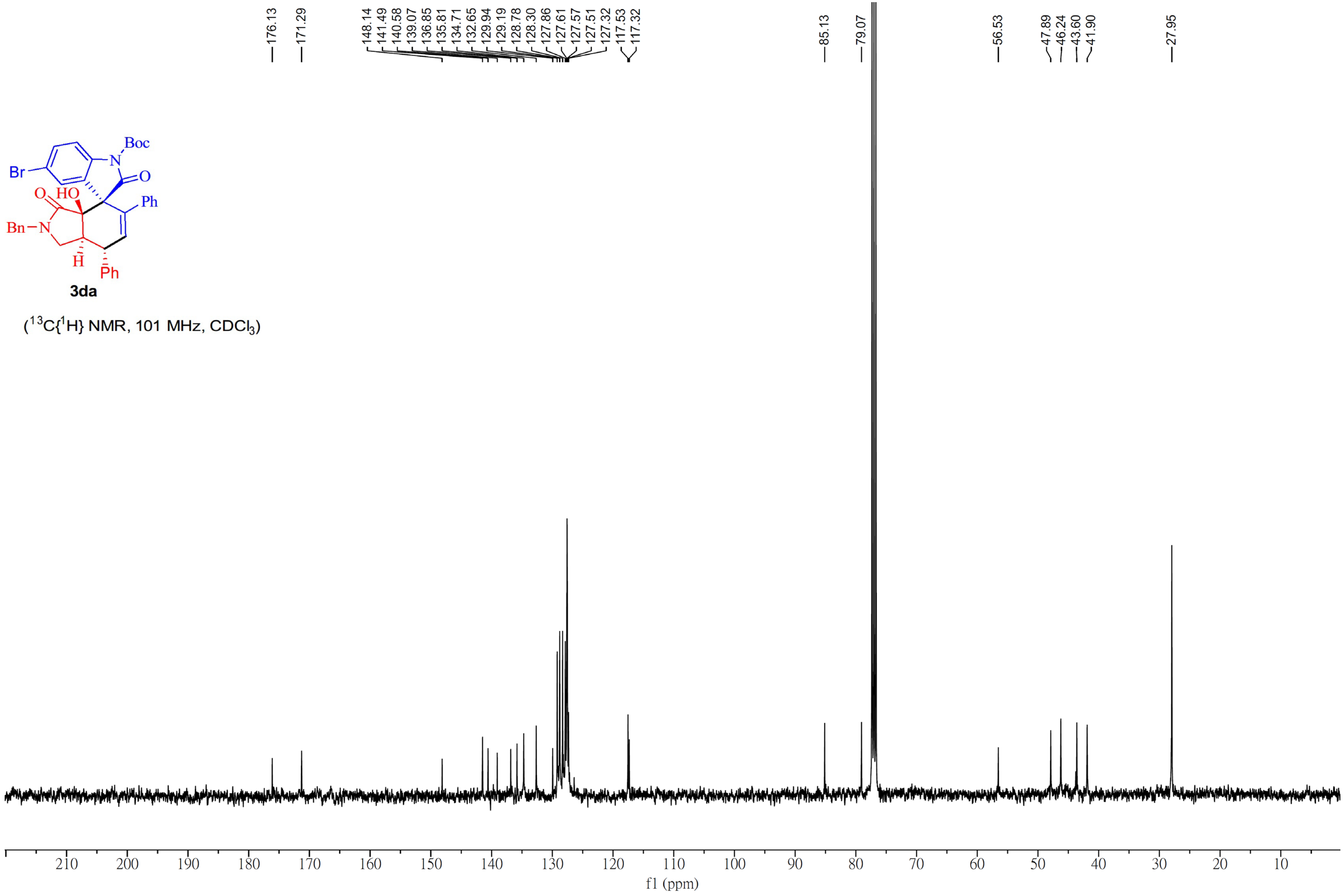


**3da**

($^{13}C\{^1H\}$ NMR, 101 MHz, $CDCl_3$)

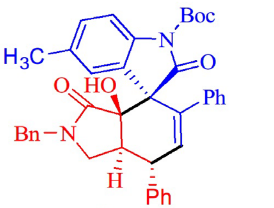

**3ea**

($^1$H NMR, 400MHz, $CDCl_3$)

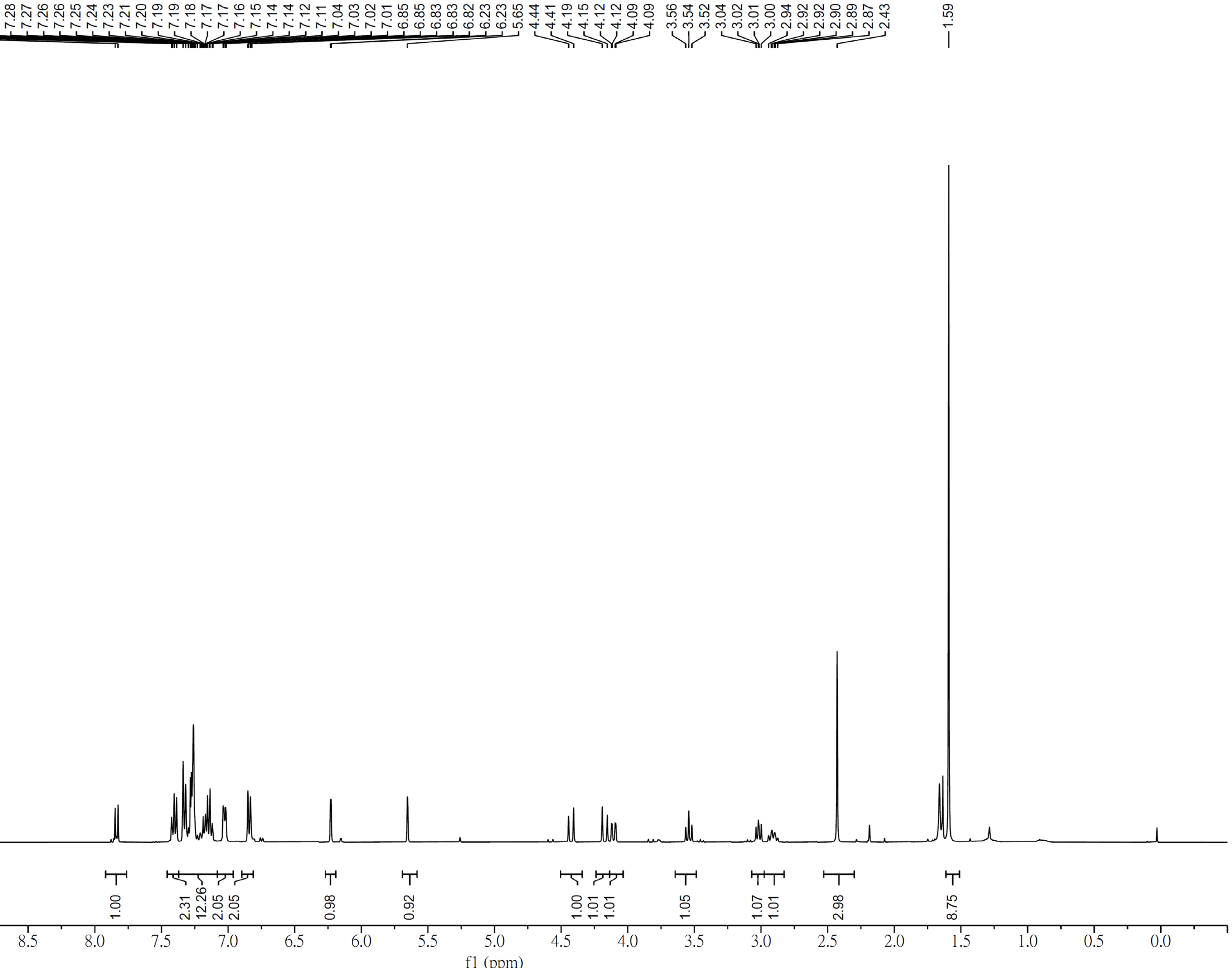

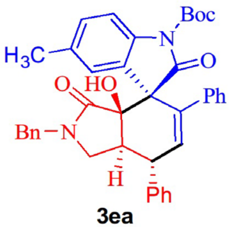

3ea

($^{13}C\{^1H\}$ NMR, 101 MHz, $CDCl_3$)

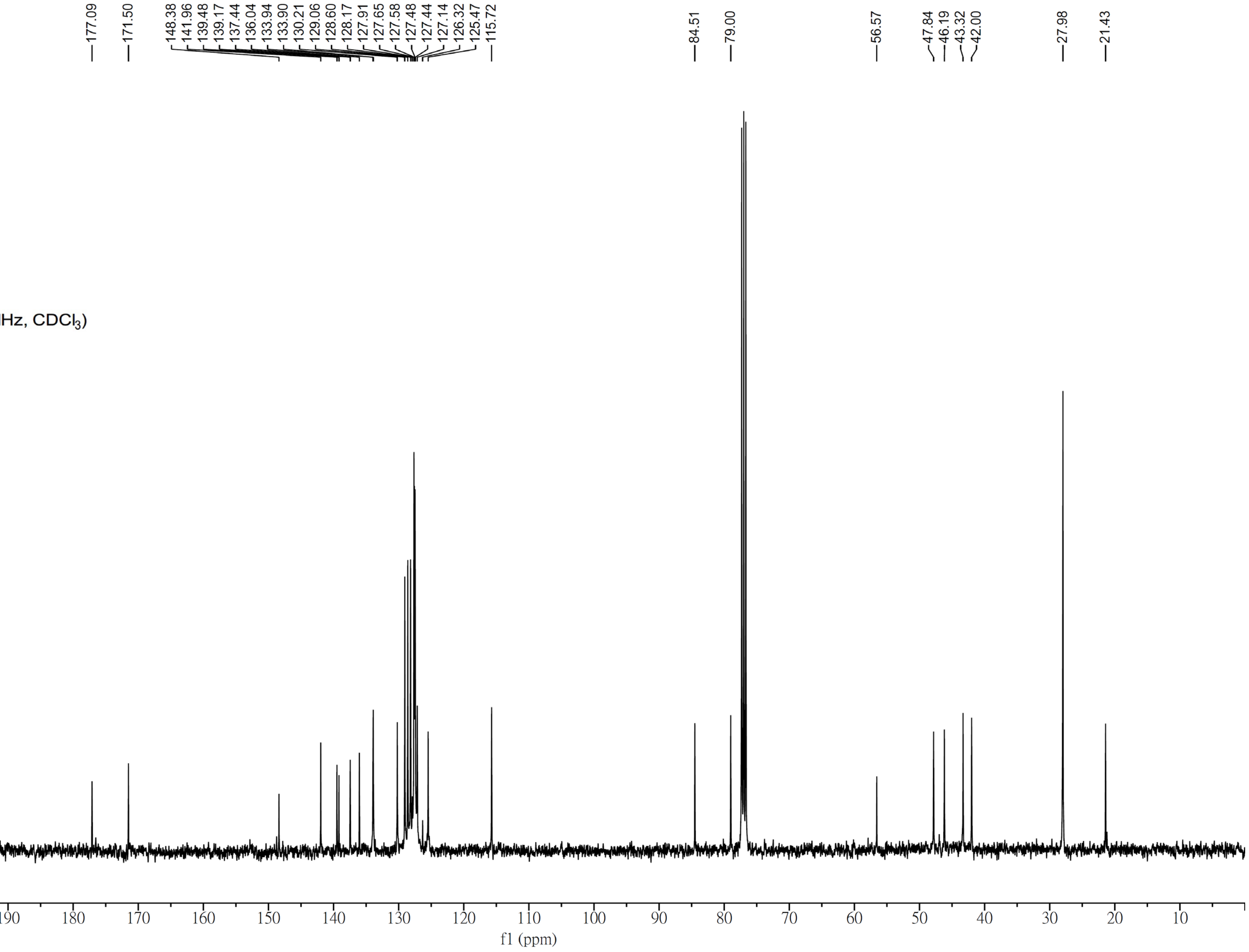

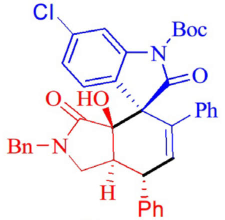


**3fa**

($^1$H NMR, 400MHz, $CDCl_3$)

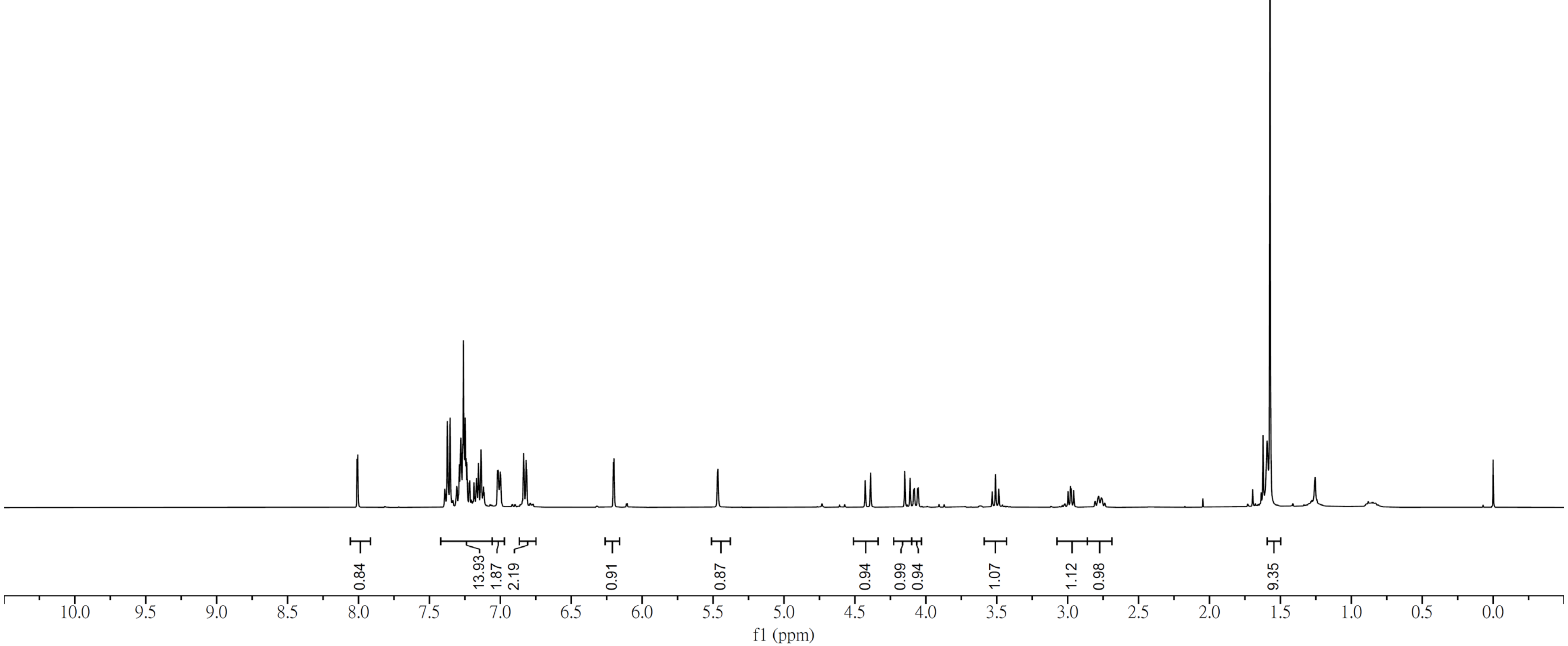

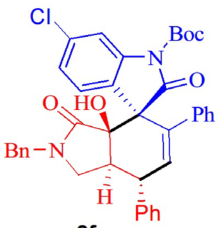

**3fa**

($^{13}C\{^{1}H\}$ NMR, 101 MHz, $CDCl_3$)

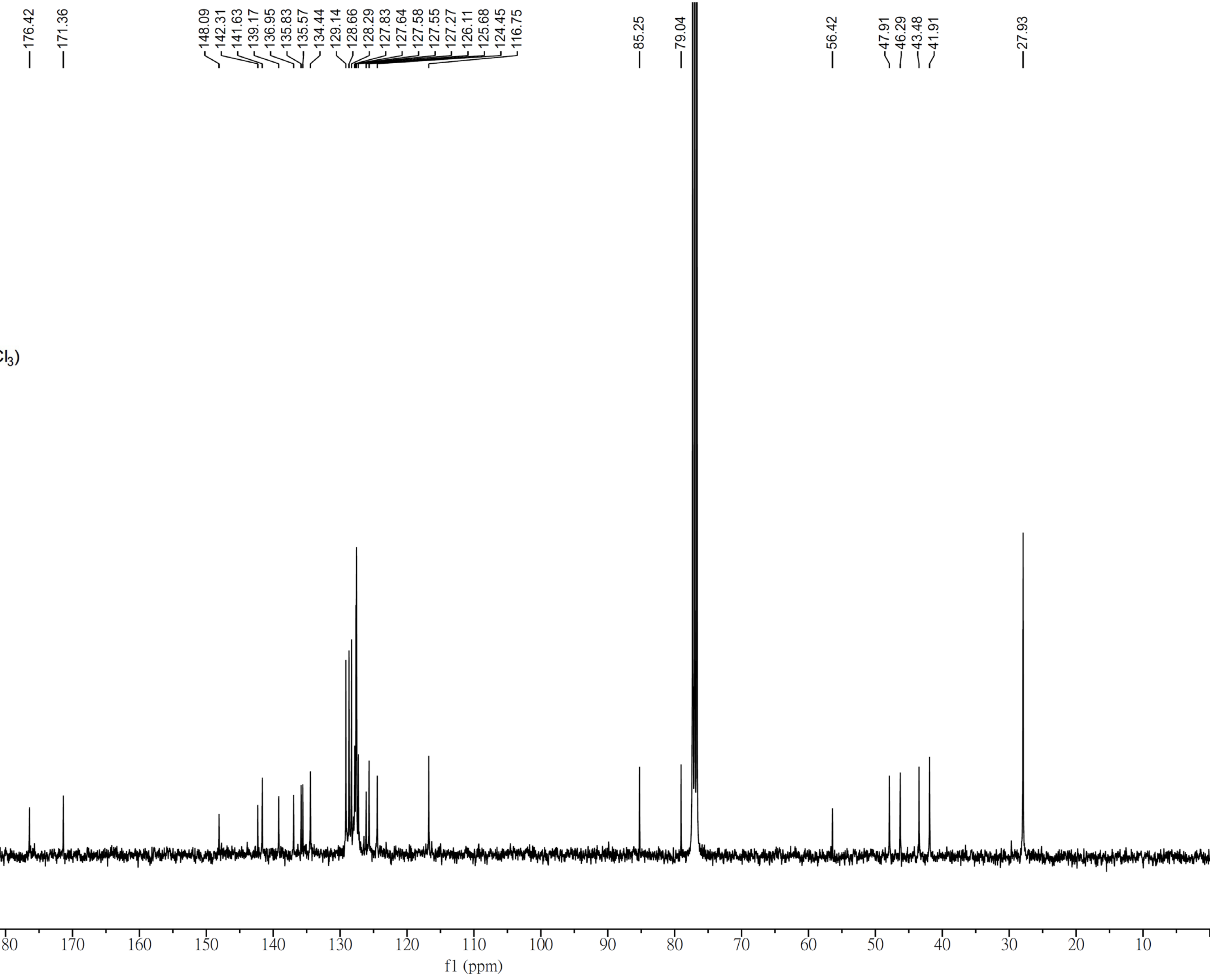

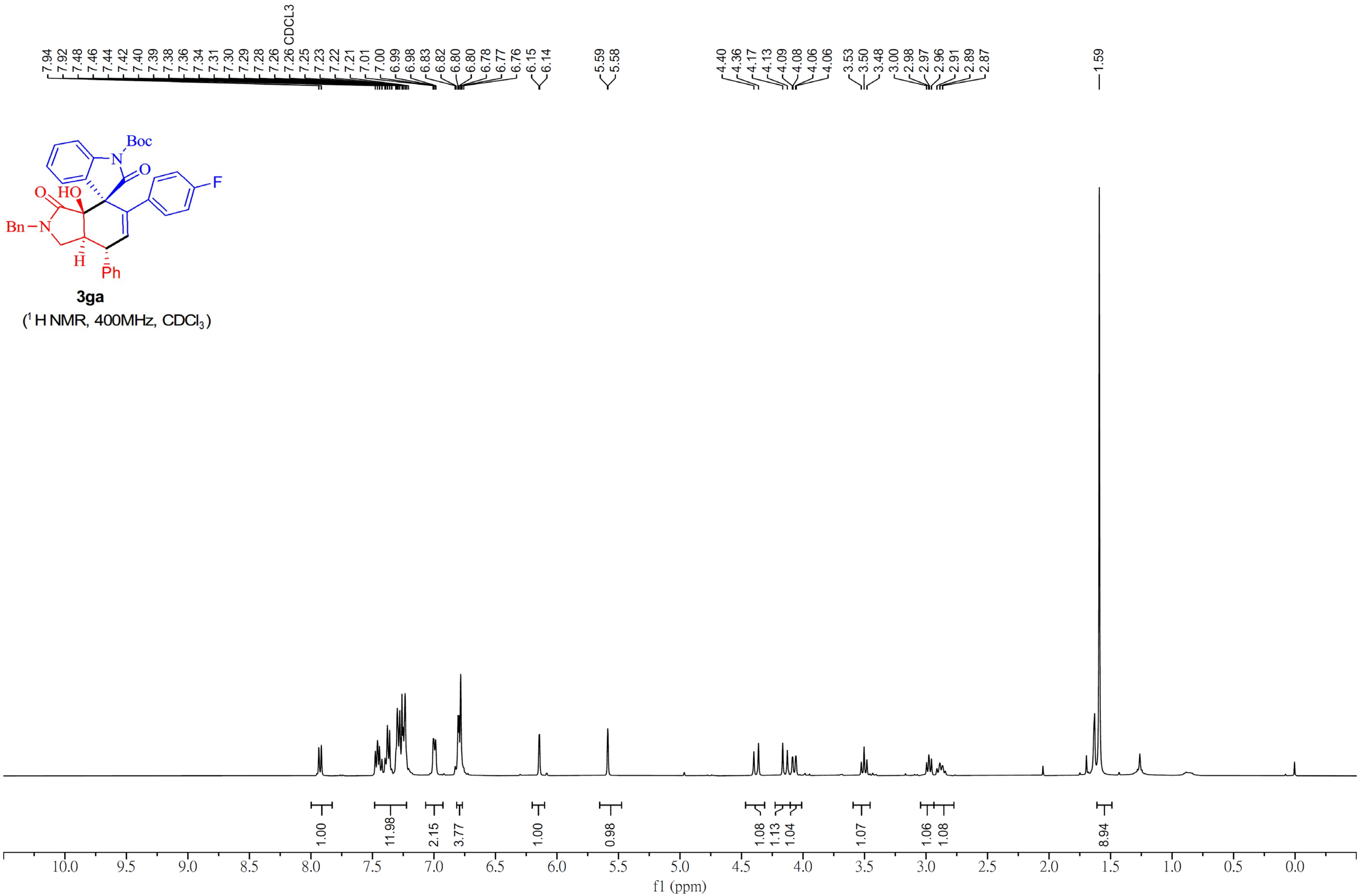


**3ga**

($^1$H NMR, 400MHz, $CDCl_3$)

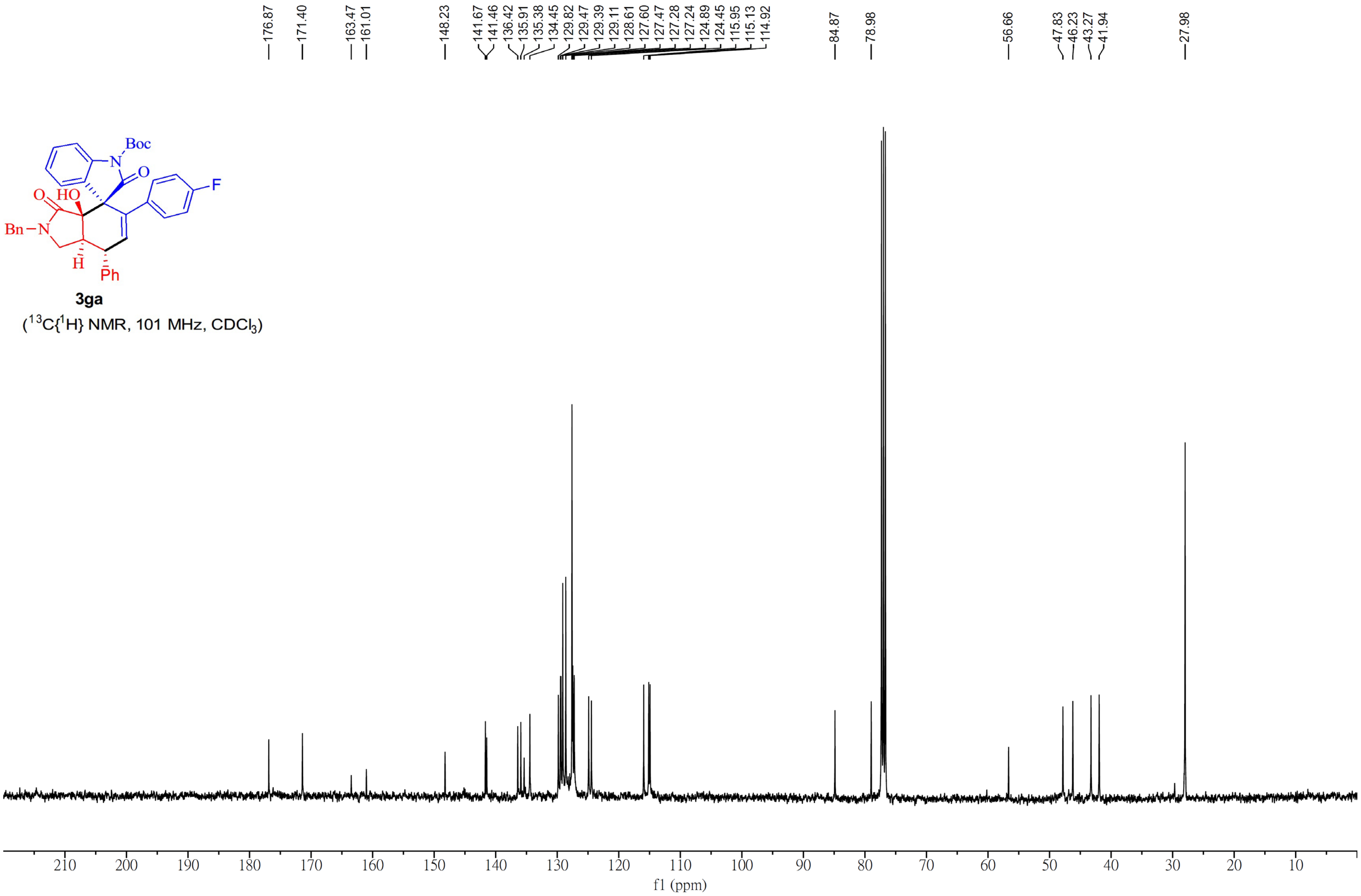


**3ga**

($^{13}C\{^1H\}$ NMR, 101 MHz, $CDCl_3$)

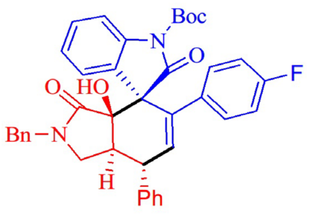


**3ga**

($^{19}$F NMR, 376MHz, $CDCl_3$)

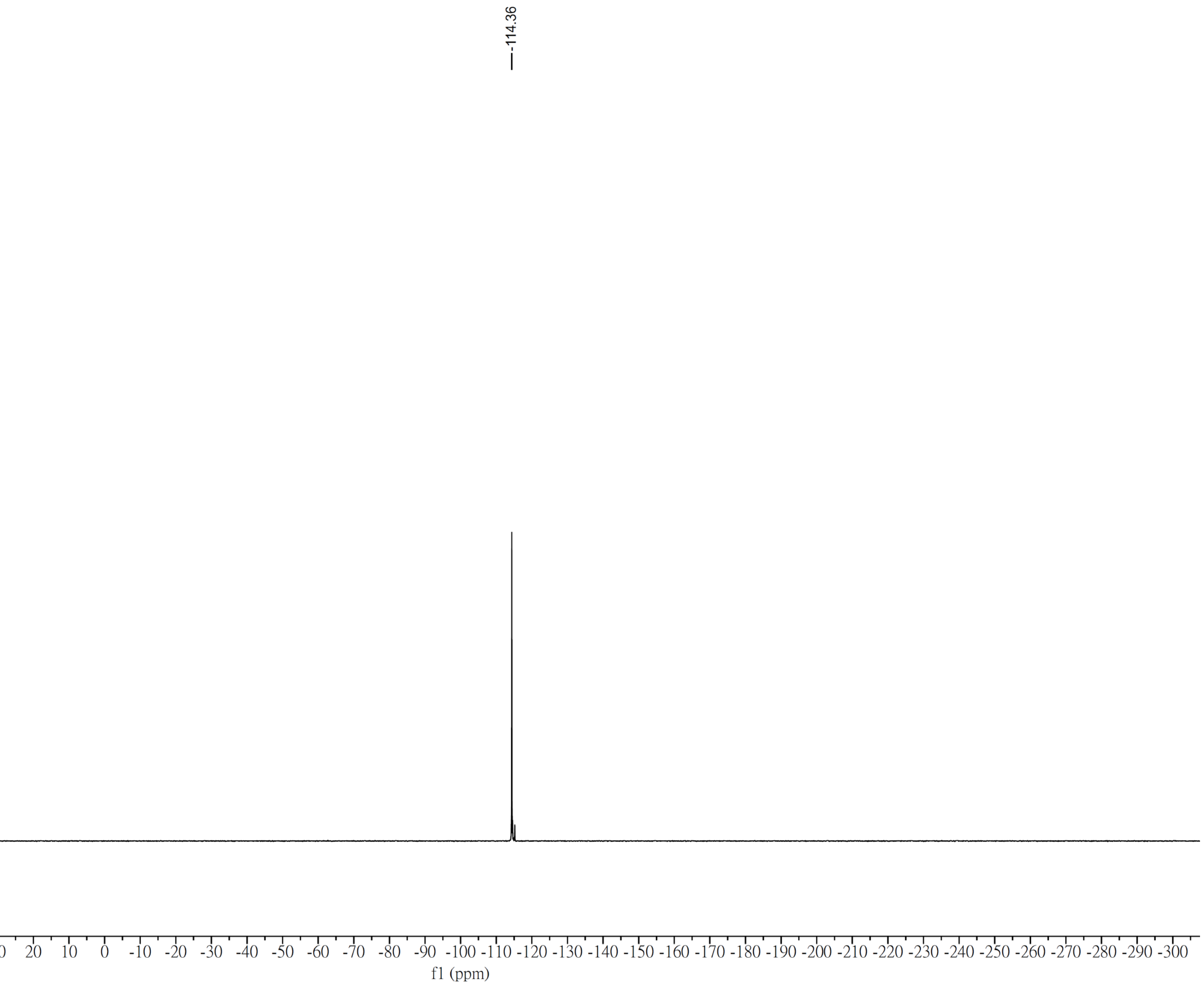

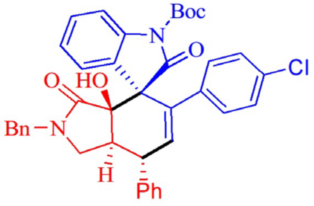


**3ha**

($^1$H NMR, 400MHz, $CDCl_3$)

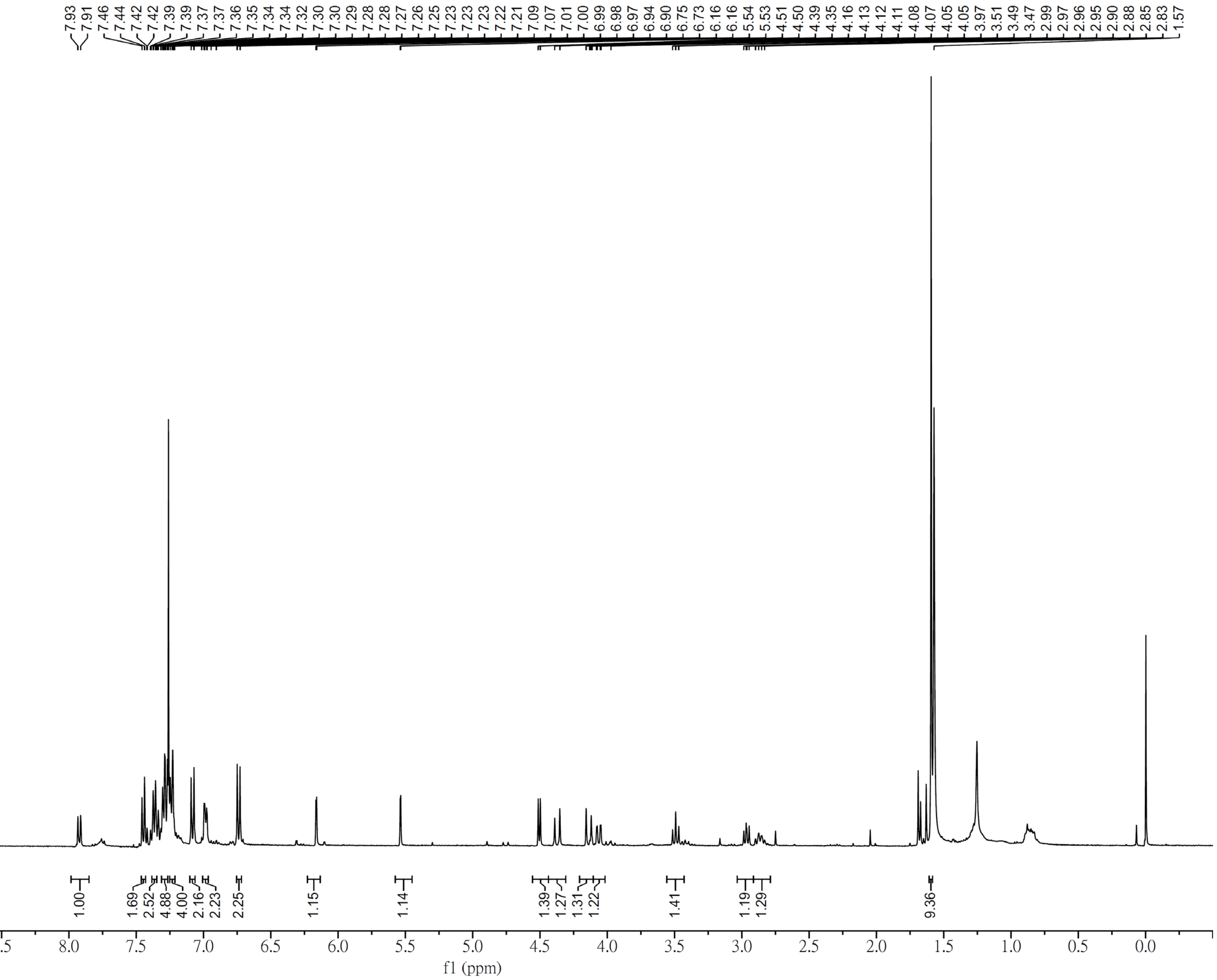

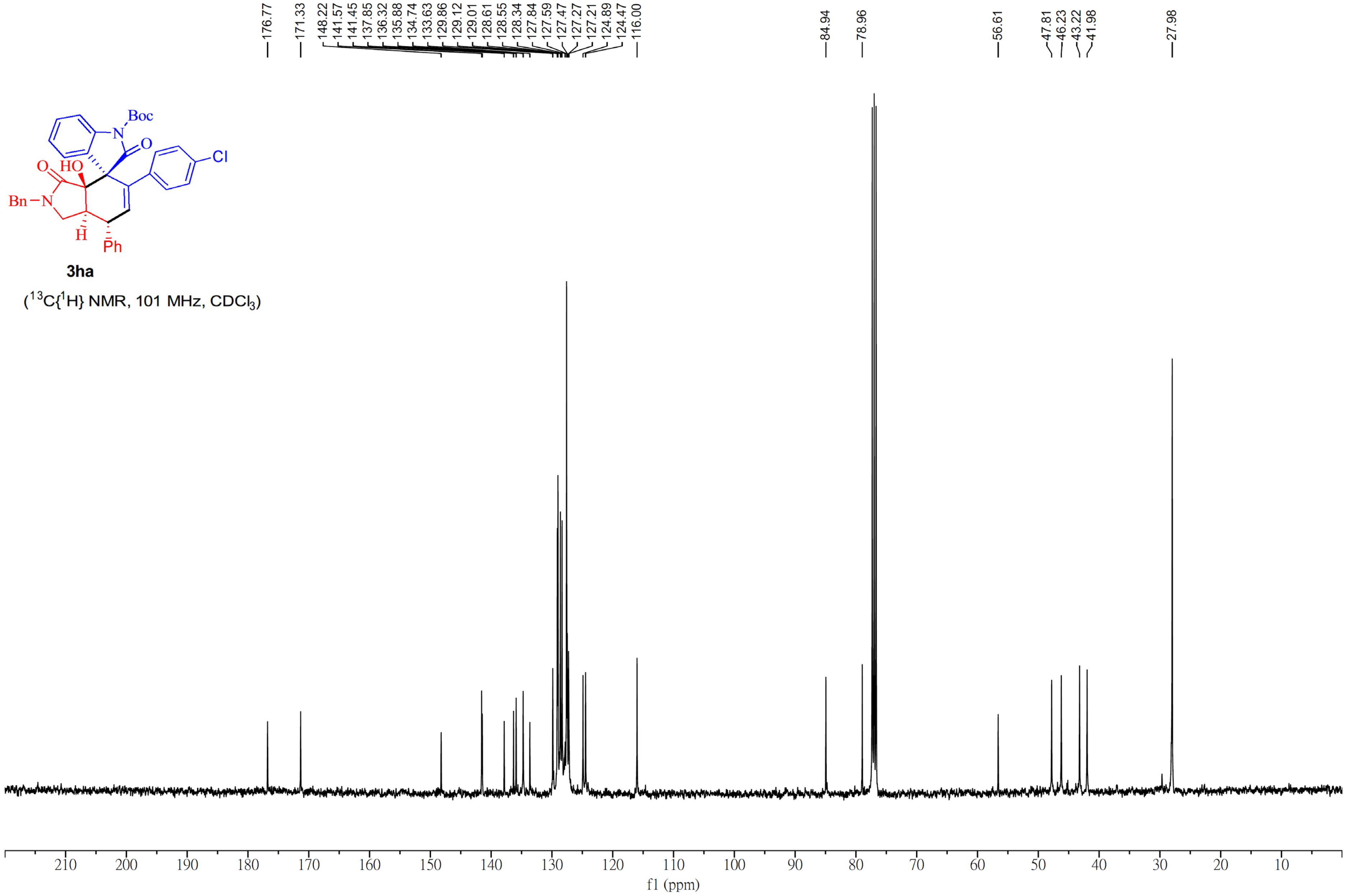


**3ha**

($^{13}C\{^{1}H\}$ NMR, 101 MHz, $CDCl_3$)

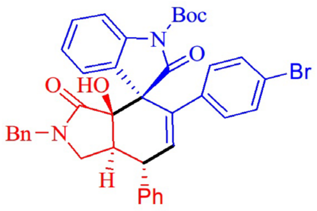


**3ia**

($^{1}$H NMR, 400MHz, $CDCl_3$)

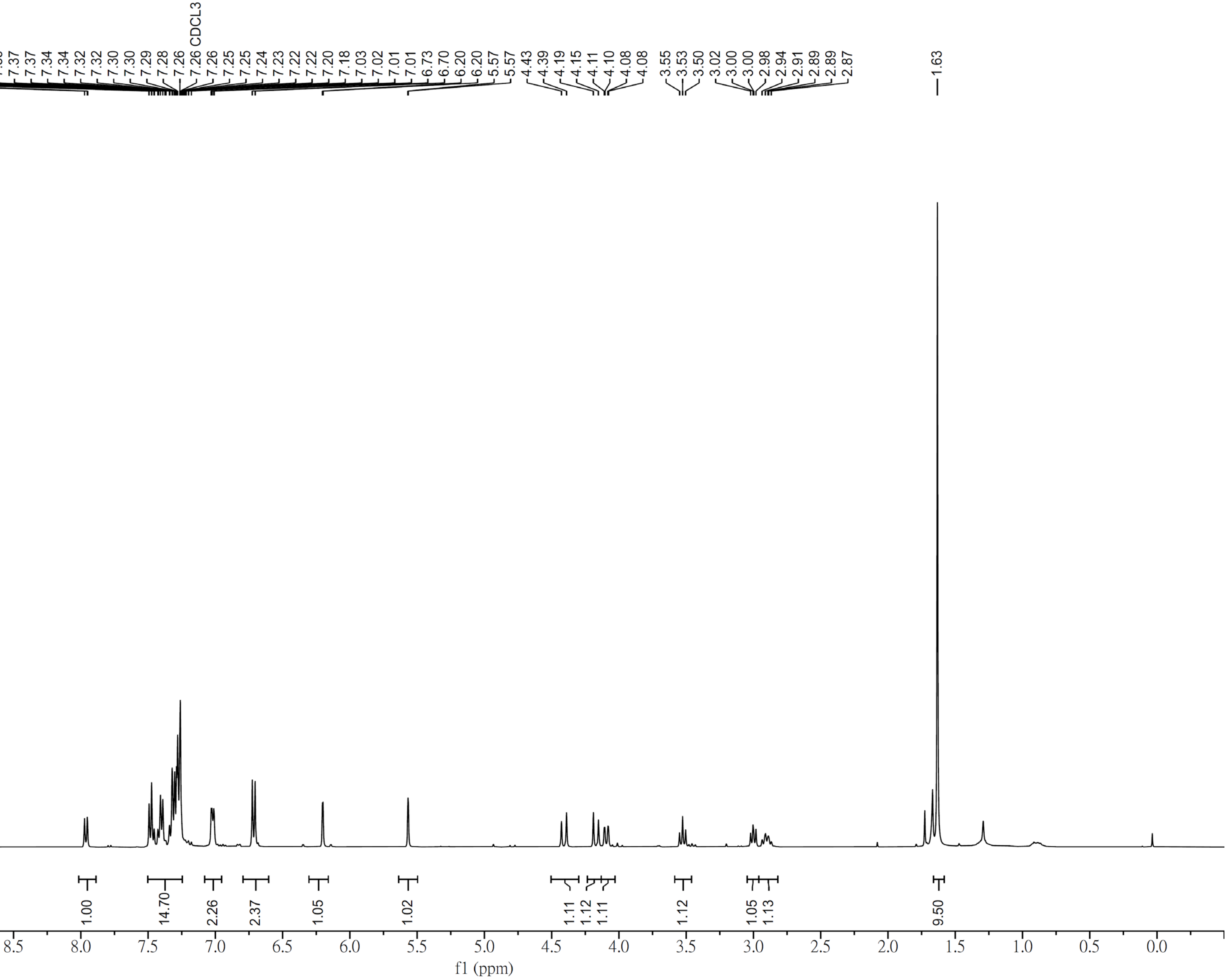

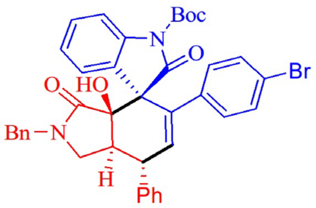


**3ia**

($^{13}C\{^1H\}$ NMR, 101 MHz, $CDCl_3$)

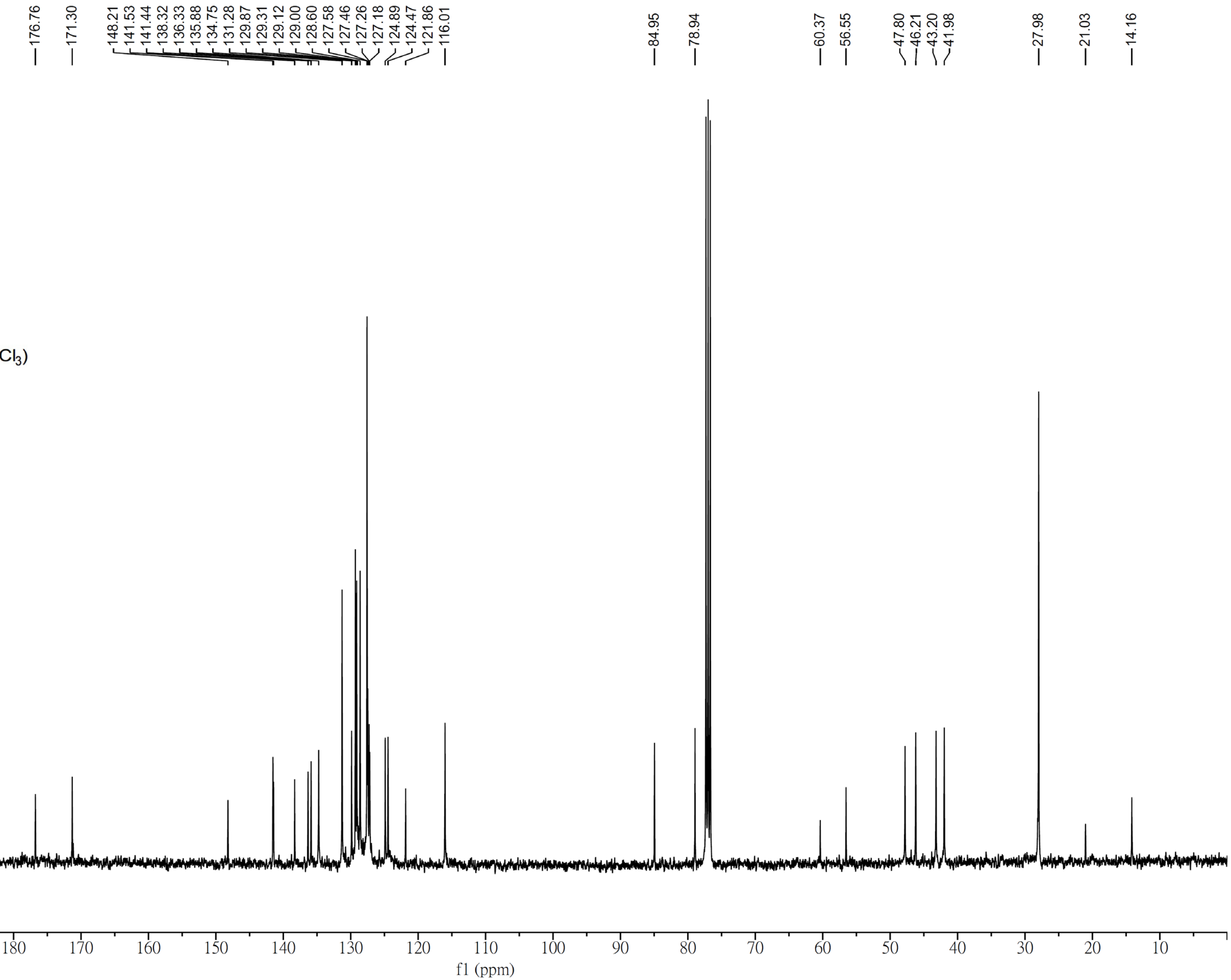

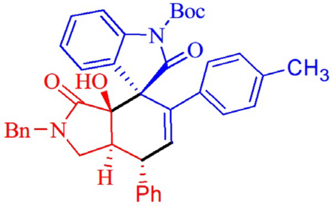


**3ja**

($^1$H NMR, 400MHz, $CDCl_3$)

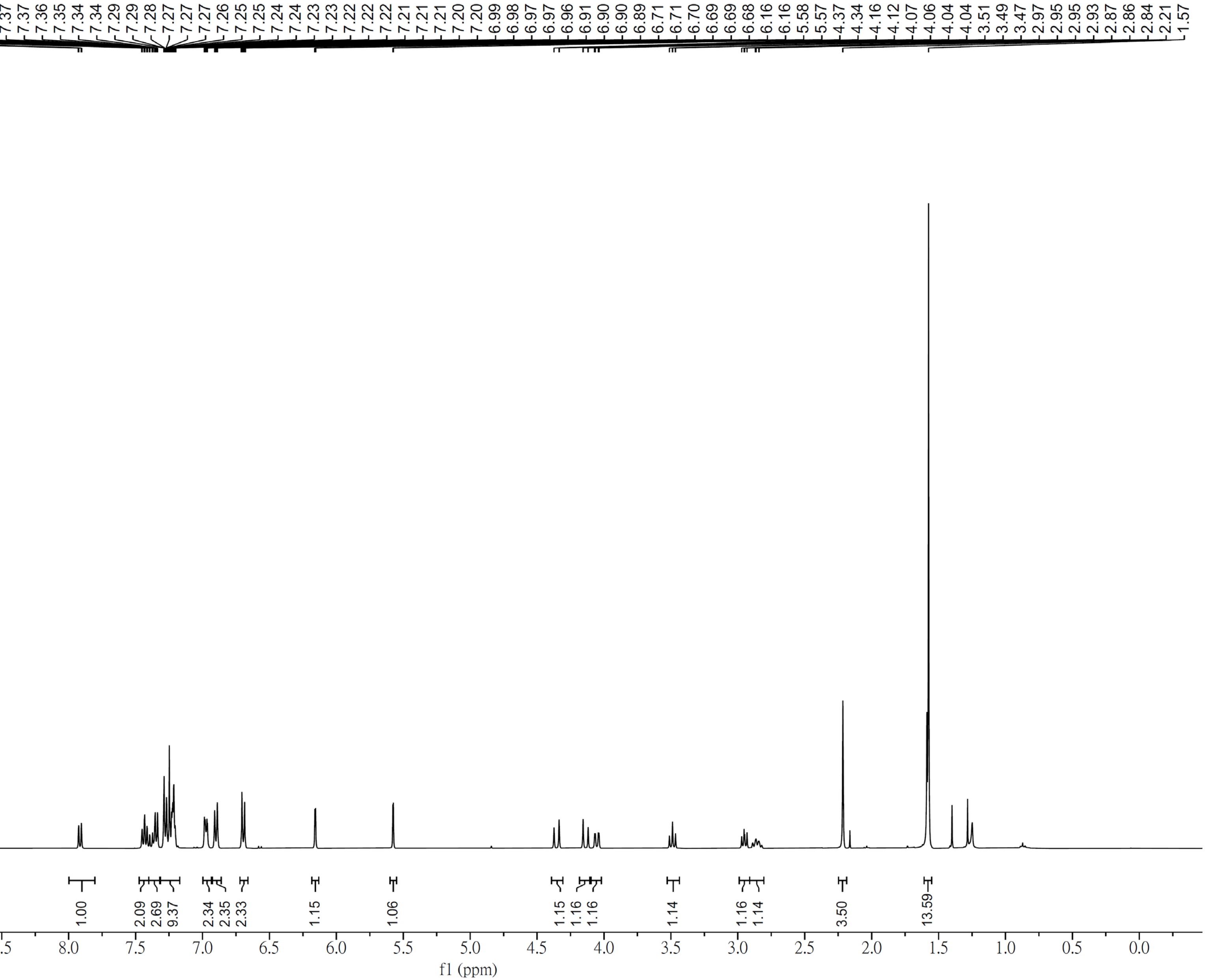

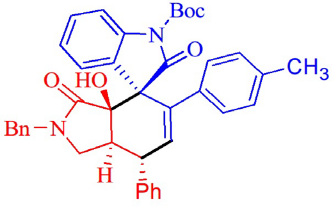


**3ja**

($^{13}C\{^{1}H\}$ NMR, 101 MHz, $CDCl_3$)

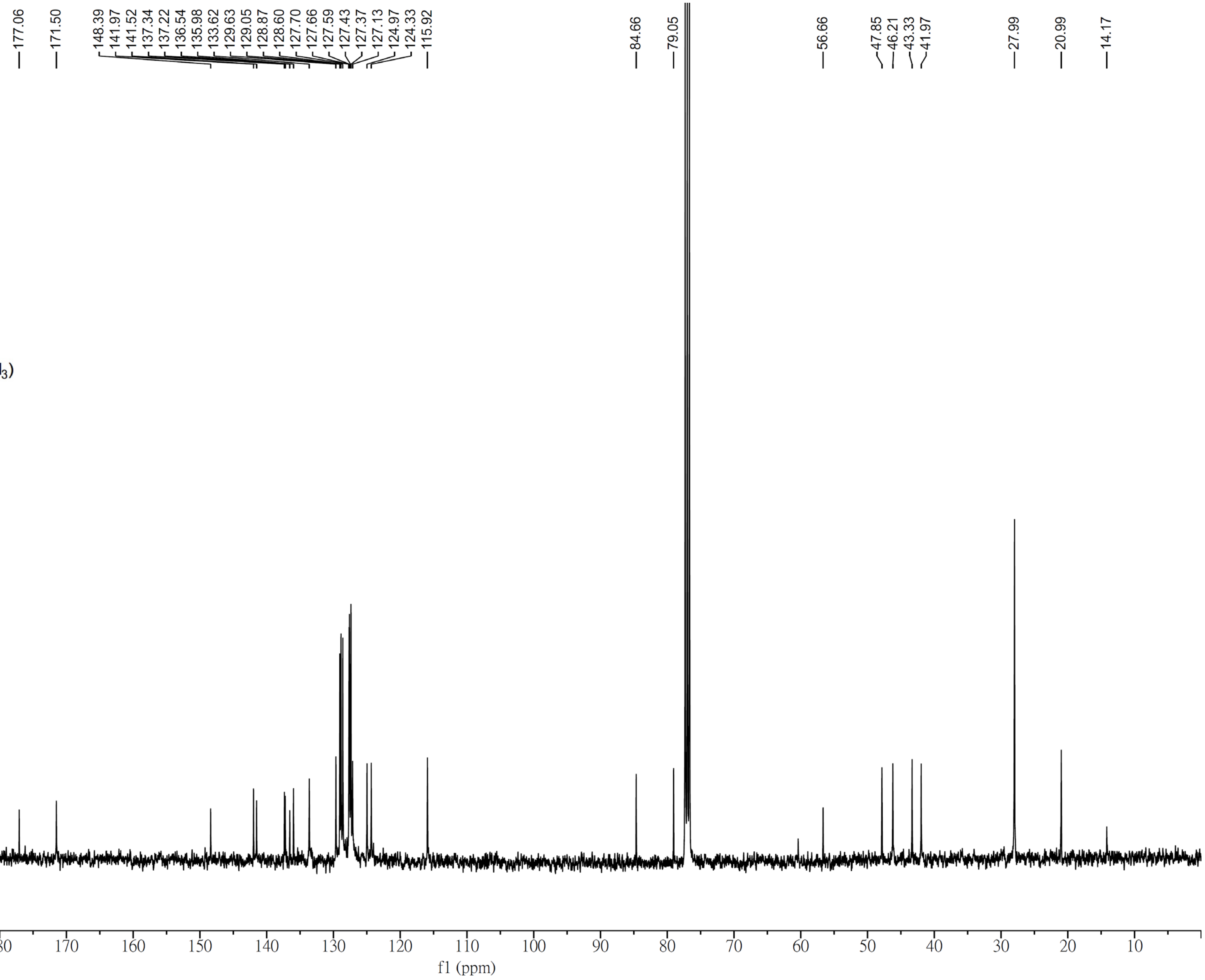

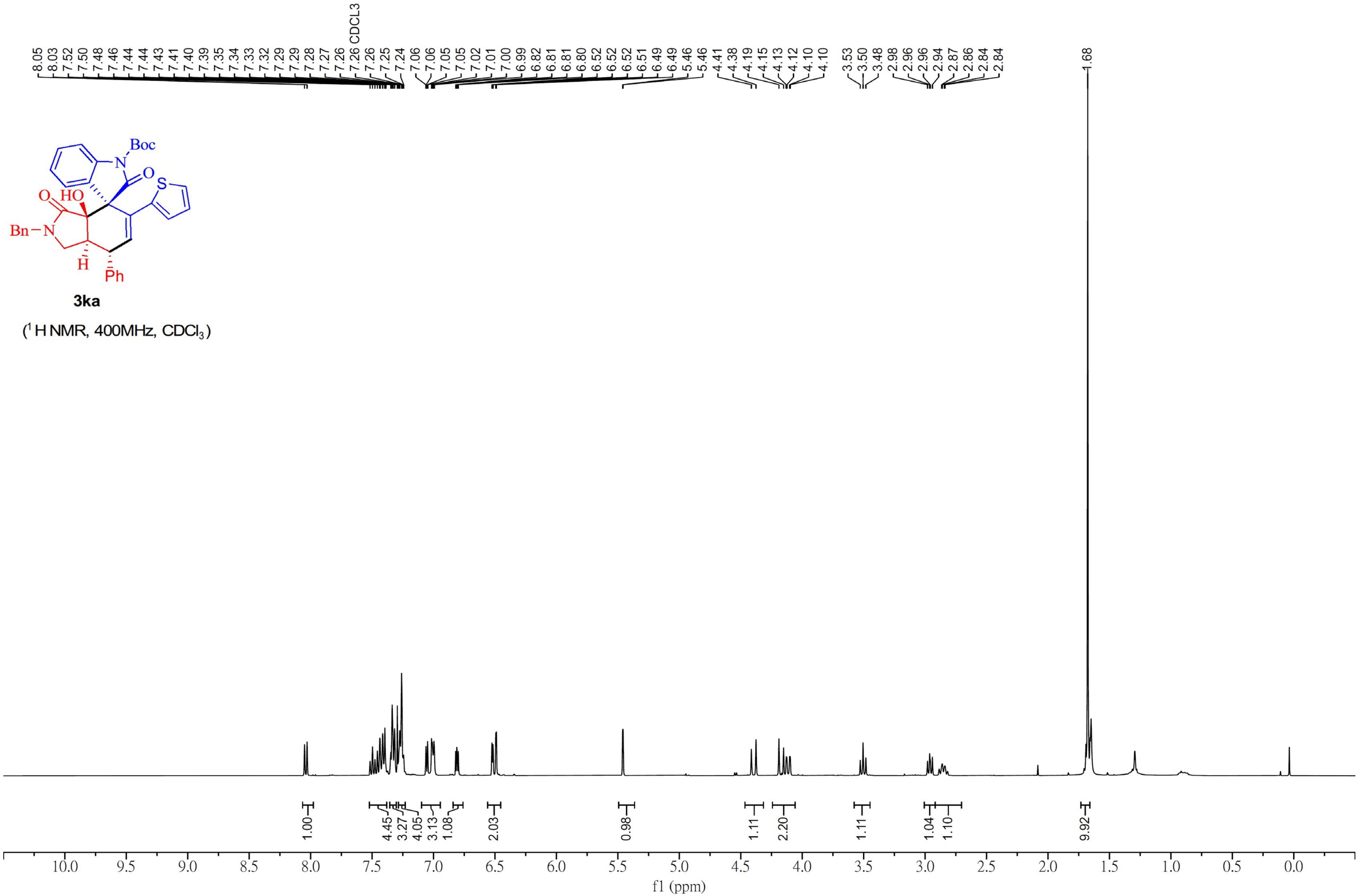


**3ka**

($^1$H NMR, 400MHz, $CDCl_3$)

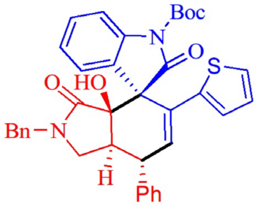


**3ka**

($^{13}C\{^1H\}$ NMR, 101 MHz, $CDCl_3$)

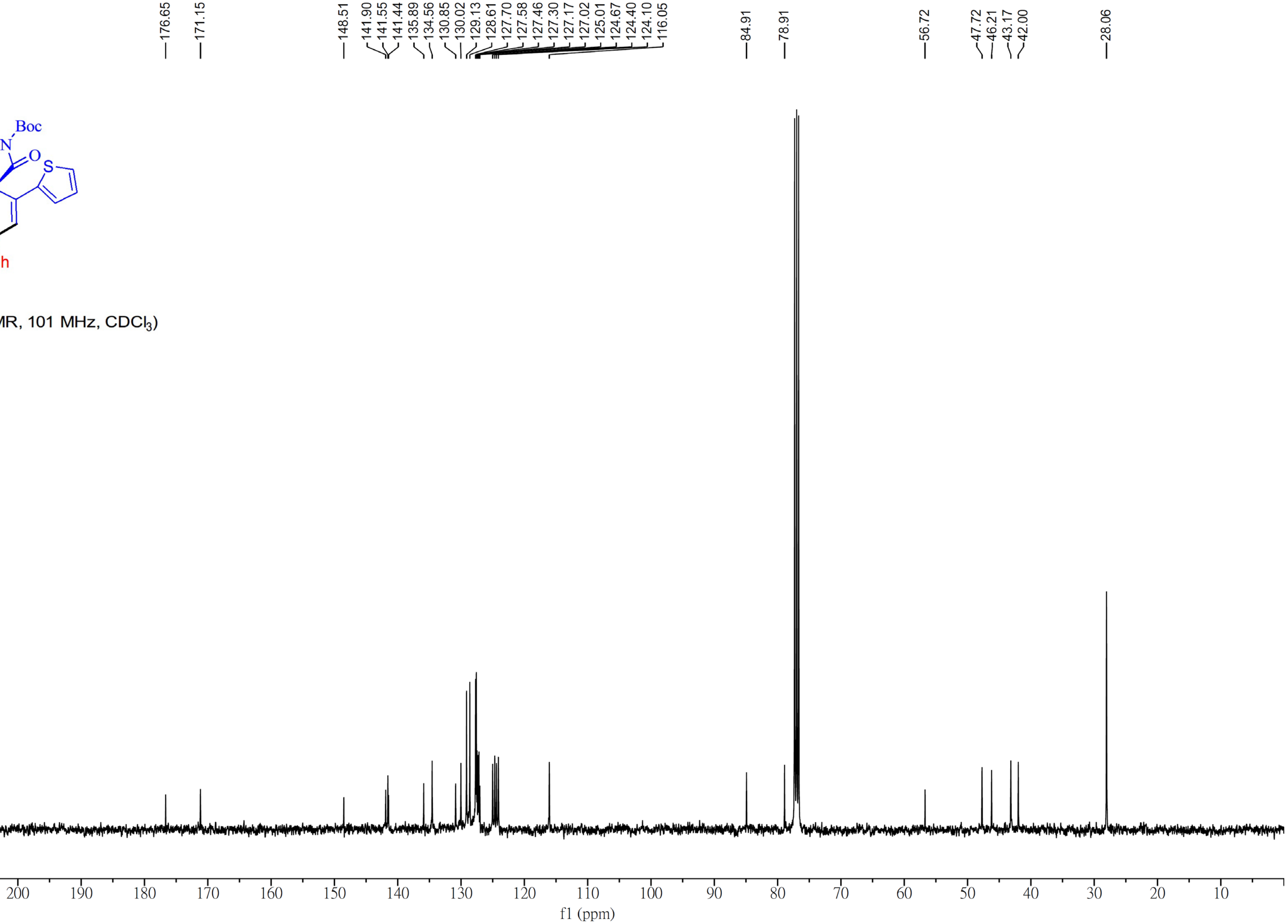

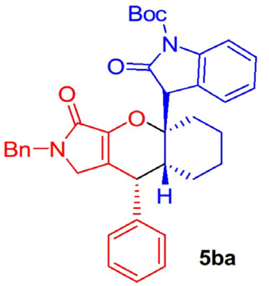


**5ba**

($^1$H NMR, 400MHz, $CDCl_3$)

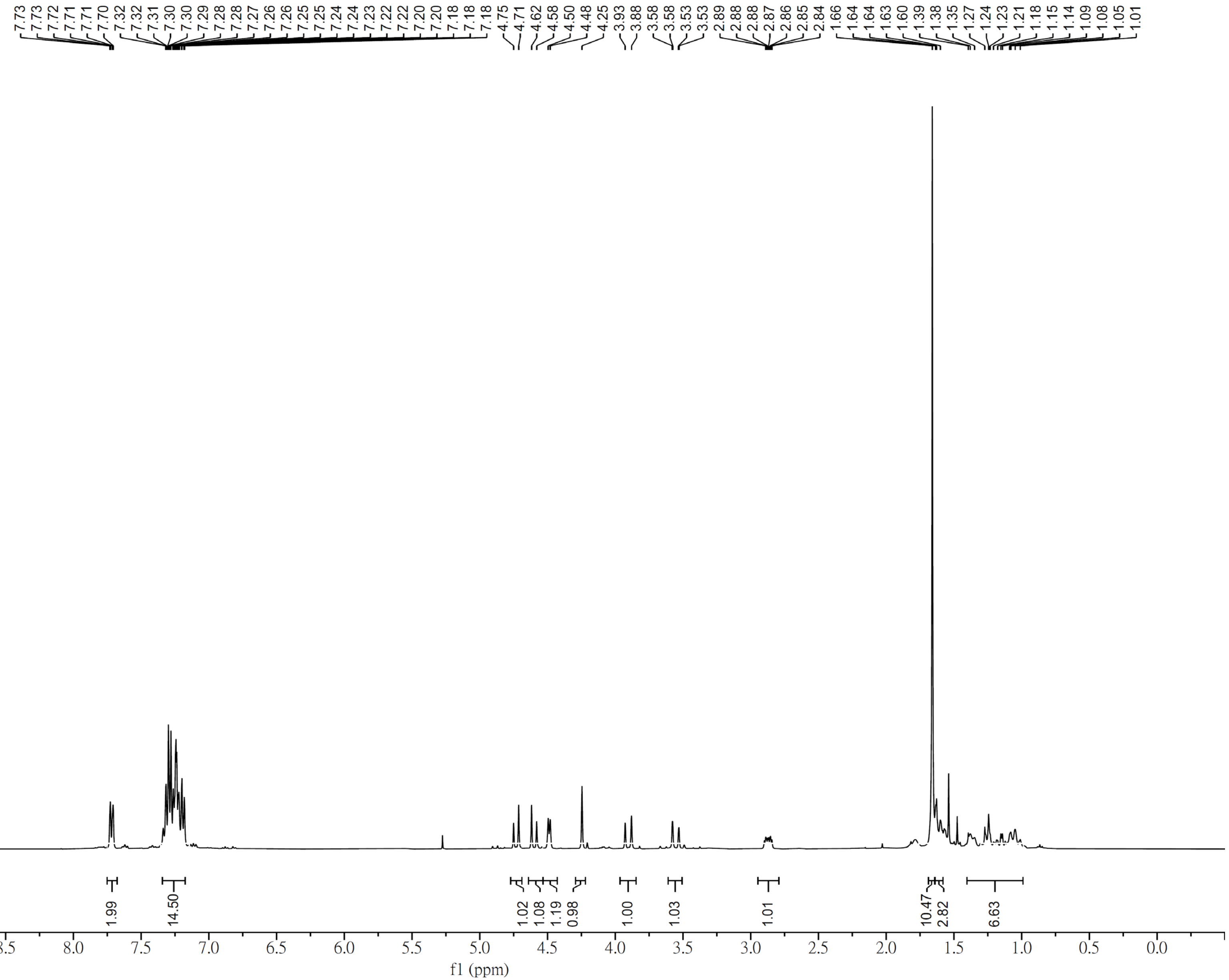

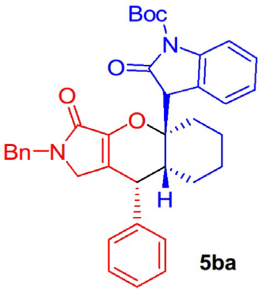

**5ba**

($^{13}C\{^{1}H\}$ NMR, 101 MHz, $CDCl_3$)

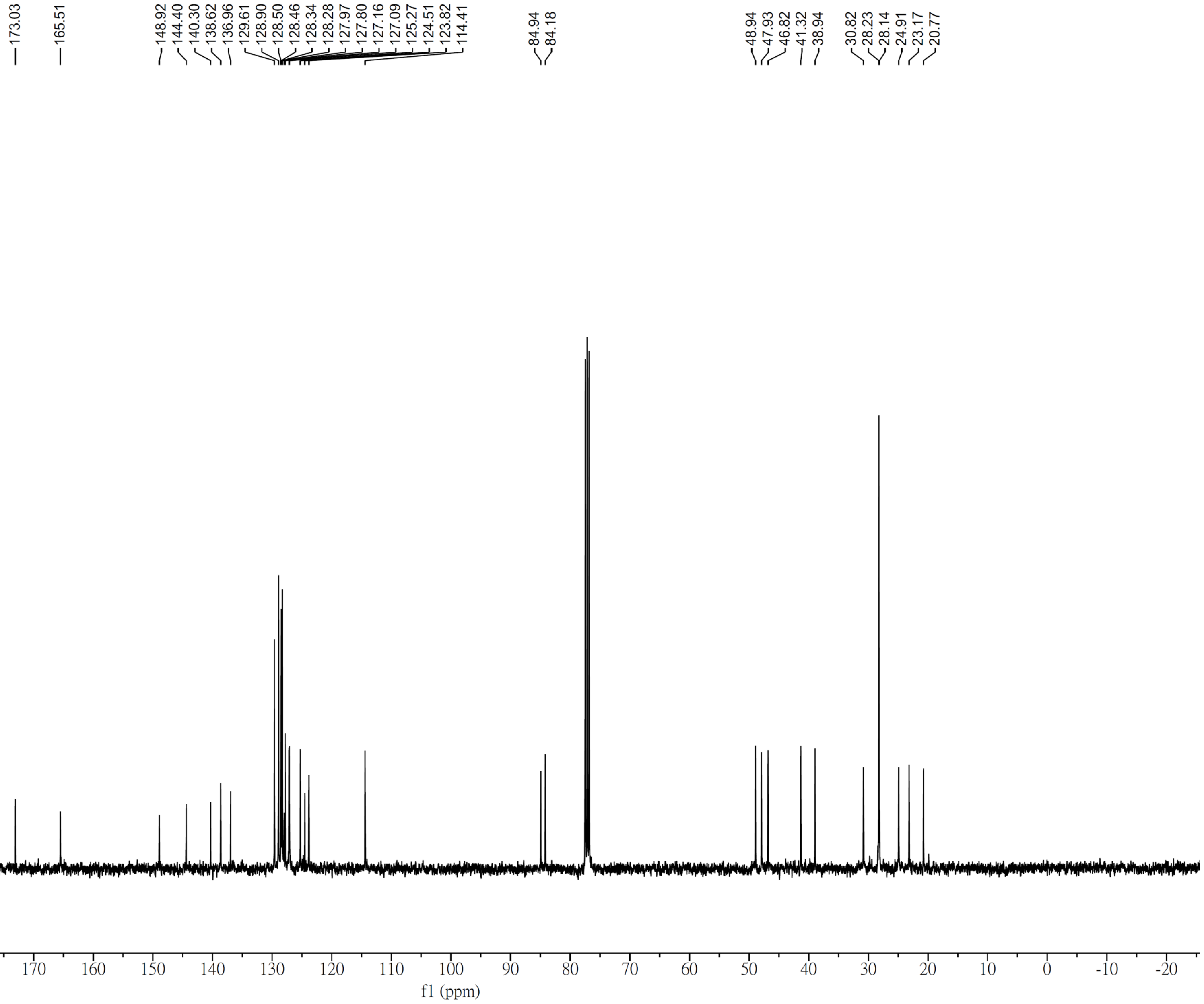

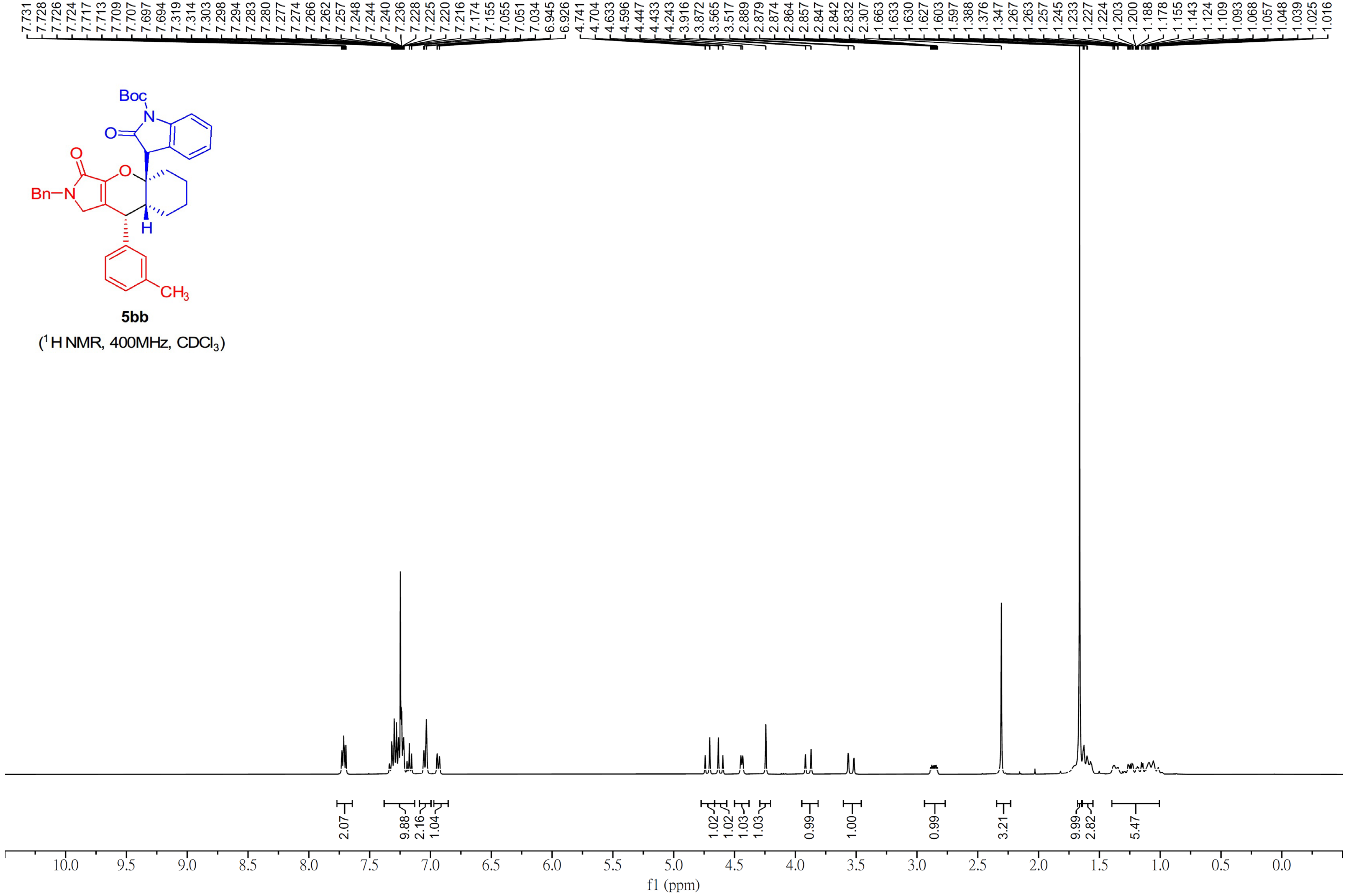

Boc
N
O
O
O
Bn
N
H
$CH_3$
5bb
($^1$H NMR, 400MHz, $CDCl_3$)
7.731
7.728
7.726
7.724
7.717
7.713
7.709
7.707
7.697
7.694
7.319
7.314
7.303
7.298
7.294
7.283
7.280
7.277
7.274
7.266
7.262
7.257
7.248
7.244
7.240
7.236
7.228
7.225
7.220
7.216
7.174
7.155
7.055
7.051
7.034
6.945
6.926
4.741
4.704
4.633
4.596
4.447
4.433
4.243
3.916
3.872
3.565
3.517
2.889
2.879
2.874
2.864
2.857
2.847
2.842
2.832
2.307
1.663
1.633
1.630
1.627
1.603
1.597
1.388
1.376
1.347
1.267
1.263
1.257
1.245
1.233
1.227
1.224
1.203
1.200
1.188
1.178
1.155
1.143
1.124
1.109
1.093
1.068
1.057
1.048
1.039
1.025
1.016
2.07
9.88
2.16
1.04
1.02
1.02
1.03
1.03
0.99
1.00
0.99
3.21
9.99
2.82
5.47
10.0
9.5
9.0
8.5
8.0
7.5
7.0
6.5
6.0
5.5
5.0
4.5
4.0
3.5
3.0
2.5
2.0
1.5
1.0
0.5
0.0
f1 (ppm)

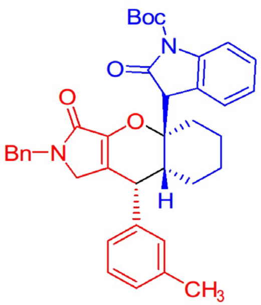

**5bb**

($^{13}C\{^1H\}$ NMR, 101 MHz, $CDCl_3$)

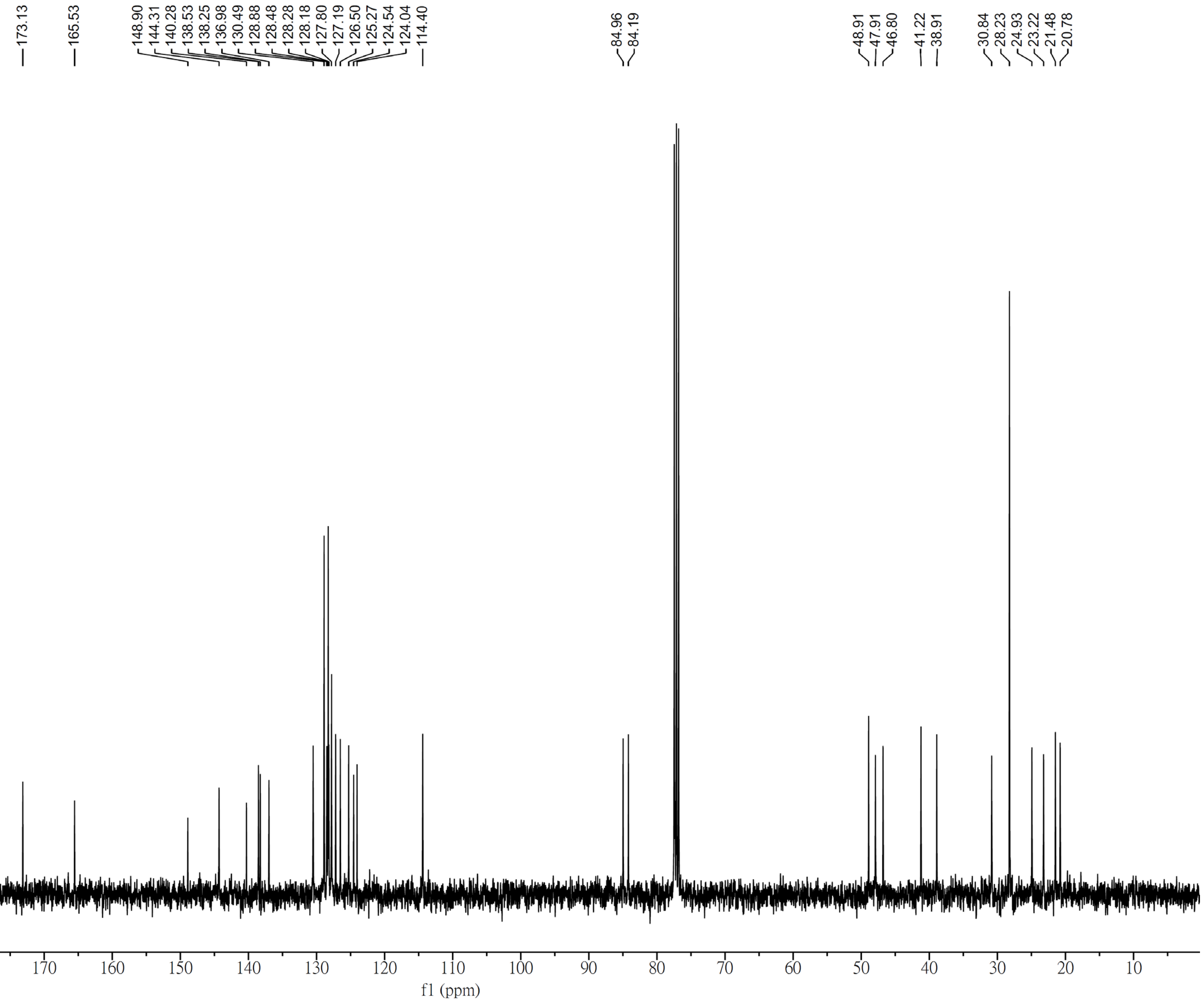

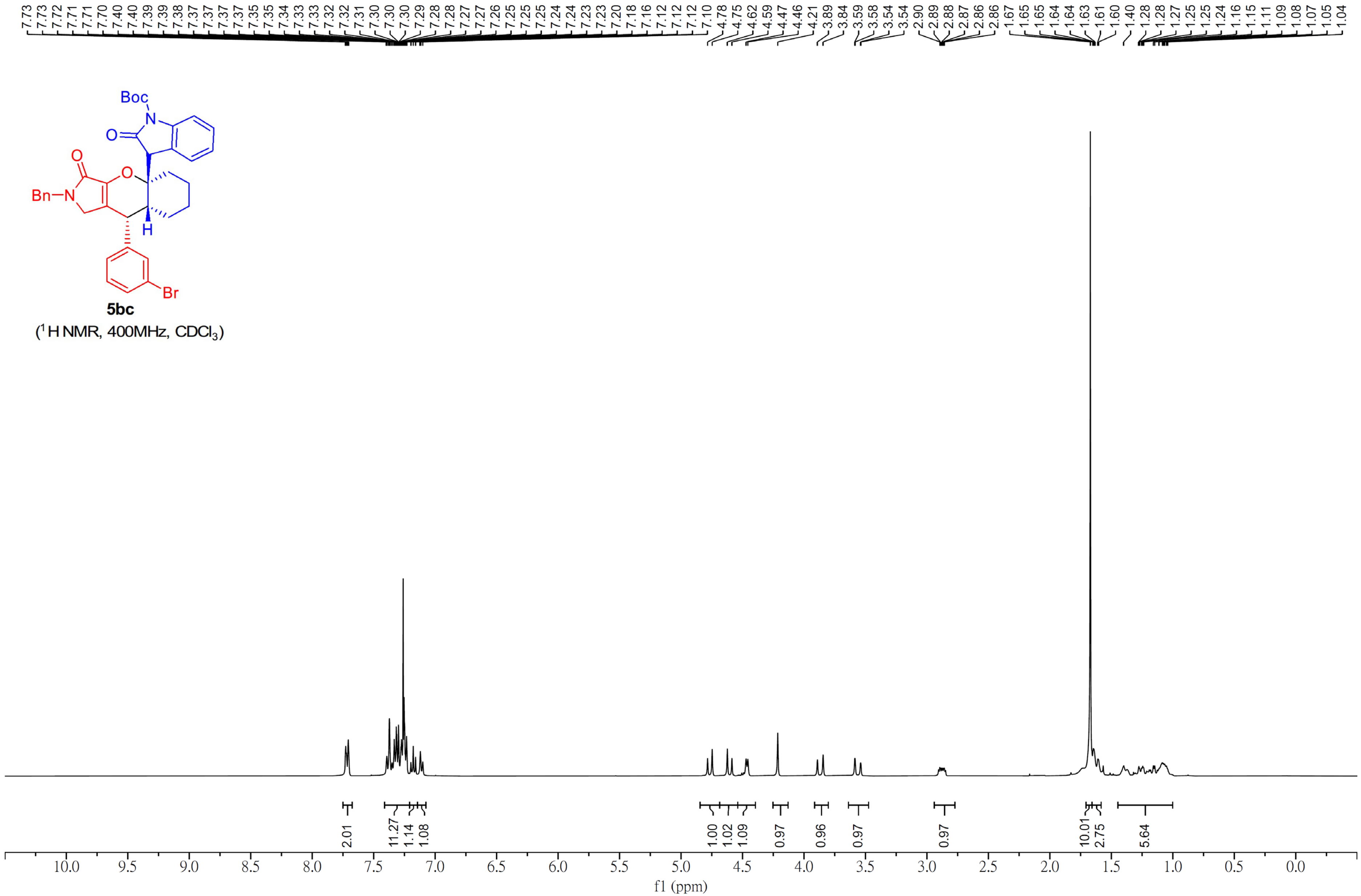
Boc
N
O
O
O
Bn
N
H
Br
5bc
(1H NMR, 400MHz, CDCl3)
f1 (ppm)

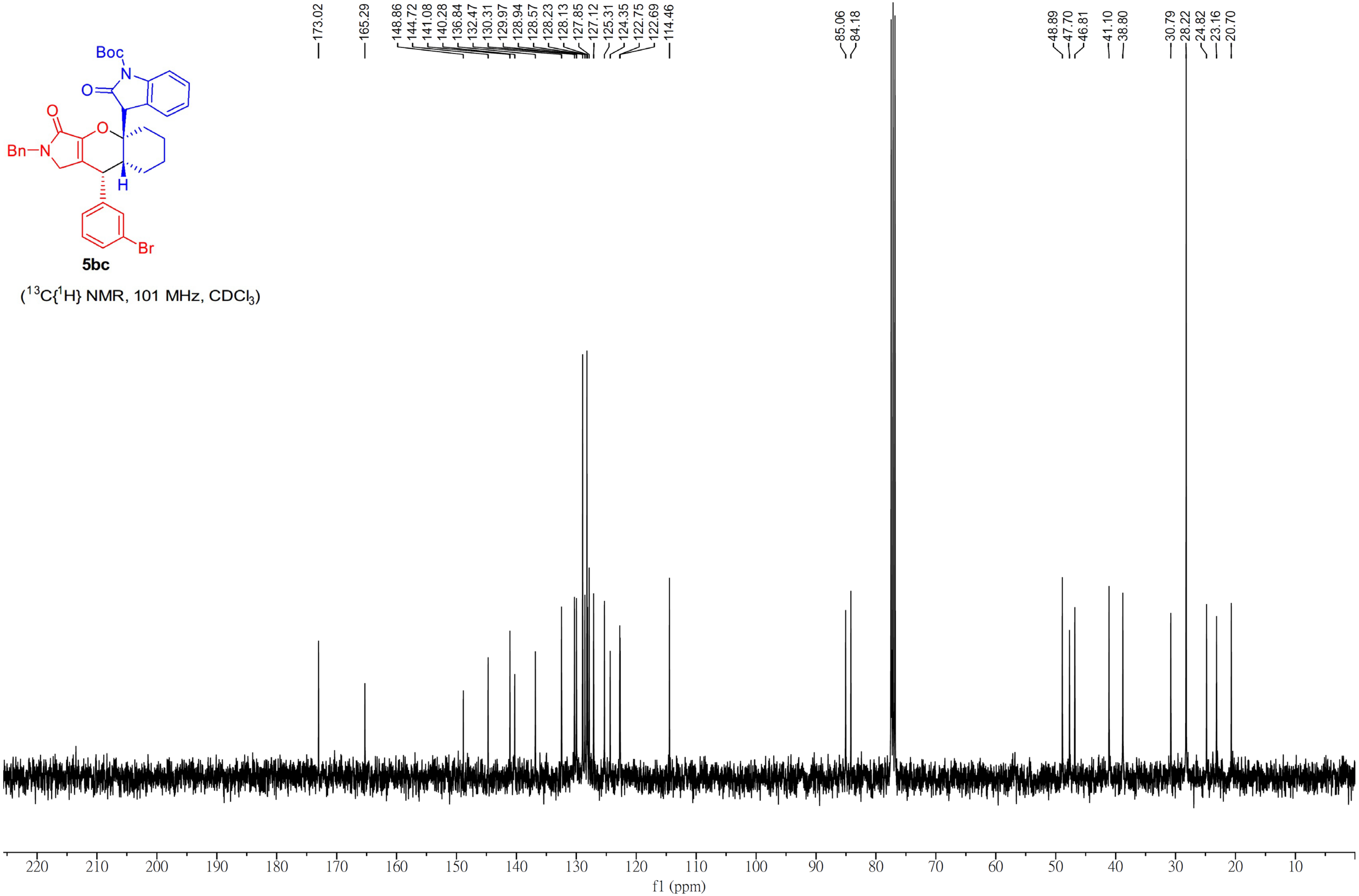


**5bc**

($^{13}C\{^{1}H\}$ NMR, 101 MHz, $CDCl_3$)

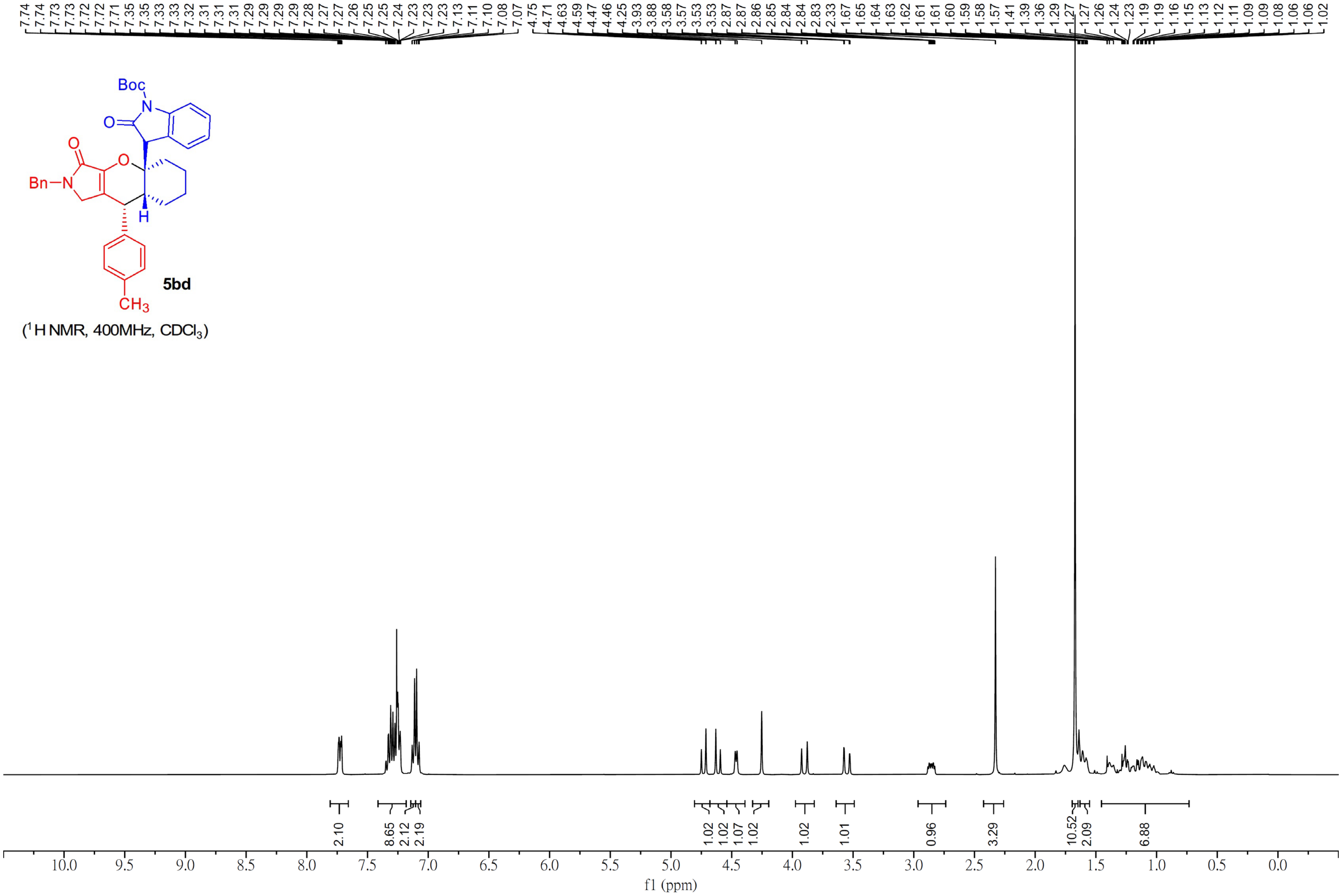
7.74 7.74 7.73 7.73 7.72 7.72 7.71 7.35 7.35 7.33 7.33 7.32 7.31 7.31 7.31 7.29 7.29 7.29 7.29 7.28 7.27 7.27 7.26 7.25 7.25 7.24 7.23 7.23 7.23 7.13 7.11 7.10 7.08 7.07
4.75 4.71 4.63 4.59 4.47 4.46 4.25 3.93 3.88 3.58 3.57 3.53 3.53 2.87 2.87 2.86 2.85 2.84 2.84 2.83 2.33 1.67 1.65 1.64 1.63 1.62 1.61 1.61 1.60 1.59 1.58 1.57 1.41 1.39 1.36 1.29 1.27 1.27 1.26 1.24 1.23 1.19 1.19 1.16 1.15 1.13 1.12 1.11 1.09 1.09 1.08 1.06 1.06 1.02
Boc
N
O
O
O
Bn
N
H
CH3
5bd
(1 H NMR, 400MHz, CDCl3)
2.10 8.65 2.12 2.19 1.02 1.02 1.07 1.02 1.02 1.01 0.96 3.29 10.52 2.09 6.88
10.0 9.5 9.0 8.5 8.0 7.5 7.0 6.5 6.0 5.5 5.0 4.5 4.0 3.5 3.0 2.5 2.0 1.5 1.0 0.5 0.0
f1 (ppm)

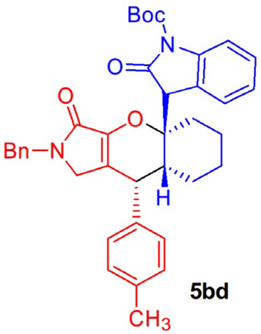


**5bd**

($^{13}C\{^1H\}$ NMR, 101 MHz, $CDCl_3$)

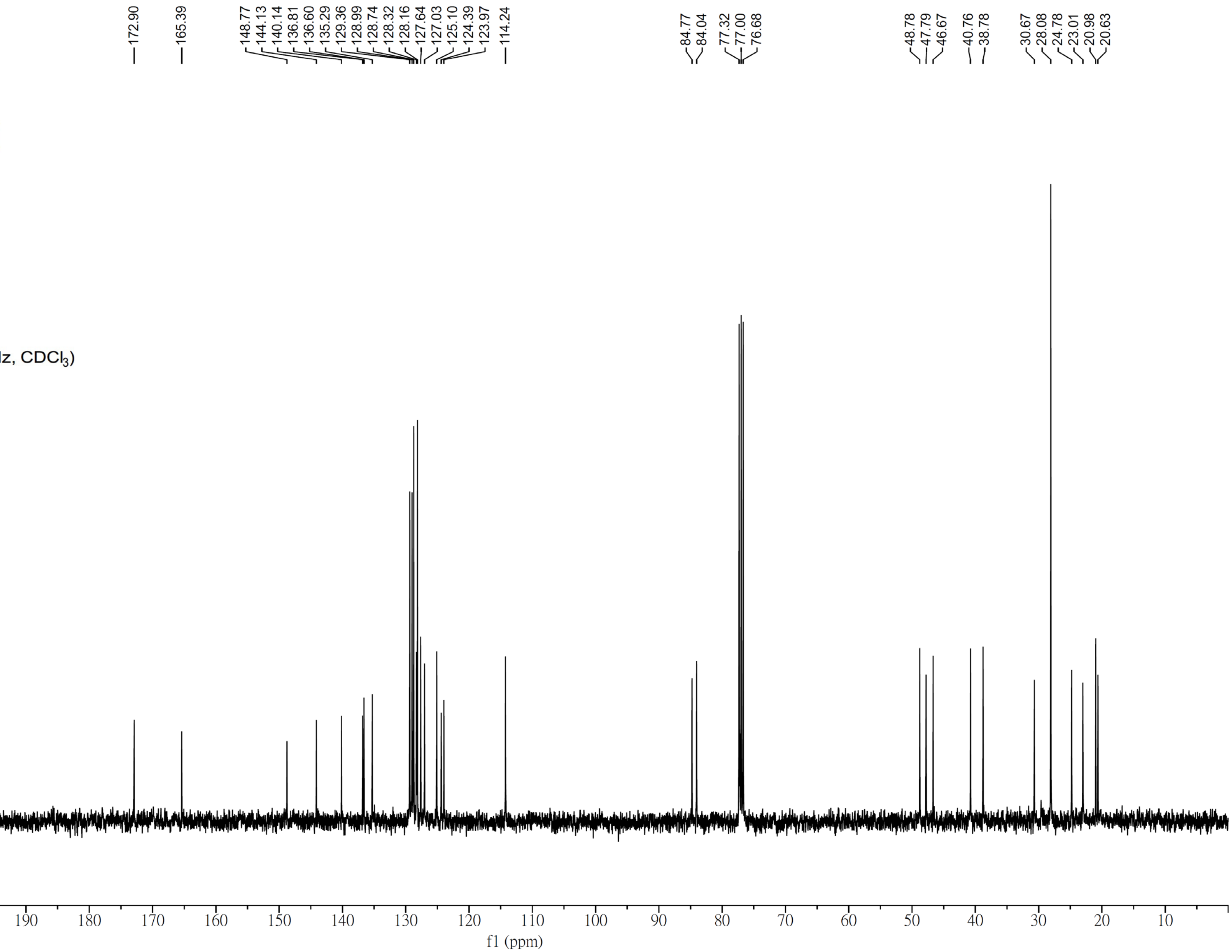

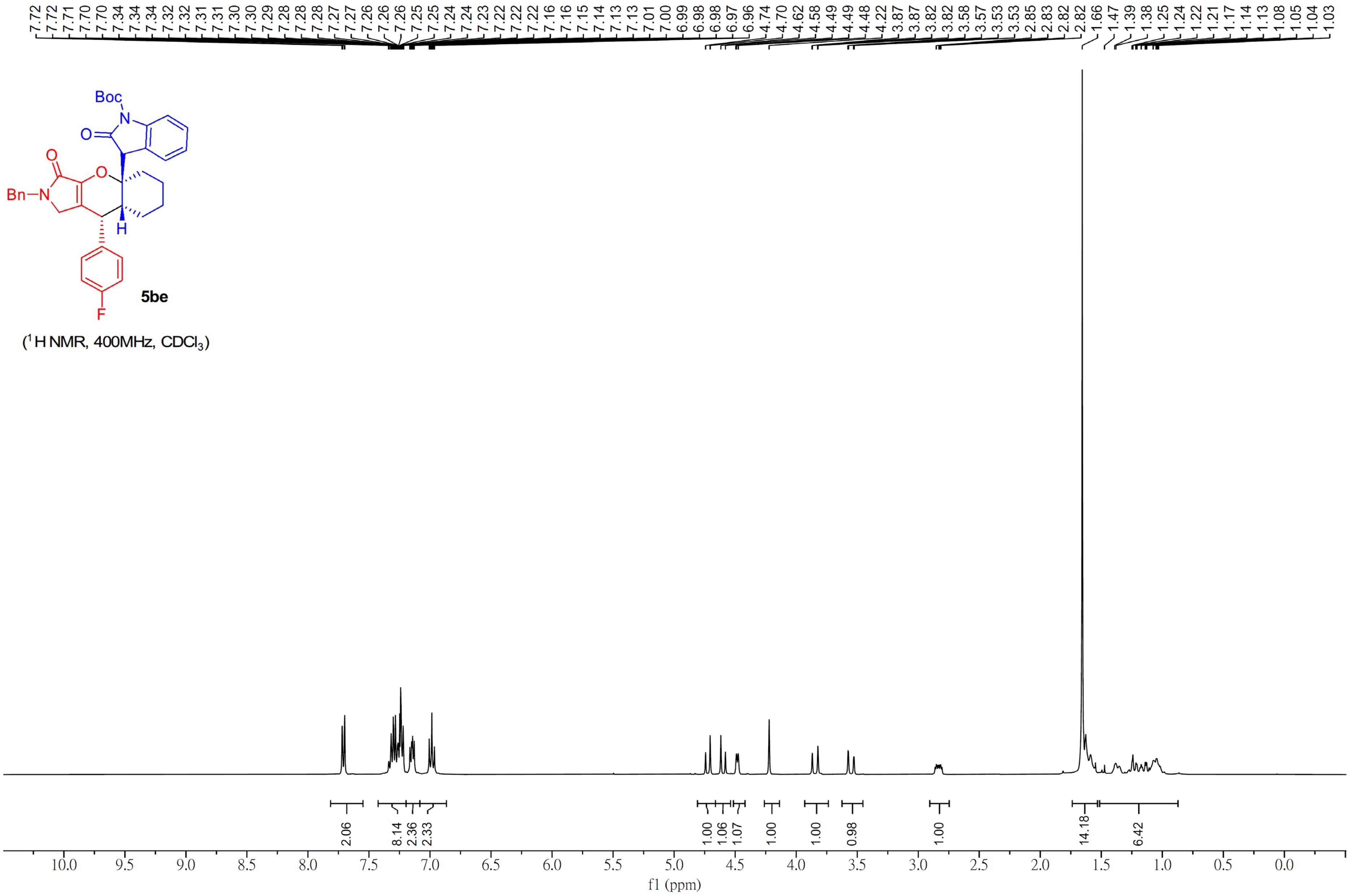
7.72 7.72 7.71 7.70 7.70 7.34 7.34 7.34 7.32 7.32 7.31 7.31 7.30 7.30 7.29 7.28 7.28 7.28 7.27 7.27 7.26 7.26 7.26 7.25 7.25 7.24 7.24 7.23 7.22 7.22 7.22 7.16 7.16 7.15 7.14 7.13 7.13 7.01 7.00 6.99 6.98 6.98 6.97 6.96 4.74 4.70 4.62 4.58 4.49 4.49 4.48 4.22 3.87 3.87 3.82 3.82 3.58 3.57 3.53 3.53 2.85 2.83 2.82 2.82 1.66 1.47 1.39 1.38 1.25 1.24 1.22 1.21 1.17 1.14 1.13 1.08 1.05 1.04 1.03
Boc
N
O
O
Bn
N
O
H
F
5be
(1H NMR, 400MHz, CDCl3)
2.06
8.14
2.36
2.33
1.00
1.06
1.07
1.00
1.00
0.98
1.00
14.18
6.42
10.0 9.5 9.0 8.5 8.0 7.5 7.0 6.5 6.0 5.5 5.0 4.5 4.0 3.5 3.0 2.5 2.0 1.5 1.0 0.5 0.0
f1 (ppm)

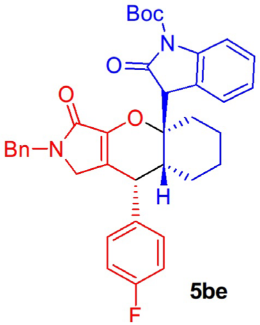


**5be**

($^{13}C\{^{1}H\}$ NMR, 101 MHz, $CDCl_3$)

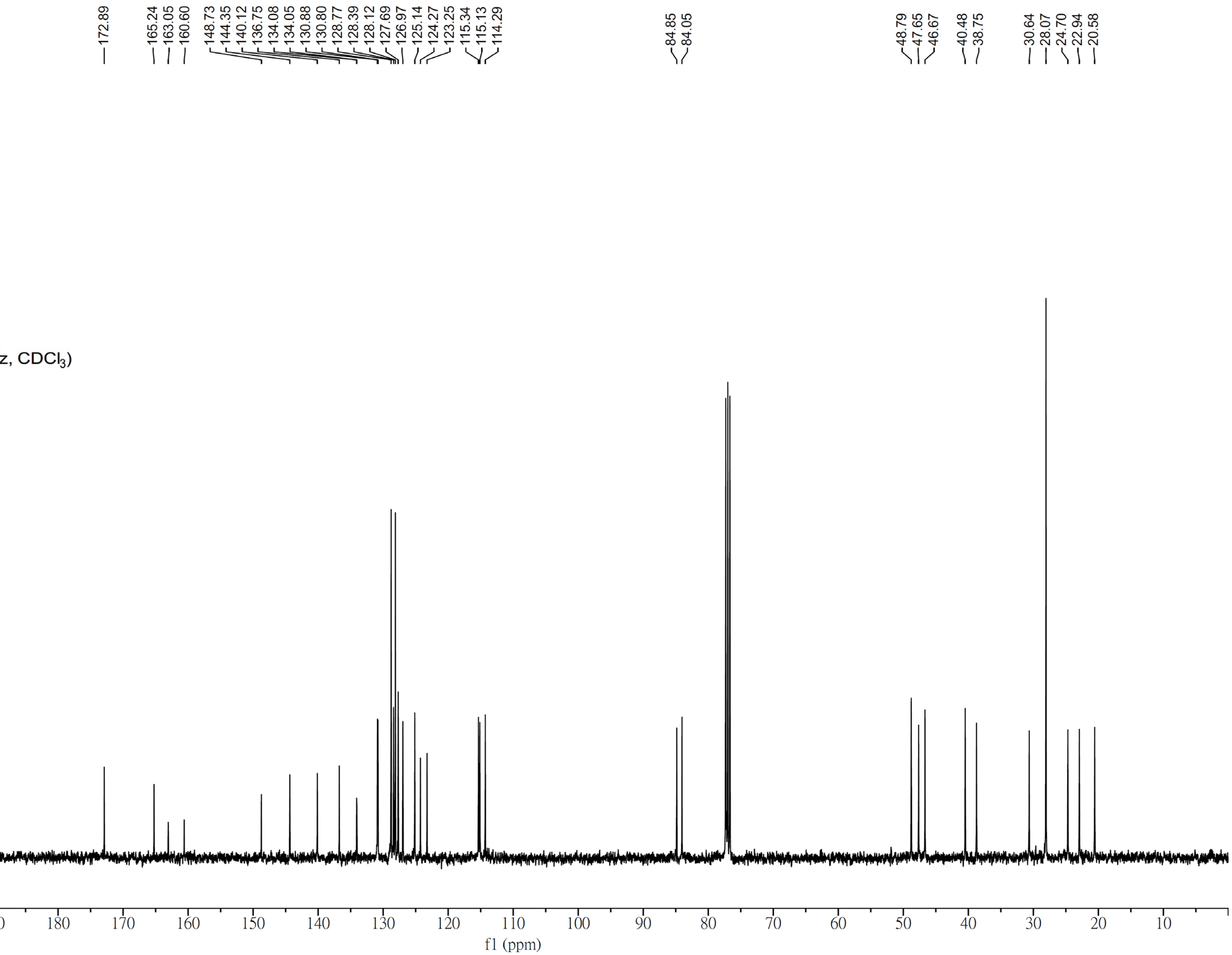

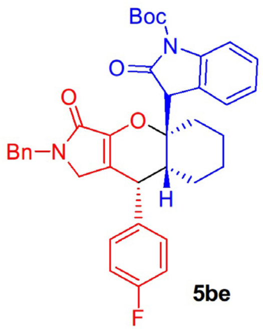

**5be**

($^{19}$F NMR, 376MHz, $CDCl_3$)

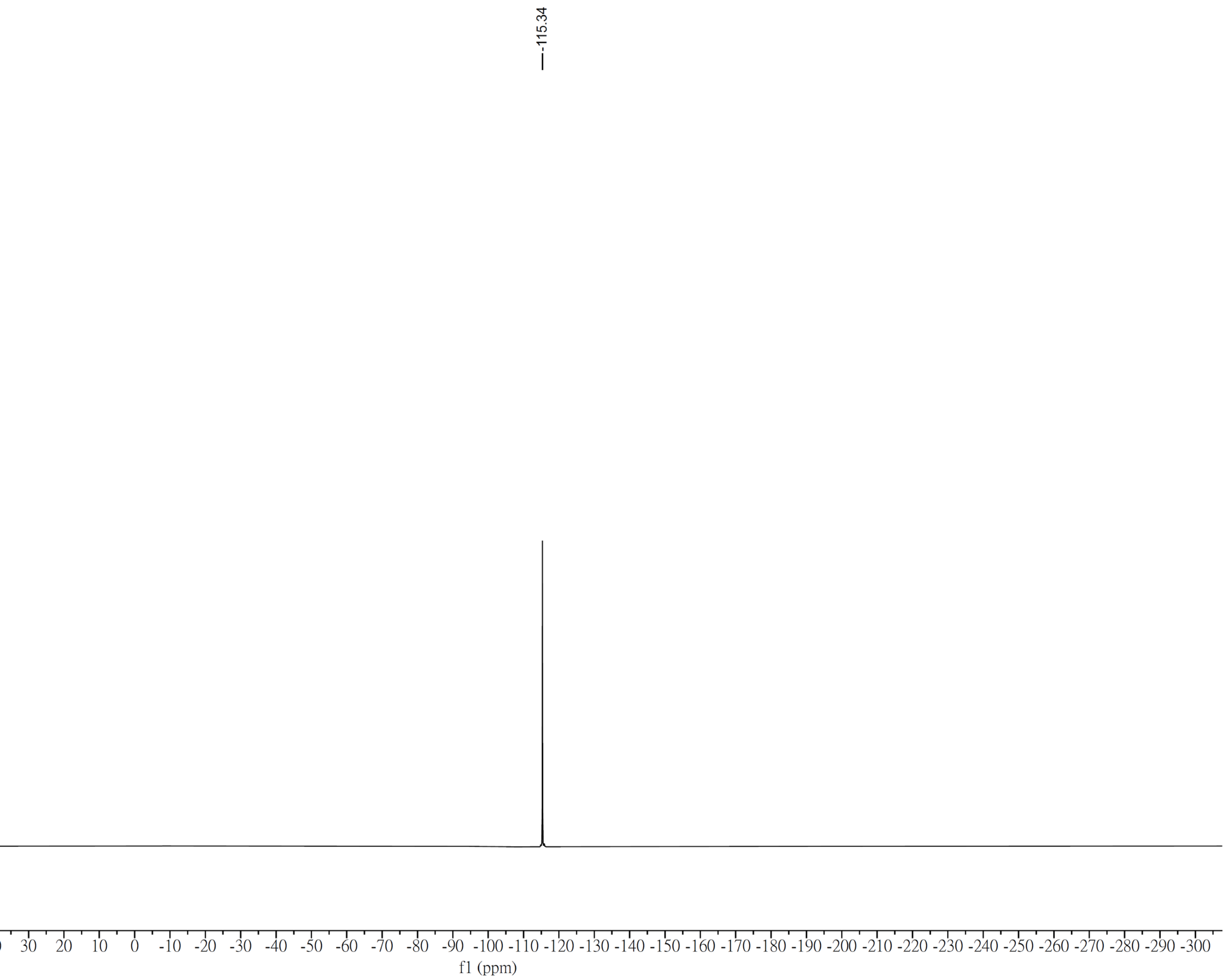

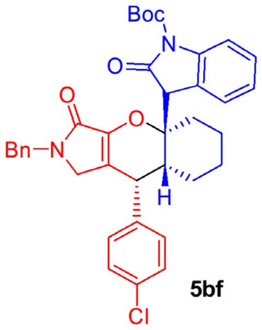


**5bf**

($^1$H NMR, 400MHz, $CDCl_3$)

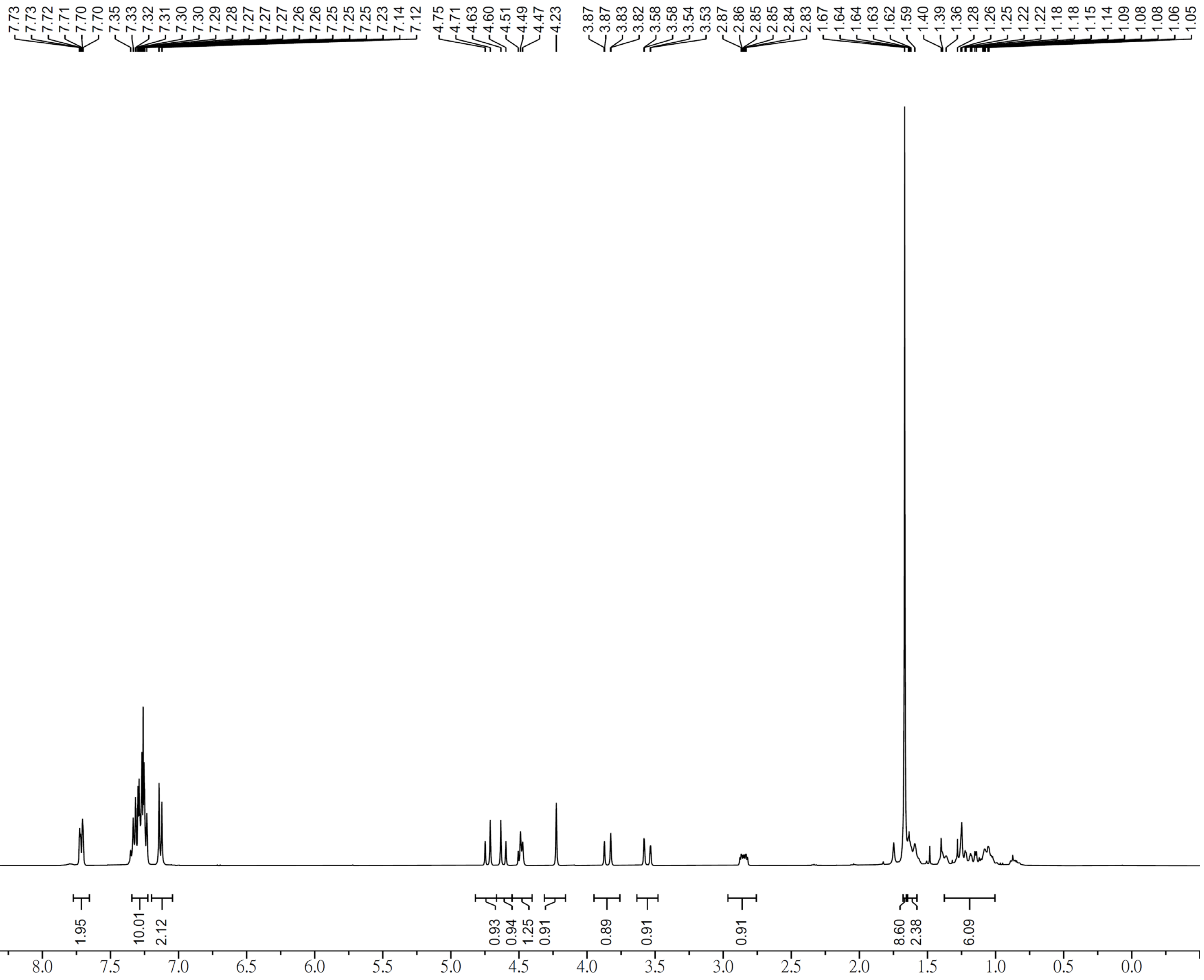

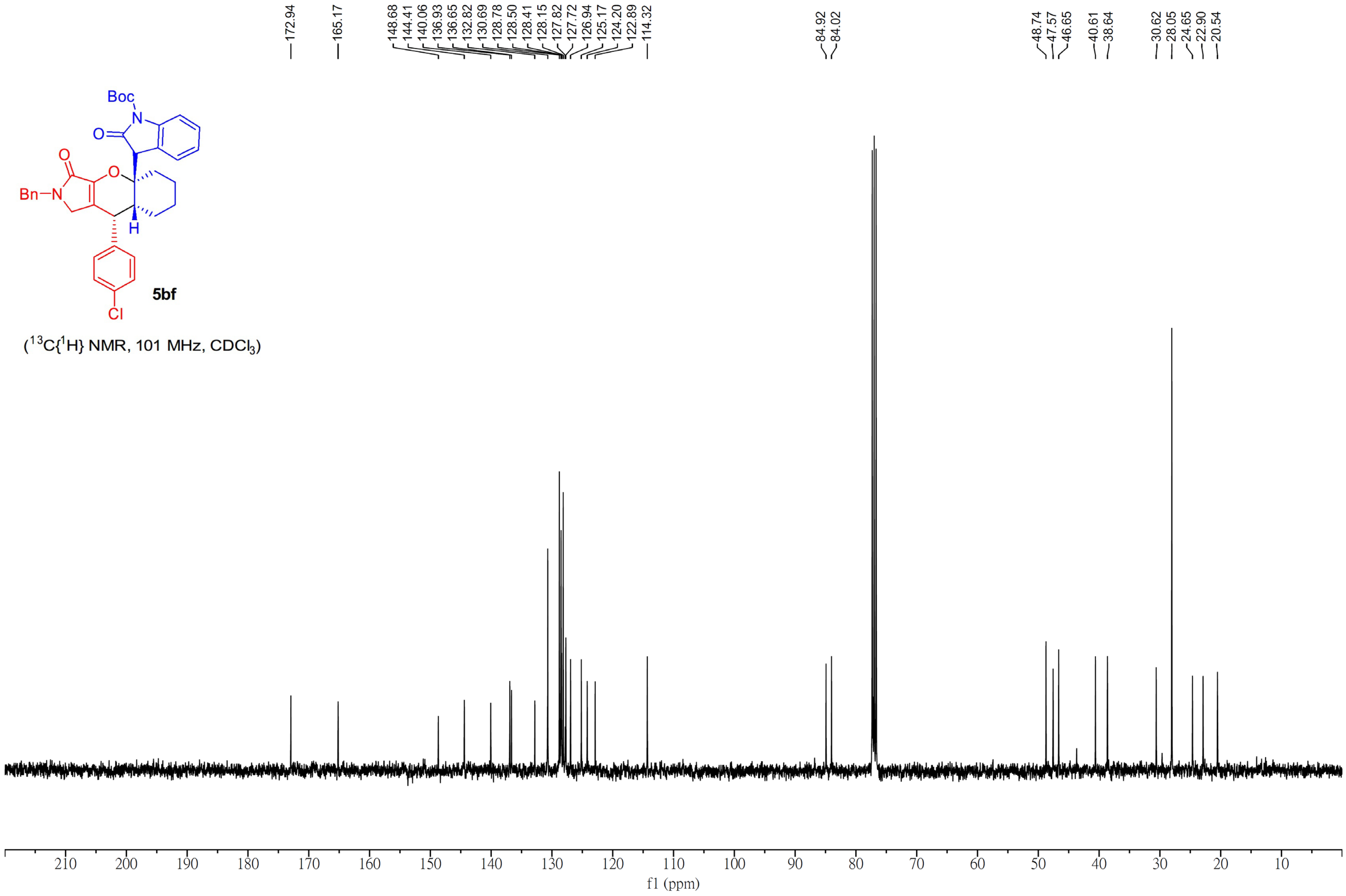
172.94
165.17
148.68
144.41
140.06
136.93
136.65
132.82
130.69
128.78
128.50
128.41
128.15
127.82
127.72
126.94
125.17
124.20
122.89
114.32
84.92
84.02
48.74
47.57
46.65
40.61
38.64
30.62
28.05
24.65
22.90
20.54
Boc
N
O
O
O
Bn
N
H
Cl
5bf
($^{13}$C{$^{1}$H} NMR, 101 MHz, $CDCl_3$)
210
200
190
180
170
160
150
140
130
120
110
100
90
80
70
60
50
40
30
20
10
f1 (ppm)

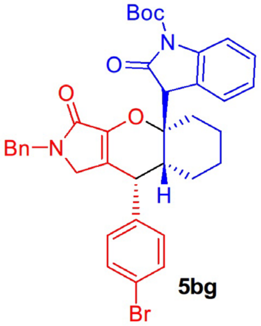


**5bg**

($^1$H NMR, 400MHz, $CDCl_3$)

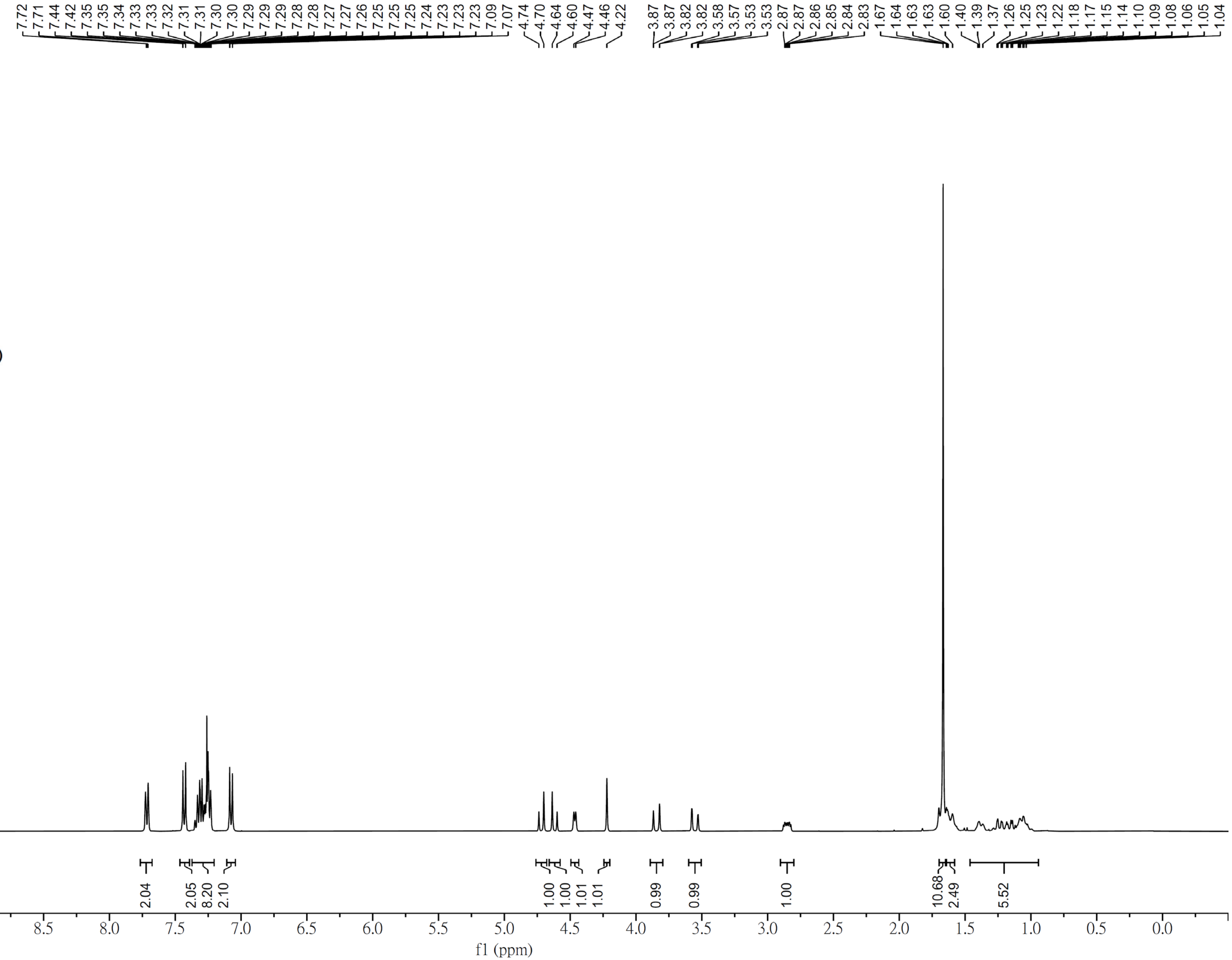

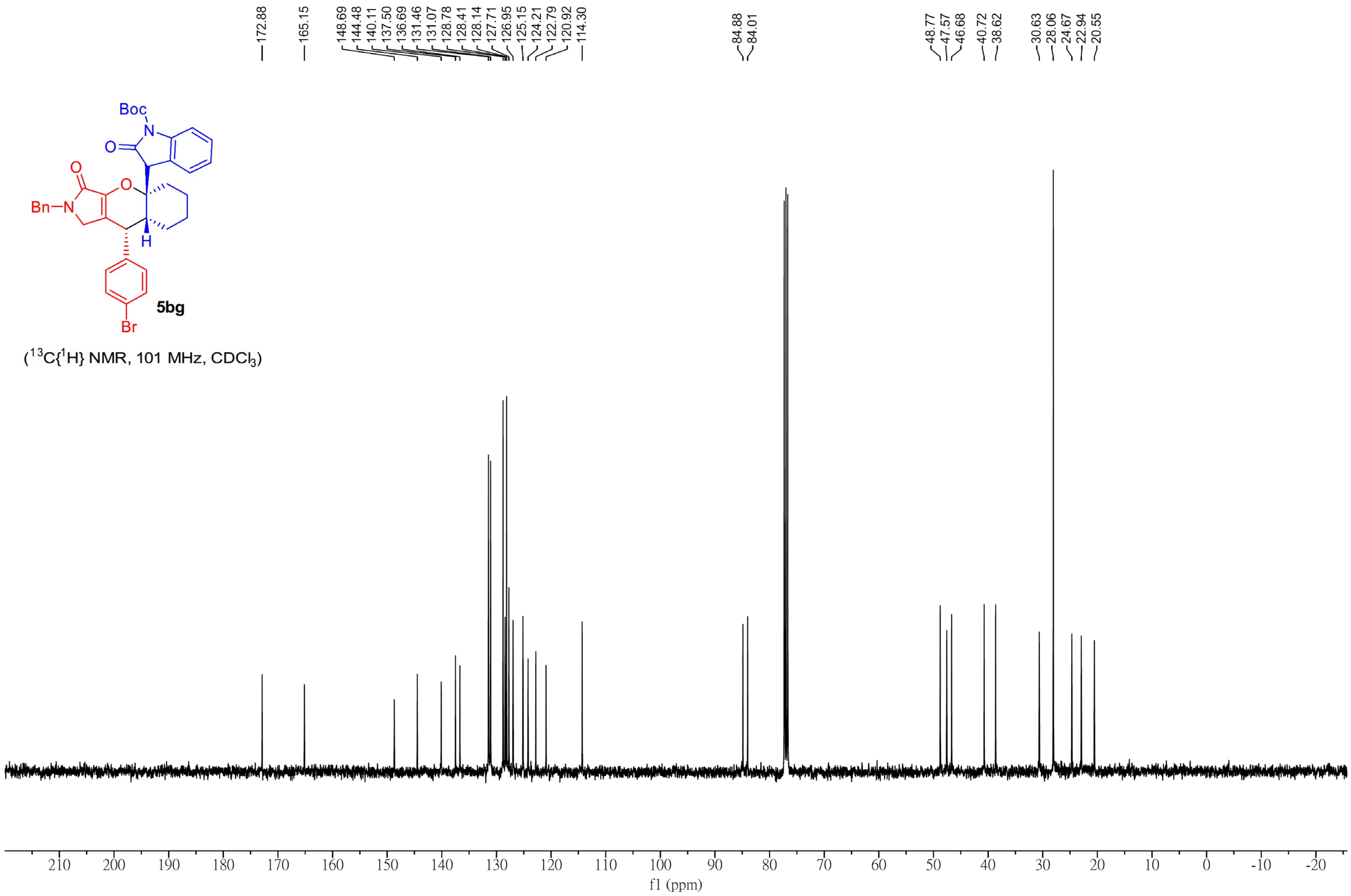


($^{13}C\{^{1}H\}$ NMR, 101 MHz, $CDCl_3$)

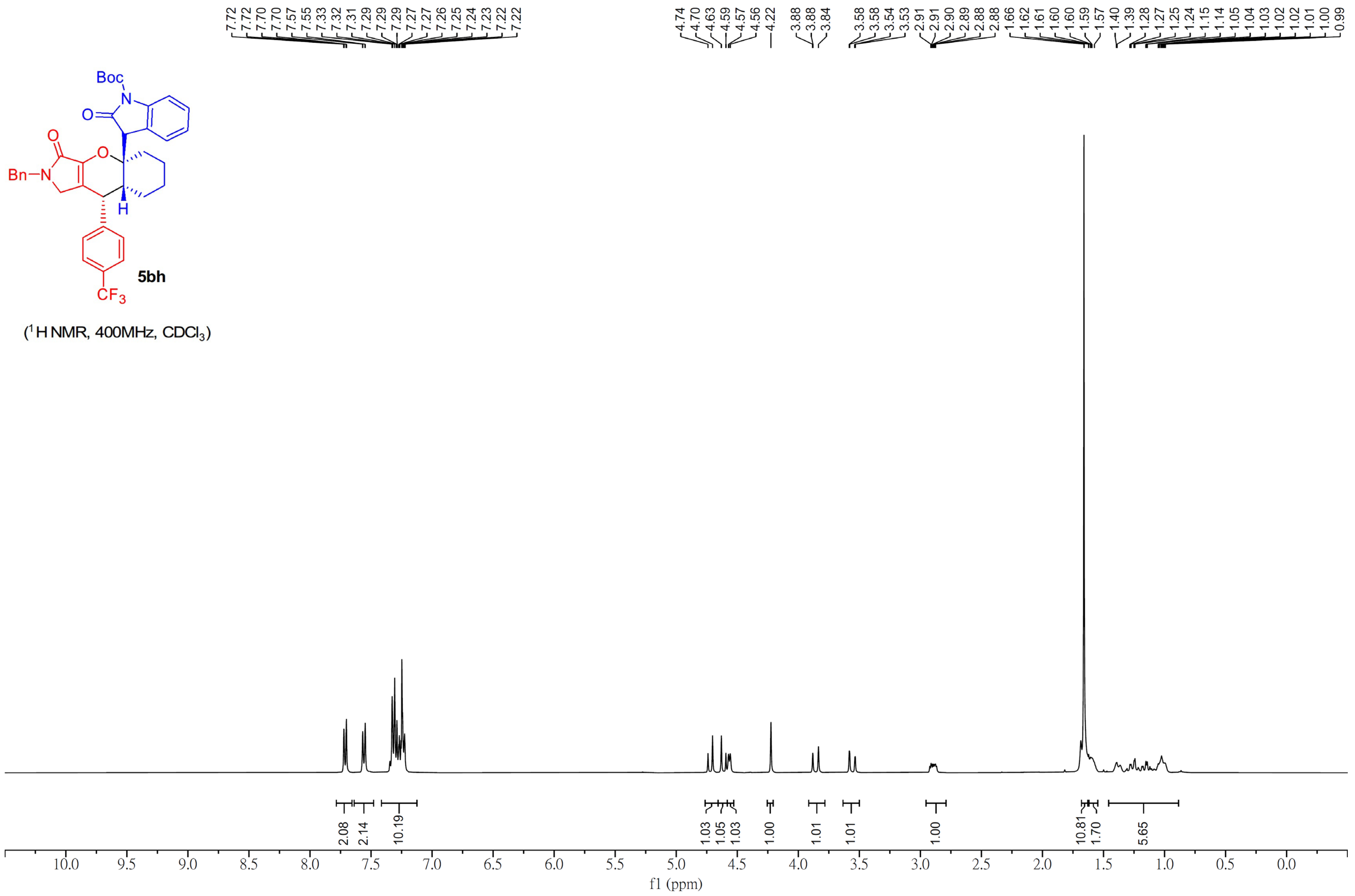

Boc
N
O
O
O
Bn
N
H
CF3
5bh
(1H NMR, 400MHz, CDCl3)
7.72 7.72 7.70 7.57 7.55 7.33 7.32 7.31 7.29 7.29 7.29 7.27 7.27 7.26 7.25 7.24 7.23 7.22 7.22
4.74 4.70 4.63 4.59 4.57 4.56 4.22 3.88 3.88 3.84 3.58 3.58 3.54 3.53 2.91 2.91 2.90 2.89 2.88 2.88 1.66 1.62 1.61 1.60 1.60 1.59 1.57 1.40 1.39 1.28 1.27 1.25 1.24 1.15 1.14 1.05 1.04 1.03 1.02 1.01 1.00 0.99
2.08 2.14 10.19 1.03 1.05 1.03 1.00 1.01 1.01 1.00 10.81 1.70 5.65
10.0 9.5 9.0 8.5 8.0 7.5 7.0 6.5 6.0 5.5 5.0 4.5 4.0 3.5 3.0 2.5 2.0 1.5 1.0 0.5 0.0
f1 (ppm)

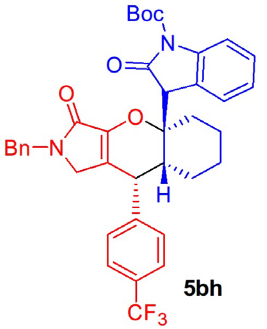


**5bh**

($^{13}C\{^{1}H\}$ NMR, 101 MHz, $CDCl_3$)

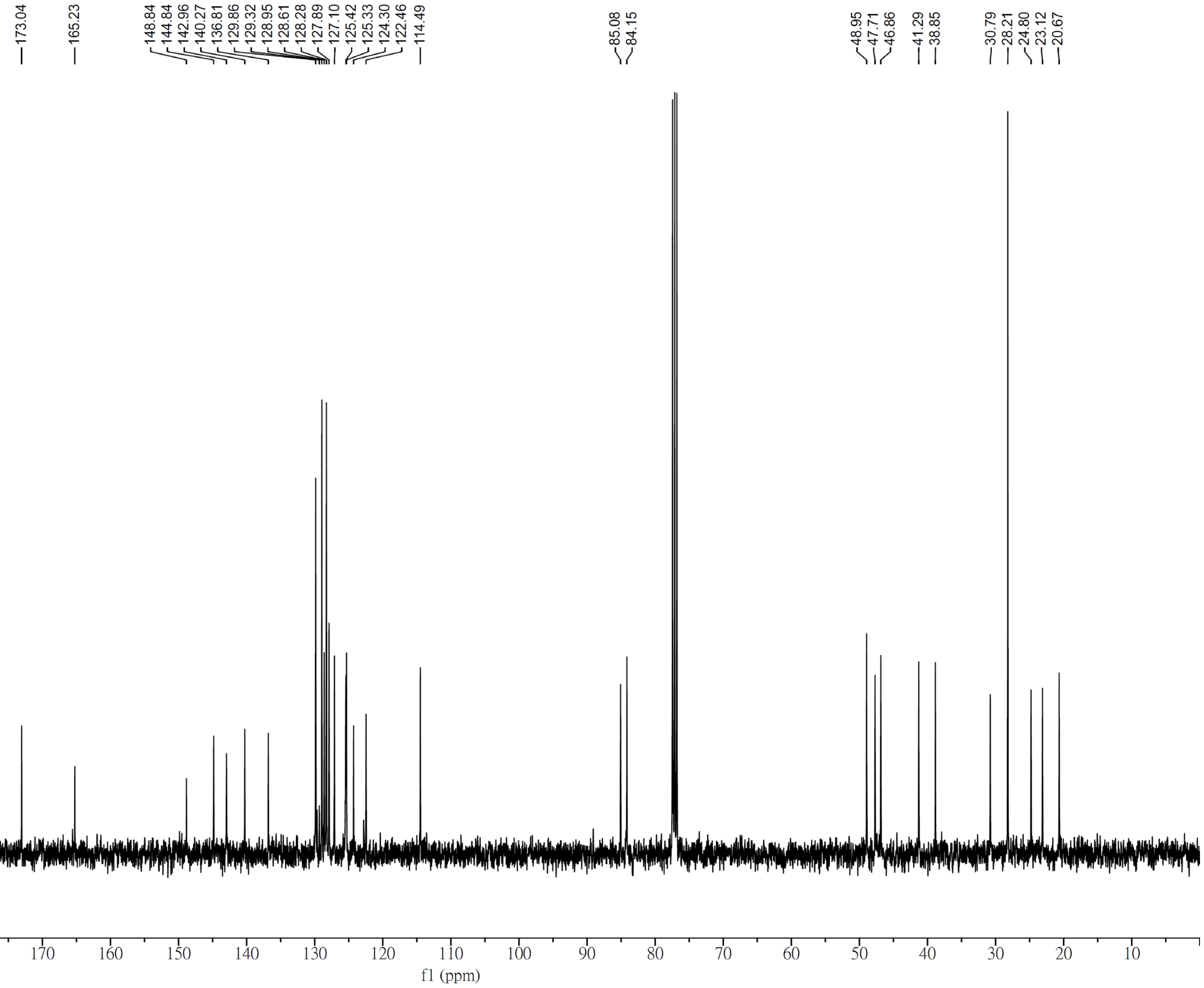

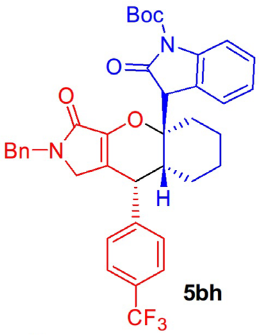


**5bh**

($^{19}F$ NMR, 376MHz, $CDCl_3$)

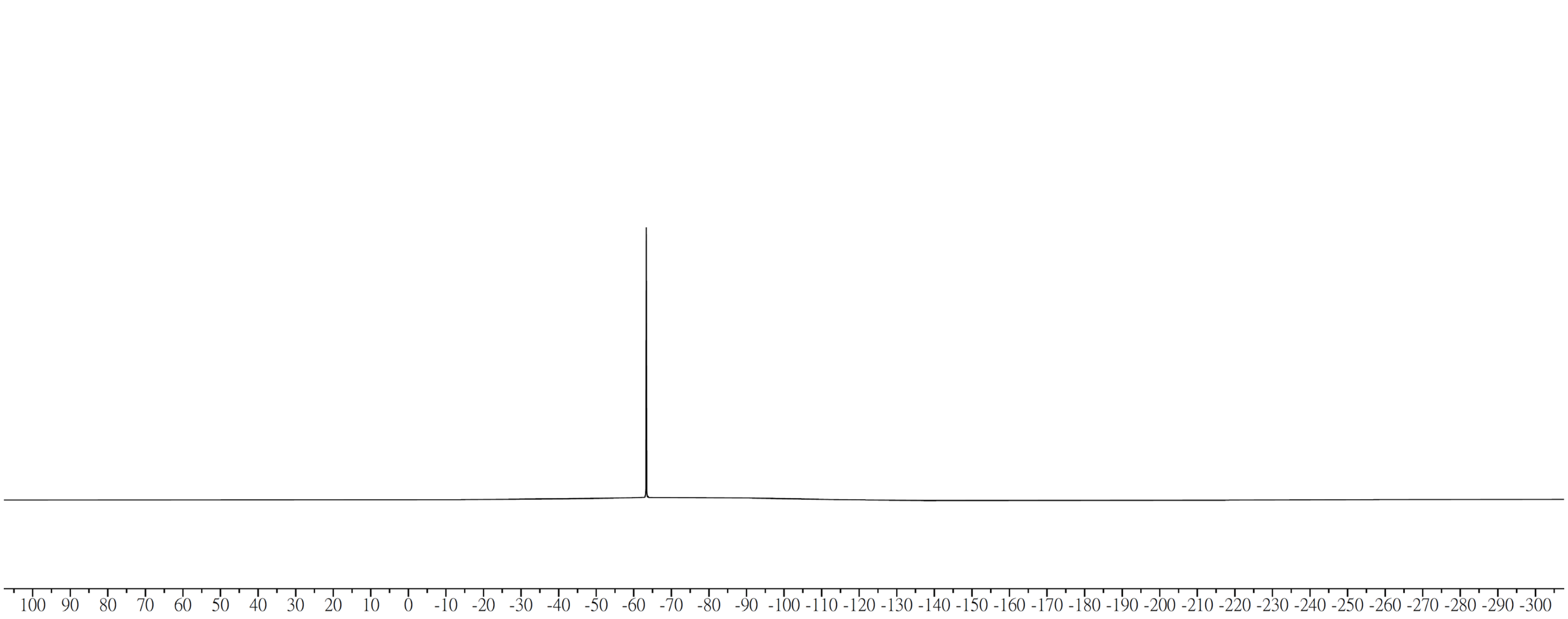

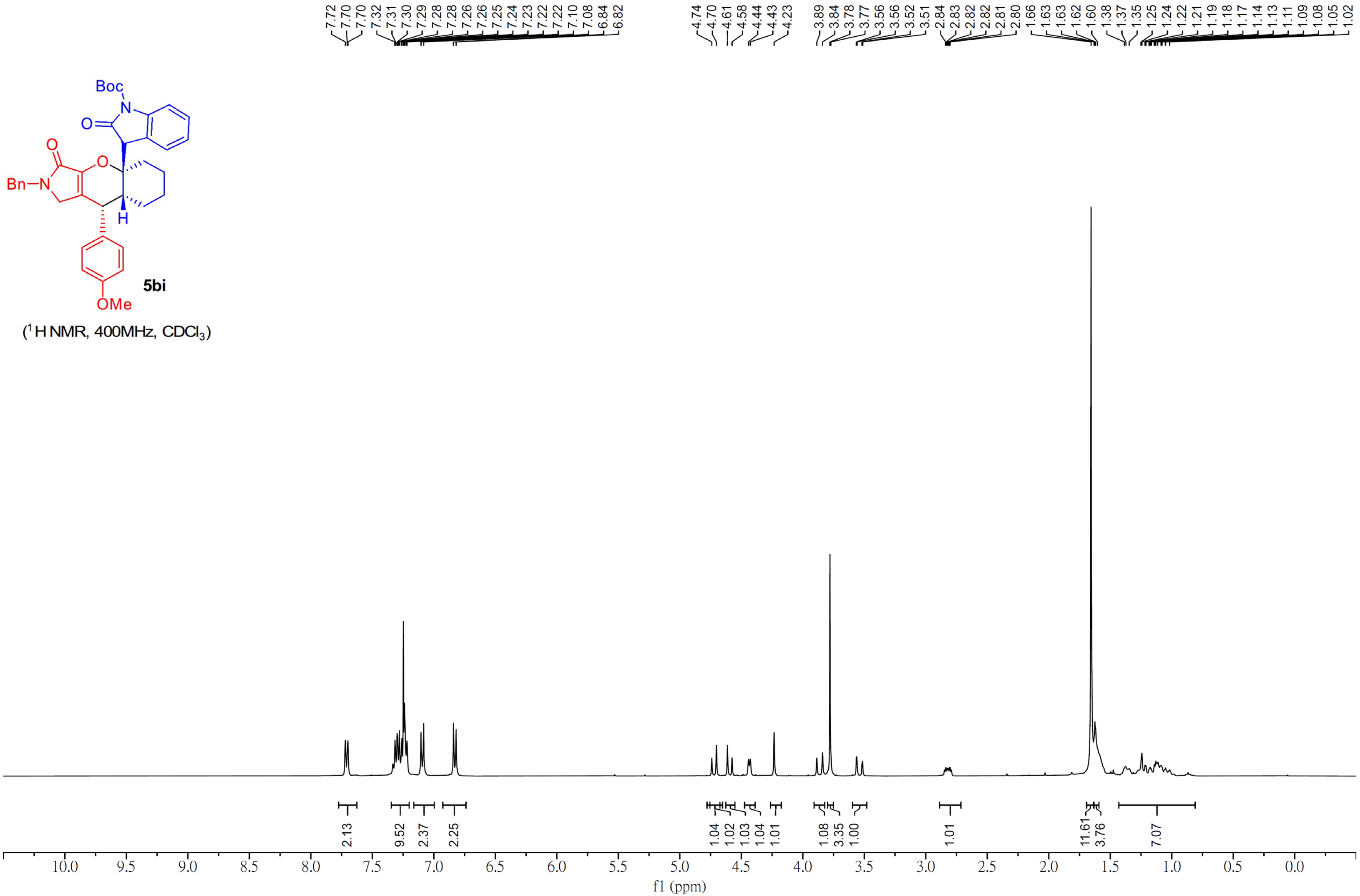

Boc
Bn
OMe
5bi
(1 H NMR, 400MHz, CDCl3)
f1 (ppm)

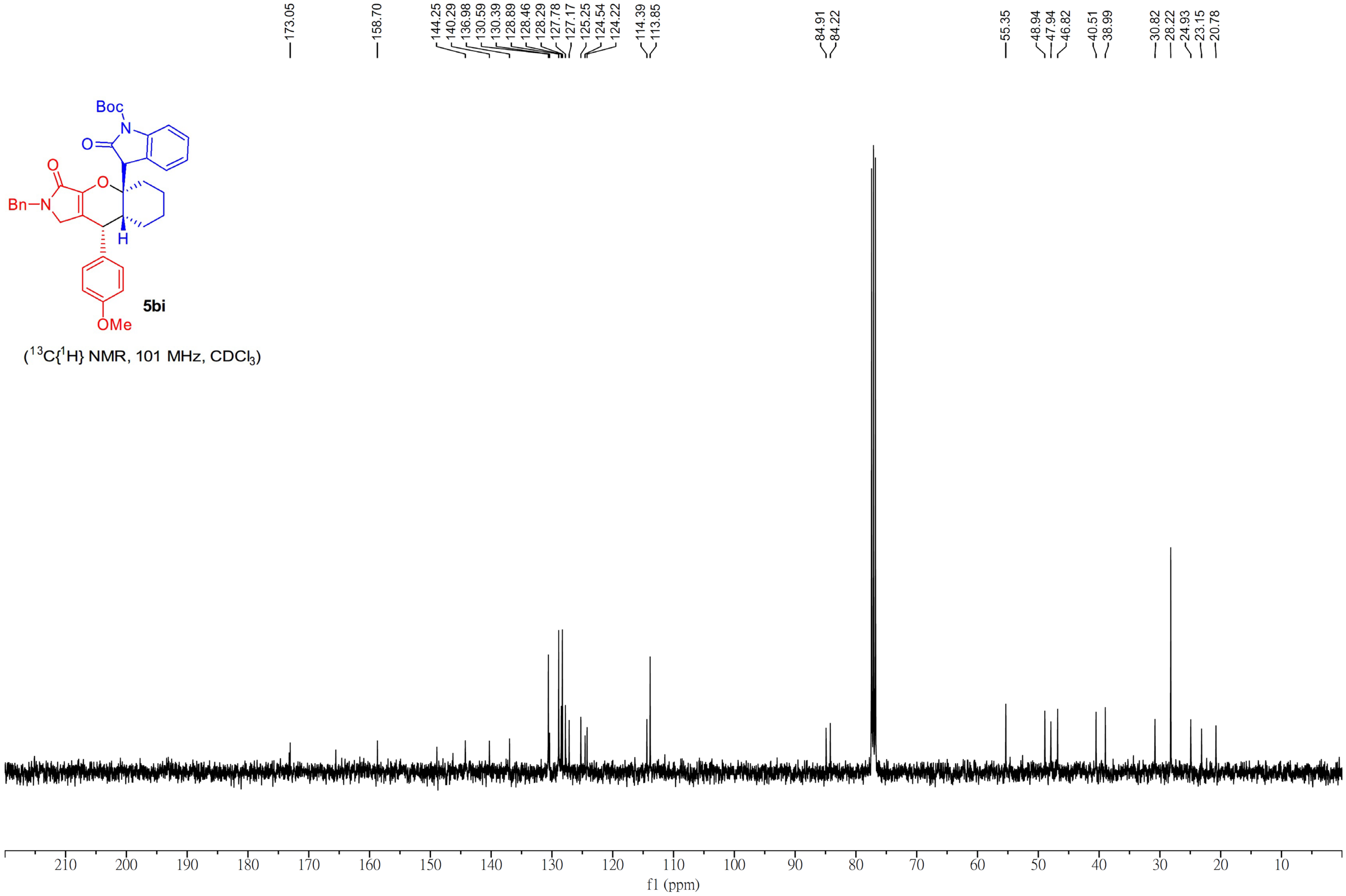
173.05
158.70
144.25
140.29
136.98
130.59
130.39
128.89
128.46
128.29
127.78
127.17
125.25
124.54
124.22
114.39
113.85
84.91
84.22
55.35
48.94
47.94
46.82
40.51
38.99
30.82
28.22
24.93
23.15
20.78
Boc
N
O
Bn
H
OMe
5bi
($^{13}C\{^1H\}$ NMR, 101 MHz, $CDCl_3$)
210 200 190 180 170 160 150 140 130 120 110 100 90 80 70 60 50 40 30 20 10
f1 (ppm)

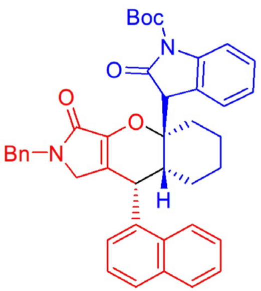


**5bj**

($^1$H NMR, 400MHz, $CDCl_3$)

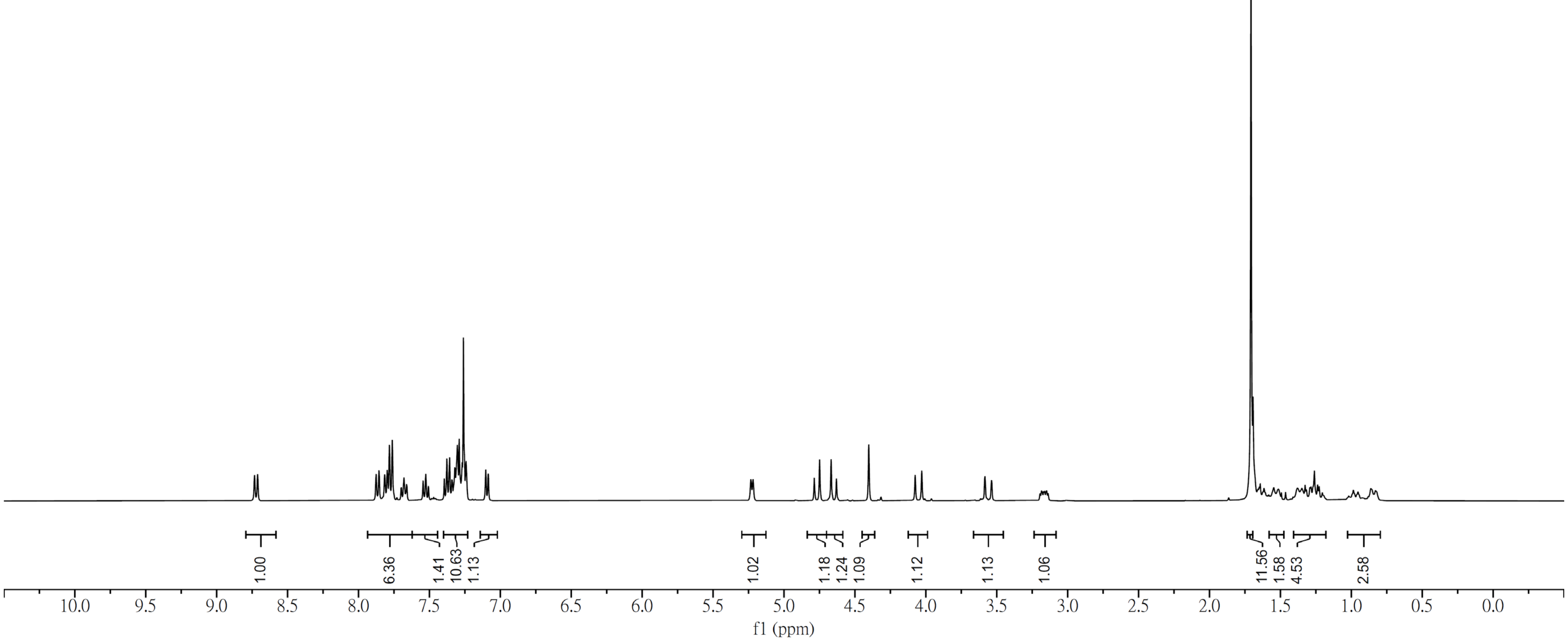

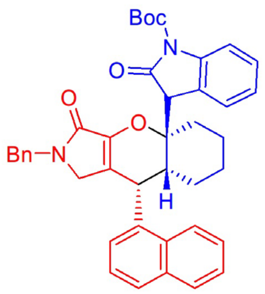

**5bj**

($^{13}C\{^1H\}$ NMR, 101 MHz, $CDCl_3$)

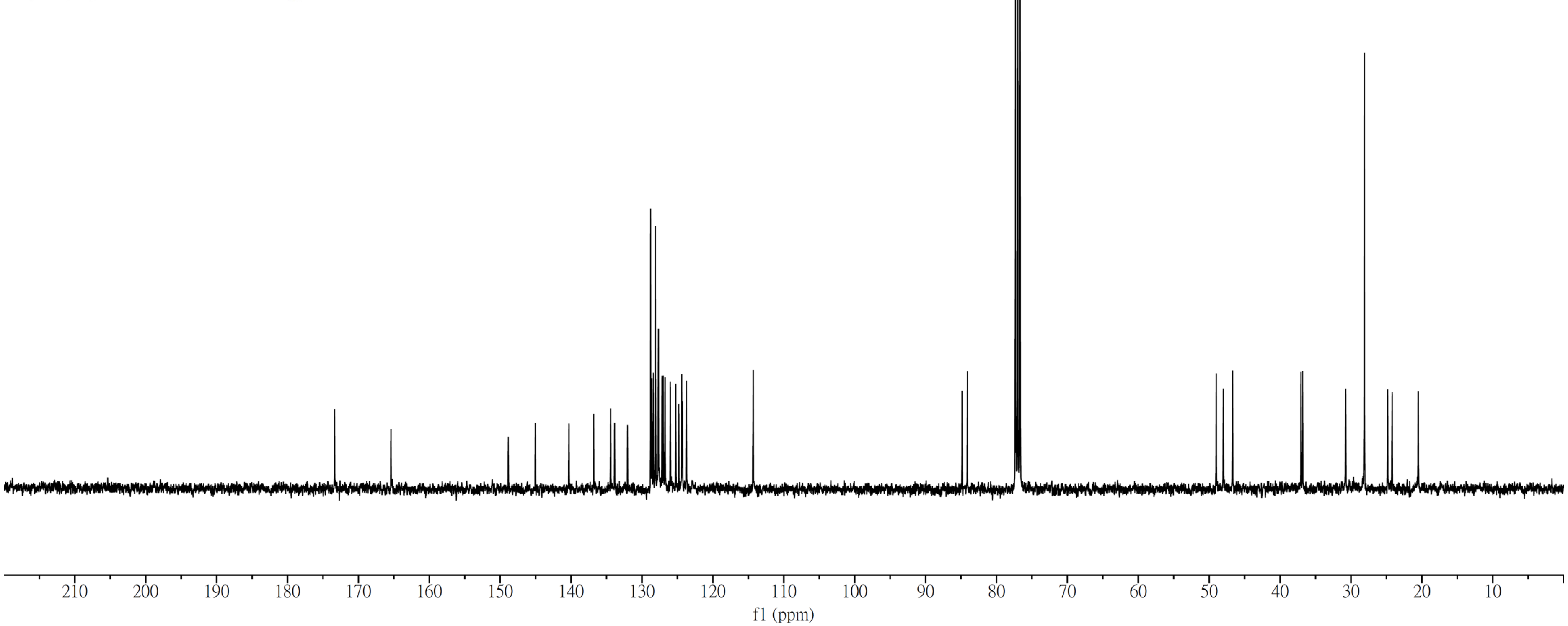

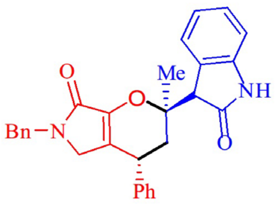


**5aa**

($^1$H NMR, 400MHz, $CDCl_3$)

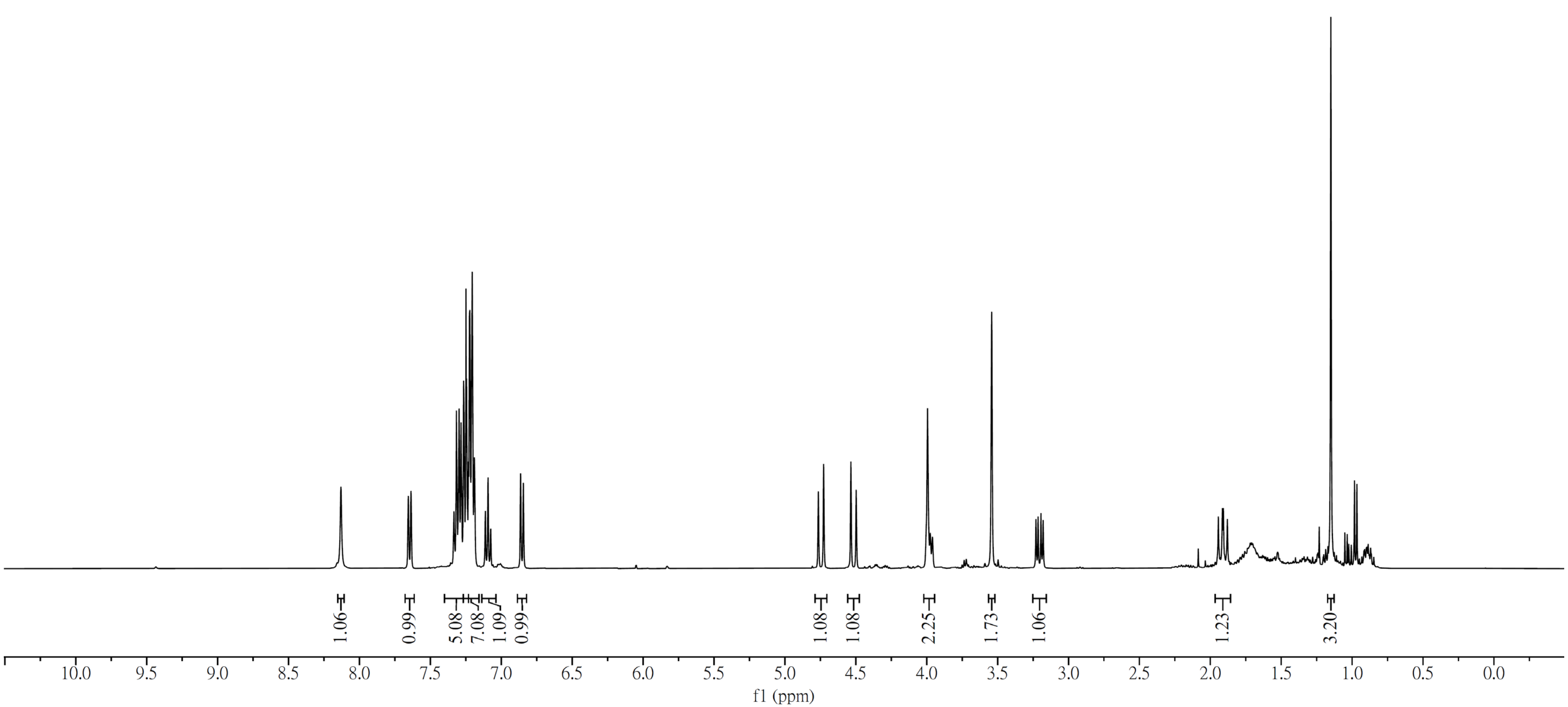

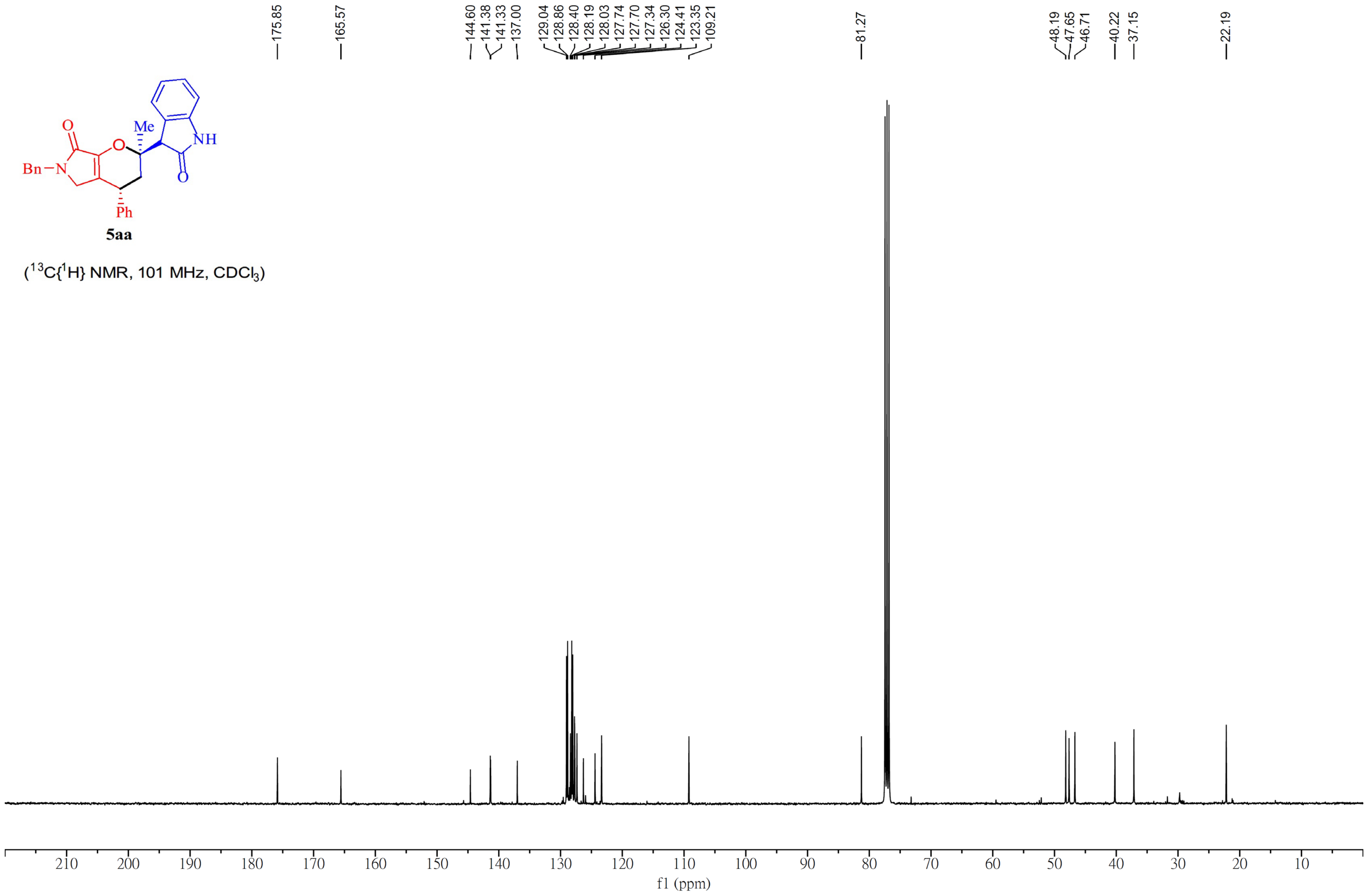


**5aa**

($^{13}C\{^{1}H\}$ NMR, 101 MHz, $CDCl_3$)

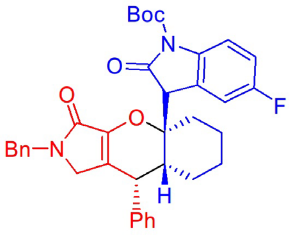

**5ca**

($^1$H NMR, 400MHz, $CDCl_3$)

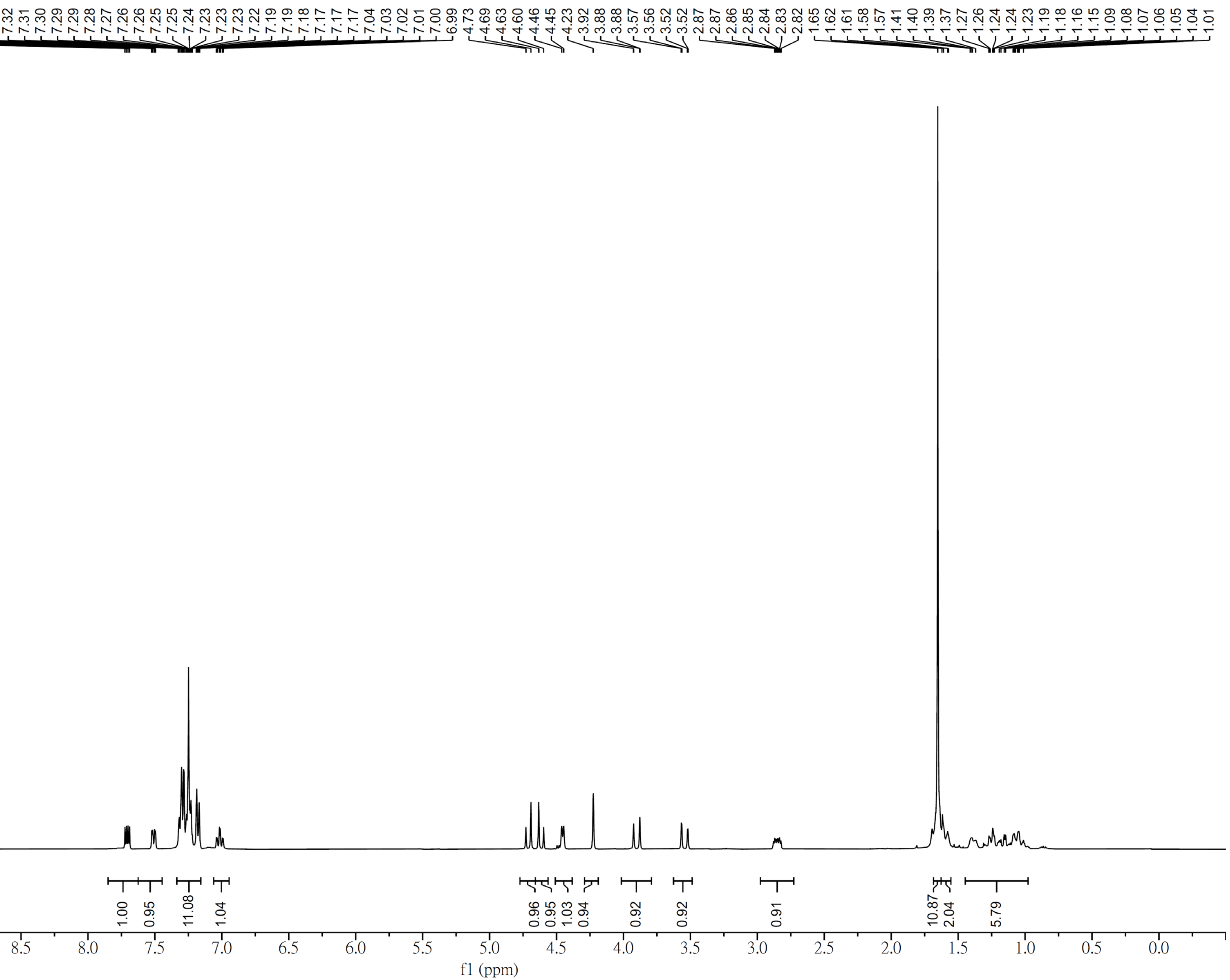

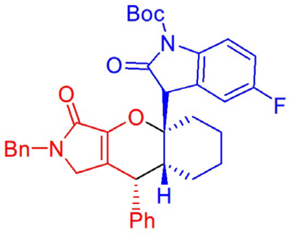

**5ca**

($^{13}C\{^{1}H\}$ NMR, 101 MHz, $CDCl_3$)

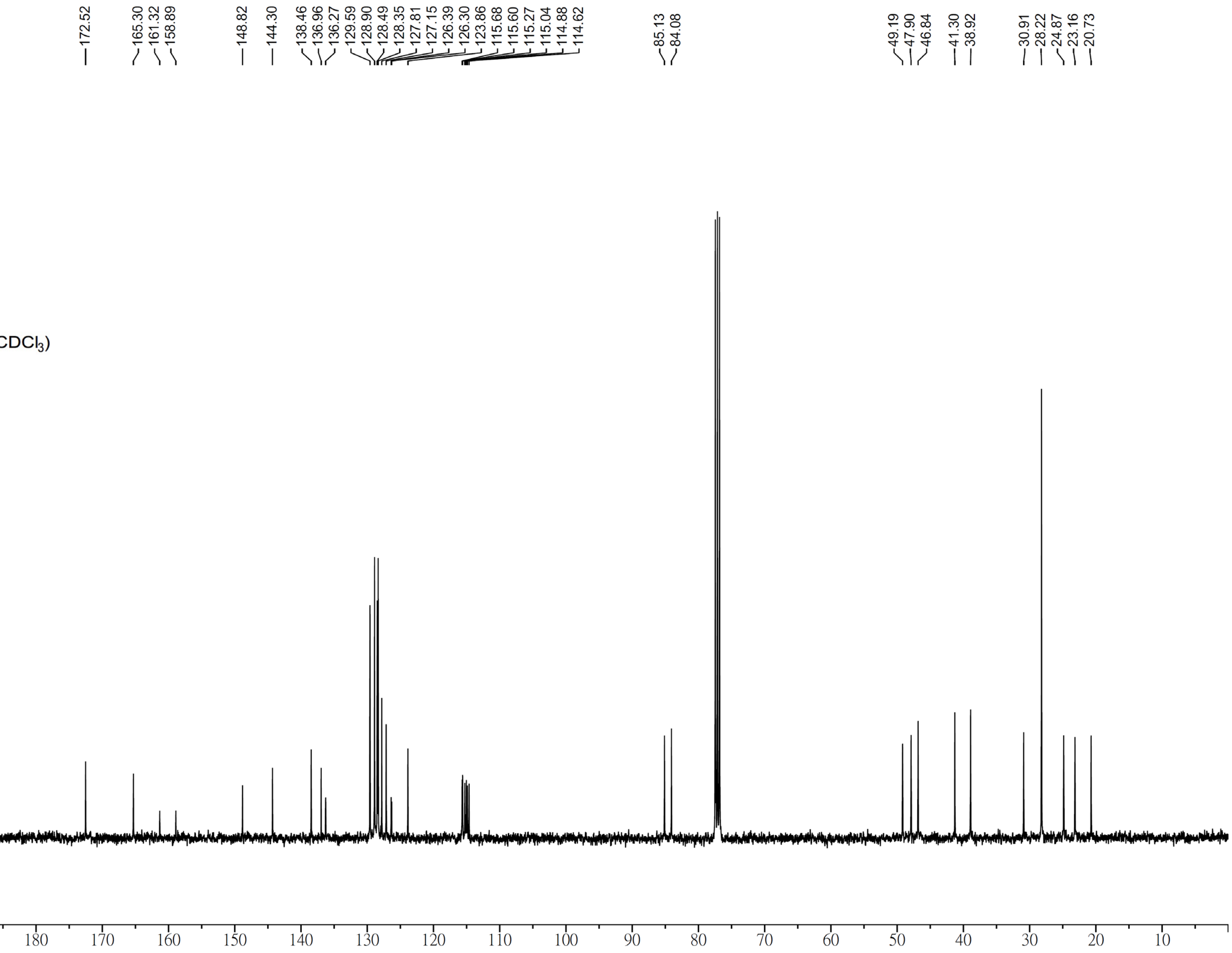

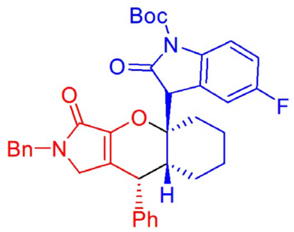


**5ca**

($^{19}$F NMR, 376MHz, $CDCl_3$)

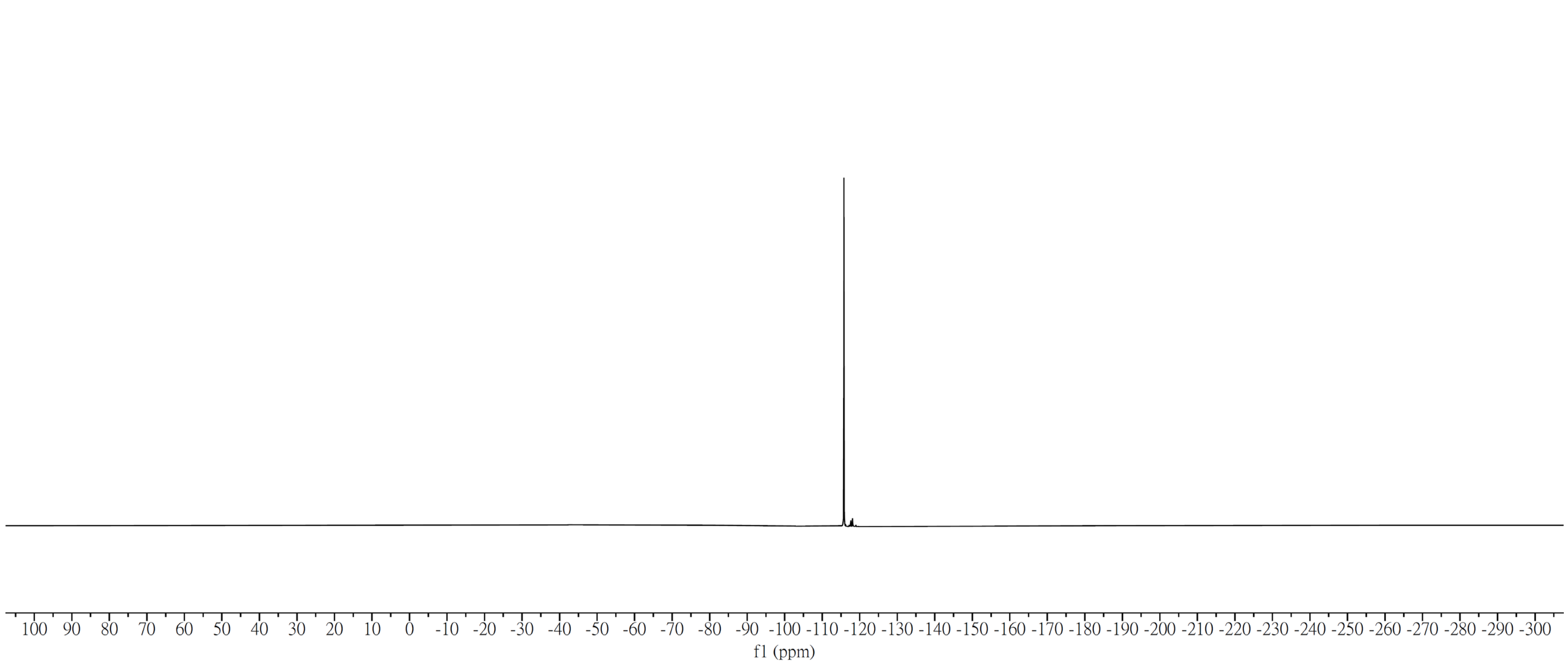

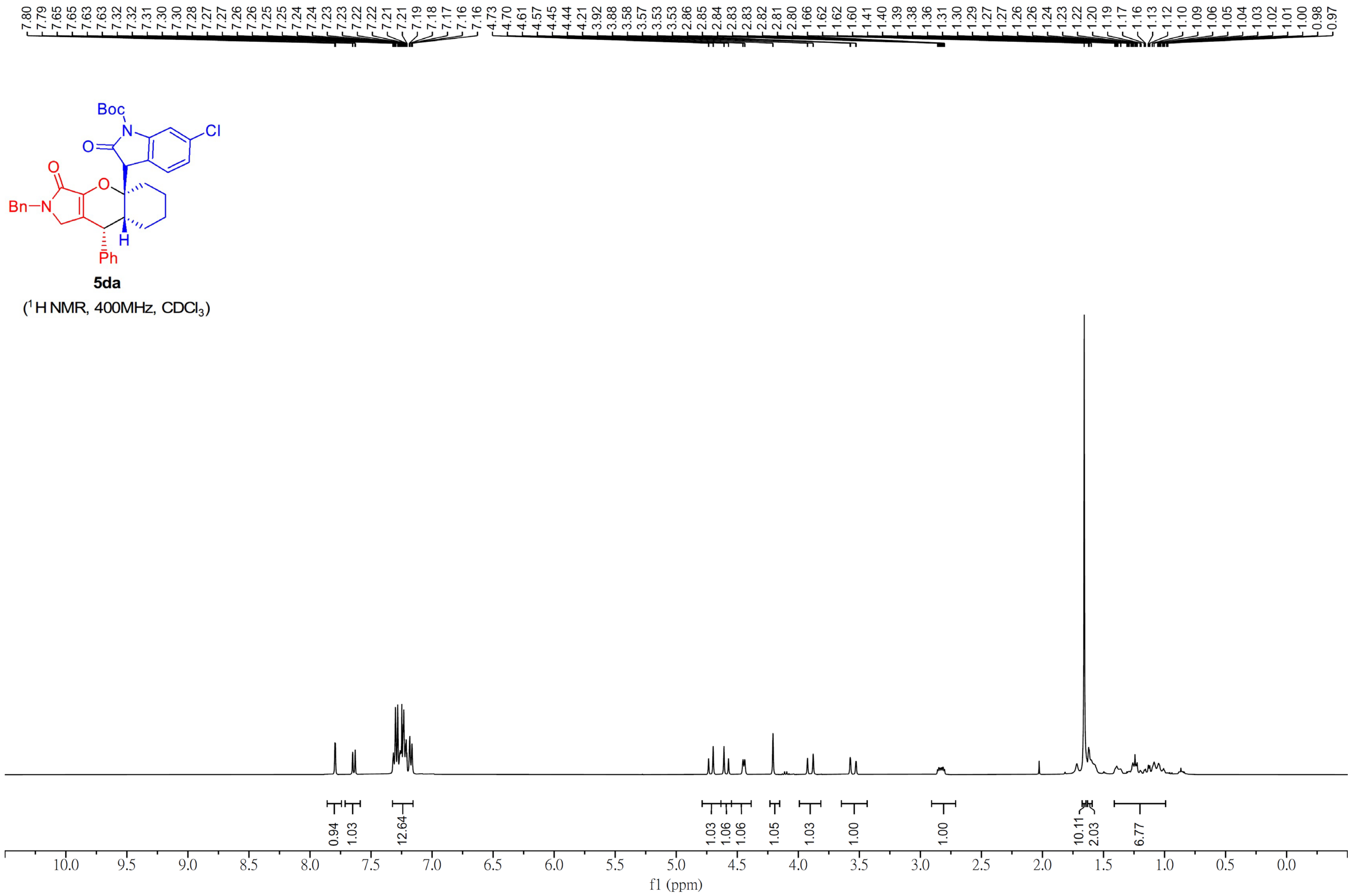
7.80
7.79
7.65
7.65
7.63
7.63
7.32
7.32
7.31
7.30
7.30
7.28
7.27
7.27
7.26
7.26
7.25
7.25
7.24
7.24
7.23
7.23
7.22
7.22
7.21
7.21
7.19
7.18
7.17
7.16
7.16
4.73
4.70
4.61
4.57
4.45
4.44
4.21
3.92
3.88
3.58
3.57
3.53
3.53
2.86
2.85
2.84
2.83
2.83
2.82
2.81
2.80
1.66
1.62
1.62
1.60
1.41
1.40
1.39
1.38
1.36
1.31
1.30
1.29
1.27
1.27
1.26
1.26
1.24
1.23
1.22
1.20
1.19
1.17
1.16
1.13
1.12
1.10
1.09
1.06
1.05
1.04
1.03
1.02
1.01
1.00
0.98
0.97
Boc
N
Cl
O
O
O
Bn
N
H
Ph
5da
(1H NMR, 400MHz, CDCl3)
0.94
1.03
12.64
1.03
1.06
1.06
1.05
1.03
1.00
1.00
10.11
2.03
6.77
10.0
9.5
9.0
8.5
8.0
7.5
7.0
6.5
6.0
5.5
5.0
4.5
4.0
3.5
3.0
2.5
2.0
1.5
1.0
0.5
0.0
f1 (ppm)

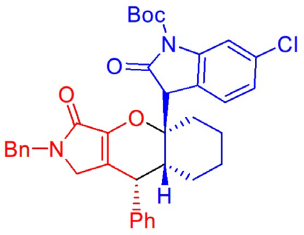


**5da**

($^{13}C\{^1H\}$ NMR, 101 MHz, $CDCl_3$)

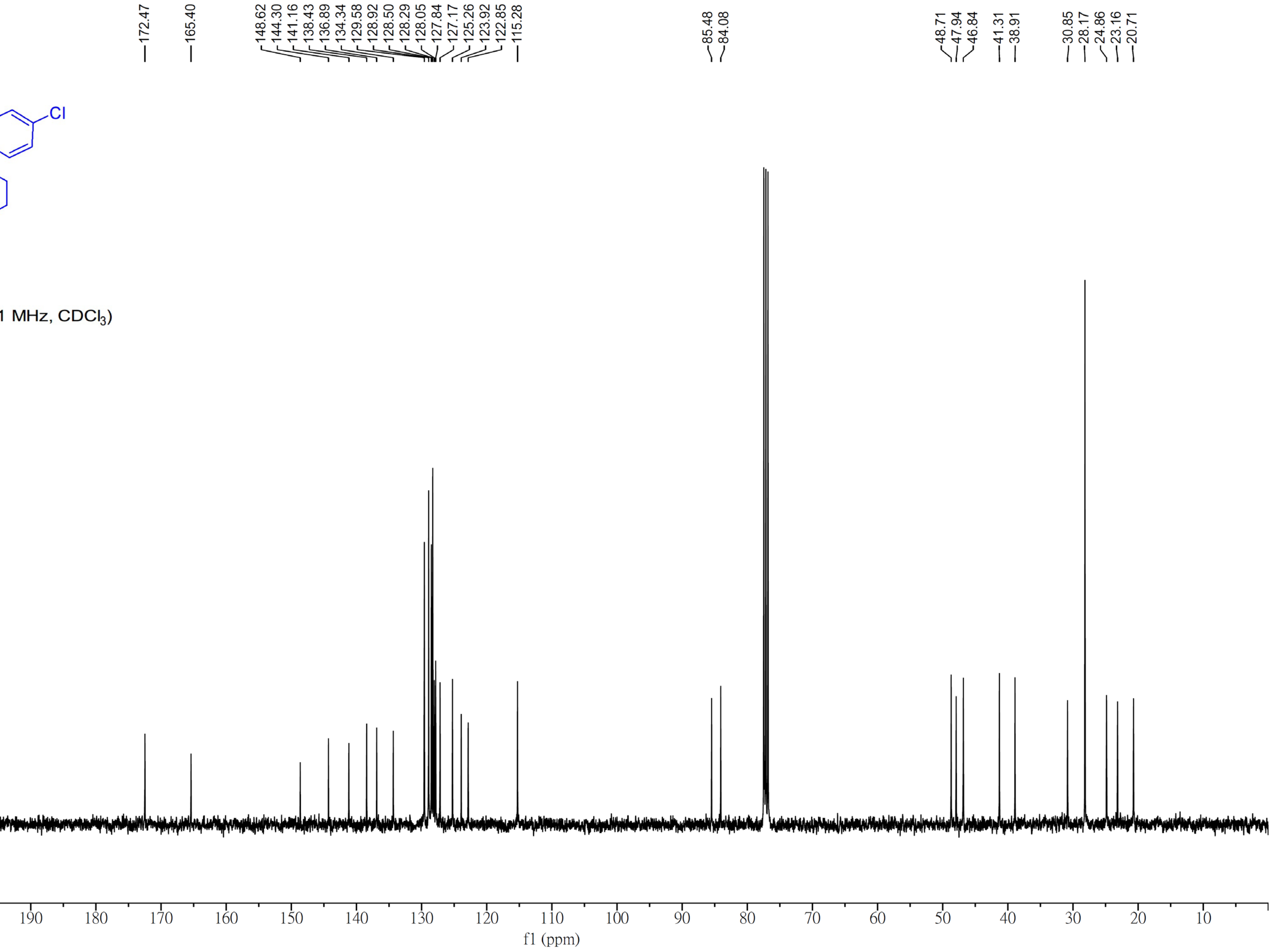

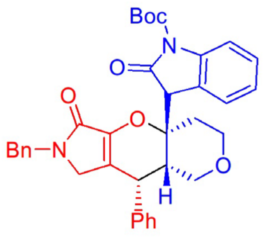


**5ea**

($^1$H NMR, 400MHz, $CDCl_3$)

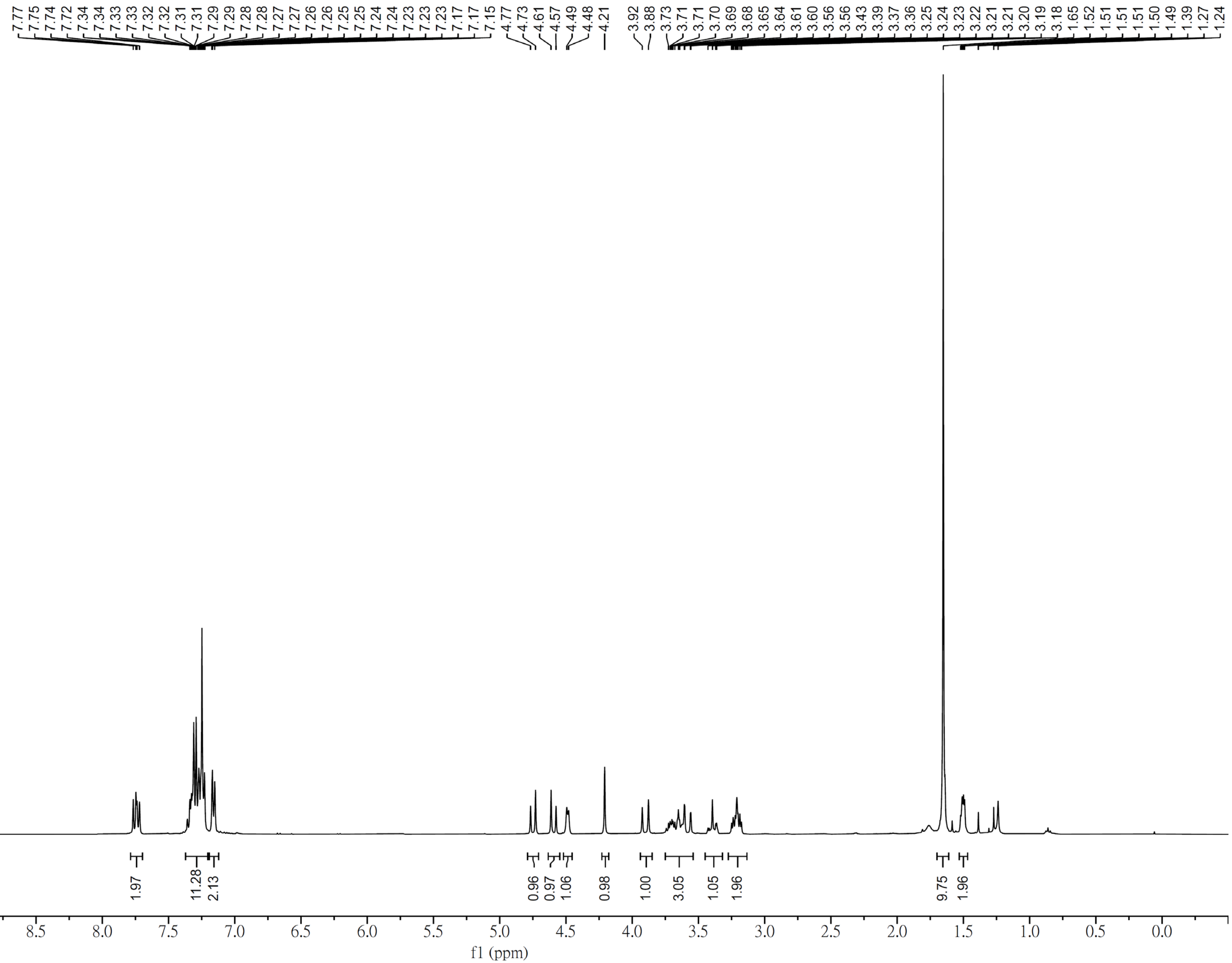

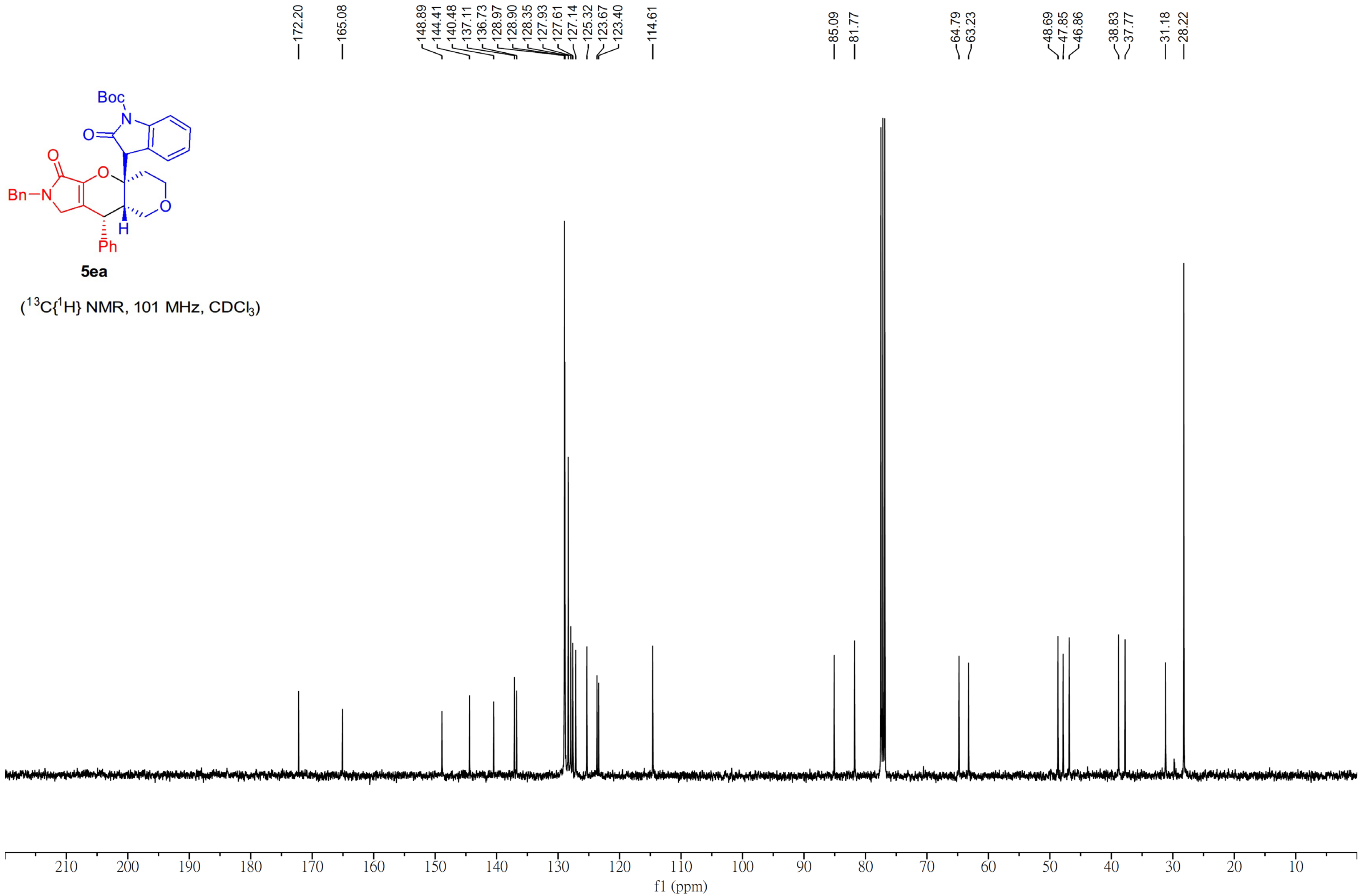


($^{13}C\{^{1}H\}$ NMR, 101 MHz, $CDCl_3$)

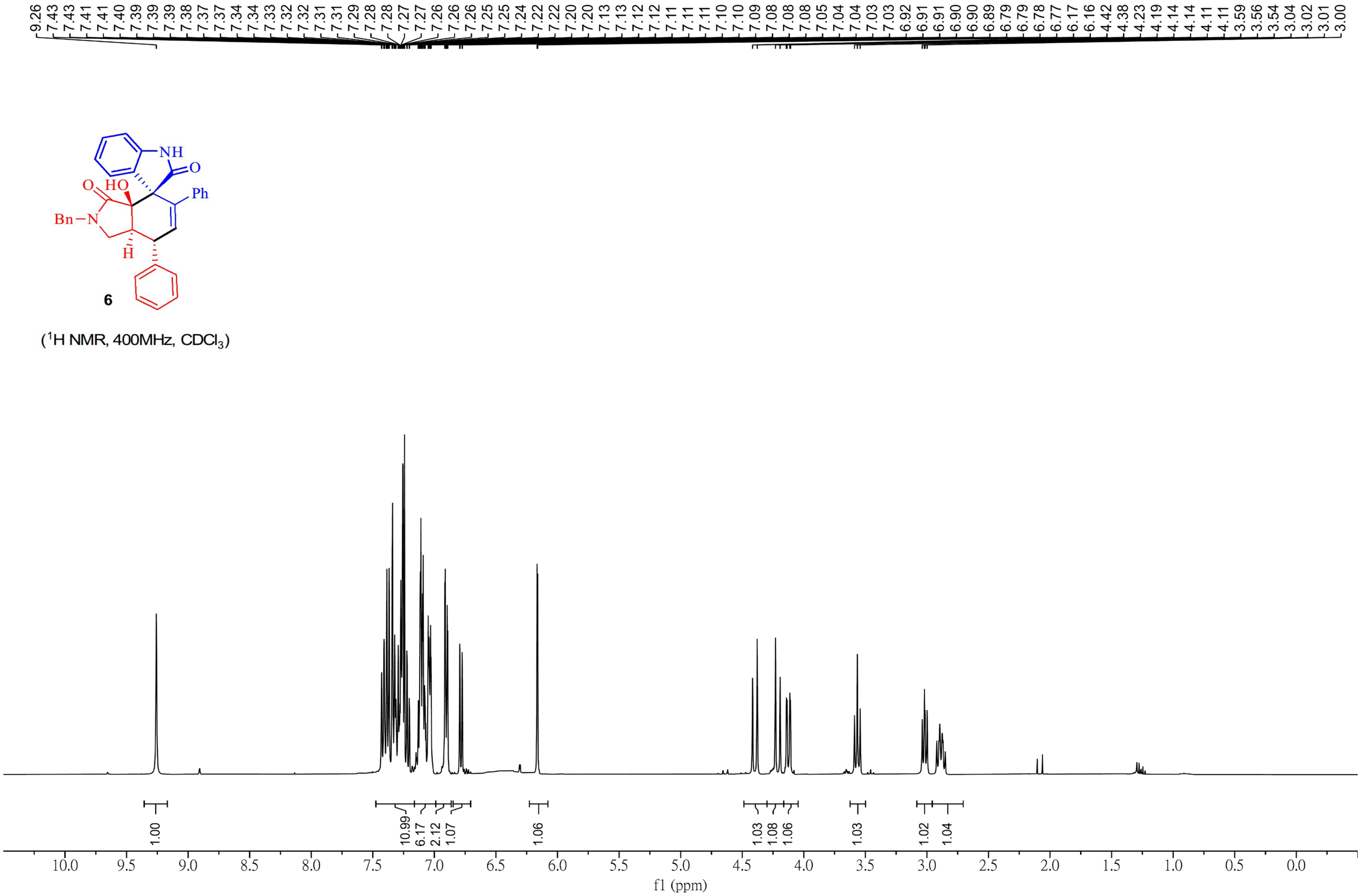


**6**

($^{1}$H NMR, 400MHz, $CDCl_3$)

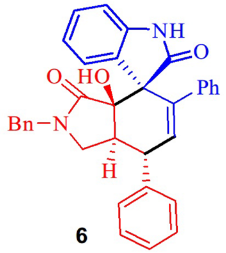


**6**

($^{13}C\{^1H\}$ NMR, 101 MHz, $CDCl_3$)

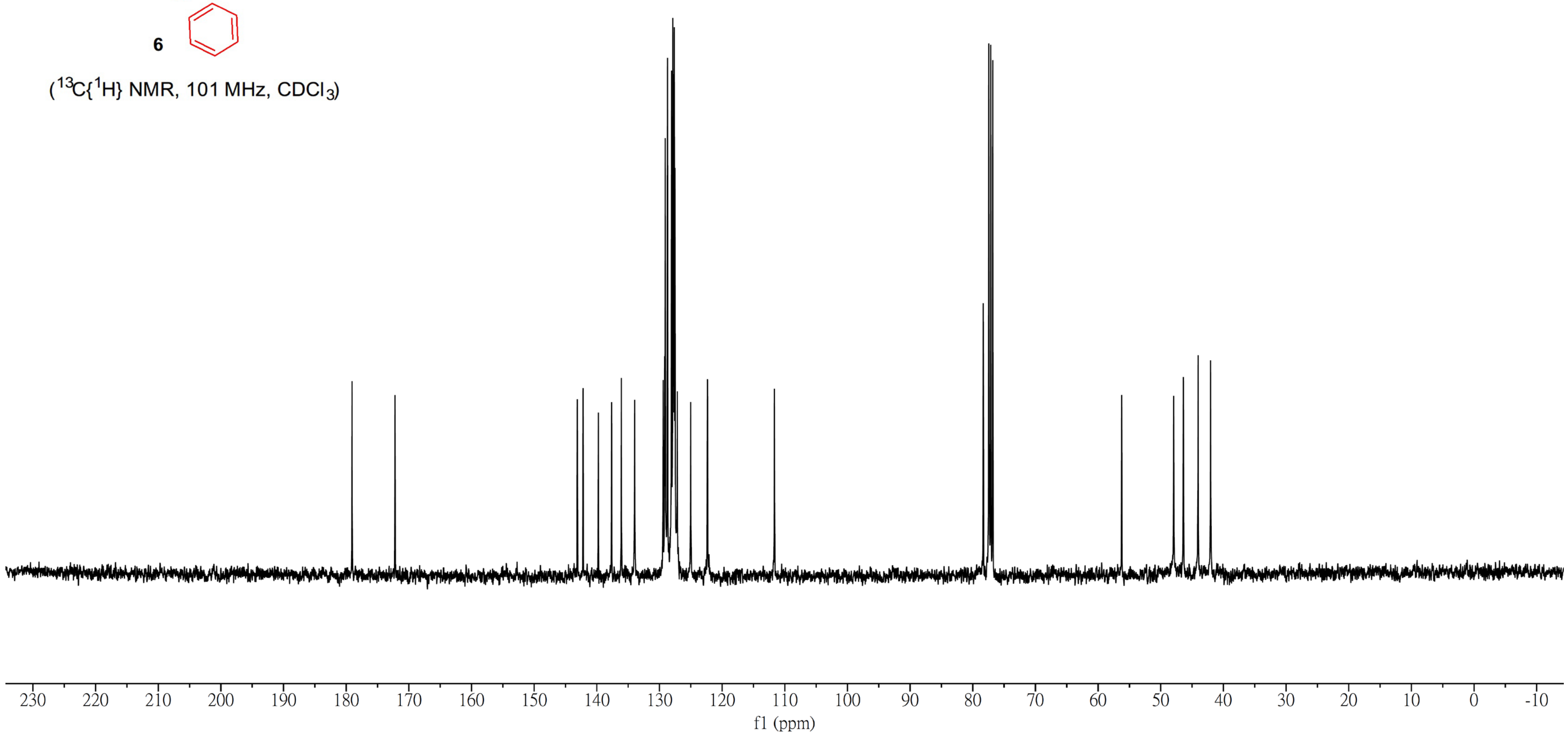

## 8. Copies of HPLC Spectra of Products

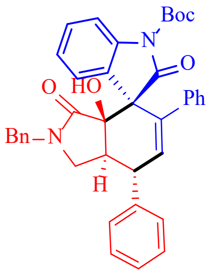

**3aa**

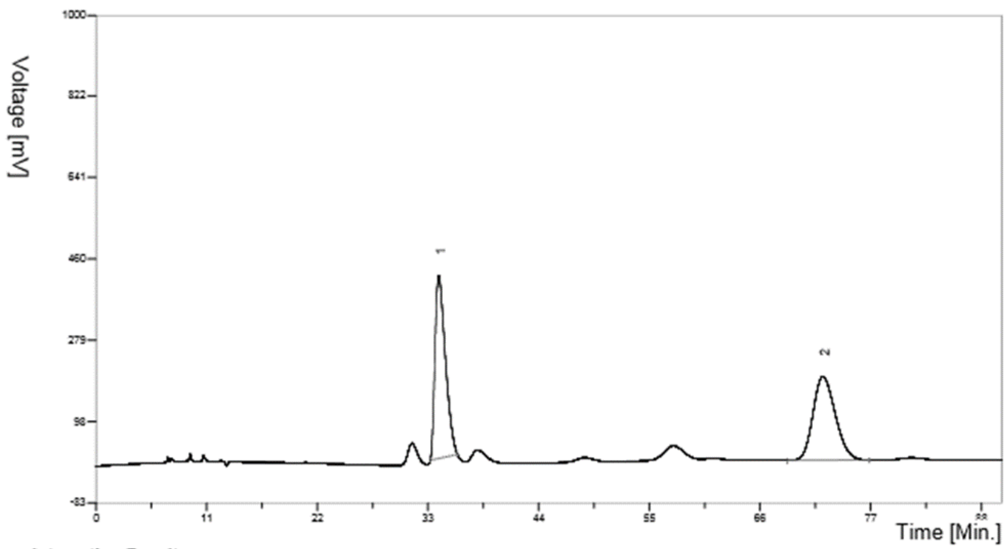

Integration Result

| # | Ret. Time(min) | Area(mv.sec) | Area Percentage(%) |
|---|---|---|---|
| 1 | 34.07 | 29231.52 | 50.4977 |
| 2 | 72.26 | 28655.31 | 49.5023 |
| Total | | 57886.83 | 100 |

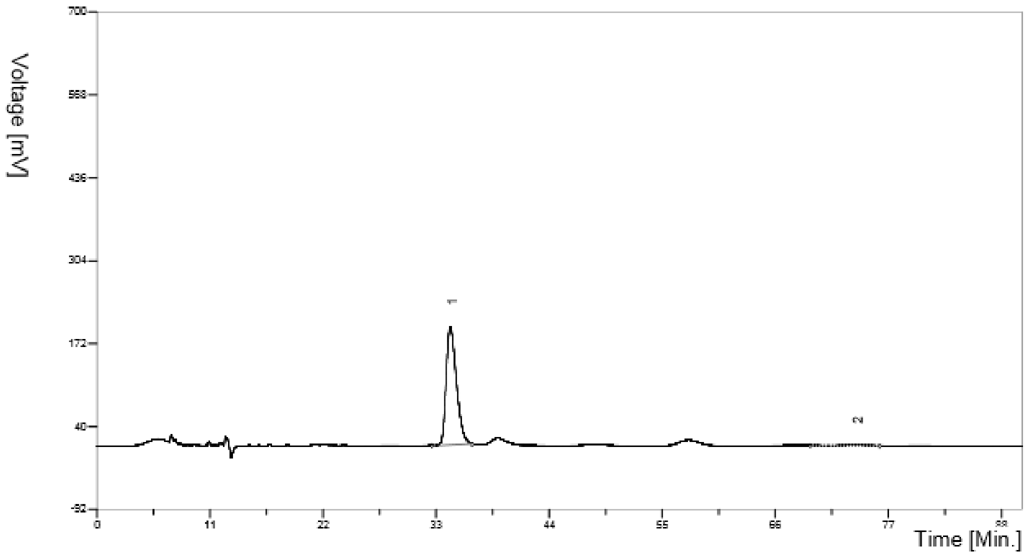

Integration Result

| # | Ret. Time(min) | Area(mv.sec) | Area Percentage(%) |
|---|---|---|---|
| 1 | 34.39 | 13010.95 | 98.6128 |
| 2 | 73.84 | 183.03 | 1.3872 |
| Total | | 13193.99 | 100 |

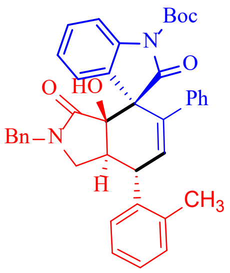


**3ab**

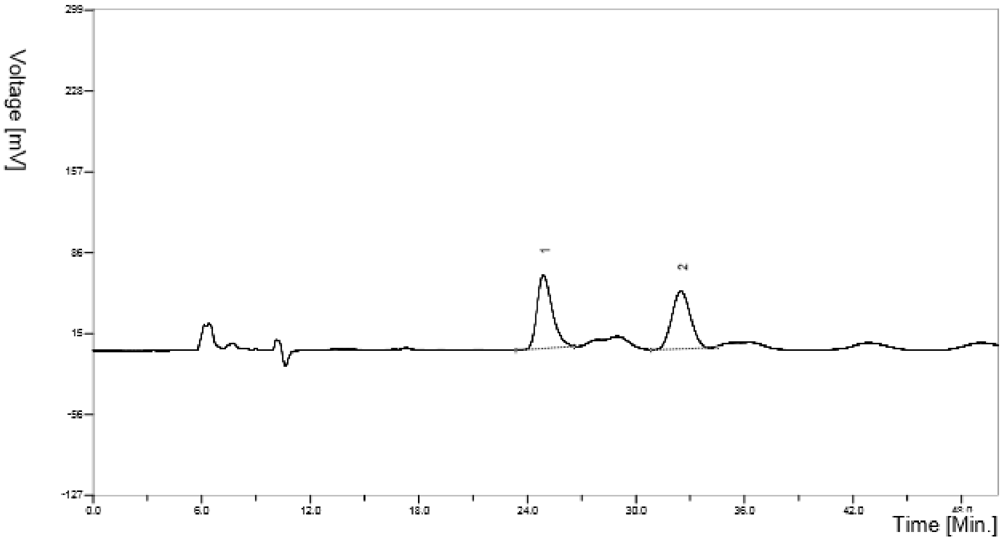


Integration Result

| # | Ret. Time(min) | Area(mv.sec) | Area Percentage(%) |
|---|---|---|---|
| 1 | 24.88 | 3718.48 | 51.3191 |
| 2 | 32.49 | 3527.32 | 48.6809 |
| Total | | 7245.80 | 100 |

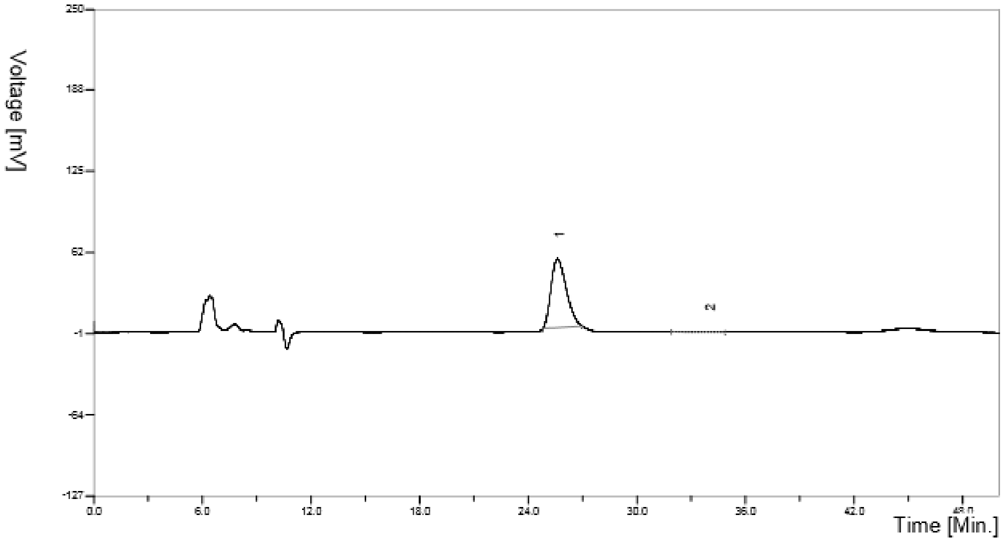


Integration Result

| # | Ret. Time(min) | Area(mv.sec) | Area Percentage(%) |
|---|---|---|---|
| 1 | 25.61 | 3130.09 | 99.7901 |
| 2 | 33.96 | 6.58 | 0.2099 |
| Total | | 3136.68 | 100 |

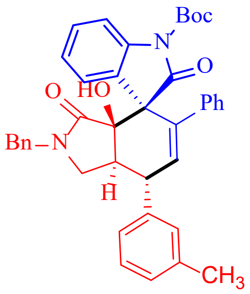


**3ac**

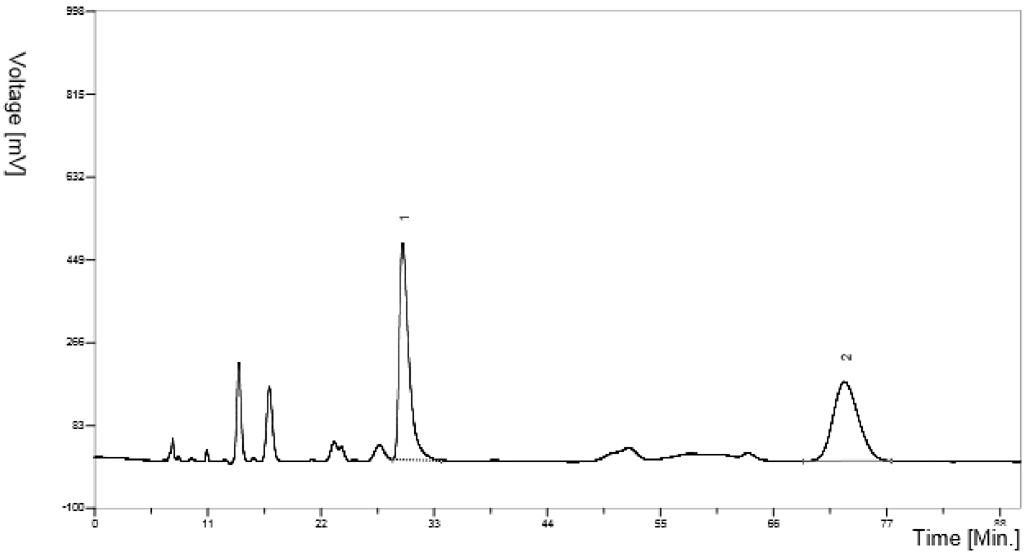


Integration Result

| # | Ret. Time(min) | Area(mv.sec) | Area Percentage(%) |
|---|---|---|---|
| 1 | 29.93 | 31639.38 | 51.3736 |
| 2 | 72.90 | 29947.44 | 48.6264 |
| Total | | 61586.83 | 100 |

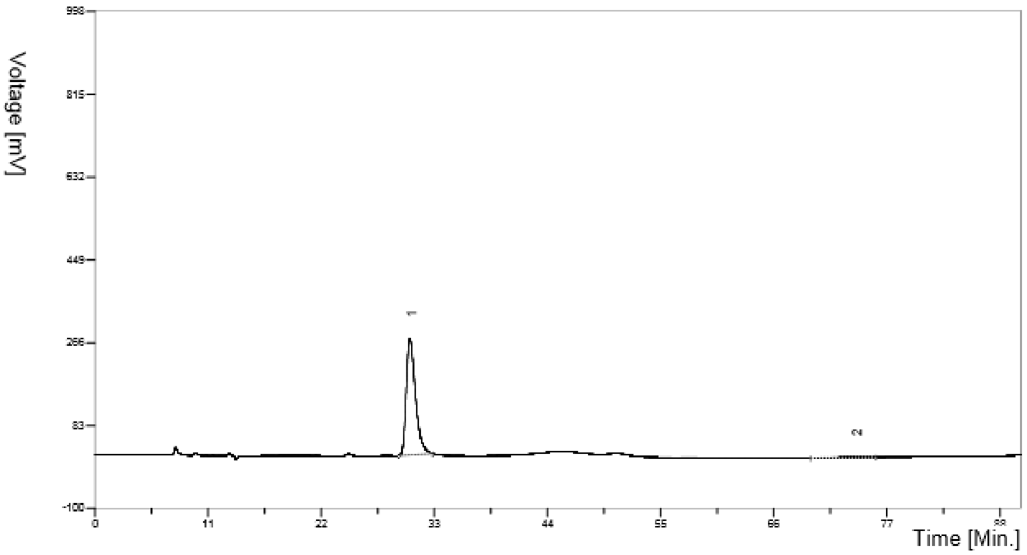


Integration Result

| # | Ret. Time(min) | Area(mv.sec) | Area Percentage(%) |
|---|---|---|---|
| 1 | 30.62 | 16093.20 | 98.6845 |
| 2 | 73.91 | 214.53 | 1.3155 |
| Total | | 16307.73 | 100 |

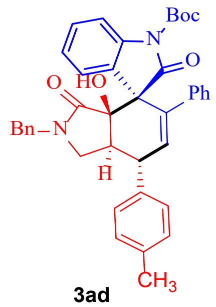


**3ad**

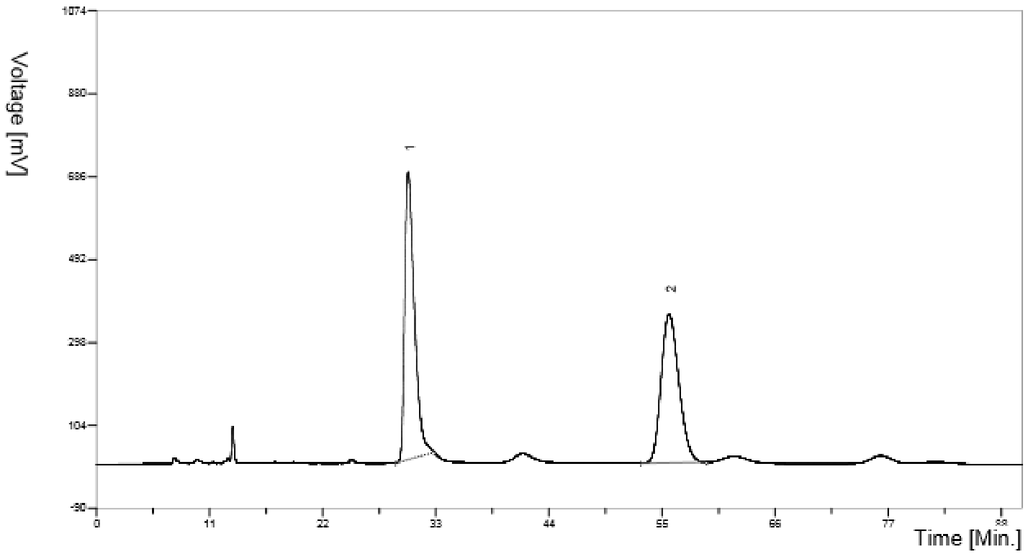


**Integration Result**

| # | Ret. Time(min) | Area(mv.sec) | Area Percentage(%) |
|---|---|---|---|
| 1 | 30.31 | 43960.86 | 51.2135 |
| 2 | 55.68 | 41877.63 | 48.7865 |
| Total | | 85838.49 | 100 |

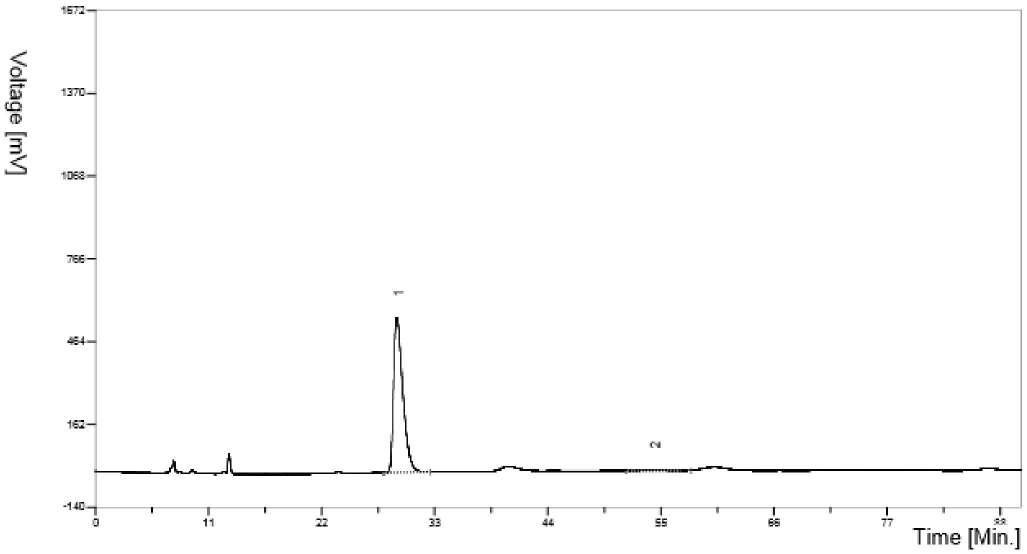


**Integration Result**

| # | Ret. Time(min) | Area(mv.sec) | Area Percentage(%) |
|---|---|---|---|
| 1 | 29.31 | 35532.83 | 99.4794 |
| 2 | 54.25 | 185.95 | 0.5206 |
| Total | | 35718.79 | 100 |

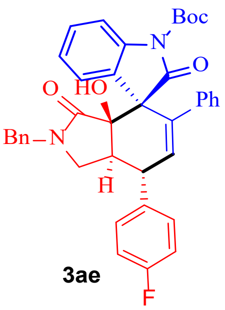


**3ae**

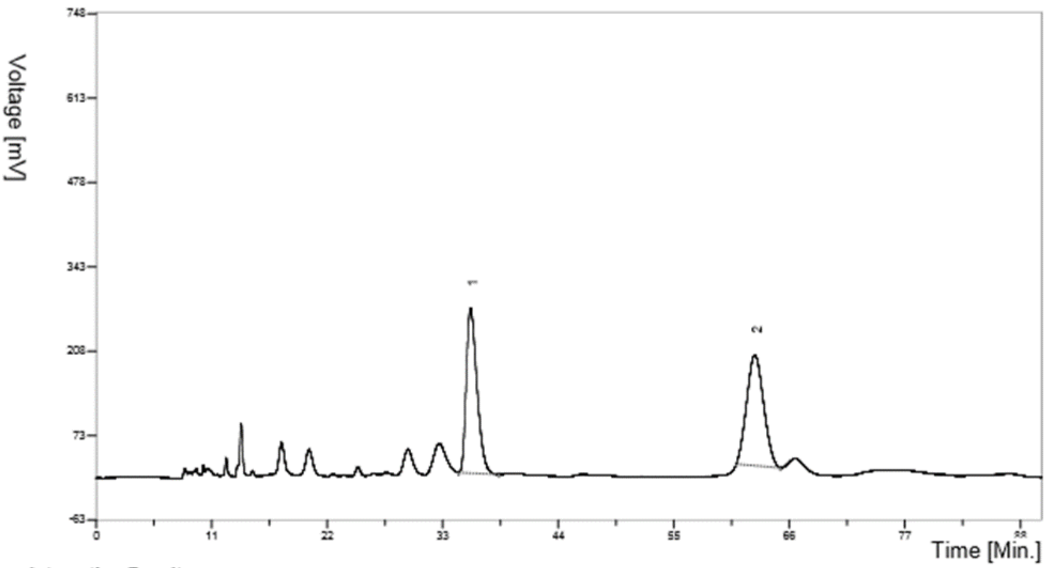


Integration Result

| # | Ret. Time(min) | Area(mv.sec) | Area Percentage(%) |
|---|---|---|---|
| 1 | 35.65 | 18724.21 | 48.5190 |
| 2 | 62.72 | 19867.30 | 51.4810 |
| Total | | 38591.50 | 100 |

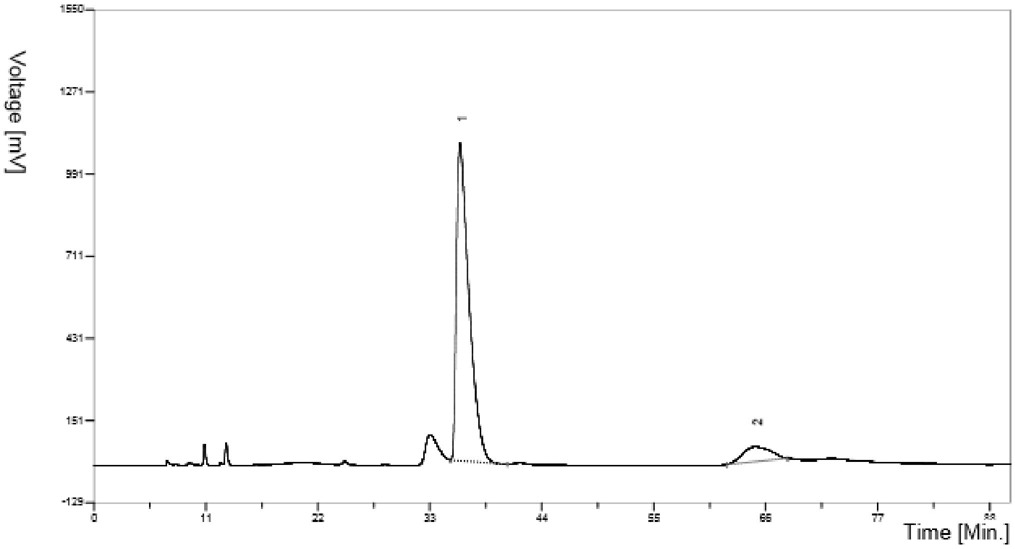


Integration Result

| # | Ret. Time(min) | Area(mv.sec) | Area Percentage(%) |
|---|---|---|---|
| 1 | 35.96 | 92827.59 | 90.6878 |
| 2 | 65.02 | 9531.94 | 9.3122 |
| Total | | 102359.54 | 100 |

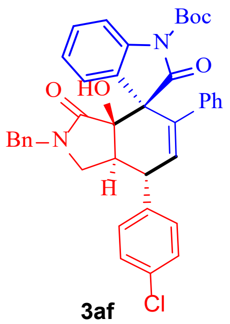


**3af**

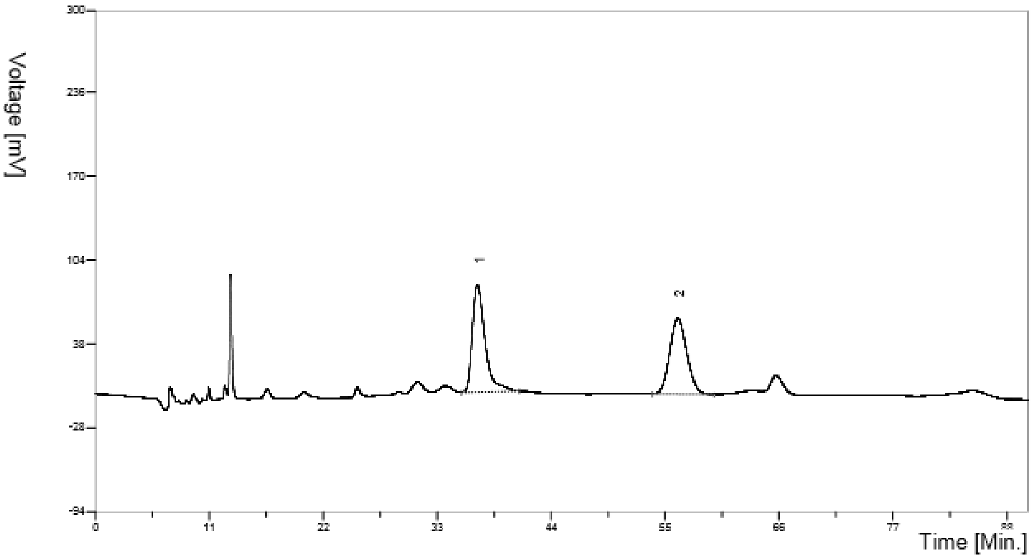


**Integration Result**

| # | Ret. Time(min) | Area(mv.sec) | Area Percentage(%) |
|---|---|---|---|
| 1 | 36.89 | 7158.56 | 51.1141 |
| 2 | 56.23 | 6846.49 | 48.8859 |
| Total | | 14005.05 | 100 |

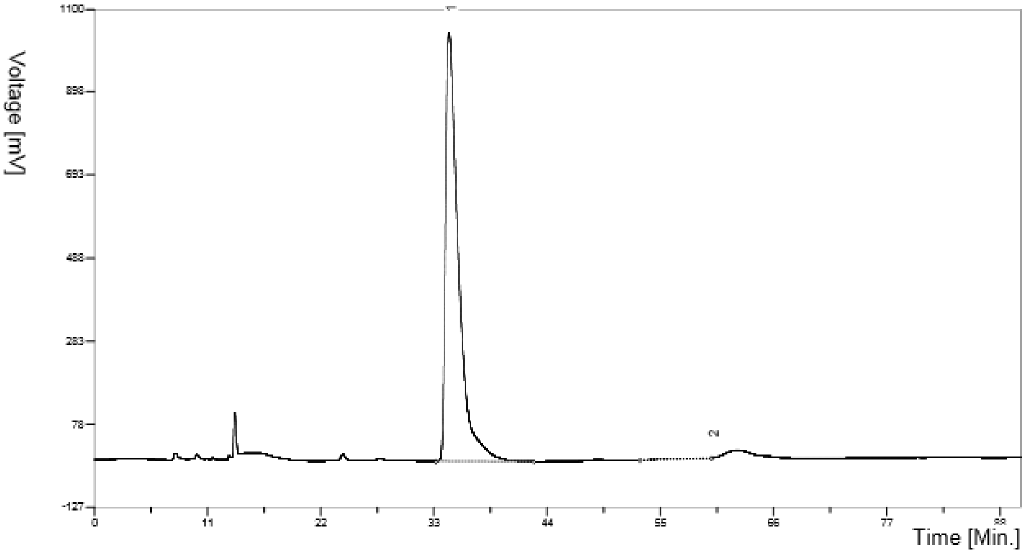


**Integration Result**

| # | Ret. Time(min) | Area(mv.sec) | Area Percentage(%) |
|---|---|---|---|
| 1 | 34.47 | 92486.85 | 99.8978 |
| 2 | 60.00 | 94.58 | 0.1022 |
| Total | | 92581.42 | 100 |

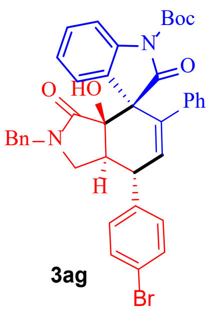


**3ag**

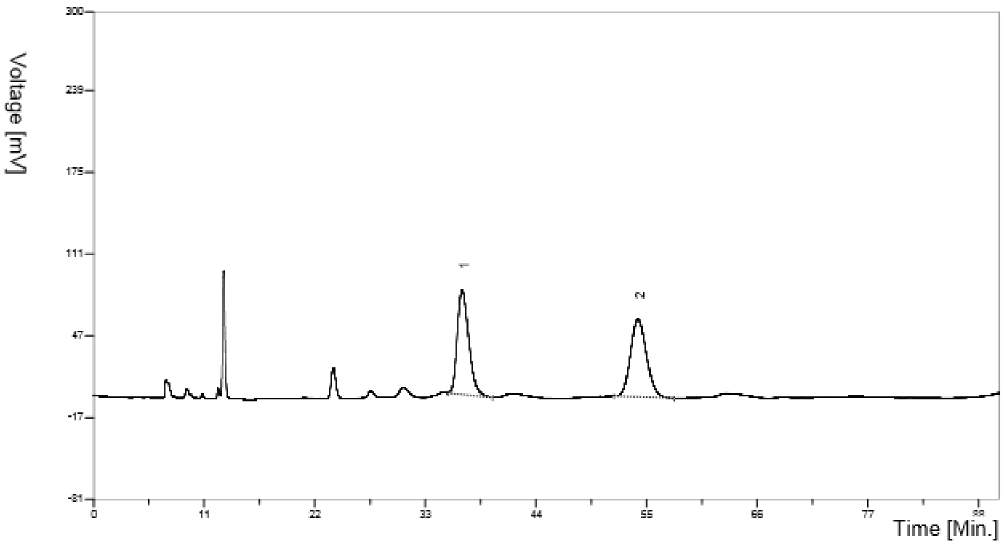


**Integration Result**

| # | Ret. Time(min) | Area(mv.sec) | Area Percentage(%) |
|---|---|---|---|
| 1 | 36.65 | 6498.89 | 49.1368 |
| 2 | 54.14 | 6727.24 | 50.8632 |
| Total | | 13226.13 | 100 |

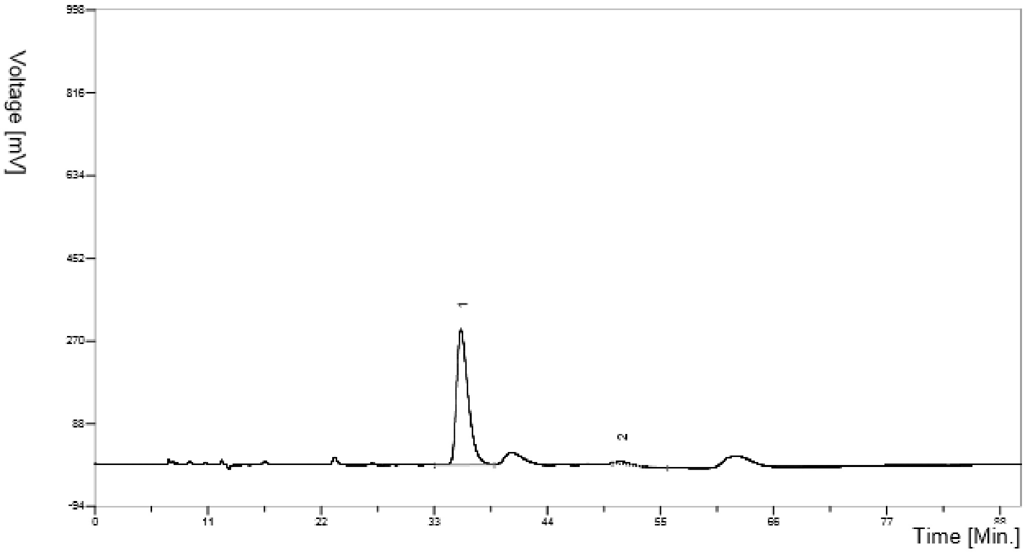


**Integration Result**

| # | Ret. Time(min) | Area(mv.sec) | Area Percentage(%) |
|---|---|---|---|
| 1 | 35.59 | 23194.18 | 99.4172 |
| 2 | 51.13 | 135.97 | 0.5828 |
| Total | | 23330.15 | 100 |

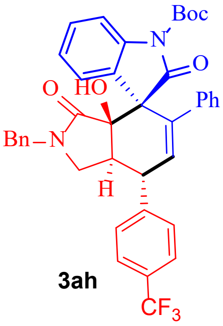


**3ah**

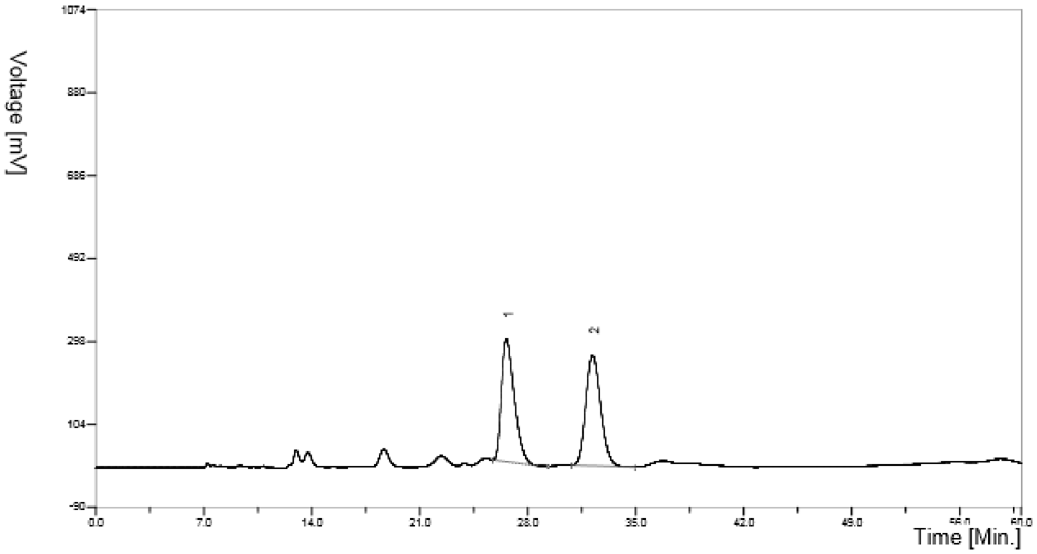

**Integration Result**

| # | Ret. Time(min) | Area(mv.sec) | Area Percentage(%) |
|---|---|---|---|
| 1 | 26.62 | 16487.03 | 48.6885 |
| 2 | 32.20 | 17375.27 | 51.3115 |
| Total | | 33862.30 | 100 |

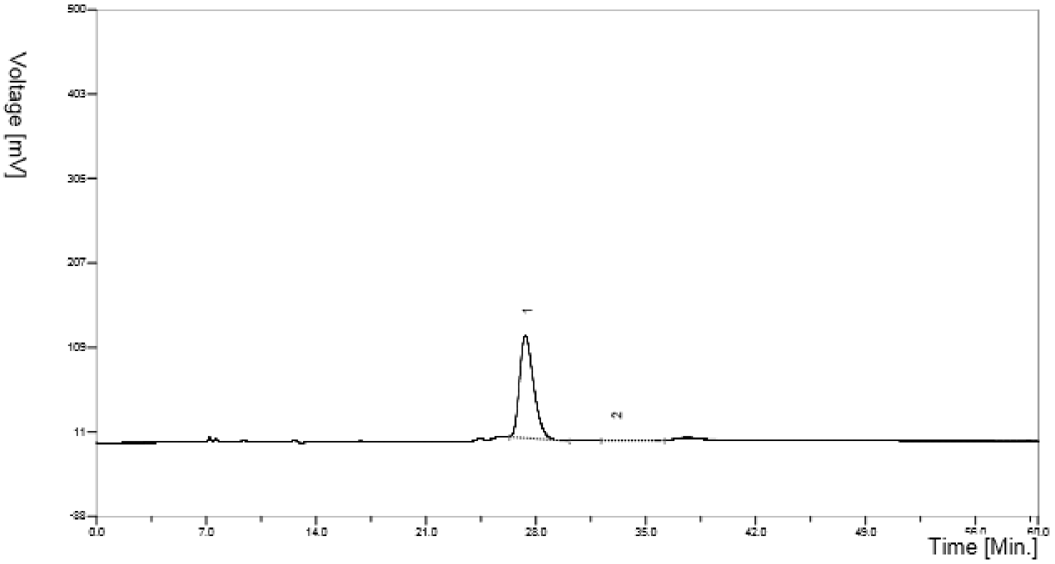

**Integration Result**

| # | Ret. Time(min) | Area(mv.sec) | Area Percentage(%) |
|---|---|---|---|
| 1 | 27.33 | 7340.24 | 99.7345 |
| 2 | 33.04 | 19.54 | 0.2655 |
| Total | | 7359.78 | 100 |

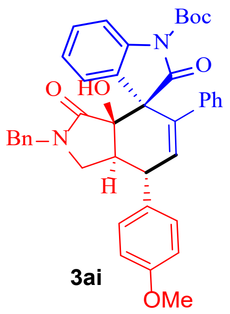


**3ai**

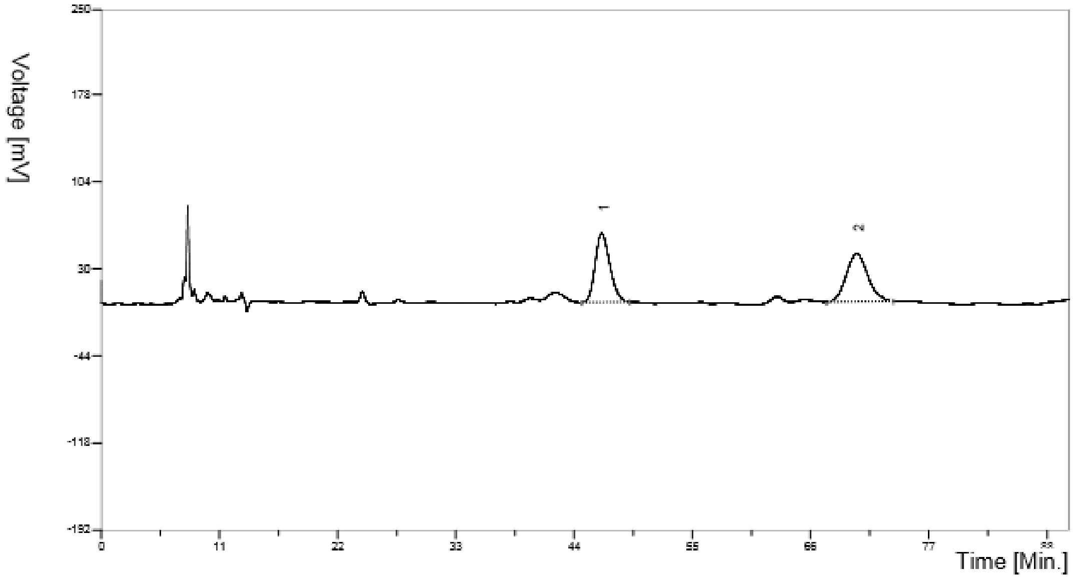


**Integration Result**

| # | Ret. Time(min) | Area(mv.sec) | Area Percentage(%) |
|---|---|---|---|
| 1 | 46.56 | 5538.10 | 49.2885 |
| 2 | 70.30 | 5697.99 | 50.7115 |
| Total | | 11236.09 | 100 |

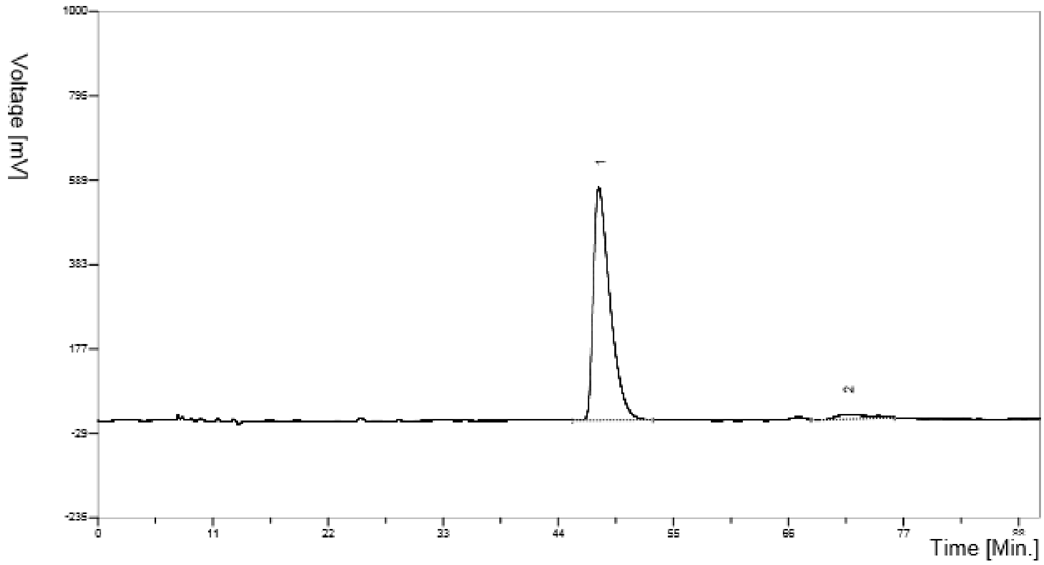


**Integration Result**

| # | Ret. Time(min) | Area(mv.sec) | Area Percentage(%) |
|---|---|---|---|
| 1 | 47.87 | 62808.35 | 96.7724 |
| 2 | 71.60 | 2094.81 | 3.2276 |
| Total | | 64903.17 | 100 |

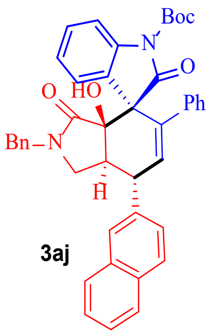


**3aj**

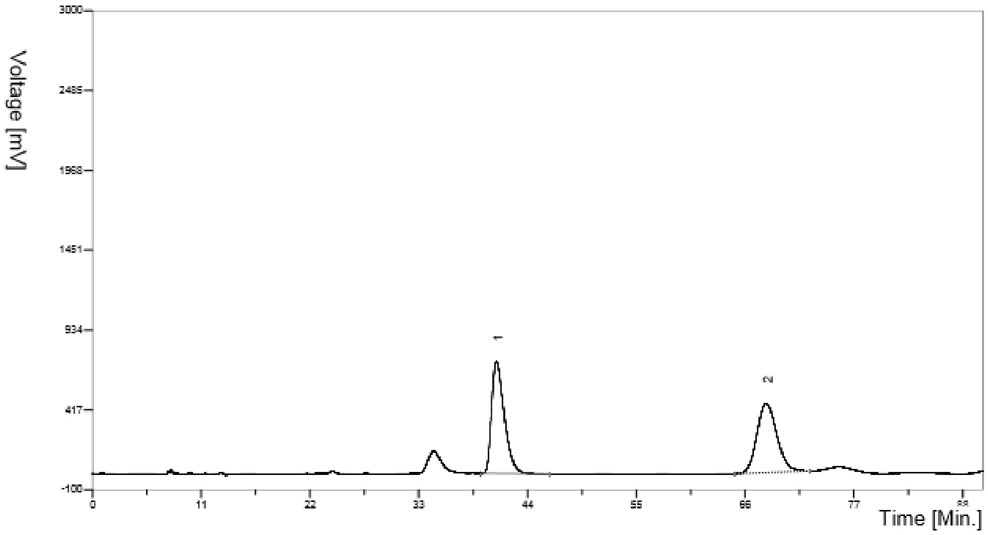


**Integration Result**

| # | Ret. Time(min) | Area(mv.sec) | Area Percentage(%) |
|---|---|---|---|
| 1 | 40.85 | 64890.43 | 50.7676 |
| 2 | 68.14 | 62928.27 | 49.2324 |
| Total | | 127818.69 | 100 |

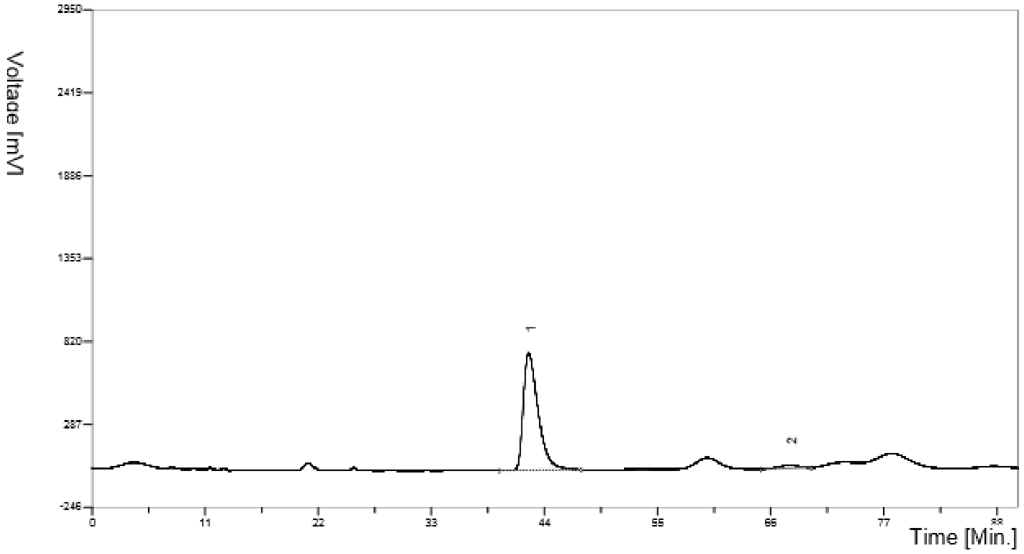


**Integration Result**

| # | Ret. Time(min) | Area(mv.sec) | Area Percentage(%) |
|---|---|---|---|
| 1 | 42.47 | 74905.18 | 96.7827 |
| 2 | 67.87 | 2490.04 | 3.2173 |
| Total | | 77395.22 | 100 |

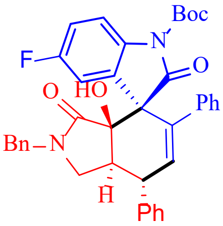


**3ba**

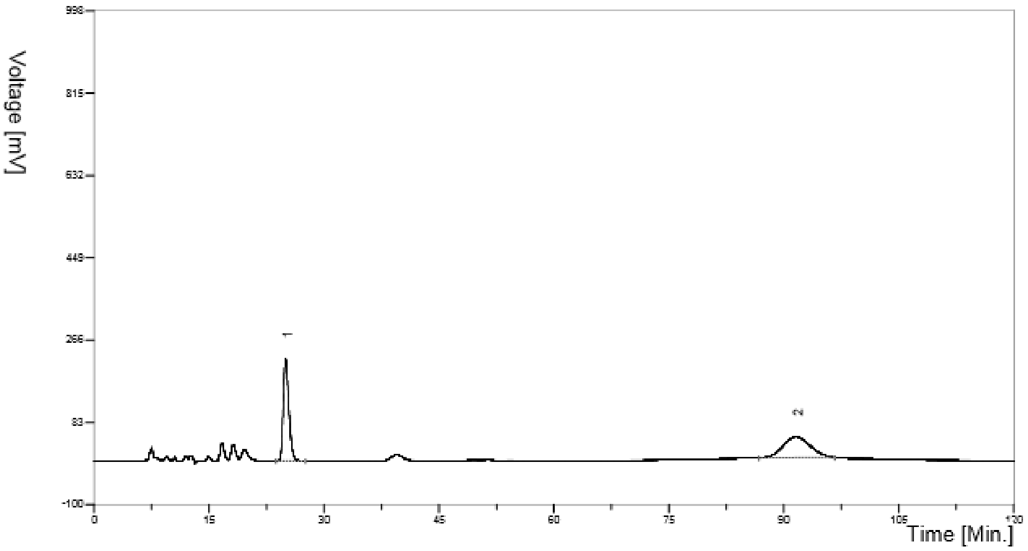


**Integration Result**

| # | Ret. Time(min) | Area(mv.sec) | Area Percentage(%) |
|---|---|---|---|
| 1 | 25.00 | 11525.59 | 50.2390 |
| 2 | 91.60 | 11415.95 | 49.7610 |
| Total | | 22941.53 | 100 |

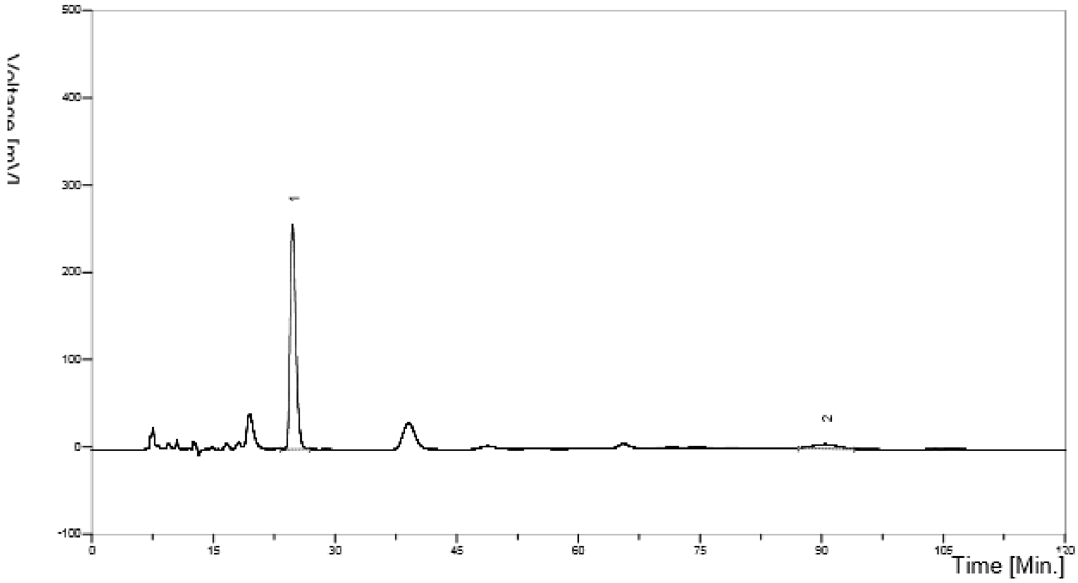


**Integration Result**

| # | Ret. Time(min) | Area(mv.sec) | Area Percentage(%) |
|---|---|---|---|
| 1 | 24.75 | 12615.33 | 92.1197 |
| 2 | 90.43 | 1079.16 | 7.8803 |
| Total | | 13694.49 | 100 |

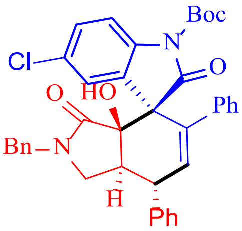

**3ca**

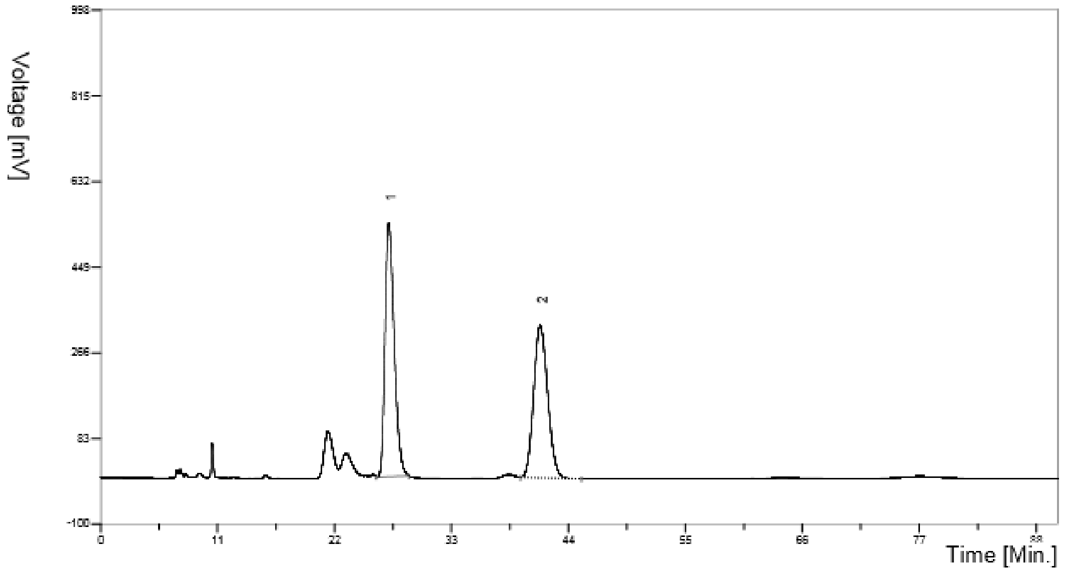

**Integration Result**

| # | Ret. Time(min) | Area(mv.sec) | Area Percentage(%) |
|---|---|---|---|
| 1 | 27.11 | 32347.71 | 51.9810 |
| 2 | 41.35 | 29882.12 | 48.0190 |
| Total | | 62229.83 | 100 |

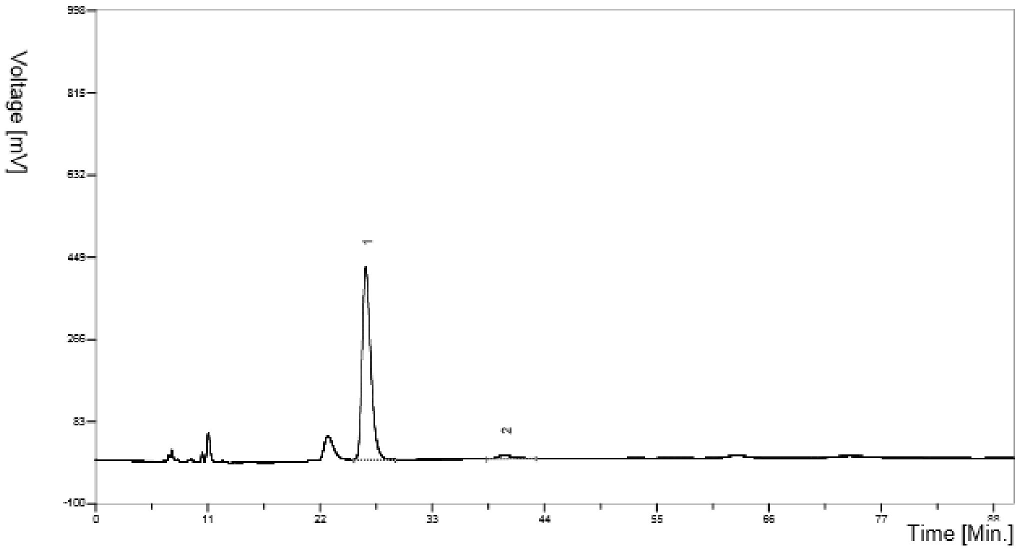

**Integration Result**

| # | Ret. Time(min) | Area(mv.sec) | Area Percentage(%) |
|---|---|---|---|
| 1 | 26.47 | 25492.97 | 97.0451 |
| 2 | 40.08 | 776.23 | 2.9549 |
| Total | | 26269.20 | 100 |

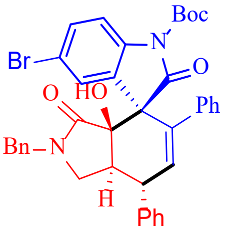


**3da**

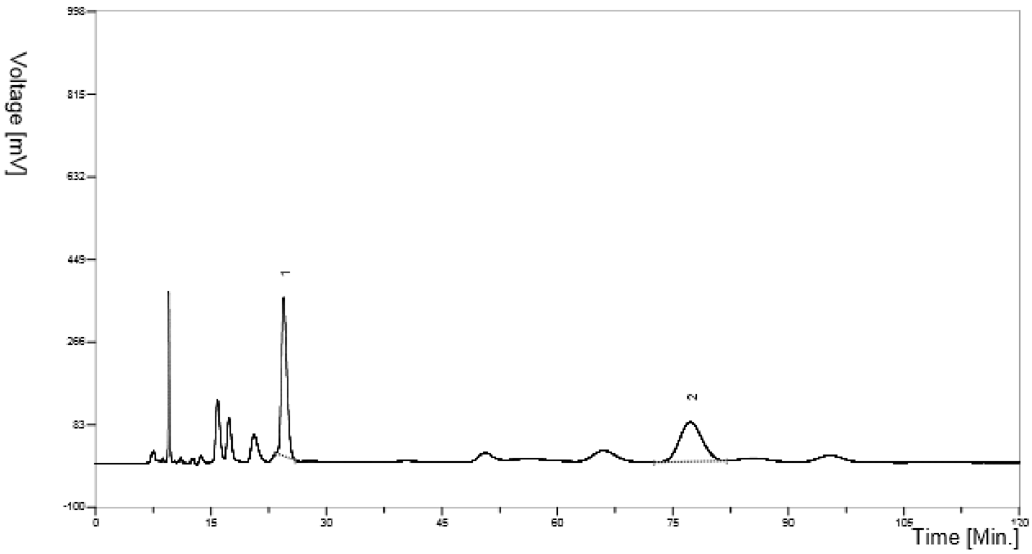


**Integration Result**

| # | Ret. Time(min) | Area(mv.sec) | Area Percentage(%) |
|---|---|---|---|
| 1 | 24.43 | 17429.29 | 49.2496 |
| 2 | 77.27 | 17960.42 | 50.7504 |
| Total | | 35389.71 | 100 |

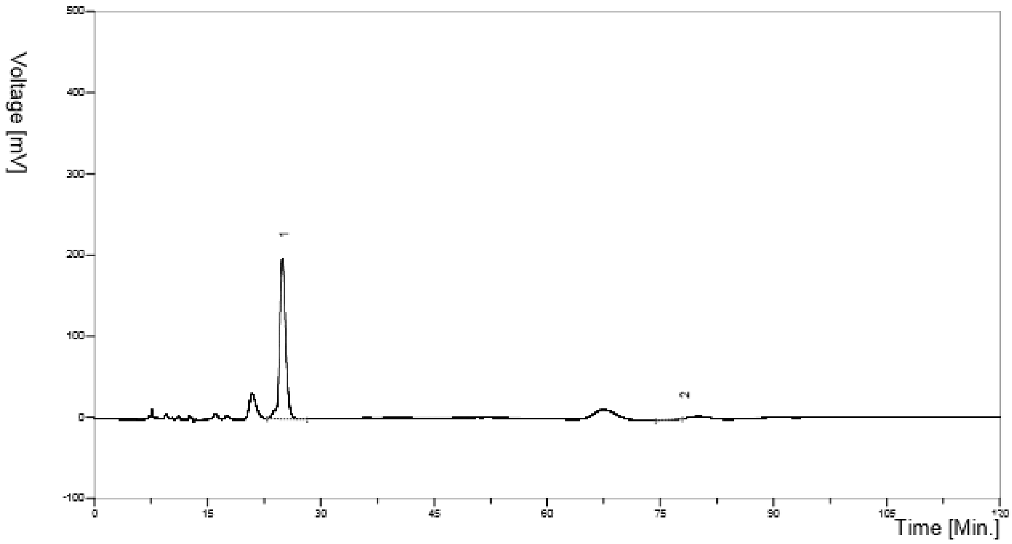


**Integration Result**

| # | Ret. Time(min) | Area(mv.sec) | Area Percentage(%) |
|---|---|---|---|
| 1 | 24.91 | 10854.72 | 98.9502 |
| 2 | 77.94 | 115.16 | 1.0498 |
| Total | | 10969.88 | 100 |

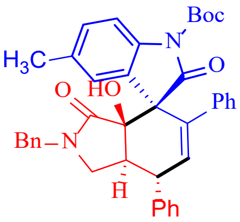


**3ea**

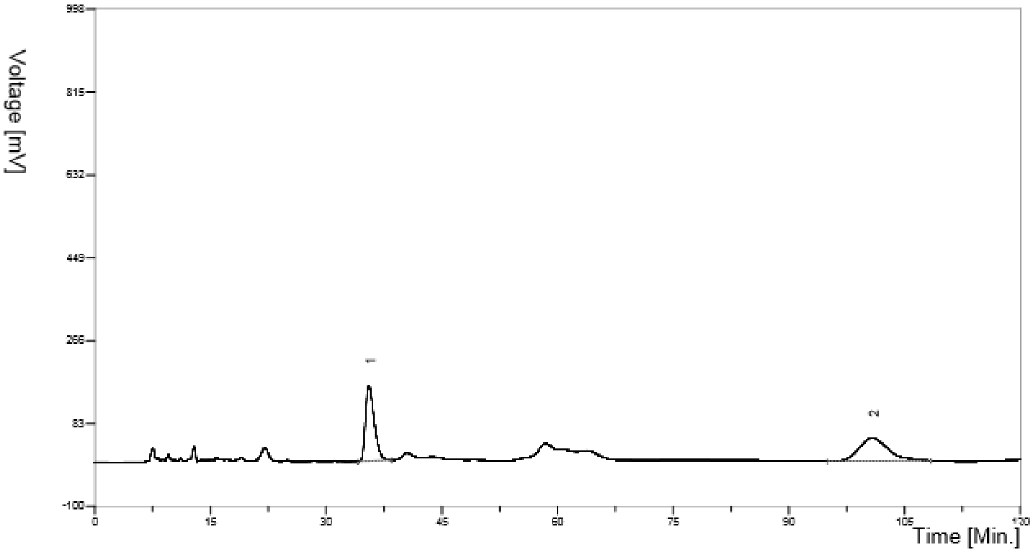


Integration Result

| # | Ret. Time(min) | Area(mv.sec) | Area Percentage(%) |
|---|---|---|---|
| 1 | 35.53 | 13026.07 | 49.4967 |
| 2 | 100.80 | 13290.96 | 50.5033 |
| Total | | 26317.03 | 100 |

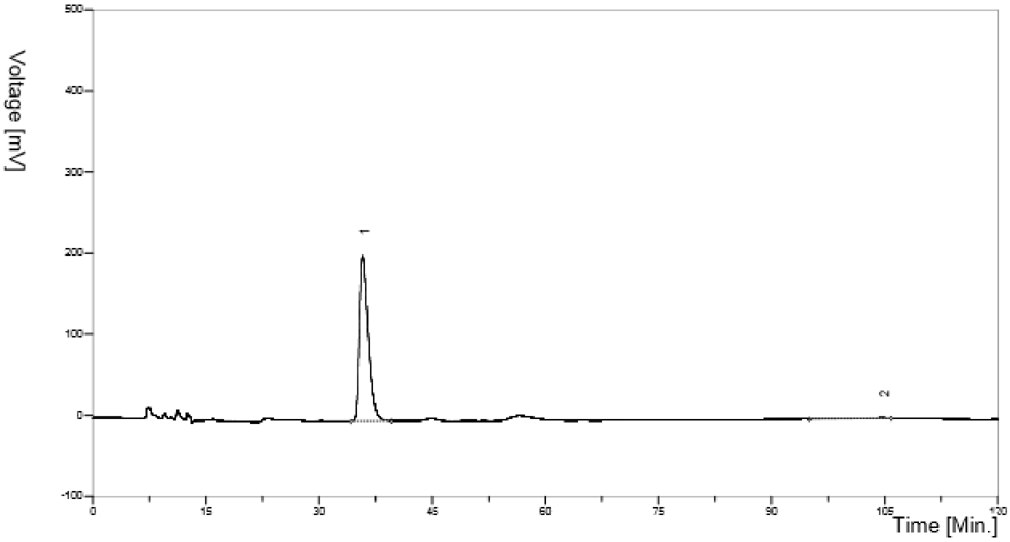


Integration Result

| # | Ret. Time(min) | Area(mv.sec) | Area Percentage(%) |
|---|---|---|---|
| 1 | 35.78 | 16491.14 | 99.1777 |
| 2 | 104.70 | 136.74 | 0.8223 |
| Total | | 16627.88 | 100 |

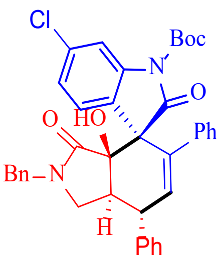


**3fa**

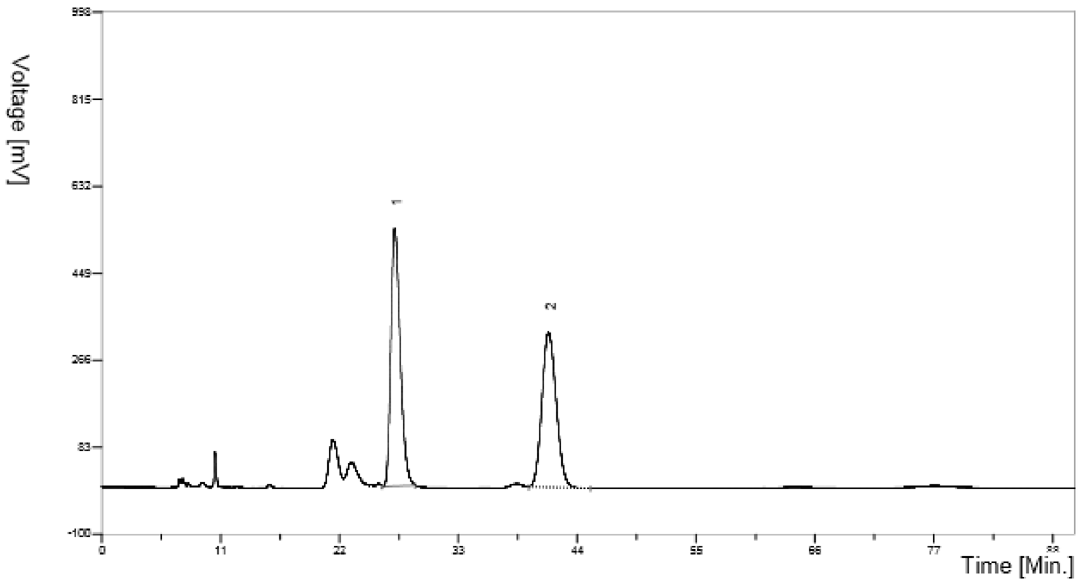


Integration Result

| # | Ret. Time(min) | Area(mv.sec) | Area Percentage(%) |
|---|---|---|---|
| 1 | 27.11 | 32347.71 | 51.9810 |
| 2 | 41.35 | 29882.12 | 48.0190 |
| Total | | 62229.83 | 100 |

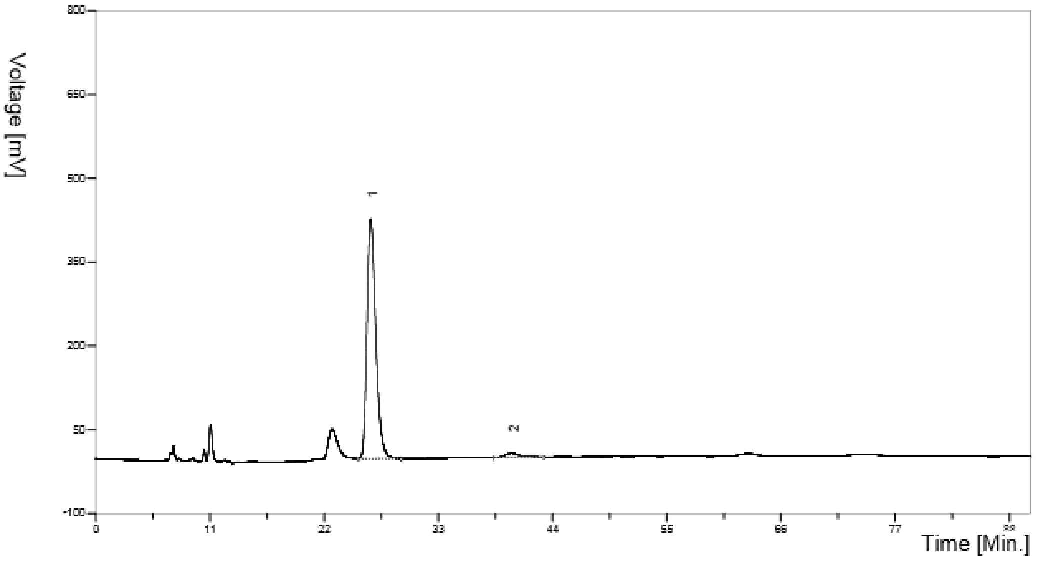


Integration Result

| # | Ret. Time(min) | Area(mv.sec) | Area Percentage(%) |
|---|---|---|---|
| 1 | 26.47 | 25492.97 | 97.0451 |
| 2 | 40.08 | 776.23 | 2.9549 |
| Total | | 26269.20 | 100 |

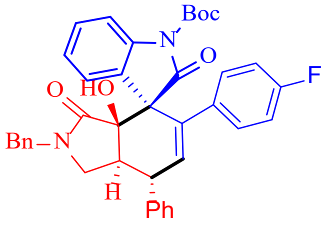


**3ga**

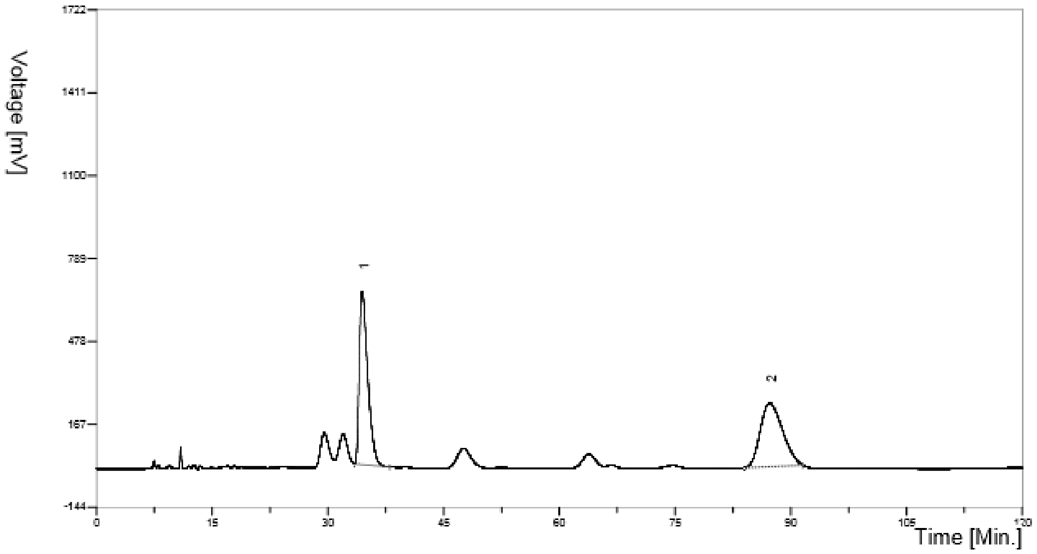


**Integration Result**

| # | Ret. Time(min) | Area(mv.sec) | Area Percentage(%) |
|---|---|---|---|
| 1 | 34.44 | 49517.95 | 50.8478 |
| 2 | 87.27 | 47866.76 | 49.1522 |
| Total | | 97384.71 | 100 |

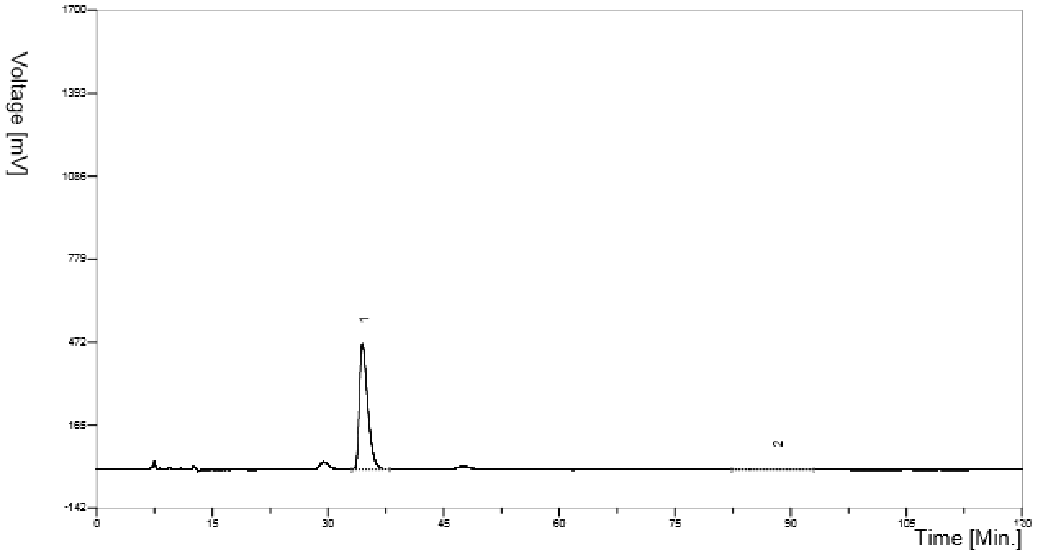


**Integration Result**

| # | Ret. Time(min) | Area(mv.sec) | Area Percentage(%) |
|---|---|---|---|
| 1 | 34.46 | 35020.00 | 98.3651 |
| 2 | 88.07 | 582.05 | 1.6349 |
| Total | | 35602.05 | 100 |

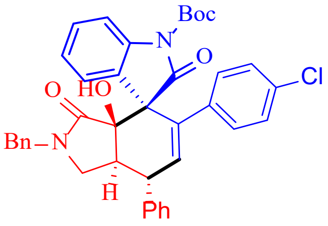


**3ha**

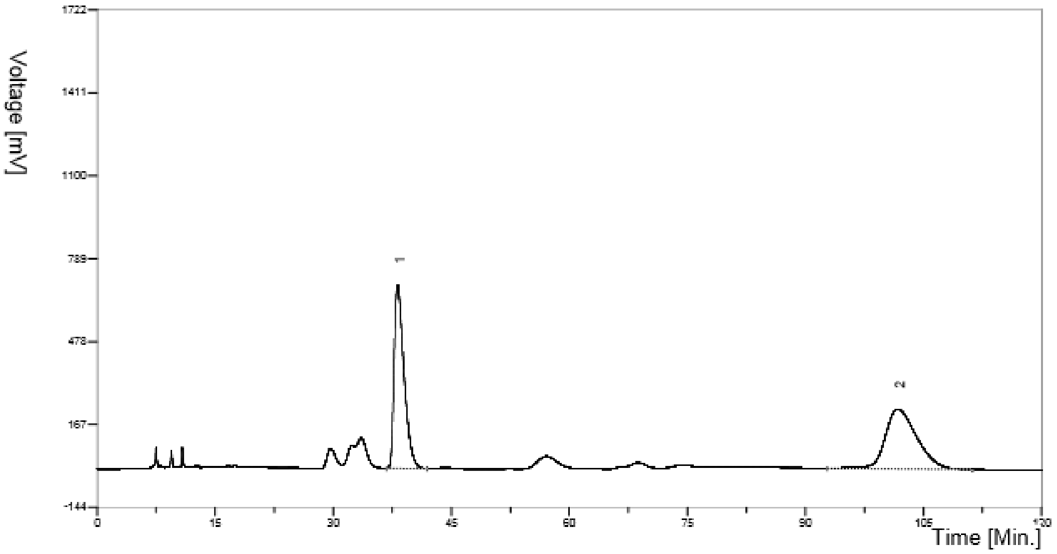


**Integration Result**

| # | Ret. Time(min) | Area(mv.sec) | Area Percentage(%) |
|---|---|---|---|
| 1 | 38.19 | 60153.10 | 49.0332 |
| 2 | 101.75 | 62525.08 | 50.9668 |
| Total | | 122678.18 | 100 |

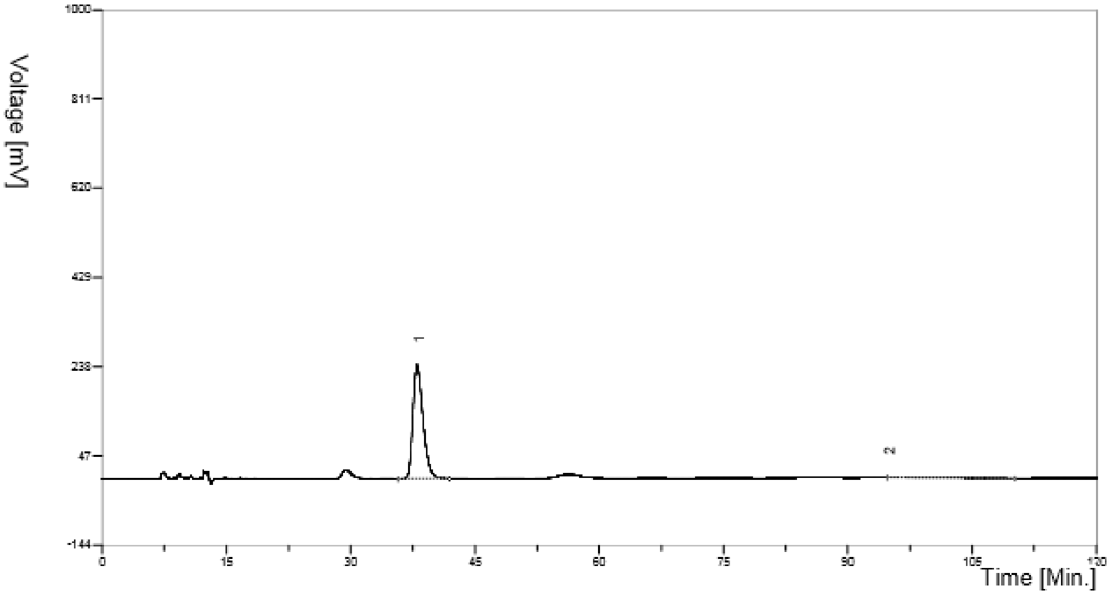


**Integration Result**

| # | Ret. Time(min) | Area(mv.sec) | Area Percentage(%) |
|---|---|---|---|
| 1 | 38.00 | 20356.09 | 99.9661 |
| 2 | 94.81 | 6.91 | 0.0339 |
| Total | | 20363.00 | 100 |

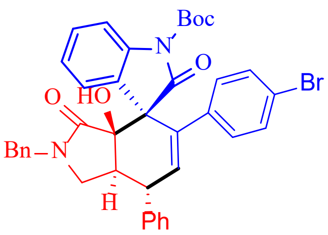


**3ia**

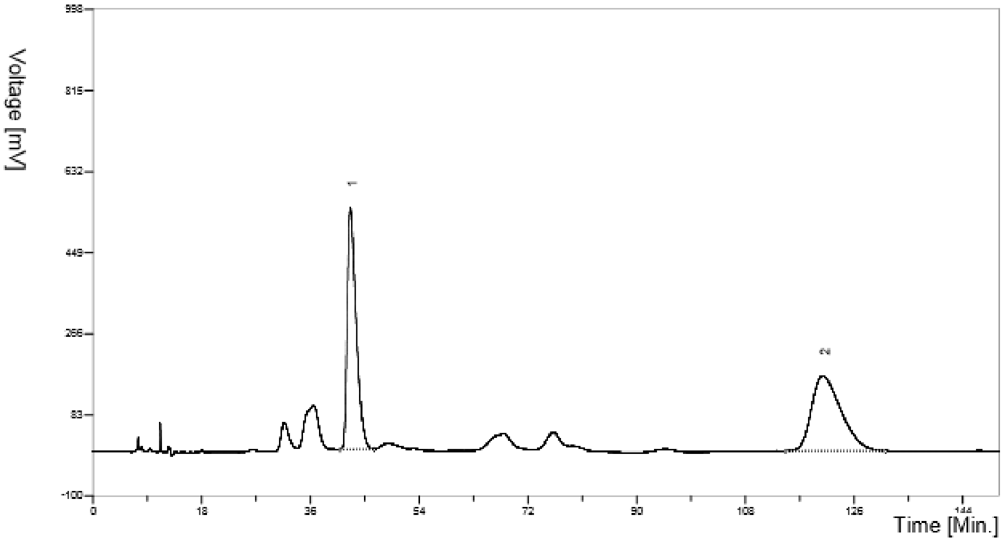


**Integration Result**

| # | Ret. Time(min) | Area(mv.sec) | Area Percentage(%) |
|---|---|---|---|
| 1 | 42.62 | 55950.97 | 49.3638 |
| 2 | 120.84 | 57393.10 | 50.6362 |
| Total | | 113344.07 | 100 |

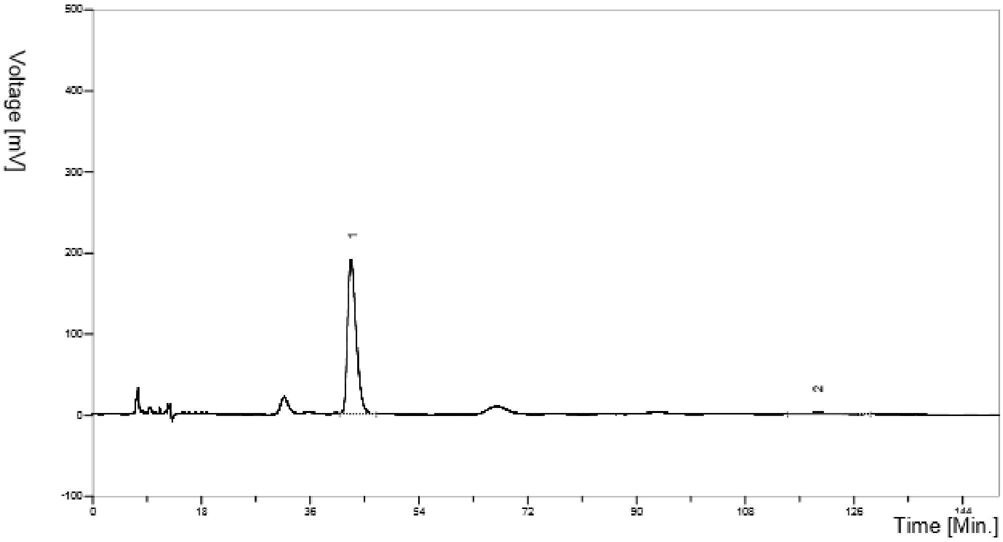


**Integration Result**

| # | Ret. Time(min) | Area(mv.sec) | Area Percentage(%) |
|---|---|---|---|
| 1 | 42.75 | 18836.51 | 98.1691 |
| 2 | 119.76 | 351.31 | 1.8309 |
| Total | | 19187.82 | 100 |

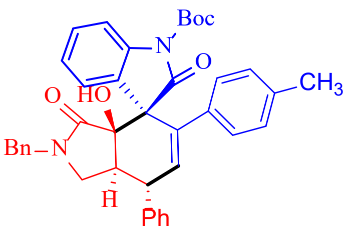


**3ja**

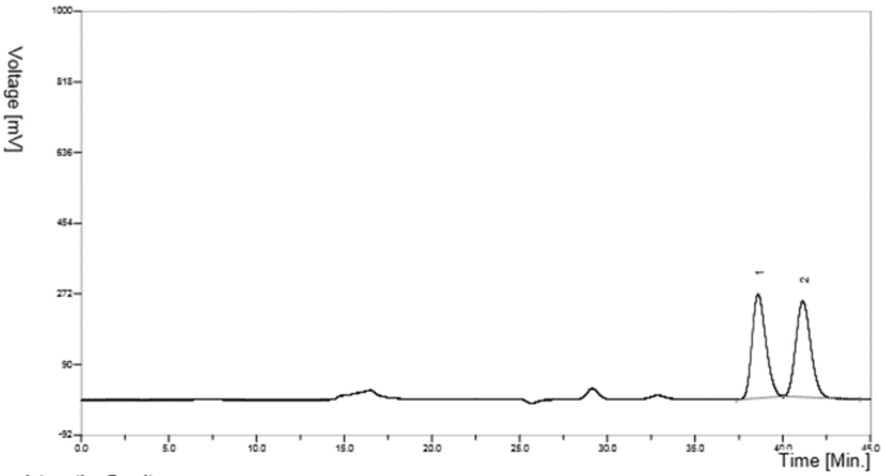


**Integration Result**

| # | Ret. Time(min) | Area(mv.sec) | Area Percentage(%) |
|---|---|---|---|
| 1 | 38.60 | 14544.78 | 50.0983 |
| 2 | 41.15 | 14487.73 | 49.9017 |
| Total | | 29032.51 | 100 |

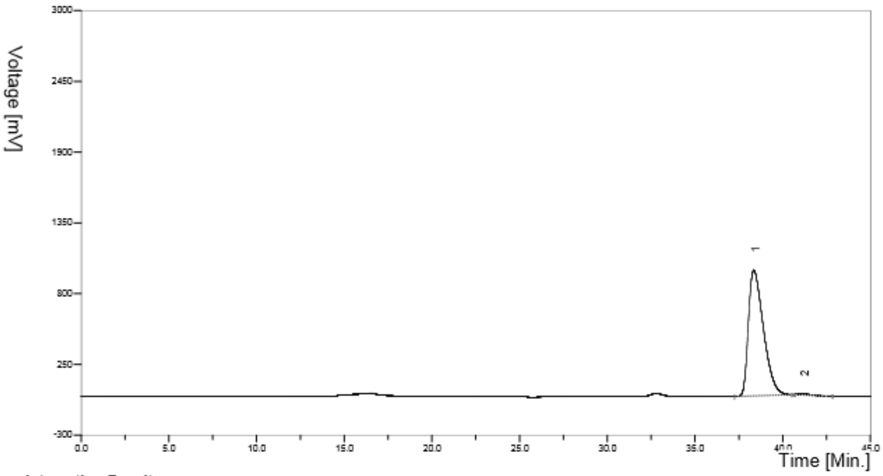


**Integration Result**

| # | Ret. Time(min) | Area(mv.sec) | Area Percentage(%) |
|---|---|---|---|
| 1 | 38.36 | 58182.26 | 99.5042 |
| 2 | 41.16 | 289.88 | 0.4958 |
| Total | | 58472.14 | 100 |

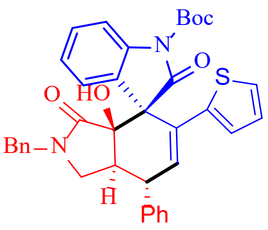


**3ka**

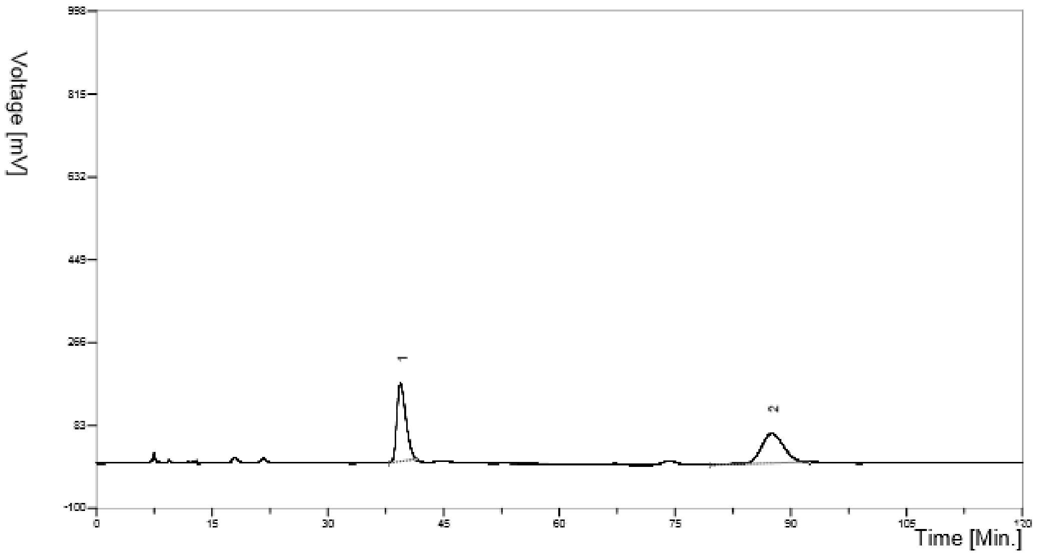

**Integration Result**

| # | Ret. Time(min) | Area(mv.sec) | Area Percentage(%) |
|---|---|---|---|
| 1 | 39.42 | 13979.33 | 51.6202 |
| 2 | 87.55 | 13101.82 | 48.3798 |
| Total | | 27081.15 | 100 |

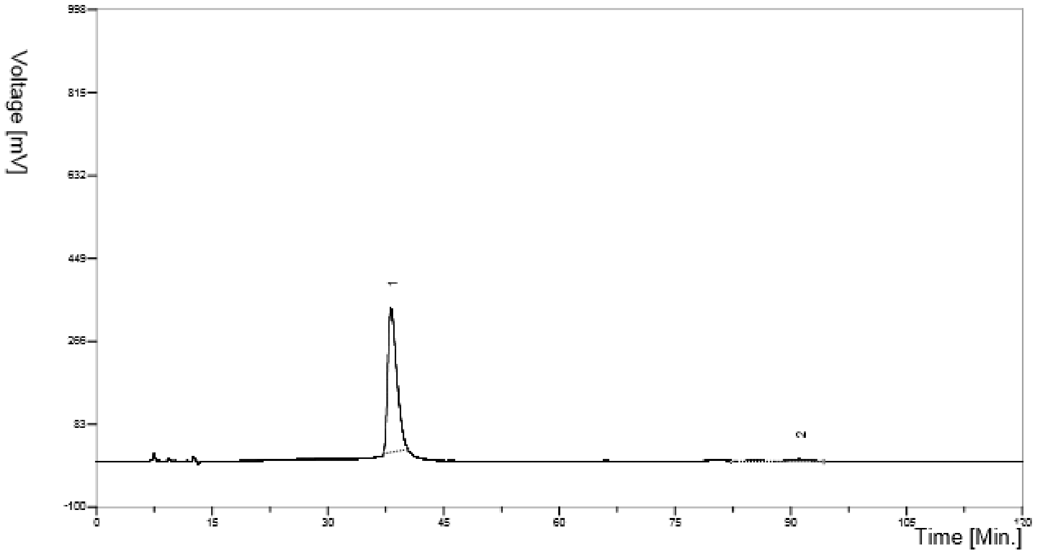

**Integration Result**

| # | Ret. Time(min) | Area(mv.sec) | Area Percentage(%) |
|---|---|---|---|
| 1 | 38.18 | 25678.96 | 97.0216 |
| 2 | 91.08 | 788.29 | 2.9784 |
| Total | | 26467.26 | 100 |

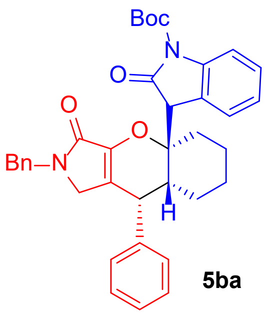


**5ba**

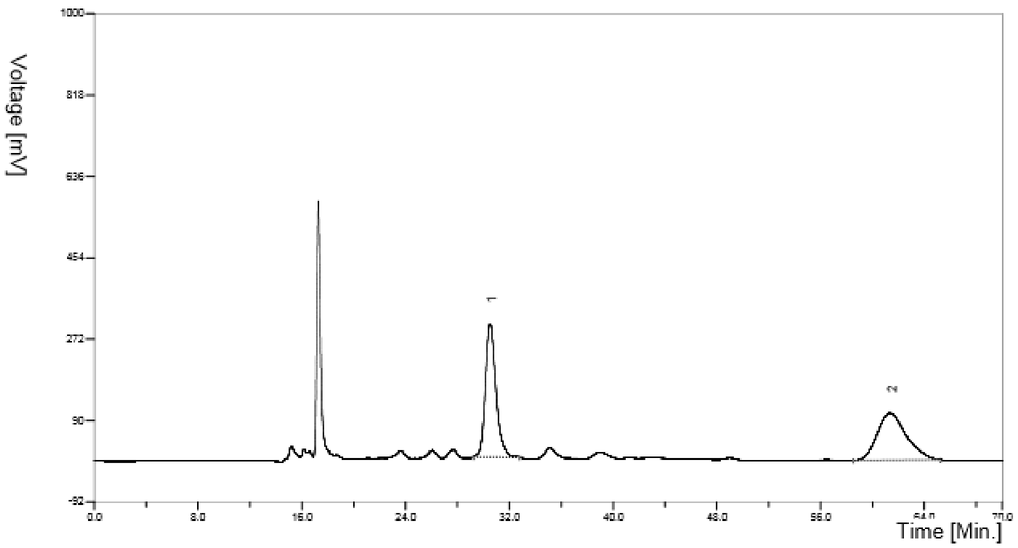

Integration Result

| # | Ret. Time(min) | Area(mv.sec) | Area Percentage(%) |
|---|---|---|---|
| 1 | 30.52 | 16856.63 | 50.6475 |
| 2 | 61.34 | 16425.62 | 49.3525 |
| Total | | 33282.25 | 100 |

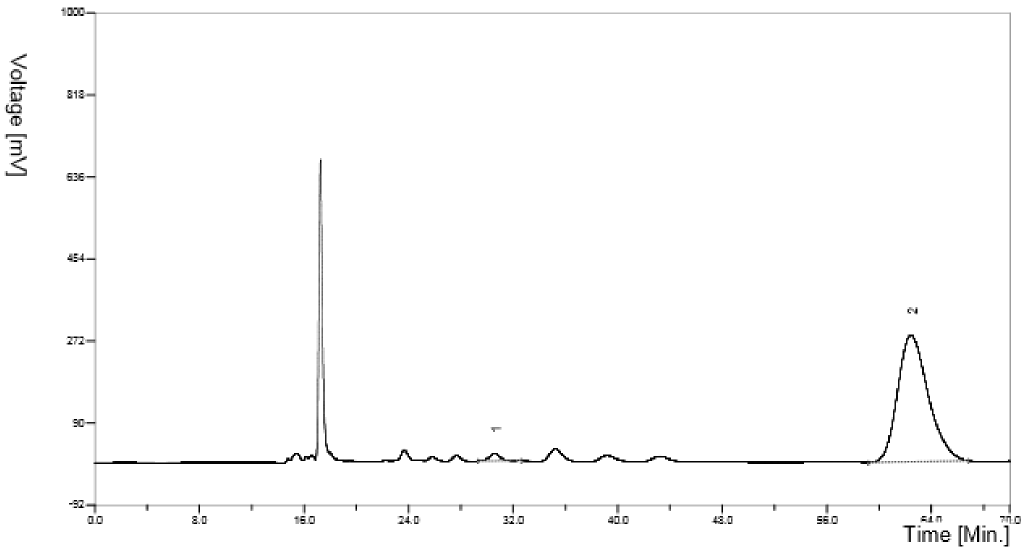

Integration Result

| # | Ret. Time(min) | Area(mv.sec) | Area Percentage(%) |
|---|---|---|---|
| 1 | 30.57 | 917.37 | 1.9748 |
| 2 | 62.43 | 45536.81 | 98.0252 |
| Total | | 46454.18 | 100 |

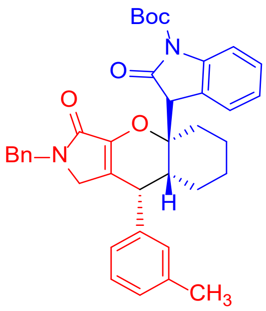


**5bb**

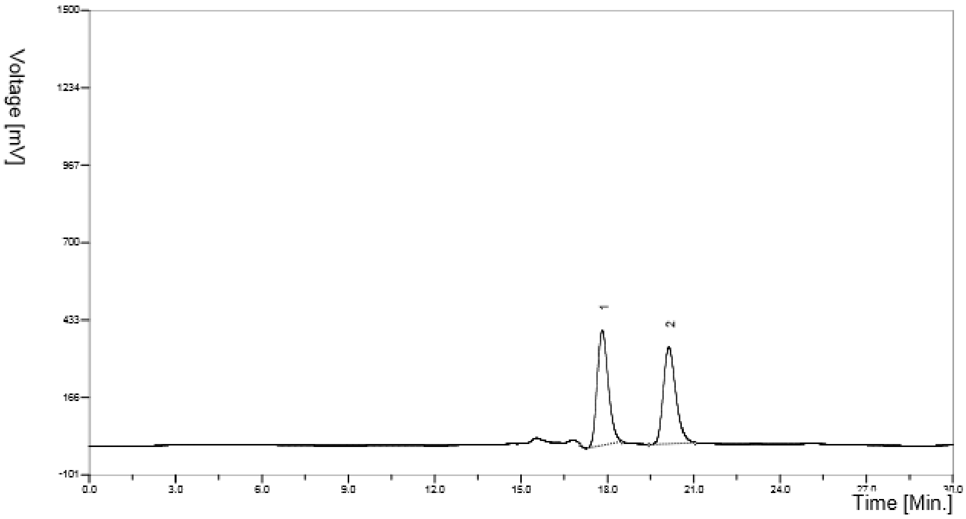


Integration Result

| # | Ret. Time(min) | Area(mv.sec) | Area Percentage(%) |
|---|---|---|---|
| 1 | 17.82 | 9676.96 | 48.8258 |
| 2 | 20.13 | 10142.39 | 51.1742 |
| Total | | 19819.35 | 100 |

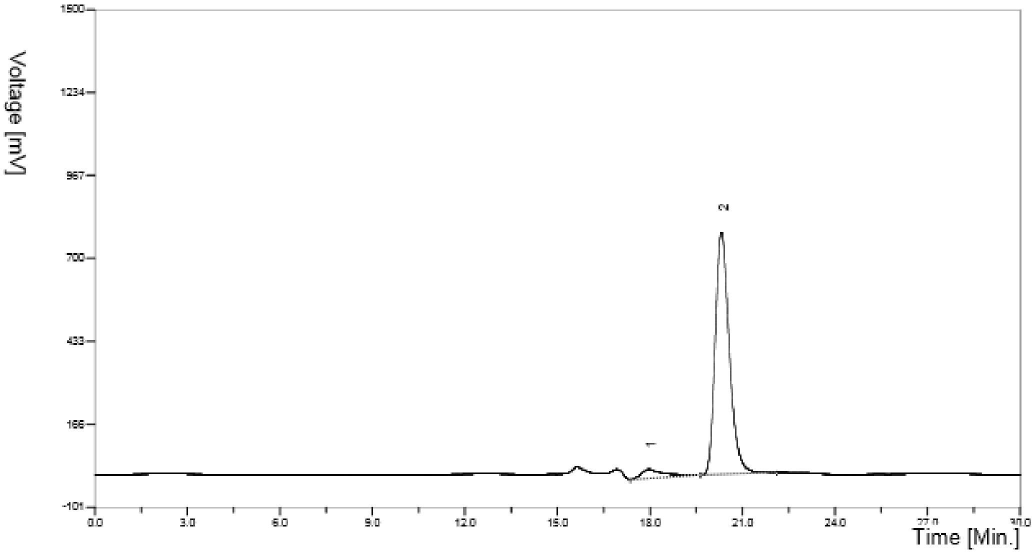


Integration Result

| # | Ret. Time(min) | Area(mv.sec) | Area Percentage(%) |
|---|---|---|---|
| 1 | 17.97 | 1499.95 | 5.7295 |
| 2 | 20.32 | 24679.71 | 94.2705 |
| Total | | 26179.66 | 100 |

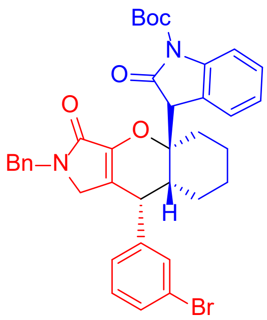


**5bc**

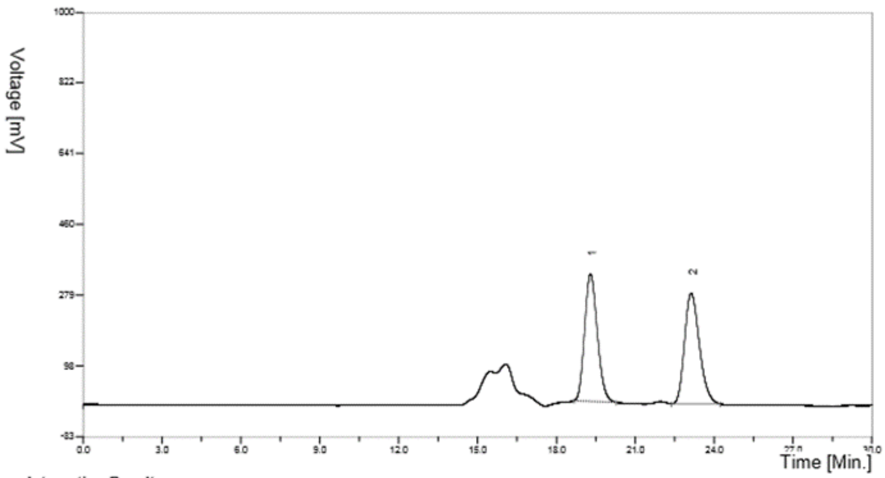


**Integration Result**

| # | Ret. Time(min) | Area(mv.sec) | Area Percentage(%) |
|---|---|---|---|
| 1 | 19.29 | 10942.15 | 49.6063 |
| 2 | 23.13 | 11115.83 | 50.3937 |
| Total | | 22057.98 | 100 |

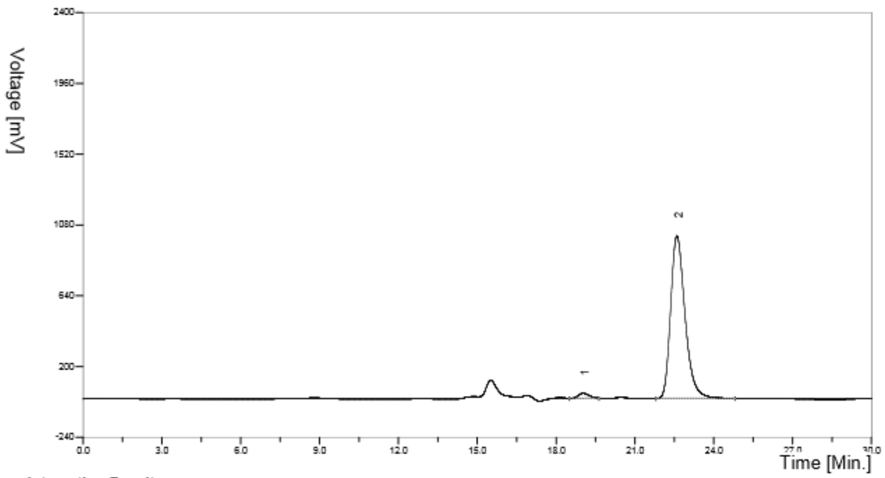


**Integration Result**

| # | Ret. Time(min) | Area(mv.sec) | Area Percentage(%) |
|---|---|---|---|
| 1 | 19.03 | 853.34 | 2.1741 |
| 2 | 22.60 | 38397.33 | 97.8259 |
| Total | | 39250.67 | 100 |

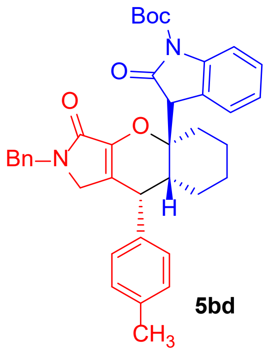


**5bd**

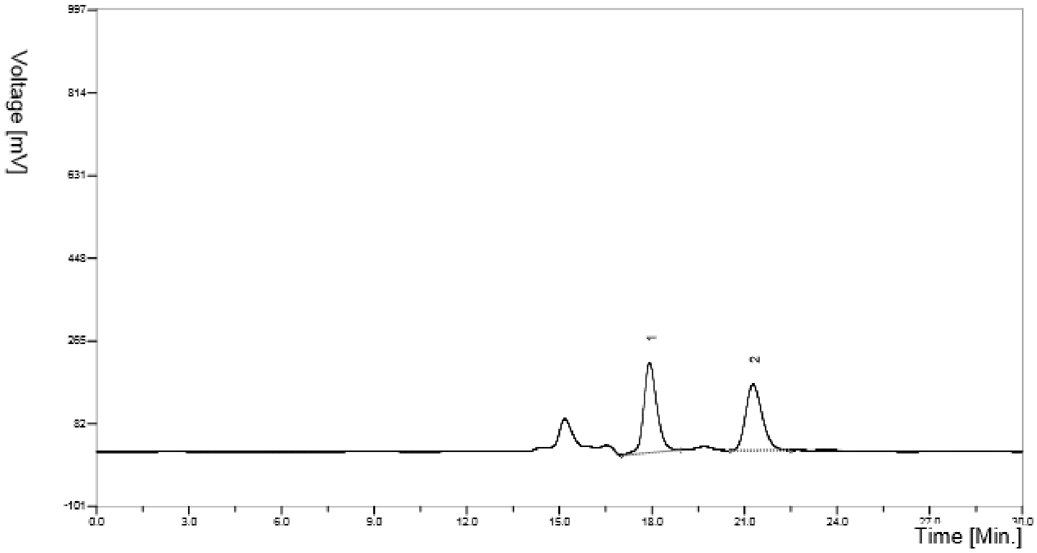


**Integration Result**

| # | Ret. Time(min) | Area(mv.sec) | Area Percentage(%) |
|---|---|---|---|
| 1 | 17.92 | 5869.71 | 51.8173 |
| 2 | 21.28 | 5457.99 | 48.1827 |
| Total | | 11327.70 | 100 |

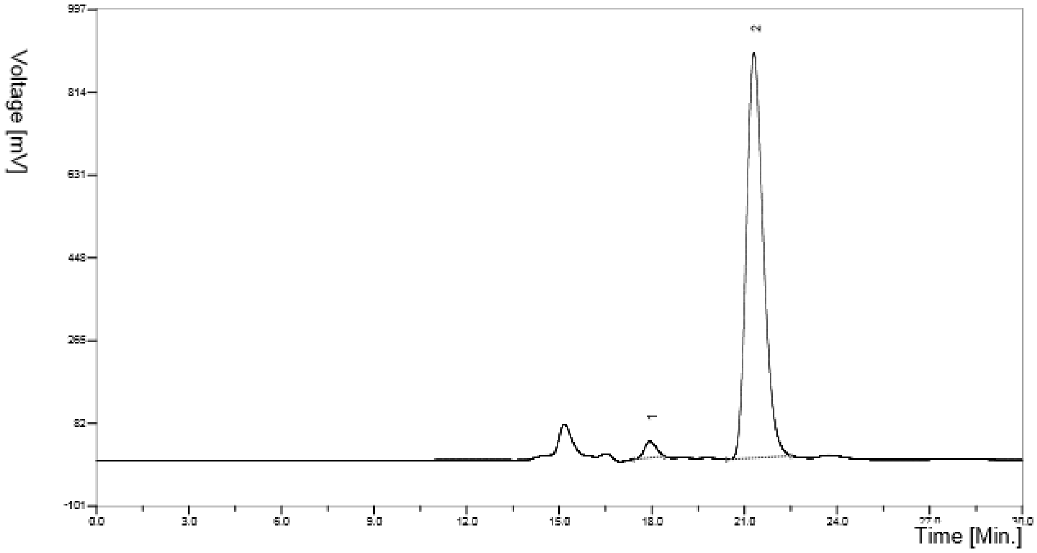


**Integration Result**

| # | Ret. Time(min) | Area(mv.sec) | Area Percentage(%) |
|---|---|---|---|
| 1 | 17.93 | 972.10 | 2.7717 |
| 2 | 21.31 | 34100.31 | 97.2283 |
| Total | | 35072.41 | 100 |

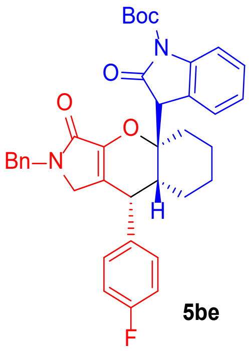


**5be**

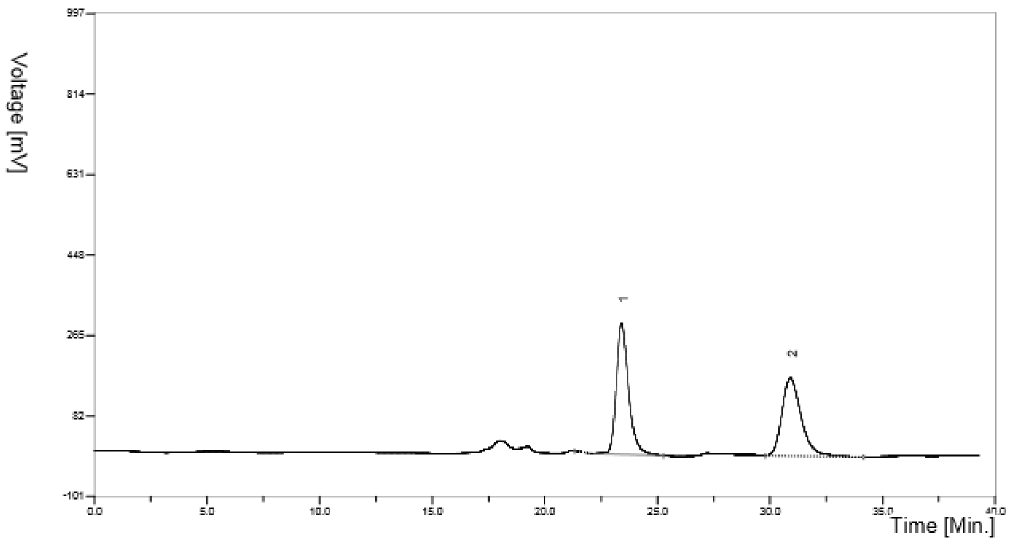


Integration Result

| # | Ret. Time(min) | Area(mv.sec) | Area Percentage(%) |
|---|---|---|---|
| 1 | 23.42 | 10909.58 | 50.8614 |
| 2 | 30.90 | 10540.06 | 49.1386 |
| Total | | 21449.64 | 100 |

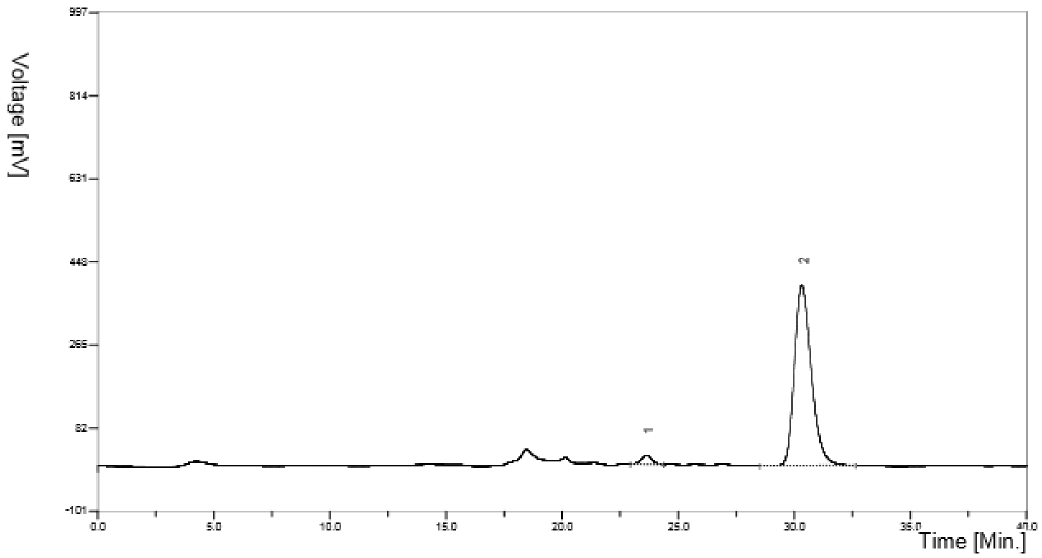


Integration Result

| # | Ret. Time(min) | Area(mv.sec) | Area Percentage(%) |
|---|---|---|---|
| 1 | 23.63 | 624.22 | 3.0058 |
| 2 | 30.32 | 20142.89 | 96.9942 |
| Total | | 20767.11 | 100 |

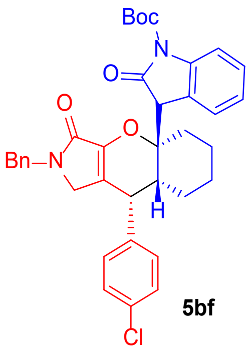

**5bf**

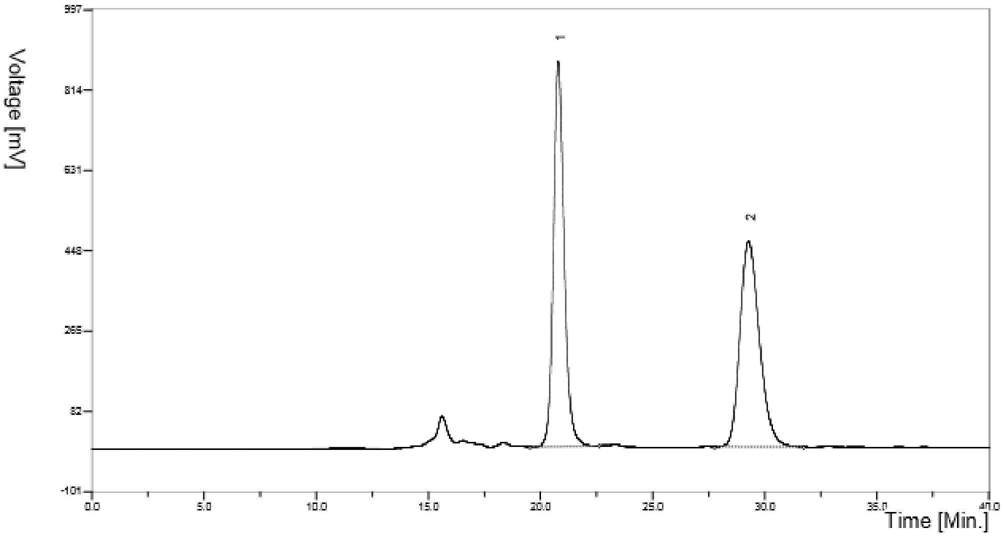


**Integration Result**

| # | Ret. Time(min) | Area(mv.sec) | Area Percentage(%) |
|---|---|---|---|
| 1 | 20.80 | 29084.30 | 50.7134 |
| 2 | 29.28 | 28266.01 | 49.2866 |
| Total | | 57350.31 | 100 |

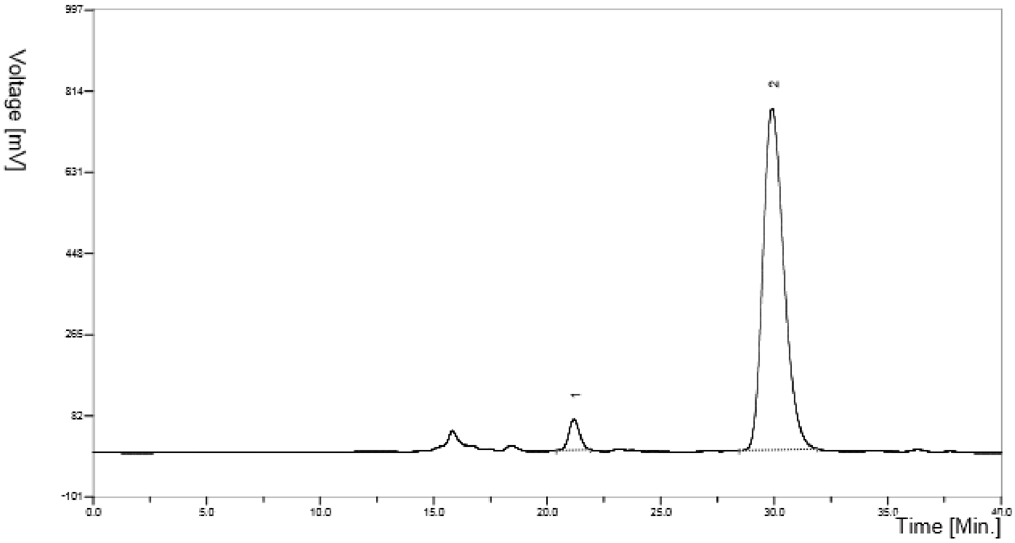


**Integration Result**

| # | Ret. Time(min) | Area(mv.sec) | Area Percentage(%) |
|---|---|---|---|
| 1 | 21.17 | 2308.45 | 4.4606 |
| 2 | 29.91 | 49443.67 | 95.5394 |
| Total | | 51752.12 | 100 |

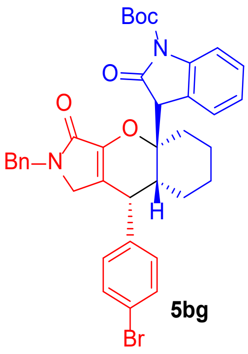


**5bg**

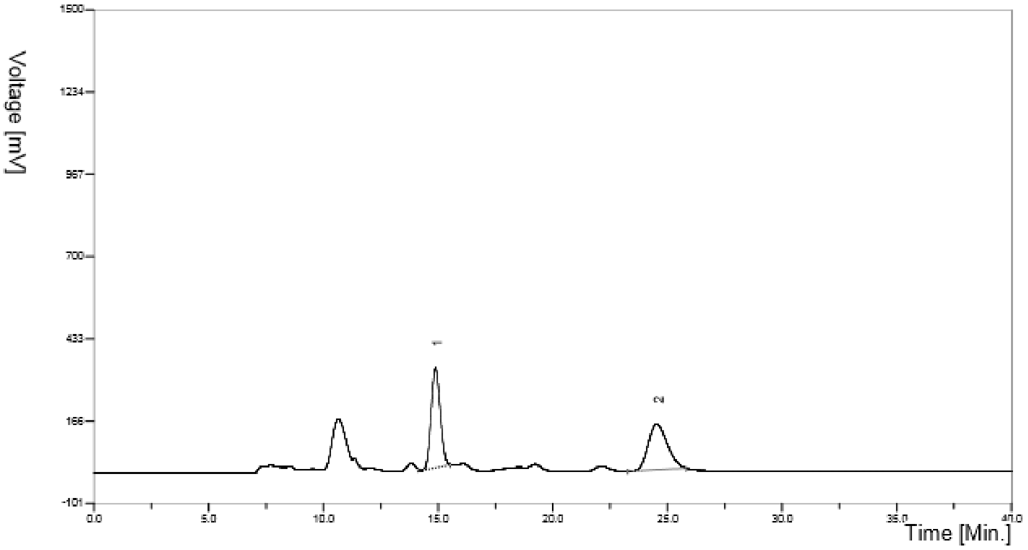


**Integration Result**

| # | Ret. Time(min) | Area(mv.sec) | Area Percentage(%) |
|---|---|---|---|
| 1 | 14.89 | 8661.36 | 50.2719 |
| 2 | 24.54 | 8567.66 | 49.7281 |
| Total | | 17229.02 | 100 |

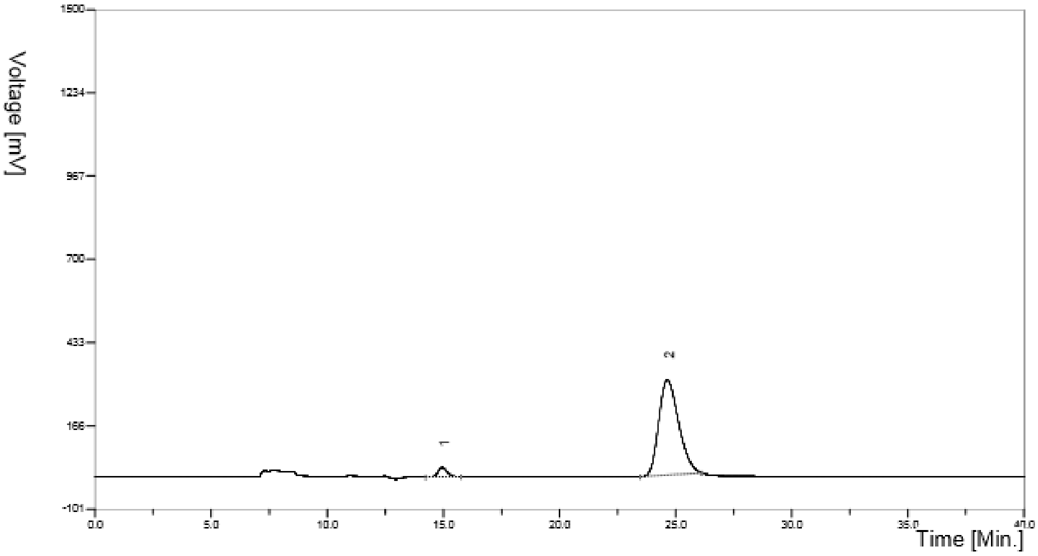


**Integration Result**

| # | Ret. Time(min) | Area(mv.sec) | Area Percentage(%) |
|---|---|---|---|
| 1 | 14.95 | 837.31 | 4.4135 |
| 2 | 24.64 | 18134.38 | 95.5865 |
| Total | | 18971.70 | 100 |

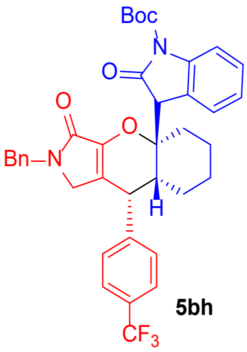


**5bh**

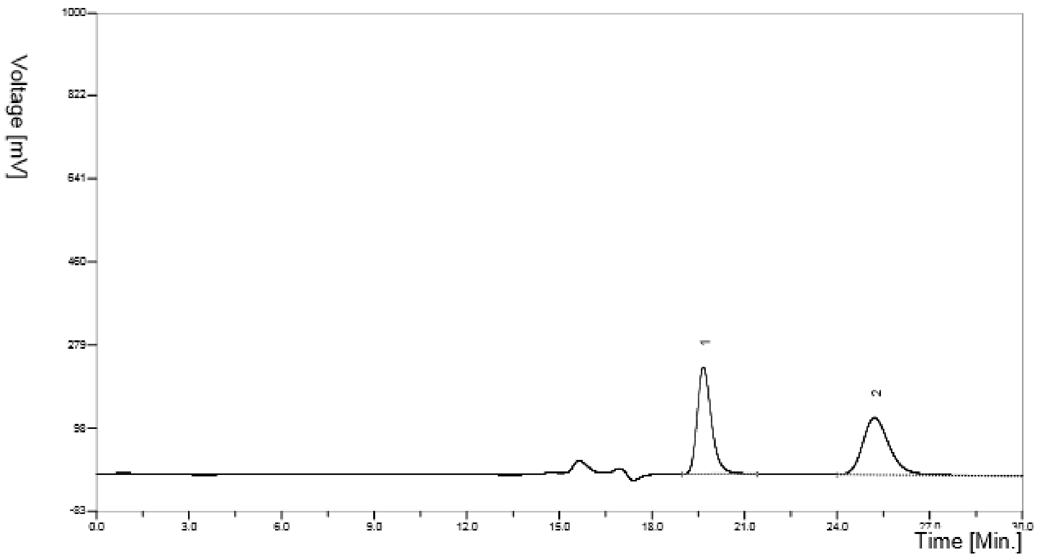


**Integration Result**

| # | Ret. Time(min) | Area(mv.sec) | Area Percentage(%) |
|---|---|---|---|
| 1 | 19.67 | 7402.90 | 49.7310 |
| 2 | 25.21 | 7482.98 | 50.2690 |
| Total | | 14885.89 | 100 |

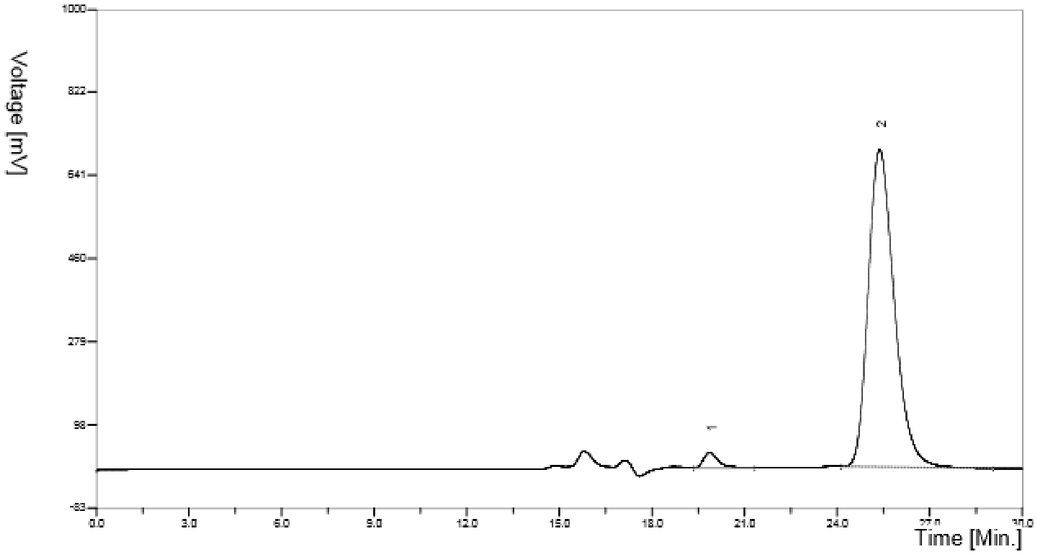


**Integration Result**

| # | Ret. Time(min) | Area(mv.sec) | Area Percentage(%) |
|---|---|---|---|
| 1 | 19.87 | 1128.50 | 2.7146 |
| 2 | 25.37 | 40443.62 | 97.2854 |
| Total | | 41572.12 | 100 |

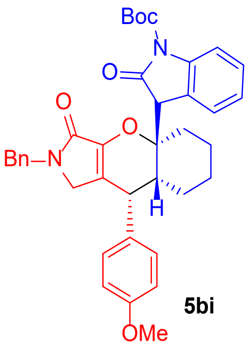

**5bi**

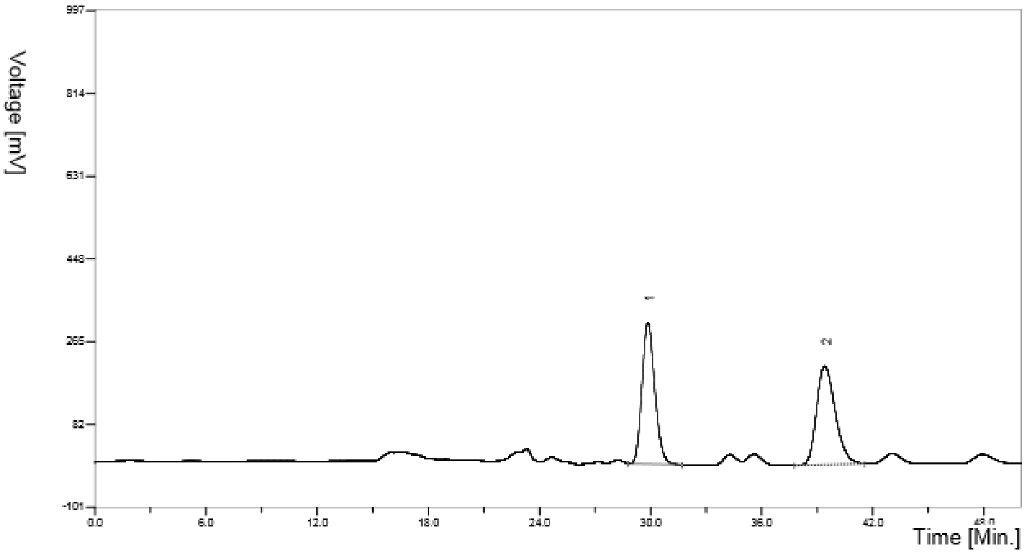


**Integration Result**

| # | Ret. Time(min) | Area(mv.sec) | Area Percentage(%) |
|---|---|---|---|
| 1 | 29.86 | 15056.11 | 49.5351 |
| 2 | 39.41 | 15338.75 | 50.4649 |
| Total | | 30394.86 | 100 |

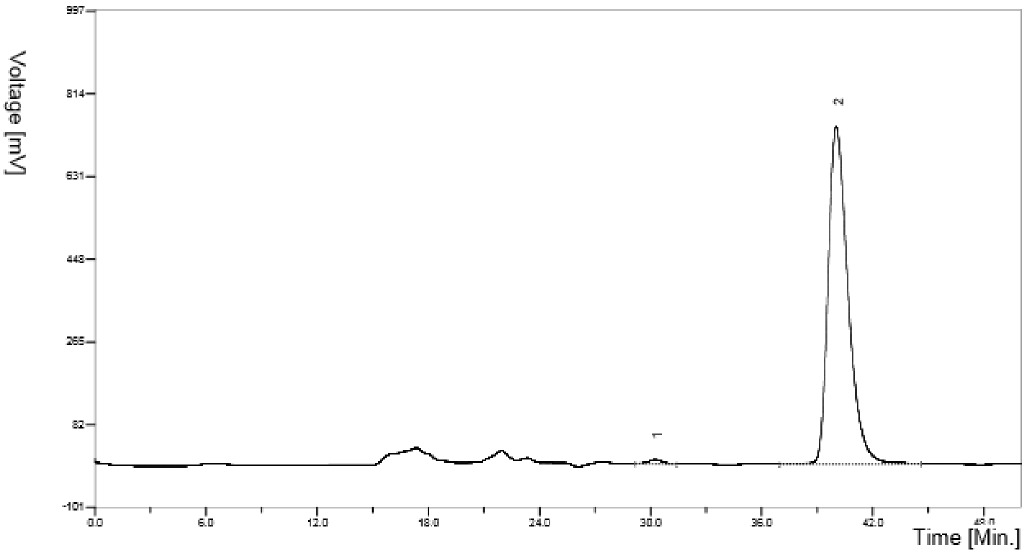


**Integration Result**

| # | Ret. Time(min) | Area(mv.sec) | Area Percentage(%) |
|---|---|---|---|
| 1 | 30.26 | 513.12 | 0.9101 |
| 2 | 40.04 | 55865.32 | 99.0899 |
| Total | | 56378.44 | 100 |

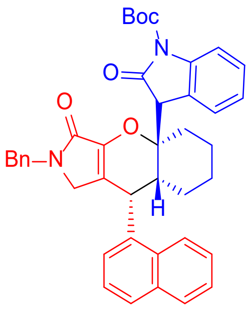

**5bj**

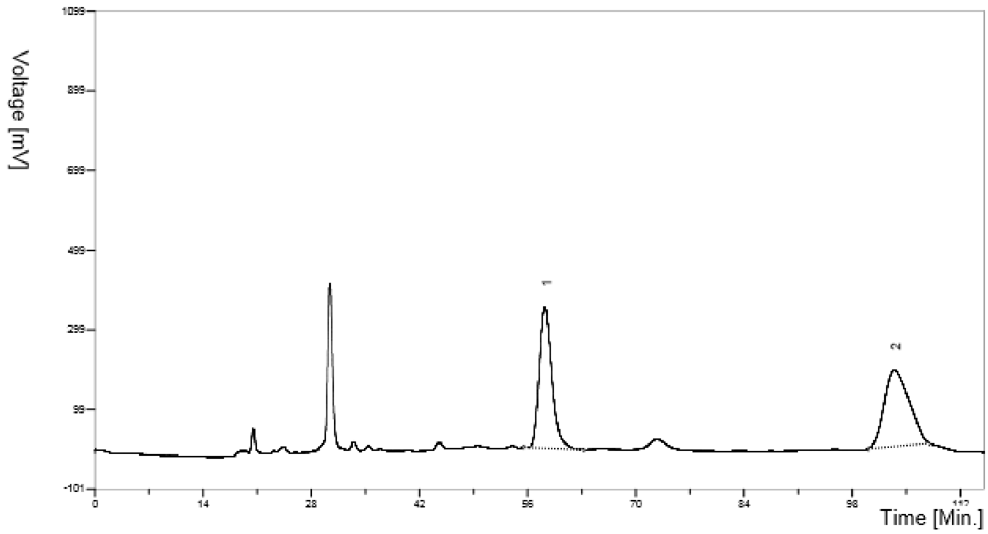


**Integration Result**

| # | Ret. Time(min) | Area(mv.sec) | Area Percentage(%) |
|---|---|---|---|
| 1 | 58.23 | 39646.07 | 48.2944 |
| 2 | 103.44 | 42446.48 | 51.7056 |
| Total | | 82092.56 | 100 |

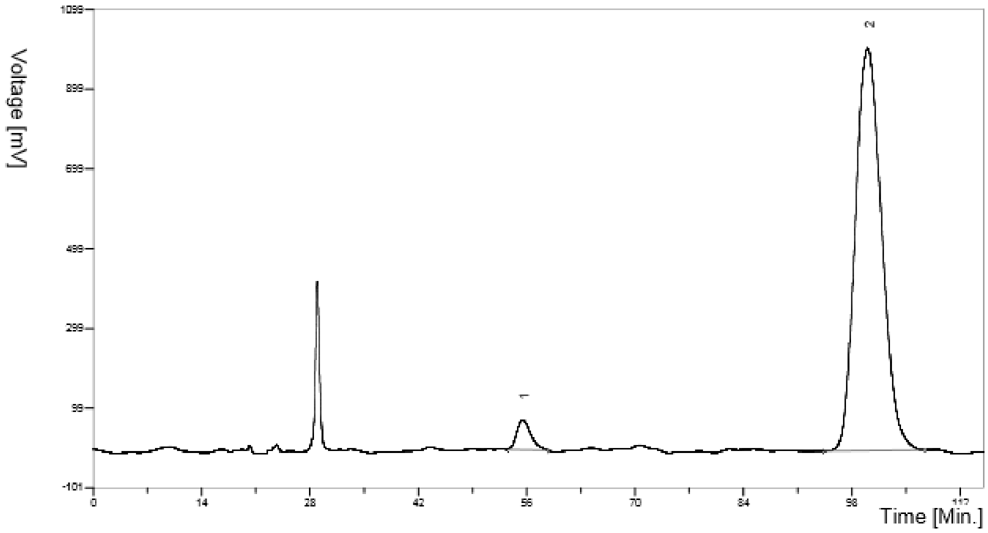


**Integration Result**

| # | Ret. Time(min) | Area(mv.sec) | Area Percentage(%) |
|---|---|---|---|
| 1 | 55.48 | 9517.09 | 3.8328 |
| 2 | 100.06 | 238789.31 | 96.1672 |
| Total | | 248306.39 | 100 |

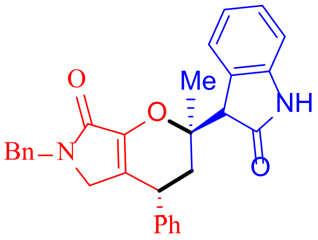

**5aa**

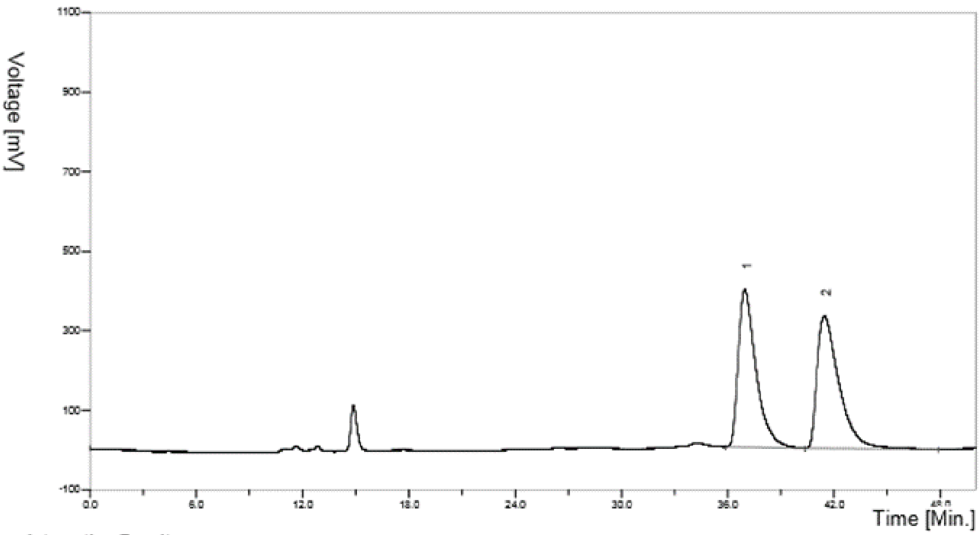


**Integration Result**

| # | Ret. Time(min) | Area(mv.sec) | Area Percentage(%) |
|---|---|---|---|
| 1 | 36.97 | 27511.14 | 49.0499 |
| 2 | 41.45 | 28576.89 | 50.9501 |
| Total | | 56088.03 | 100 |

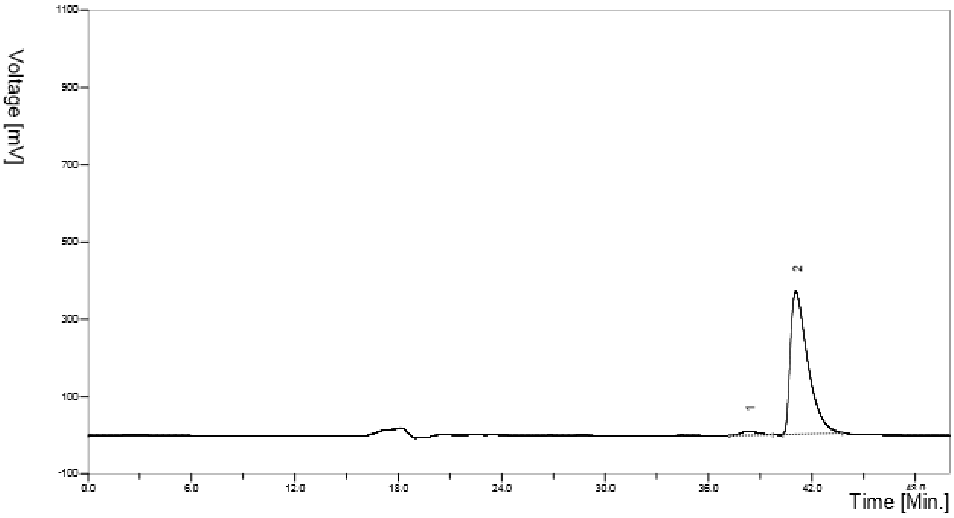


**Integration Result**

| # | Ret. Time(min) | Area(mv.sec) | Area Percentage(%) |
|---|---|---|---|
| 1 | 38.32 | 757.24 | 2.8530 |
| 2 | 41.08 | 25784.50 | 97.1470 |
| Total | | 26541.74 | 100 |

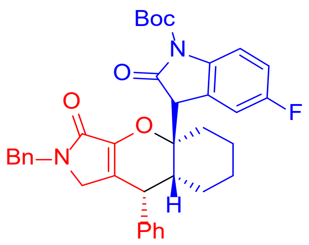


**5ca**

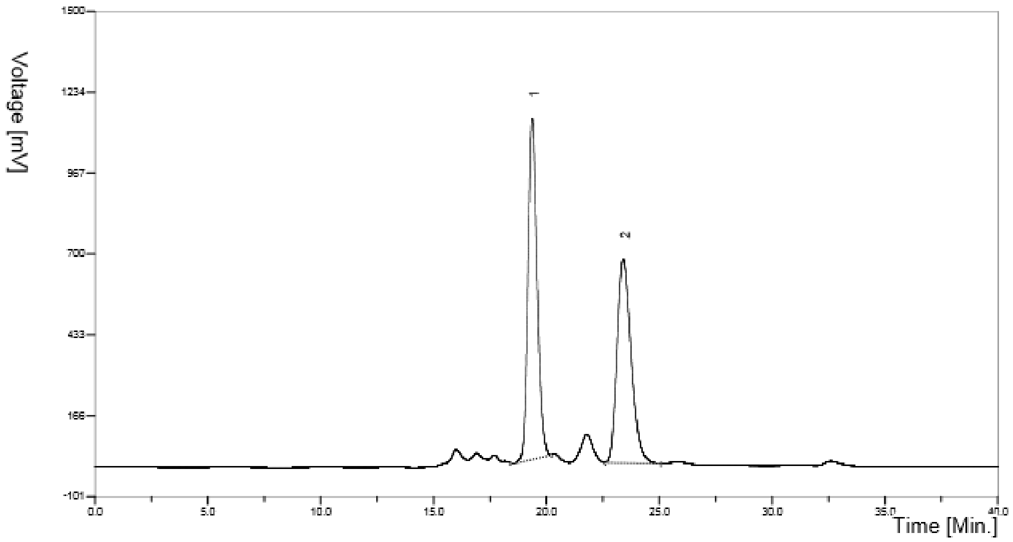


**Integration Result**

| # | Ret. Time(min) | Area(mv.sec) | Area Percentage(%) |
|---|---|---|---|
| 1 | 19.38 | 31392.56 | 51.8183 |
| 2 | 23.40 | 29189.48 | 48.1817 |
| Total | | 60582.04 | 100 |

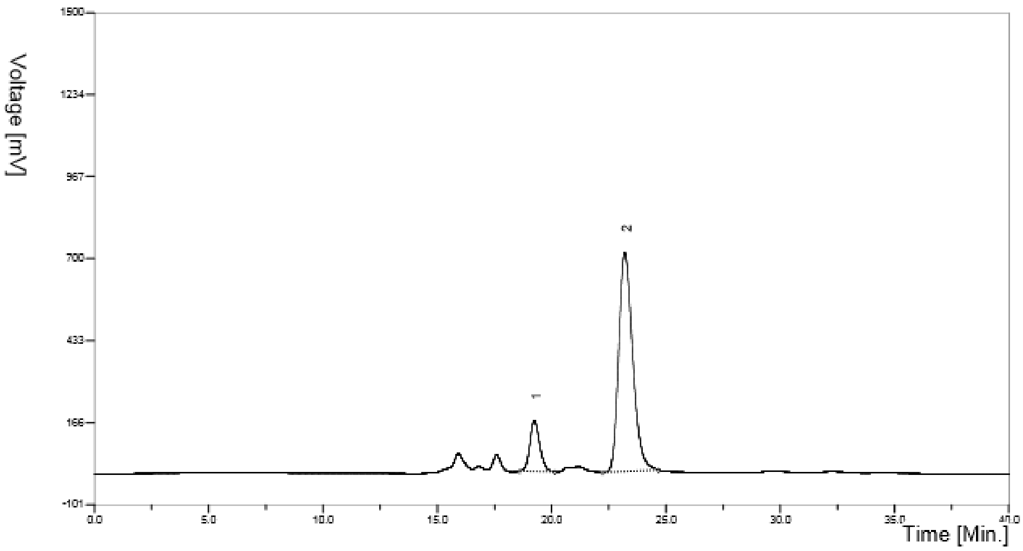


**Integration Result**

| # | Ret. Time(min) | Area(mv.sec) | Area Percentage(%) |
|---|---|---|---|
| 1 | 19.24 | 4984.75 | 14.2060 |
| 2 | 23.20 | 30104.21 | 85.7940 |
| Total | | 35088.96 | 100 |

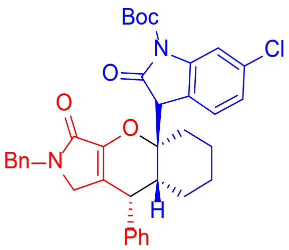


**5da**

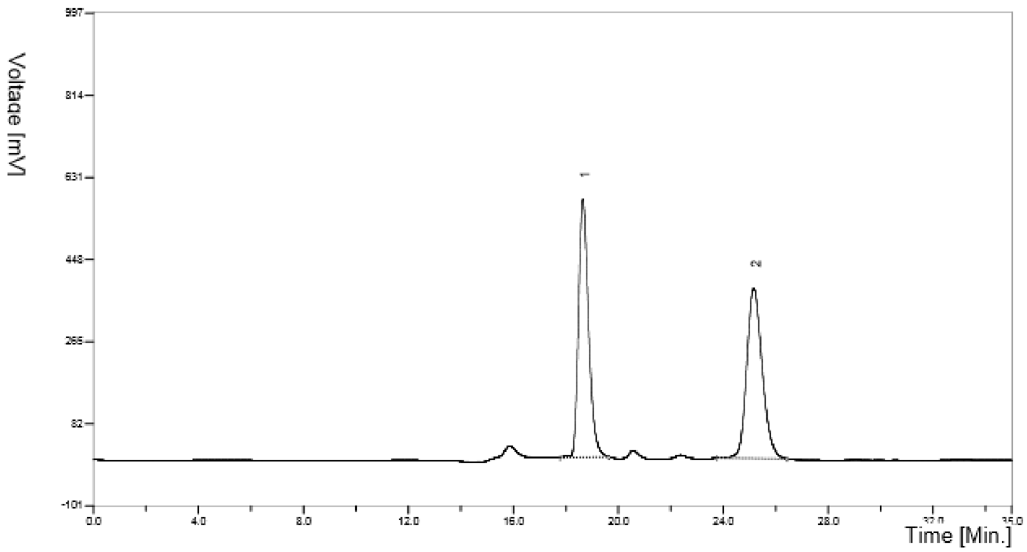


Integration Result

| # | Ret. Time(min) | Area(mv.sec) | Area Percentage(%) |
|---|---|---|---|
| 1 | 18.65 | 15377.61 | 49.8024 |
| 2 | 25.16 | 15499.65 | 50.1976 |
| Total | | 30877.26 | 100 |

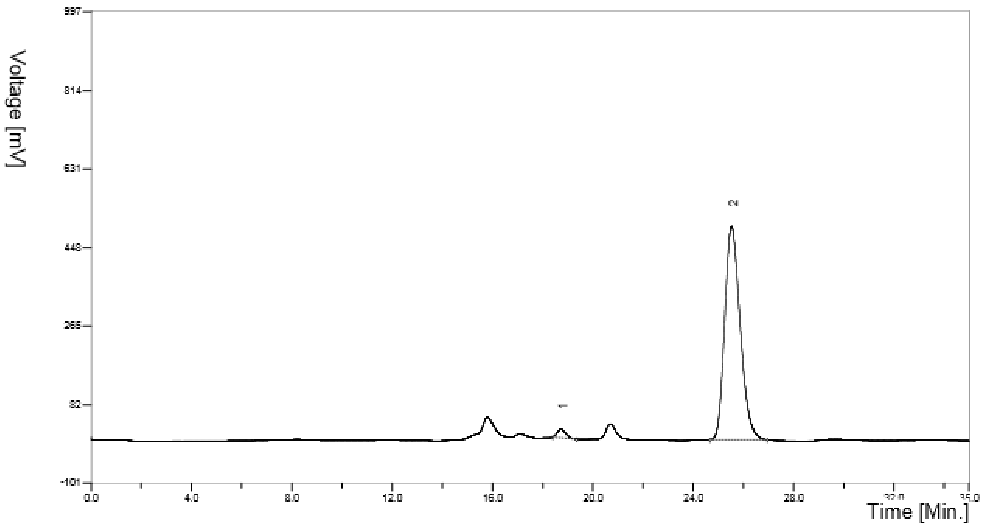


Integration Result

| # | Ret. Time(min) | Area(mv.sec) | Area Percentage(%) |
|---|---|---|---|
| 1 | 18.72 | 464.32 | 2.1274 |
| 2 | 25.53 | 21361.88 | 97.8726 |
| Total | | 21826.21 | 100 |

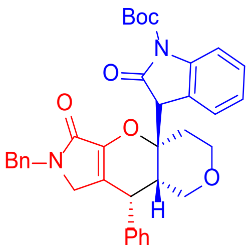


**5ea**

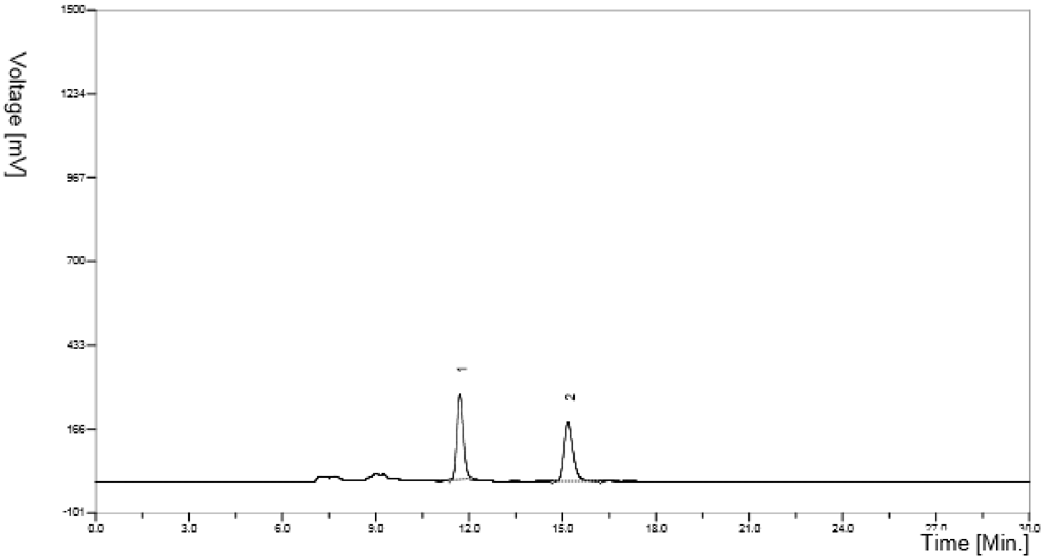


**Integration Result**

| # | Ret. Time(min) | Area(mv.sec) | Area Percentage(%) |
|---|---|---|---|
| 1 | 11.70 | 3831.99 | 50.3863 |
| 2 | 15.17 | 3773.23 | 49.6137 |
| Total | | 7605.22 | 100 |

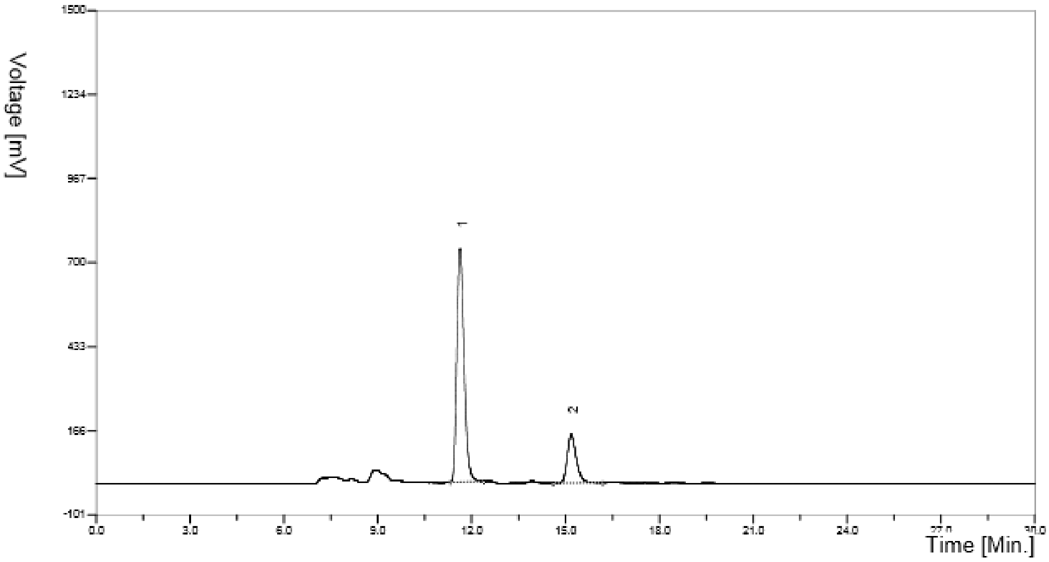


**Integration Result**

| # | Ret. Time(min) | Area(mv.sec) | Area Percentage(%) |
|---|---|---|---|
| 1 | 11.63 | 11887.45 | 79.2166 |
| 2 | 15.18 | 3118.82 | 20.7834 |
| Total | | 15006.27 | 100 |

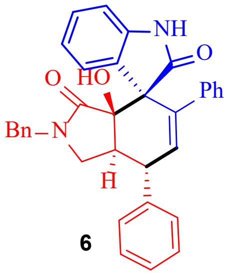


**6**

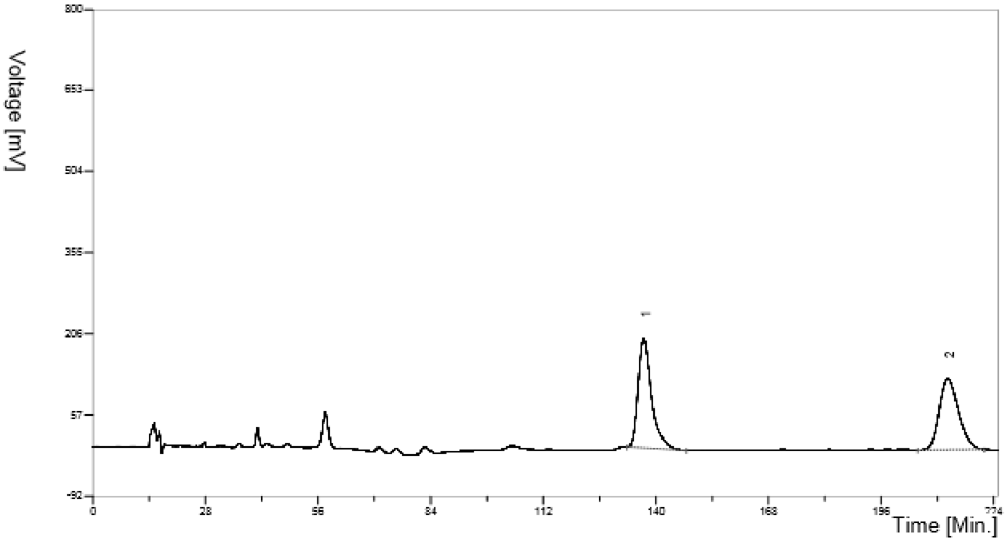


**Integration Result**

| # | Ret. Time(min) | Area(mv.sec) | Area Percentage(%) |
|---|---|---|---|
| 1 | 136.97 | 46701.90 | 51.8229 |
| 2 | 212.58 | 43416.31 | 48.1771 |
| Total | | 90118.21 | 100 |

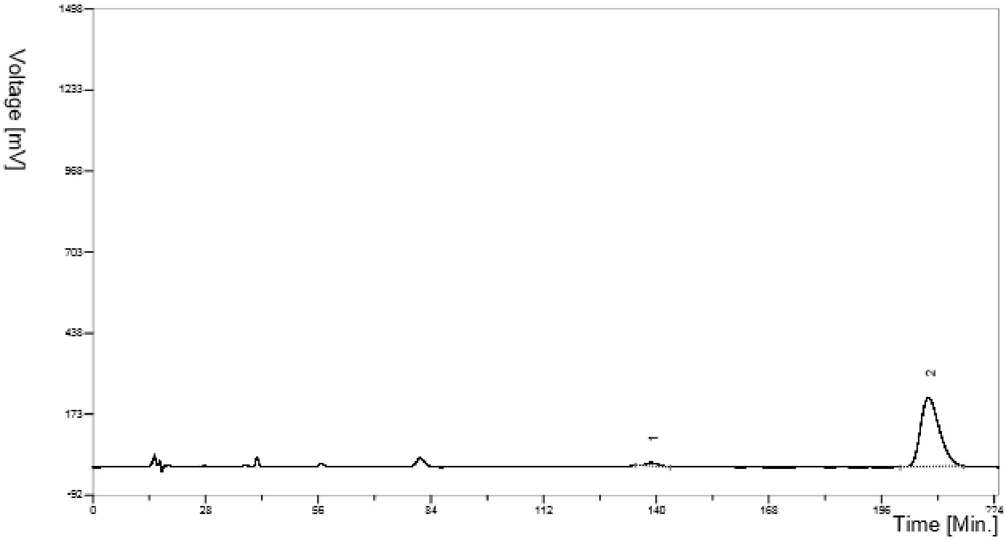


**Integration Result**

| # | Ret. Time(min) | Area(mv.sec) | Area Percentage(%) |
|---|---|---|---|
| 1 | 138.77 | 2224.73 | 2.9457 |
| 2 | 207.68 | 73299.32 | 97.0543 |
| Total | | 75524.05 | 100 |

**Cartesian Coordinates of 5ba and 5ba'**

**5ba**

Optimization energy : E(B3LYP-D3/6-311G(d,p)/IEFPCM(DCM)) = -1919.193139 Hartree

Single point energy : E(M06-2X-D3/def2-TZVPP/SMD(DCM))= -1918.508082 Hartree

| | | | |
|---|---|---|---|
| C | -0.14193 | 1.219986 | -0.81994 |
| C | 0.981801 | 2.109311 | -1.40607 |
| C | 1.798367 | 2.908011 | -0.38869 |
| C | 2.35708 | 1.974633 | 0.683306 |
| C | 1.205903 | 1.277683 | 1.409267 |
| C | 0.263694 | 0.47793 | 0.502649 |
| H | 2.609875 | 3.423711 | -0.91065 |
| H | 1.664115 | 1.467372 | -1.96229 |
| H | 0.527839 | 2.782399 | -2.13821 |
| H | -0.3194 | 0.43225 | -1.5551 |
| H | 3.03999 | 1.251084 | 0.228468 |
| H | 2.944941 | 2.533141 | 1.416861 |
| H | 1.570539 | 0.619197 | 2.199856 |
| H | 0.595335 | 2.041687 | 1.894125 |
| H | 1.183332 | 3.677353 | 0.085835 |
| C | -2.10682 | 0.23994 | 0.850474 |
| C | -2.49731 | 1.047894 | -0.13533 |
| C | -1.52095 | 1.973393 | -0.77281 |
| H | -1.79166 | 2.132865 | -1.82052 |
| C | 0.789353 | -0.97044 | 0.173584 |
| C | 1.20866 | -1.75358 | 1.388444 |
| C | 2.043699 | -1.0055 | -0.70041 |
| C | 2.583594 | -2.00686 | 1.330263 |
| C | 3.248556 | -2.68476 | 2.343169 |
| H | 4.307152 | -2.87925 | 2.279551 |
| C | 0.463452 | -2.20653 | 2.467758 |
| H | -0.60058 | -2.01665 | 2.511377 |
| C | 2.489 | -3.12075 | 3.432406 |
| H | 2.983998 | -3.65416 | 4.235406 |
| C | 1.115503 | -2.89439 | 3.494738 |
| H | 0.547049 | -3.25536 | 4.343406 |
| O | 2.118665 | -0.75171 | -1.87572 |
| N | 3.106392 | -1.46968 | 0.116538 |
| C | 4.472864 | -1.47134 | -0.22329 |

| | | | |
|---|---|---|---|
| O | 5.272819 | -2.21214 | 0.29985 |
| O | 4.715427 | -0.53336 | -1.12486 |
| C | 6.055103 | -0.37619 | -1.75005 |
| C | 7.067916 | 0.036851 | -0.68607 |
| C | 6.437969 | -1.66962 | -2.46345 |
| C | 5.809539 | 0.75121 | -2.74627 |
| H | 6.719842 | 0.927746 | -0.15859 |
| H | 7.232464 | -0.76232 | 0.033997 |
| H | 8.017375 | 0.275349 | -1.17084 |
| H | 5.648873 | -1.96418 | -3.1587 |
| H | 7.354265 | -1.50395 | -3.03417 |
| H | 6.610593 | -2.47832 | -1.75542 |
| H | 6.731303 | 0.97186 | -3.2879 |
| H | 5.039205 | 0.464027 | -3.46374 |
| H | 5.483983 | 1.654984 | -2.22754 |
| O | -0.87595 | 0.17138 | 1.383942 |
| C | -3.23662 | -0.59127 | 1.337481 |
| C | -3.96211 | 0.848558 | -0.37859 |
| H | -4.17107 | 0.469569 | -1.38588 |
| H | -4.53499 | 1.772522 | -0.24038 |
| N | -4.32374 | -0.14628 | 0.62944 |
| O | -3.21929 | -1.49097 | 2.169728 |
| C | -5.6069 | -0.82852 | 0.63336 |
| H | -5.64417 | -1.40351 | 1.560251 |
| H | -6.40743 | -0.08561 | 0.664107 |
| C | -5.77858 | -1.73901 | -0.56513 |
| C | -4.91342 | -2.82451 | -0.74392 |
| C | -6.77064 | -1.4989 | -1.51536 |
| C | -5.04277 | -3.65346 | -1.85326 |
| H | -4.14218 | -3.01617 | -0.00622 |
| C | -6.90399 | -2.33043 | -2.6278 |
| H | -7.44207 | -0.65662 | -1.38601 |
| C | -6.03977 | -3.4082 | -2.79909 |
| H | -4.3694 | -4.49328 | -1.98035 |
| H | -7.67903 | -2.13289 | -3.35931 |
| H | -6.14021 | -4.05465 | -3.66309 |
| C | -1.49624 | 3.344532 | -0.10946 |
| C | -1.36898 | 4.490541 | -0.89887 |

| | | | |
|---|---|---|---|
| C | -1.57531 | 3.496084 | 1.278448 |
| C | -1.28434 | 5.754487 | -0.31879 |
| H | -1.33061 | 4.392572 | -1.97806 |
| C | -1.49079 | 4.75705 | 1.862053 |
| H | -1.69928 | 2.624404 | 1.907869 |
| C | -1.33749 | 5.891413 | 1.066215 |
| H | -1.18106 | 6.630237 | -0.94908 |
| H | -1.54727 | 4.853558 | 2.940193 |
| H | -1.27131 | 6.87271 | 1.521175 |
| H | -0.01479 | -1.45899 | -0.38167 |

**5ba’**

Optimization energy : E(B3LYP-D3/6-311G(d,p)/IEFPCM(DCM)) = -1919.184302 Hartree

Single point energy : E(M06-2X-D3/def2-TZVPP/SMD(DCM))= -1918.501602 Hartree

| | | | |
|---|---|---|---|
| C | 0.521122 | 1.296483 | 1.165986 |
| C | -0.297 | 2.405575 | 1.871122 |
| C | -1.25312 | 3.200607 | 0.9777 |
| C | -2.17203 | 2.255324 | 0.205959 |
| C | -1.32878 | 1.294671 | -0.63286 |
| C | -0.34176 | 0.443732 | 0.178805 |
| H | -1.83857 | 3.879523 | 1.604558 |
| H | -0.8869 | 1.954037 | 2.667376 |
| H | 0.408962 | 3.079611 | 2.363382 |
| H | 0.857798 | 0.604719 | 1.942143 |
| H | -2.81538 | 1.708089 | 0.900168 |
| H | -2.83354 | 2.817447 | -0.45869 |
| H | -1.95266 | 0.639687 | -1.24273 |
| H | -0.73168 | 1.882224 | -1.33247 |
| H | -0.69801 | 3.82032 | 0.270092 |
| C | 1.774629 | -0.16724 | -0.81242 |
| C | 2.510532 | 0.750245 | -0.18319 |
| C | 1.877385 | 1.848254 | 0.592632 |
| H | 2.480867 | 2.067542 | 1.479266 |
| C | -1.00885 | -0.7751 | 0.940897 |
| C | -1.77536 | -0.52837 | 2.212888 |
| C | -2.07518 | -1.40178 | 0.032406 |
| H | -0.20141 | -1.49278 | 1.090116 |
| C | -3.14523 | -0.70116 | 1.97907 |

| | | | |
|---|---|---|---|
| C | -4.08633 | -0.52912 | 2.985965 |
| H | -5.13715 | -0.66427 | 2.788293 |
| C | -1.32672 | -0.26311 | 3.49766 |
| H | -0.26666 | -0.18384 | 3.704477 |
| C | -3.6188 | -0.20537 | 4.262582 |
| H | -4.33552 | -0.06648 | 5.063166 |
| C | -2.25606 | -0.09124 | 4.526204 |
| H | -1.91368 | 0.126209 | 5.530398 |
| O | -1.9072 | -2.03055 | -0.97485 |
| N | -3.33979 | -1.10189 | 0.626988 |
| C | -4.60037 | -1.23614 | 0.018642 |
| O | -5.62906 | -1.27757 | 0.655139 |
| O | -4.47237 | -1.25706 | -1.29815 |
| C | -5.63276 | -1.50996 | -2.19166 |
| C | -6.24552 | -2.86801 | -1.86076 |
| C | -6.63141 | -0.36279 | -2.06651 |
| C | -4.97613 | -1.52431 | -3.56743 |
| H | -5.47873 | -3.64515 | -1.89252 |
| H | -6.71147 | -2.86545 | -0.87696 |
| H | -7.00626 | -3.10641 | -2.60728 |
| H | -6.133 | 0.592528 | -2.24577 |
| H | -7.41438 | -0.48657 | -2.81831 |
| H | -7.09095 | -0.34559 | -1.08015 |
| H | -5.7304 | -1.71202 | -4.33394 |
| H | -4.4995 | -0.56406 | -3.77338 |
| H | -4.21808 | -2.30751 | -3.6188 |
| O | 0.438242 | -0.26923 | -0.84574 |
| C | 2.644675 | -1.11839 | -1.55981 |
| C | 3.959488 | 0.511579 | -0.48288 |
| H | 4.540009 | 0.276575 | 0.417187 |
| H | 4.429005 | 1.374066 | -0.97014 |
| N | 3.917316 | -0.63607 | -1.38282 |
| O | 2.320438 | -2.11636 | -2.18794 |
| C | 5.106107 | -1.37034 | -1.77822 |
| H | 4.783396 | -2.08535 | -2.53721 |
| H | 5.817069 | -0.68203 | -2.24199 |
| C | 5.754922 | -2.0884 | -0.61276 |
| C | 5.051973 | -3.08991 | 0.066409 |

| | | | |
|---|---|---|---|
| C | 7.037913 | -1.75046 | -0.18299 |
| C | 5.626148 | -3.74097 | 1.153204 |
| H | 4.054897 | -3.35743 | -0.2654 |
| C | 7.617072 | -2.40343 | 0.905541 |
| H | 7.587209 | -0.97165 | -0.70142 |
| C | 6.911883 | -3.39896 | 1.57597 |
| H | 5.07426 | -4.518 | 1.669499 |
| H | 8.615022 | -2.13137 | 1.229497 |
| H | 7.358938 | -3.90707 | 2.422422 |
| C | 1.804136 | 3.143314 | -0.2079 |
| C | 2.014453 | 4.366927 | 0.433883 |
| C | 1.530104 | 3.148718 | -1.57869 |
| C | 1.906941 | 5.568638 | -0.26211 |
| H | 2.258984 | 4.379598 | 1.490177 |
| C | 1.421129 | 4.347226 | -2.2787 |
| H | 1.404171 | 2.210158 | -2.10397 |
| C | 1.600276 | 5.563092 | -1.62106 |
| H | 2.067273 | 6.50711 | 0.255955 |
| H | 1.200719 | 4.330572 | -3.33979 |
| H | 1.515412 | 6.496052 | -2.16571 |
